# Coordinated Cellular Neighborhoods Orchestrate Antitumoral Immunity at the Colorectal Cancer Invasive Front

**DOI:** 10.1016/j.cell.2020.07.005

**Published:** 2020-09-03

**Authors:** Christian M. Schürch, Salil S. Bhate, Graham L. Barlow, Darci J. Phillips, Luca Noti, Inti Zlobec, Pauline Chu, Sarah Black, Janos Demeter, David R. McIlwain, Nikolay Samusik, Yury Goltsev, Garry P. Nolan

**Affiliations:** 1 Department of Microbiology & Immunology, Stanford University School of Medicine, Stanford, CA 94305, USA; 2 Department of Pathology, Stanford University School of Medicine, Stanford, CA 94305, USA; 3 Department of Bioengineering, Stanford University School of Medicine, Stanford, CA 94305, USA; 4 Department of Dermatology, Stanford University School of Medicine, Stanford, CA 94305, USA; 5 Institute of Pathology, University of Bern, 3008 Bern, Switzerland

**Keywords:** antitumoral immunity, cellular neighborhoods, CODEX, colorectal cancer, FFPE, immune checkpoints, immune tumor microenvironment, multiplexed imaging, tertiary lymphoid structures, tissue architecture

## Abstract

Antitumoral immunity requires organized, spatially nuanced interactions between components of the immune tumor microenvironment (iTME). Understanding this coordinated behavior in effective versus ineffective tumor control will advance immunotherapies. We re-engineered co-detection by indexing (CODEX) for paraffin-embedded tissue microarrays, enabling simultaneous profiling of 140 tissue regions from 35 advanced-stage colorectal cancer (CRC) patients with 56 protein markers. We identified nine conserved, distinct cellular neighborhoods (CNs)—a collection of components characteristic of the CRC iTME. Enrichment of PD-1^+^CD4^+^ T cells only within a granulocyte CN positively correlated with survival in a high-risk patient subset. Coupling of tumor and immune CNs, fragmentation of T cell and macrophage CNs, and disruption of inter-CN communication was associated with inferior outcomes. This study provides a framework for interrogating how complex biological processes, such as antitumoral immunity, occur through concerted actions of cells and spatial domains.

## Highlights

• FFPE-CODEX multiplexed tissue imaging of 56 markers in 140 tissues of 35 CRC patients
• Cellular neighborhoods reveal spatial organization of the tumor microenvironment
• Altered organization of tumor and immune components in low- versus high-risk patients
• Local enrichment of PD-1^+^CD4^+^ T cells correlates with survival in high-risk patients

## Graphical Abstract

**Figure:**
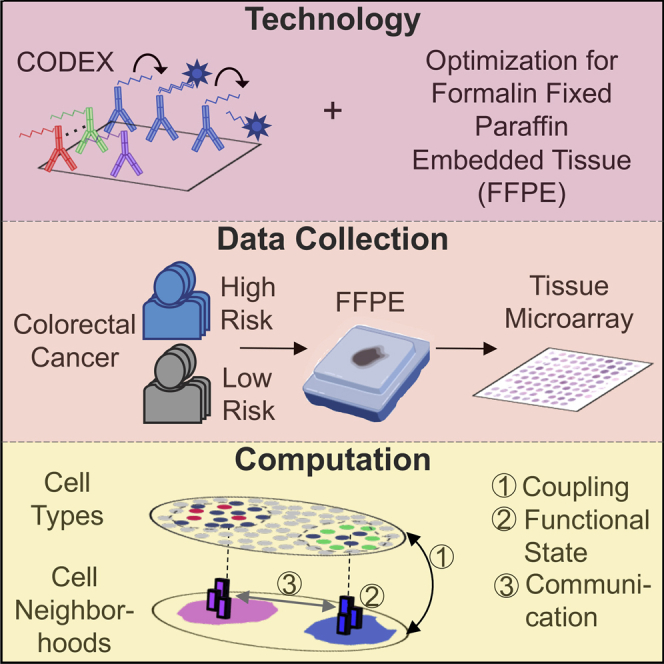

## Teaser

A multiplexed tissue imaging and computational analysis framework applied to colorectal cancer allows interrogation of how spatial organization of the immune tumor microenvironment is linked to clinical outcomes.

## Introduction

The immune tumor microenvironment (iTME) is a complex assembly of tumor, immune, stromal, and extracellular components. Organization of these components at the cellular and tissue levels plays a crucial role in the effectiveness of antitumor immunity (Binnewies et al., 2018). Technological and computational advances have allowed new spatial relationships to be identified, but determining which of these are informative of tissue behavior and, therefore, have the highest clinical utility remains challenging.

Multiplexed imaging technologies allow capturing many parameters of single cells while preserving their spatial location (Agasti et al., 2017; Angelo et al., 2014; Gerdes et al., 2013; Giesen et al., 2014; Gut et al., 2018; Huang et al., 2013; Lin et al., 2018; Moffitt et al., 2018; Saka et al., 2019; Schubert et al., 2006; Wang et al., 2017b, 2018; Wei et al., 2017). We previously introduced co-detection by indexing (CODEX), a technology that uses DNA-conjugated antibodies and iterative polymerase extension with fluorescent nucleotides and chemical fluorophore cleavage to achieve high-parameter immunofluorescence imaging of fresh-frozen tissue. Among other applications, we demonstrated that the identity of cells could often be predicted simply by their neighbors, implying mutual information relationships that link cell marker expression and tissue localization. In addition, these neighbor relationships were predictive of disease progression in a murine model of autoimmunity (Goltsev et al., 2018). To better study the iTME in cancer patients with long-term clinical follow-up, including survival data, we re-engineered the CODEX method to be compatible with formalin-fixed, paraffin-embedded (FFPE) tissue and tissue microarrays (TMAs).

Focusing on the iTME, other investigators applying multiplexed imaging have explored cellular functional states, spatial and functional interactions between multiple cell types, as well as how these interactions are modulated by tissue context (Ali et al., 2020; Arnol et al., 2019; Jackson et al., 2020; Keren et al., 2018; Schapiro et al., 2017; Stoltzfus et al., 2019; Zhu et al., 2018). We reasoned that viewing the tissue only with respect to interacting cell types (CTs) limits the ability to capture the tissue-level processes that emerge from such spatial relationships. Tissue biology is organized into at least two levels, distinct tissue regions and the CTs of which they are composed; therefore, in our representation of the tissue, we sought to reflect coordination at both levels. By analogy, the iTME is like a city composed of neighborhoods (e.g., industrial, residential, or agricultural), which are regions where specific functions of the city occur. These neighborhoods are distinguished by their composition of buildings, activities, and people, but they exhibit behavior of their own, such as industrial output or energy consumption. At a more granular level, people (e.g., teachers, doctors, and construction workers) play integral roles in the city’s function. The same concept applies when studying tissue. Identifying these different levels and integrating information between them allows us to pose novel questions about the tissue’s behavior. How do certain CTs perform specialized roles to accomplish specific tissue processes? How do specific tissue regions interact with each other? How do different types of tissues compare? We developed experimental and computational methodologies to address these questions. We then applied them to the iTME of human colorectal cancer (CRC) because it has long been recognized in CRC that distinct, histologically observed forms of its spatial architecture are linked to prognosis. Because CRC is a leading cause of cancer deaths in the Western world (Siegel et al., 2018), it offered a compelling clinical question and an opportunity to determine whether higher-level abstractions of tissue architecture in the iTME could allow inference of underlying mechanisms driving outcomes.

Is there evidence that neighborhoods as described above are functionally relevant in the CRC tissue context? One spatial structure that could be interpreted as a neighborhood is the tertiary lymphoid structure (TLS). The biological and clinical relevance of TLSs has been demonstrated not only in CRC but also in multiple other cancers (Binnewies et al., 2018; Cabrita et al., 2020; Di Caro et al., 2014; Dieu-Nosjean et al., 2016; Graham and Appelman, 1990; Helmink et al., 2020; Petitprez et al., 2020; Sautès-Fridman et al., 2016). To study TLSs and additional, currently unknown neighborhoods that could affect the behavior of the iTME in CRC, we selected two patient groups from opposite ends of a spectrum of iTME architectures. One group exhibited *de novo* formation of numerous TLSs at the tumor invasive front—the “Crohn’s-like reaction” (CLR). The other group was defined by the absence of TLSs and the presence of diffuse inflammatory infiltration (DII). Overall survival of patients with CLR is much longer than that of patients with DII, as reported previously (Di Caro et al., 2014; Graham and Appelman, 1990). The presence of TLSs in CLR patients and their absence in DII patients indicates that the differences in survival between these patient groups are likely influenced by differences in their antitumoral immune responses as opposed to only tumor-intrinsic factors. We reasoned that comparing the iTME of CLR and DII patients using highly multiplexed tissue imaging would allow us to identify new neighborhoods beyond the TLS whose behavior could be associated with effective or ineffective antitumoral immunity.

**Figure 1:**
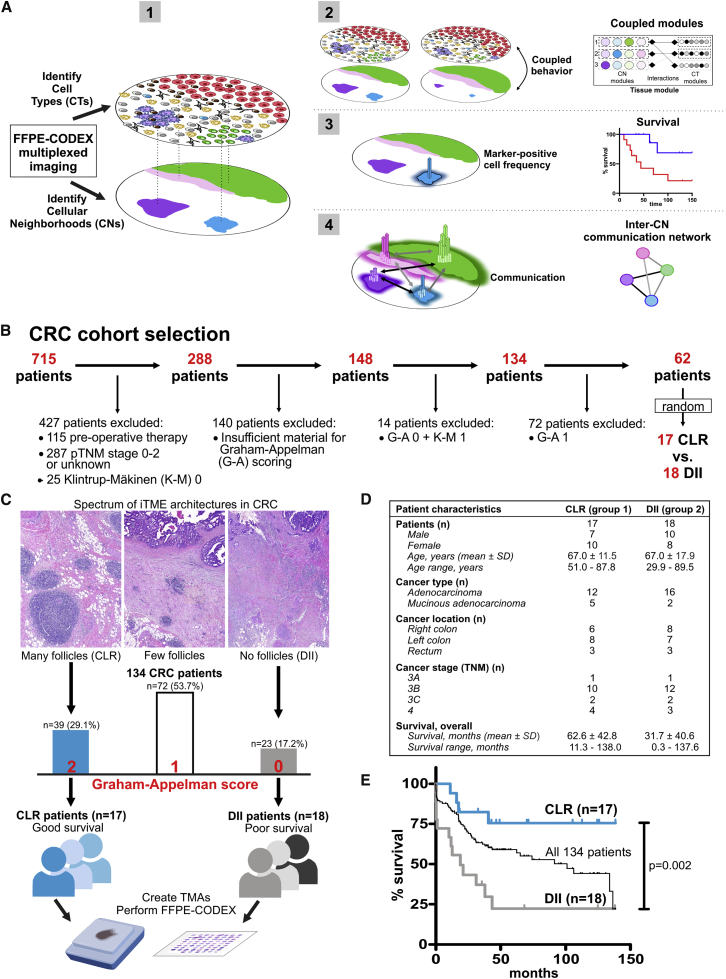
CRC Study Cohort (A) Conceptual framework. (B) Exclusion criteria: pre-operative therapy, pathological tumor, nodes, metastasis (pTNM) score 0–2 or unknown, absent immune infiltration (Klintrup-Mäkinen [K-M] score 0), insufficient material for Graham-Appelman (G-A) scoring, a combination of low immune infiltration (K-M 1) and absent follicles (G-A 0) or few follicles (G-A 1). (C) Spectrum of iTME architectures in 134 advanced-stage CRC patients. (D) Characteristics of patients in the CRC study cohort. (E) Kaplan-Meier survival curve of the CRC study cohort (p determined with a log-rank test). See also Table S1A and STAR Methods.

To maximize our ability to identify novel tissue behaviors in these opposing phenotypes, we constructed TMAs that specifically represented the iTME at the CRC invasive front. We imaged these TMAs with 56 markers that would recapitulate cellular phenotypes established to be associated with antitumoral immunity in solid cancers, such as different T cell and macrophage subsets (Joyce and Fearon, 2015). We identified CTs in each patient sample and identified cellular neighborhoods (CNs) as regions with a characteristic local stoichiometry of CTs (Figure 1A.1). Thus, a patient sample can be viewed simultaneously as a collection of CTs and as a collection of CNs.

In our model, a CN can retain its identity even when the individual cells within it change (like a cell retains its identity even when the molecules within it change). We would expect changes in spatial regions corresponding to CTs and CNs to be correlated (Figure 1A.2, left). Understanding the dynamic processes in the iTME therefore requires understanding them from the view of CTs, from the view of CNs, as well as how these two views are coupled. In gene expression data, principal-component analysis (PCA) decomposes variation in samples to reveal the organization of genes into modules. We use tensor techniques to decompose variation in samples to reveal organization from the view of CTs, organization from the view of CNs, as well as how the organization from these two views is coupled (Figure 1A.2, right).

The functional states of cells can be identified by key markers and are associated with patient outcomes (Hashimoto et al., 2018). Therefore, the frequency of a given marker-positive cell subset within a CN could be an indicator of its functional state. Moreover, the functional state of a CN could be associated with survival even when the frequency across the tissue of the CT defining that functional state is not (Figure 1A.3).

If the functional state of one CT were correlated, across patients, with the functional state of another, one could infer that something is mediating this correlation; i.e., these CTs “communicate.” The same holds true for CNs and enables inference of an inter-CN communication network (Figure 1A.4). Alterations between patient groups in inter-CN communication could reveal insights into how the tumor modulates the iTME and possibilities for therapeutic intervention.

Using this analytical framework, we identified 28 CTs and 9 CNs that recapitulated known tissue components, such as the TLS and the tumor, as well as novel ones. Tensor decomposition identified differences in the coupling of CTs and CNs between patient groups and suggested that a granulocyte-enriched CN could play a significant role in DII patients. Indeed, the frequency of PD-1^+^CD4^+^ T cells within this CN was correlated with survival in DII patients whereas the overall frequency of these cells was not. Furthermore, the tensor decomposition indicated that a T cell-enriched CN is coupled to a macrophage-enriched CN in DII patients but not in CLR patients. Investigating these CNs further, we found that the Ki-67^+^CD8^+^ T cell density in the T cell-enriched CN was anti-correlated with the frequency of regulatory T (Treg) cells in the macrophage-enriched CN in DII but not CLR patients. These findings suggest that CNs and their correlations could play a functional role in the iTME. We further identified a communication network of CNs based on the correlations in the frequencies of functional T cell subsets within them.

Our experimental and analytical techniques provide a generalizable framework for interrogating the spatial behavior of a given cancer’s iTME. Applied to large patient cohorts, it could reveal how tumors modulate the organization, functional states, and intercommunication of CNs to evade antitumoral immunity.

## Results

### Selection of Patient Samples by Classically Determined iTME Structures in CRC

The native immune infiltrates and associated histologic features at the CRC invasive front exhibit a spectrum of iTMEs from CLR with many TLSs to DII with no TLSs. From a database of 715 CRC patients, we selected cohorts of 17 CLR and 18 DII patients and constructed TMAs of the iTME at the tumor invasive front (Figures 1B and 1C). The two groups were matched with regards to gender, age, cancer type, location, and stage (Figure 1D; Table S1A). In line with previous reports, the overall survival of CLR patients was significantly better than that of DII patients (Figure 1E).

### FFPE-Optimized CODEX Enables Highly Multiplexed Fluorescence Microscopy of Archival Human Cancer Samples

**Figure 2:**
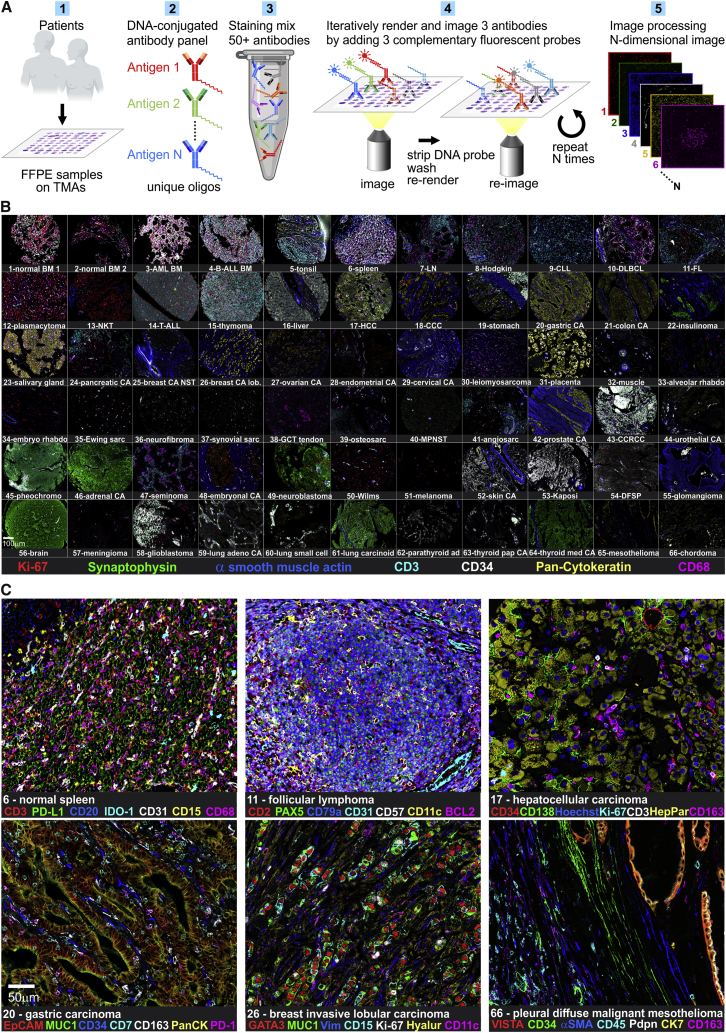
CODEX Workflow and Antibody Validation (A) CODEX workflow. (B) Seven-color overview of multi-tumor TMA, imaged using a 56-marker CODEX panel. (C) Higher-magnification seven-color images of select tissue cores. See also Figures S1 and S2, Data S1 and S2A, and Tables S1B–S1E.

**Figure S1:**
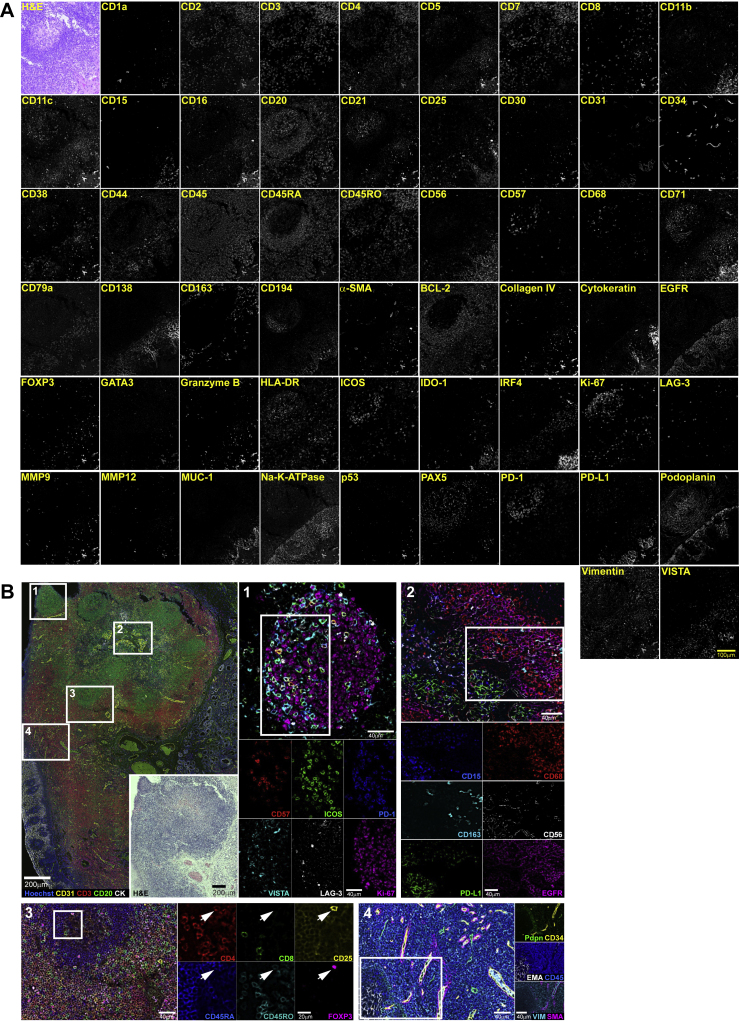
Validation and Titration of the CODEX Antibody Panel, Related to Figure 2, Table S1, and STAR Methods FFPE tonsil tissue was stained with a cocktail of 55 different DNA-conjugated antibodies, and a multi-cycle experiment was performed followed by H&E staining. (A) Images of a single tissue region at the interface of a follicle (top left in each image) and epithelium (bottom right in each image) are depicted in false gray color for each antibody; H&E staining is also shown. Scale bar, 100 μm. (B) Top left panel: Overview of the tonsil section in a five-color overlay image with Hoechst (blue; nuclei), CD31 (yellow; vasculature), CD3 (red; T cells), CD20 (green; B cells), and pan-cytokeratin (CK, white; epithelium). Inset: H&E staining. Scale bars, 200 μm. Regions 1-4 are indicated by white rectangles. Region 1: Six-color overlay and single-marker images of a follicle with CD57 (red), ICOS (also known as CD278, green), PD-1 (also known as CD279, blue), VISTA (cyan), LAG-3 (also known as CD223, white), and Ki-67 (magenta). Scale bars, 40 μm. Region 2: Six-color overlay and single-marker images of an inflamed epithelial-lymphoid parenchyma interface with CD15 (blue), CD68 (red), CD163 (cyan), CD56 (white), PD-L1 (also known as CD274, green), and EGFR (magenta). Scale bars, 40 μm. Region 3: Six-color overlay and single-marker images of a follicle with CD4 (red), CD8 (green), CD25 (yellow), CD45RA (blue), CD45RO (cyan), and FOXP3 (magenta). A CD4^+^CD25^hi^FOXP3^+^CD45RO^+^ regulatory T cell is indicated by the white arrow. Scale bars, 40 μm and 20 μm, respectively. Region 4: Six-color and two-color overlay images of an epithelial region with Pdpn (green), CD34 (yellow), EMA (also known as MUC-1, white), CD45 (blue), vimentin (cyan), and SMA (magenta). Scale bars, 40 μm. Brightness and contrast adjusted.

**Figure S2:**
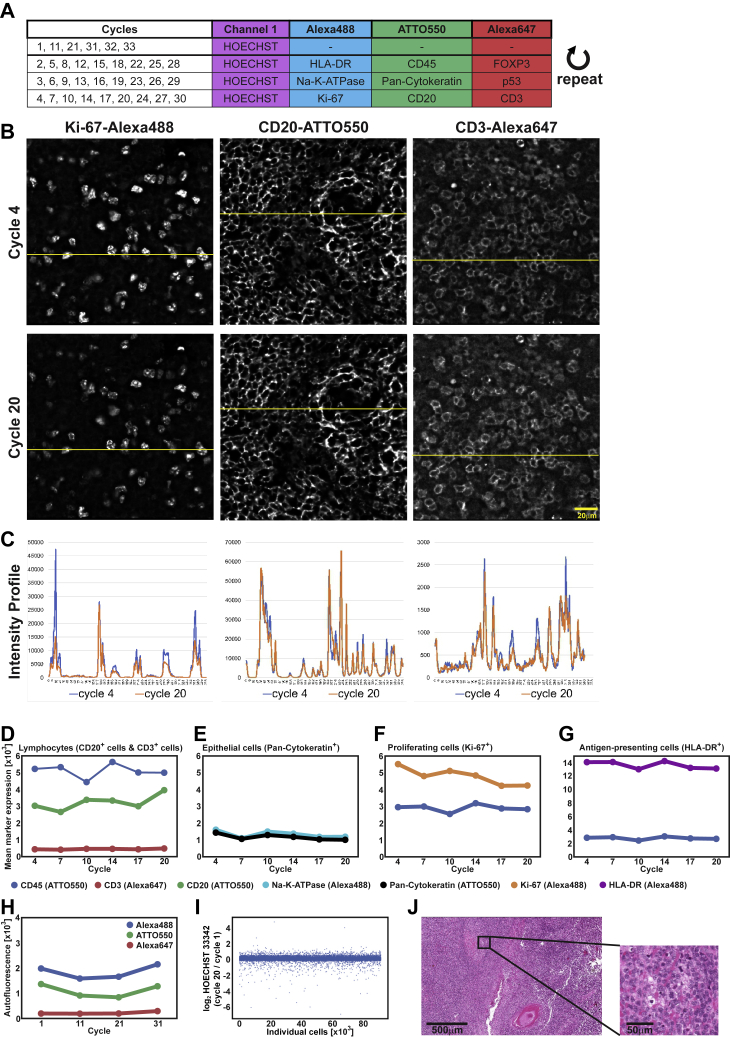
Signal and Tissue Integrity and Autofluorescence during the CODEX Multi-Cycle Experiment, Related to Figure 2, Table S1, and STAR Methods (A) FFPE tonsil tissue was stained with a cocktail of nine different DNA-conjugated antibodies. Antibodies were repeatedly rendered visible using complementary fluorescent oligonucleotides in 33 cycles with blank cycles to measure autofluorescence (no fluorescent oligonucleotides added) at the beginning (cycle 1), after each round of nine antibody rendering (cycles 11 and 21), and at the end (cycles 31, 32 and 33). The microscope light exposure times were kept constant for each antibody in each cycle. Hoechst nuclear stain was used as a reference marker. (B) Example images of nuclear marker Ki-67-Alexa488 and membrane markers CD20-ATTO550 and CD3-Alexa647 in cycles 4, and 20 are shown. Images are representative of cycles and nuclear and membrane markers that are not shown. Scale bar, 20 μm. Brightness and contrast adjusted. (C) Comparison of fluorescence intensity profiles from cycles 4 and 20, as measured by ImageJ software on the yellow lines in panel B. (D-I) Cells were segmented using the CODEX toolkit and clustered using X-shift (VorteX). (D) Mean marker expression for CD45 (ATTO550), CD20 (ATTO550), and CD3 (Alexa647) on lymphocytes (combined CD20^+^ cells and CD3^+^ cells). (E) Mean marker expression for Na-K-ATPase (Alexa488) and pan-cytokeratin (ATTO550) on epithelial cells (pan-cytokeratin^+^ cells). (F) Mean marker expression for Ki-67 (Alexa488) and CD45 (ATTO550) on proliferating cells (Ki-67^+^ cells). (G) Mean marker expression for HLA-DR (Alexa488) and CD45 (ATTO550) on antigen-presenting cells (HLA-DR^+^ cells). For lymphocytes, epithelial cells, and proliferating cells, 1500 cells were sampled; for antigen presenting cells, > 250 cells were sampled. (H) Mean autofluorescence levels on all cells combined measured in each channel in blank cycles 1, 11, 21 and 31 (no fluorescent DNA probes added). (I) Mean expression of the nuclear marker Hoechst per cell in cycle 20 versus cycle 1. (J) Representative image of H&E staining performed after the last blank cycle 33.

We re-engineered and fully automated the CODEX platform to use iterative annealing, imaging, and stripping of fluorescently labeled DNA probes complementary to DNA barcodes on tissue-bound antibodies. We further optimized CODEX for use in FFPE tissue to study archival human samples (Figure 2A). We performed extensive testing of the iterative imaging approach and individual antibodies and validated our combined CODEX antibody panel by staining and analyzing a multi-tumor TMA (Figures 2B, S1 and S2; Tables S1B–S1E, Data S1 and S2A, and STAR Methods). Expected antigen distribution patterns were observed in all tissues analyzed, exemplified by normal spleen, follicular lymphoma, hepatocellular carcinoma, gastric carcinoma, breast invasive lobular carcinoma, and pleural diffuse malignant mesothelioma (Figure 2C). For example, spleen tissue showed a normal distribution of red and white pulp (CD3, CD20, CD68), presence of granulocytes (CD15), localization of indoleamine 2,3-dioxygenase 1 (IDO-1) in red pulp macrophages, and prominent PD-L1 expression in splenic sinusoids (CD31). Notably, we detected a strong and ubiquitous expression of the T cell checkpoint marker V domain Ig suppressor of T cell activation (VISTA) in mesothelioma, which is confirmed as a feature of mesothelioma in a recent integrative genomic characterization study (Hmeljak et al., 2018). Collectively, these data demonstrate that FFPE-optimized CODEX is suitable for highly multiplexed single-cell marker visualization, quantification and biomarker discovery in clinically relevant tissues.

### FFPE-CODEX Enables *In Situ* Identification and Quantification of Major Immune CT in the iTME of the CRC Invasive Front

**Figure 3:**
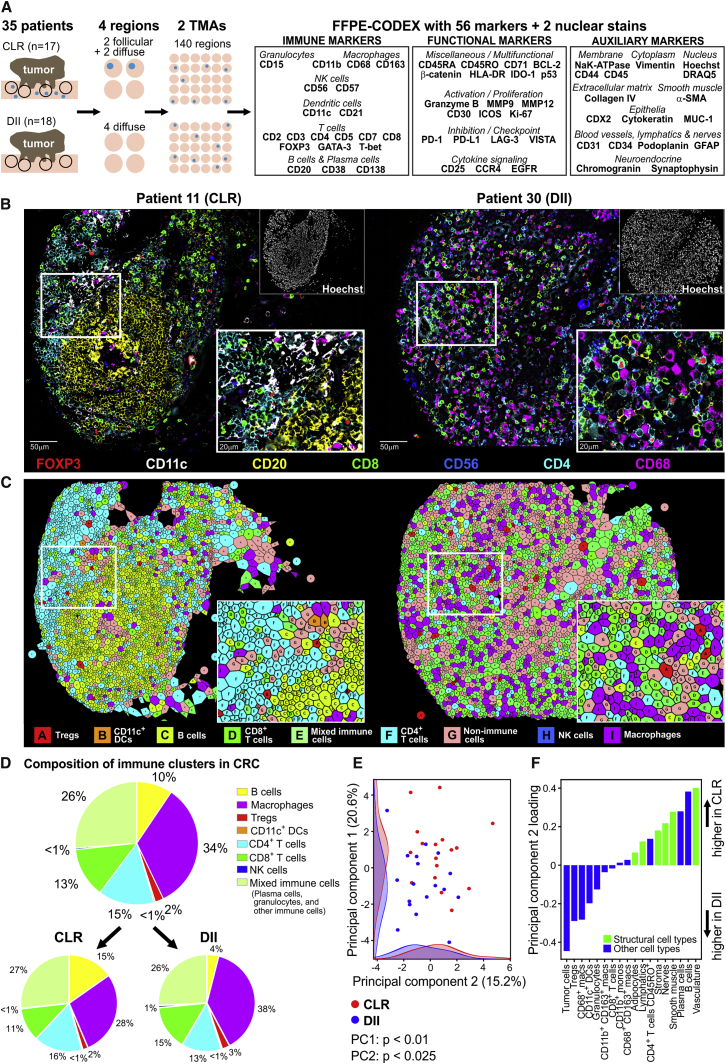
Spatial Composition of Immune Infiltrates in CRC (A) Schematic of CRC TMA assembly. Blue dots represent follicles/TLSs. (B) Representative TMA cores for CLR and DII patients depicted as seven-color images. (C) Voronoi diagrams of clustered CTs, merged to reduce complexity. (D) The eight immune clusters (n = 132,437 cells) and their frequencies in all CRC patients (top) and separated into CLR (n = 57,894 cells) and DII patients (n = 74,543 cells) (bottom). (E) PCA of CT abundances in CLR versus DII patients. (F) CT loadings in principal component 2. See also Figure S3, Figure S4A, and S4B, Data S2B, S2C, and S3–S6, and Table S1F.

**Figure S3:**
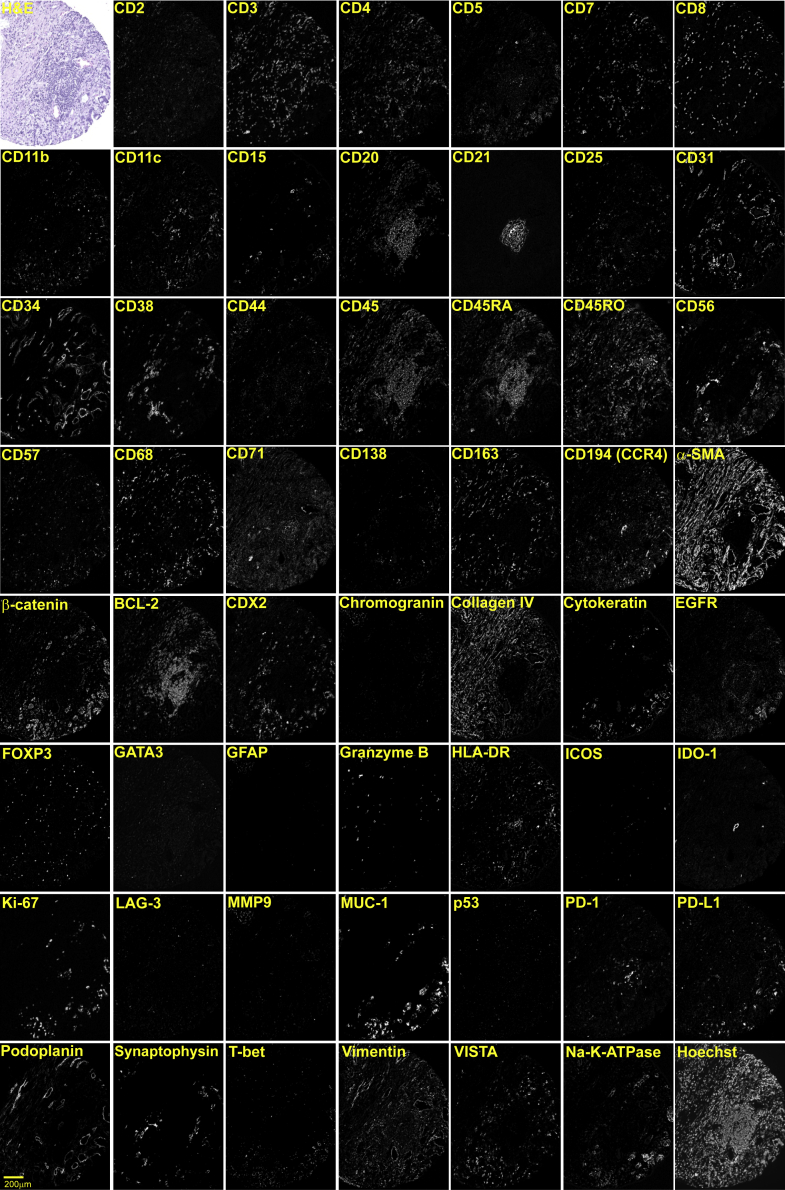
CRC CODEX Antibody Panel, Related to Figure 3, Table S1, and STAR Methods Each marker of the CRC CODEX panel is depicted individually for one representative TMA spot (spot 36 of TMA 1; patient 18). CD30 and MMP12 were not detectable in any of the spots in either TMA, and are therefore not depicted. H&E and Hoechst stainings are shown for morphological reference. Scale bar, 200 μm. Brightness and contrast adjusted.

At the CRC invasive front, the iTME is usually seen as leukocyte-dense regions alternating with regions of sparse immune infiltration. In DII patients, TLSs (follicles) are absent in the immune infiltrate but abundant in tumors from CLR patients. We performed 56-marker CODEX on two specifically created high-precision TMAs incorporating four representative leukocyte-dense iTME regions from the tumor invasive front for each patient, resulting in a total of 140 regions (Figures 3A, 3B, and S3; Data S2B and S2C).

By unsupervised clustering using X-shift (Samusik et al., 2016) followed by supervised cluster merging based on marker expression profiles, tissue localization, and morphology, we identified and validated 28 unique CT clusters. These included 18 immune cell clusters, 6 stromal and vasculature clusters, 2 mixed clusters, 1 tumor cell cluster, and 1 undefined cluster (Data S3 and S4; STAR Methods). Supervised manual gating and unsupervised X-shift clustering led to comparable results for absolute numbers and frequencies of defined major immune CTs (Data S5; Table S1F). For downstream analyses, we chose the CTs identified using unsupervised clustering.

We visualized the CTs using Voronoi diagrams, merging clusters to simplify visualization (Figure 3C; Data S6A). We quantified each immune CT as a fraction of total immune cells on an overall per-group and per-patient basis. The frequency of immune subsets across all patients revealed CTs with high (i.e., macrophages, 34%), medium (i.e., CD4^+^ T cells, 15%; CD8^+^ T cells, 13%; B cells, 10%), and low frequencies (i.e., Treg cells, 2%; natural killer [NK] cells, <1%; CD11c^+^ dendritic cells [DCs], <1%) (Figure 3D; Data S6B–S6F).

Differences in the composition of these immune cell clusters were observed between CLR and DII patients. Most notably, CLR patients had higher frequencies of B cells, whereas DII patients had higher frequencies of macrophages. Interestingly, the frequencies of CD8^+^ T cell and Treg cell clusters were not significantly different between CLR and DII patients (Figure 3D; Data S6E and S6F). No differences in the frequencies of tumor cell, smooth muscle, stroma, and vasculature clusters were observed (Data S6B–S6D).

**Figure S4:**
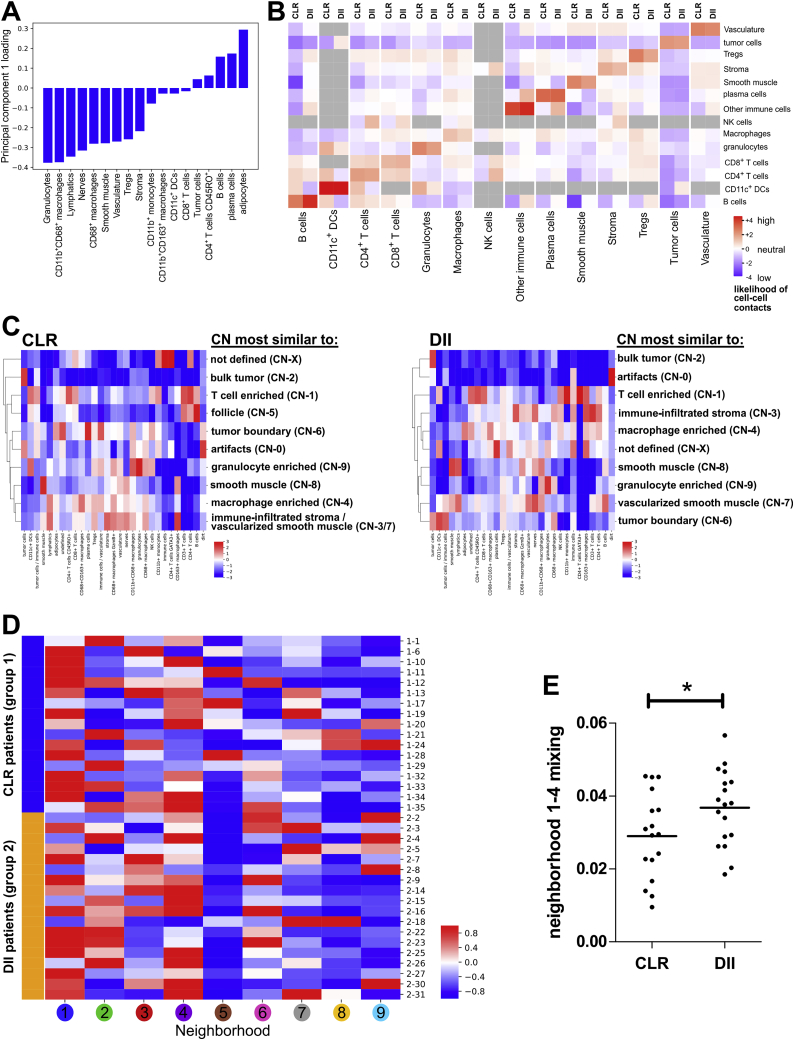
Pairwise Cell-Cell Contacts and CNs in Both Patient Groups, Related to Figure 4 (A) PCA correlating combinations of cell-type abundances in CLR versus DII patients. Cell-type loading in principal component 1 is shown. (B) Heatmap of likelihood ratios of direct cell-cell contacts for 14 selected clusters is shown for clusters with at least 100 unique interacting cells. Gray boxes indicate less than 100 unique interacting cells; these data were omitted. Pooled data from all TMA cores are shown. (C) Both CLR and DII patient groups were clustered separately. CNs were annotated manually. CN-0 corresponds to the imaging artifacts cluster; this cluster was omitted from the analysis shown in Figure 4B. Neighborhoods that did not have matching counterparts in the analysis of the combined groups are labeled “not defined.” (D) Frequencies of each CN from Figure 4B in each patient are shown. Frequencies are z scored by column to highlight major differences between CLR patients (blue) and DII patients (orange). (E) The contacts between CN 1 (T cell-enriched) and CN 4 (macrophage-enriched) were computed (see Methods) and are displayed as “CN mixing” by patient group. For each patient, the mean mixing score of four TMA cores is shown (^∗^p < 0.05, Student’s t test).

We performed PCA to identify combinations of CTs driving variation and assessed their prevalence across patient groups. The first two principal components were more prevalent in CLR patients than in DII patients (t test, p < 0.01 and p < 0.025, respectively; Figure 3E). The first principal component had a positive weight for cell populations that are found in a classically defined follicle (B cells, plasma cells, and CD4^+^ T cells) (Figure S4A). Interestingly, in the second principal component, non-immune and non-tumoral cell clusters that make up structural components, such as vessels and connective tissue, had a positive weight (Figure 3F).

A CODEX image can be analyzed for the spatial coordinates of each cell within the tissue. We computed pairwise cell-cell contact frequencies and frequency-normalized contact likelihood ratios (log-odds ratios) (Goltsev et al., 2018) for cell clusters in both groups of patients. The most dominant pairwise cell-cell contacts were homotypic (e.g., B cells with B cells; DCs with DCs), and we did not observe significant differences in these contacts between CLR and DII patients (Figure S4B). This suggested that higher-order features of tissue architecture might be required to distinguish patient groups.

### Characteristic CNs of the CRC iTME Are Conserved across Patient Groups

**Figure 4:**
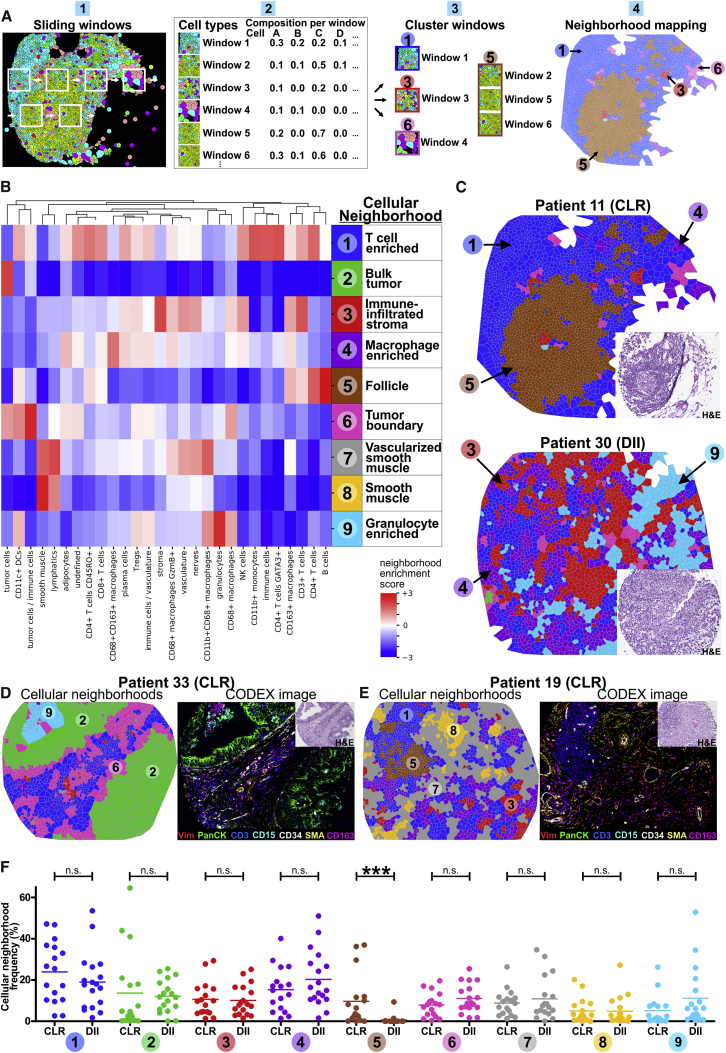
Characteristic CNs of the CRC iTME (A) Schematic of CN identification. (B) Identification of 9 distinct CNs based on the 28 original CTs and their respective frequencies (enrichment score) within each CN (pooled data from both patient groups). (C) Representative Voronoi diagrams of CNs for CLR and DII patients. Insets, H&E images. (D and E) Representative Voronoi diagrams of CNs were selected to show the nine different CNs (left panels) and corresponding seven-color images (right panels) in patient 33 (D) and 19 (E). Insets, H&E images. (F) Frequencies of CNs in CLR versus DII patients. Each point represents the mean CN frequency from four TMA cores per patient, and horizontal lines represent the means across patients (^∗∗∗^p < 0.001, Student’s t test). See also Figures S4C and S4D and Data S2D–S2G.

We reasoned that viewing patient samples in terms of spatial structures formed by cells and not just as collections of single cells would provide insights into the processes organizing the iTME. The simplest possible extension to treating an entire patient sample as a homogeneous collection of single cells (i.e., viewing it in terms of its CT composition) was to view the tissue in terms of CNs. We identified CNs as regions of the tissue with a specific local density of various CTs. Many other, more complicated local features could be used to identify CNs, but this might be less biologically interpretable. For every cell in the tissue, its 10 nearest spatial neighbors, which we labeled its “window,” were identified (Figure 4A.1). The CT composition, with respect to the 28 CTs, was determined per window (Figure 4A.2), and windows were clustered (Figure 4A.3). The tissue was colored with respect to CNs (Figure 4A.4).

CNs were identified by clustering data from both patient groups together to maximize recognition of common CNs. We found 10 distinct CNs in the CRC iTME that recapitulated core tissue components, as validated on the original H&E-stained sections and fluorescence images for both patient groups (Figures 4B–4E; Data S2D–S2G). One of the 10 CNs was comprised mainly of imaging artifacts and, therefore, was removed from further analyses (data not shown). Except for a CN corresponding to the TLS (follicle), the remaining eight CNs were broadly present in CLR and DII patients. When we applied this algorithm to each patient group separately, the identified CNs were still comparable across patient groups (Figure S4C), and the common CNs were therefore not an artifact of the data-merging process. This led to the preliminary conclusion that the two extremes of the CRC iTME spectrum, although visually distinct as a result of the presence of TLS, shared underlying architectural components that could be defined as CNs.

The nine CNs recapitulated structures that directly related to components of the CRC iTME architecture as observed in H&E-stained tissue sections. For example, CN-5 was enriched for B cells, CD4^+^ T cells, CD4^+^CD45RO^+^ T cells, CD11c^+^ DCs, and CD163^+^ macrophages and depleted of all other CTs. When compared with H&E-stained images, CN-5 nearly perfectly aligned with the TLS (follicle) (Figures 4B and 4C). Additionally, the analysis revealed previously unappreciated substructure in the remaining non-follicular leukocyte-dense tissue regions. Examples included T cell-enriched CN-1, macrophage-enriched CN-4, and granulocyte-enriched CN-9.

The tumor itself was divided into two distinct CNs: CN-2, mainly comprised of tumor cells (“bulk tumor”), and CN-6, which contained tumor cells as well as CD11c^+^ DCs, CD68^+^ macrophages, T cell subsets, and other immune and non-immune CTs (“tumor boundary”) (Figures 4B and 4D). In the stroma, we discriminated three CNs: CN-3 was enriched in immune cells, CN-7 was vascularized smooth muscle, and CN-8 mainly consisted of smooth muscle cells (Figures 4B and 4E). Voronoi maps of CNs aligned well with fluorescent CODEX images and H&E images (Figures 4D and 4E; Data S2D–S2G).

We assessed whether the frequencies of CNs differed between CRC patient groups. Except for CN-5 (follicle), which was highly enriched in CLR patients, none of the other CN frequencies differed significantly (Figures 4F and S4D). This indicates that the CNs we identified were common across patient groups and likely represent conserved tissue compartments of the CRC iTME. This raises the question whether there are other structures and relationships between these CNs that might explain the differences between CLR and DII.

### Coupling of Tumor and Immune Components in DII Patients Associated with Coupling and Fragmentation of T Cell and Macrophage CNs

**Figure 5:**
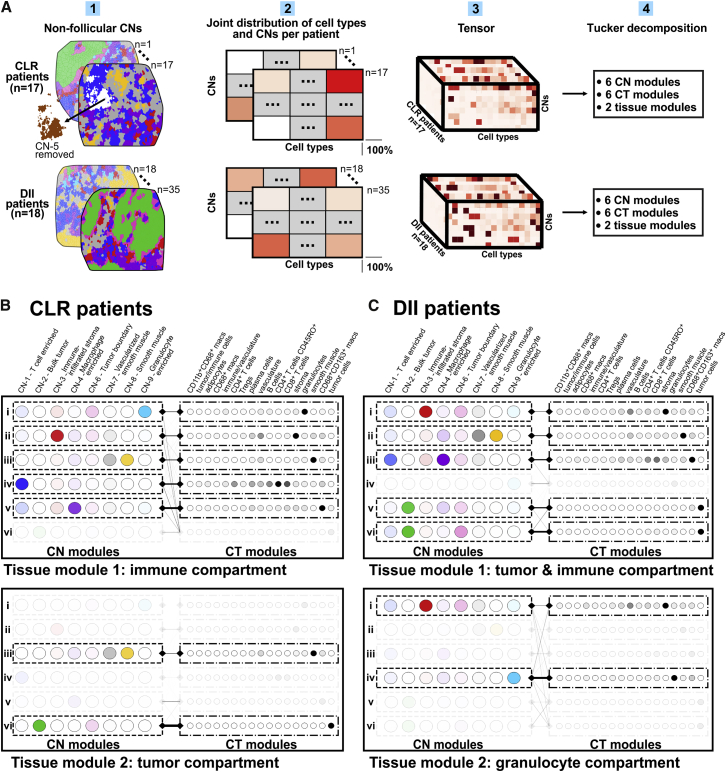
Tensor Decomposition Suggests Differences in Organization of the iTME (A) Schematic of the tensor decomposition analysis. (B) Decomposition results for CLR patients. Tissue modules (interacting pairs of CN modules and CT modules) correspond to an “immune compartment” (top) and a “tumor compartment” (bottom). (C) Decomposition results for DII patients. Tissue modules correspond to an “immune and tumor compartment” (top) and a “granulocyte compartment” (bottom). See also Figure S6C and Data S7.

The CNs we identified provided a different view of patient samples than the CTs. We reasoned that the underlying programs shaping the iTME could be better understood from the CT view and the CN view simultaneously. We therefore considered, for every patient, the joint composition matrix of CNs and CTs, removing data from CN-5 (the follicle) because it was present only in CLR patients (Figures 5A.1 and 5A.2). This matrix not only describes cellular localization at the tissue level (i.e., the probability that a randomly sampled cell of a given type is in a given CN) but also takes into account the abundance of CNs. The variation across patients’ joint CN-CT compositions should therefore provide insight into how the processes in the iTME differ between patient groups from the CN and CT views simultaneously. Tensor techniques achieve this by retaining the information that the joint CN-CT composition for each patient is a 2D matrix and not a 1D vector; the collection of 2D matrices is a 3D “tensor.” Variants of tensor decomposition have been applied previously to gene expression data collected across different tissue types to identify multi-tissue gene networks (Hore et al., 2016). We therefore applied non-negative Tucker tensor decomposition (Kim and Choi, 2007) to the tensor of patients’ joint CN-CT compositions in each patient group separately (Figures 5A.3 and 5A.4**)**. This technique identified a collection of “CN modules” and “CT modules” (from the view of CNs and CTs, respectively) that could be coupled in different ways to form “tissue modules” (from the view of CTs and CNs simultaneously).

CN modules and CT modules are not only combinations of CNs and CTs, respectively, that we could infer to be co-regulated (analogous to gene modules in gene expression data). Instead, combinations of paired CN and CT modules can be interpreted as tissue modules corresponding to programs operating simultaneously at the CN level and CT level. The differences in the decomposition between patient groups (i.e., with respect to which CNs formed CN modules, which CTs formed CT modules, as well as how combinations of paired CN and CT modules formed tissue modules) should therefore reveal differences in the organization of the iTME to further investigate in subsequent analyses. For a detailed explanation and biological interpretation of the tensor decomposition output, see Data S7.

We first describe, at a high level, how the decomposition results from our CRC data are represented. In each patient group, 6 CN modules, 6 CT modules, and 2 tissue modules were identified. Tissue modules (Figures 5B and 5C, solid outer rectangles) combined different pairs of CN and CT modules (Figures 5B and 5C, dashed inner rectangles) interacting to different extents, indicated by the weight of the line (edge) connecting them. CN and CT modules whose edges were below an empirically defined threshold were not deemed to contribute to a given tissue module and were greyed out.

The patient groups differed in the collection of CN modules, collection of CT modules, as well as how these were combined to form tissue modules. In CLR patients, the two tissue modules corresponded to an immune compartment (Figure 5B, top) and a separate tumor compartment (Figure 5B, bottom). In contrast, in DII patients, there was a single compartment containing tumor and immune modules (Figure 5C, top) and, additionally, a distinct granulocyte compartment (Figure 5C, bottom). This suggests that, in DII patients, there is greater coupling between formation of the tumor and the immune tissue compartments than in CLR patients. Although the differences in patient groups are likely due to the actions of both the immune system and the tumor, the results suggested that, in DII patients, the tumor might interfere with or regulate immune processes to a larger extent than in CLR patients. Uniquely, this was not the case for those involving the granulocyte compartment.

We noted other interesting differences between the two patient groups in CN modules and CT modules. In DII patients, one CN module had a high weight for CN-1 (T cell enriched) and CN-4 (macrophage enriched), with its corresponding CT module having a high weight for T cells and macrophages (Figure 5C, row iii). In contrast, in CLR patients, no CN module had a high weight for CN-1 and CN-4, and there was no CT module with a high weight for T cells and macrophages (Figure 5B). This suggested that there might be a common program driving the spatial organization of macrophages and adaptive immune cells into CN-1 (T cell enriched) and CN-4 (macrophage enriched) in DII patients, whereas in CLR patients, there could be distinct programs driving formation of these CNs.

That these two CNs were part of the same CN module in DII patients also suggested that these two modules might be more interdigitated in DII patients than in CLR patients. We therefore computed the number of cells in CN-1 that had a cell in CN-4 as a nearest neighbor and vice versa. We divided this number by the total number of cells in the appropriate CN, estimating the level of their contact or spatial proximity. We found that DII patients had a significantly higher contact between CN-1 (T cell enriched) and CN-4 (macrophage enriched) than CLR patients (p = 0.046, Student’s t test; Figure S4E). Despite this increased surface area of contact, CN-1 and CN-4 did not “merge” but were still distinct CNs and were represented within both patient groups (Figure S4C).

In DII patients, two CN modules had a high weight of CN-2 (bulk tumor): one alongside CN-6 (tumor boundary) and CN-1 (T cell enriched) and one alongside CN-6 and CN-4 (macrophage enriched) (Figure 5C, top, rows v and vi). In contrast, in CLR patients, no CN modules contained a high weight for tumor CNs and other CNs (Figure 5B). This suggests that the molecular programs driving recruitment of cells to the DII tumor neighborhoods (CN-2 and CN-6) could also drive recruitment of cells to CN-1 (T cell enriched) and CN-4 (macrophage enriched). Therefore, the interference by the tumor in the immune processes of the DII iTME may restrict the spatial compartmentalization of T cells and macrophages.

Taken together, the tensor decomposition suggested that there are differences in the underlying programs that result in a distinct spatial organization of the iTME in CLR and DII patients. Moreover, it suggested that, in DII patients, there was increased coupling between the tumor and immune processes specifically associated with increased coupling and spatial contact between T cell and macrophage CNs.

### Neighborhood-Specific Expression of Functional Markers on T Cell Subsets Suggests Altered T Cell Processes in the Bulk Tumor

Understanding the iTME requires appreciating cells not only in terms of simple phenotypic descriptors but also in terms of their functional states, and we would expect the same to be true for CNs. T cells exhibit diverse functional states in the iTME, often indicated by their expression of functional markers. Because the balance of these functional states is essential for a successful antitumoral immune response (Wherry, 2011), we would expect that the relative proportions of T cells expressing different functional markers are indicators of the functional state of a given CN that could be relevant for patient outcomes.

**Figure 6:**
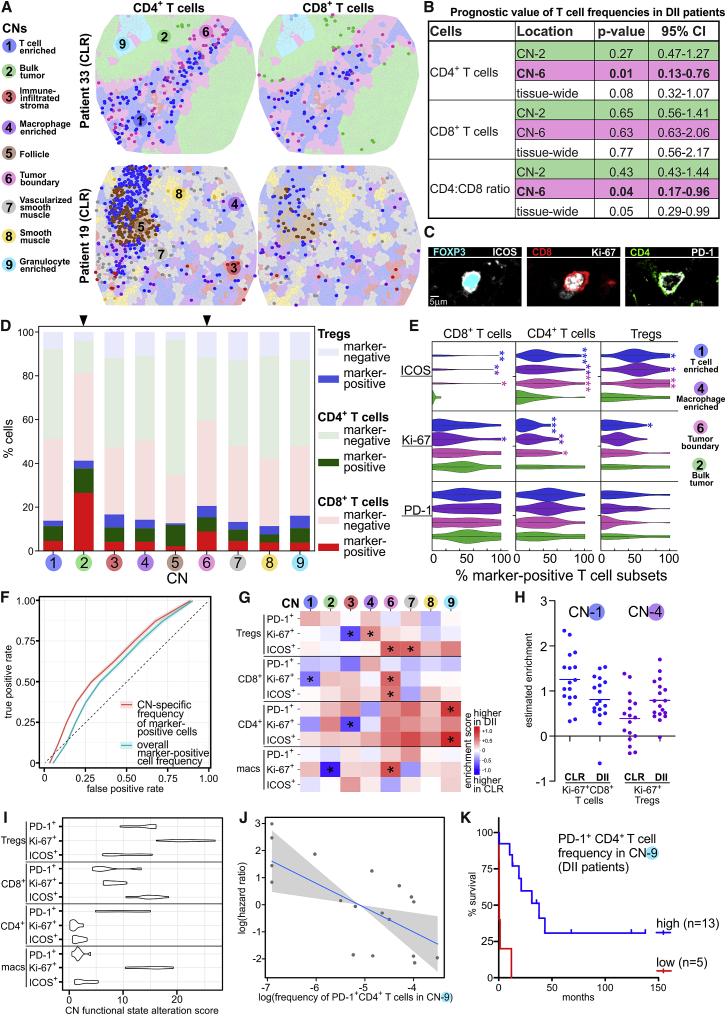
CN Functional States Are Indicators of Antitumoral Immunity (A) Example Voronoi diagrams of TMA spots, colored by CN, with CD4^+^ (left) and CD8^+^ T cells (right) overlaid in each CN as points of the corresponding CN color. (B) Table of Cox proportional hazards regression results for T cell frequencies in the indicated CNs. Each CN-specific frequency was tested individually in a distinct model (DII patients: n = 18, 13 deaths). (C) Example staining for ICOS, Ki-67, and PD-1 on different T cell subsets. (D) Relative proportions of CD4^+^ (FOXP3^−^) T cells, CD8^+^ T cells, and Treg cells positive for at least one of the functional markers (ICOS, Ki-67, and PD-1) in each CN. Pooled data from all patients are shown (cell numbers: CN-1, 17,822; CN-2, 735; CN-3, 4,031; CN-4, 11,753; CN-5, 4,695; CN-6, 2,681; CN-7, 4,504; CN-8, 1,368; CN-9, 2,884). (E) Violin plots of CN-specific CT frequencies of marker-positive CD4^+^ T cells, CD8^+^ T cells, and Treg cells in CN-1, CN-2, CN-4, and CN-6. Asterisks indicate significant differences in the CN-specific CT frequency below compared with the frequency in CN-2 (bulk tumor), tested across patients (^∗^p < 0.05, ^∗∗^p < 0.01, ^∗∗∗^p < 0.001, Student’s t test). (F) Receiver operating characteristic curves comparing the performance of L1-regularized logistic regression classifiers (CN-specific CT frequency versus overall frequency of marker-positive cells) over repeated holdout samples to classify patients by group. (G) Heatmap of estimated differential enrichment coefficients (^∗^p < 0.05, not adjusted for multiple tests). A positive coefficient (red) indicates that the corresponding CT is more enriched in DII patients in the given CN. (H) Estimated enrichment of Ki-67^+^ CD8^+^ T cells in CN-1 and Ki-67^+^ Treg cells in CN-4 for each patient. Horizontal lines represent the means across patients. (I) Estimated CN functional state alteration score for each CT. Variation corresponds to the distribution of the score across 10 resampling iterations. (J) Partial residual plot of the log frequency of PD-1^+^CD4^+^ T cells in CN-9 versus the estimated log hazard ratio with respect to overall survival in DII patients (p = 0.006; n = 18, 13 deaths; Cox proportional hazards regression). A pseudocount of 0.001 was added to the frequency for all patients when logarithms were computed. (K) Kaplan-Meier curves for overall survival in DII patients corresponding to the best splitting of DII patients into two groups along the CN-9-specific frequency of PD-1^+^CD4^+^ T cells. See also Figure S5, Figure S6A, and S6B, and STAR Methods.

The CD4^+^:CD8^+^ T cell ratio is often used as a simple measure to determine the overall balance of T cell function in cancer, providing prognostic information when measured in the tumor and tumor-immune interface (Shah et al., 2011; Wang et al., 2017a). The “tumor” and the “tumor-immune interface” are likely composed of finer sub-structures that were not addressed in previous studies. We therefore computed and visualized the frequencies of CD4^+^ and CD8^+^ T cells in each CN for each patient (Figure 6A).

We next assessed whether these frequencies or their ratios within the tumor CNs (CN-2 and CN-6) were associated with overall survival. We performed these tests only for DII patients because there was insufficient mortality (only four deaths) in the CLR patient group. The CD4^+^ T cell frequency and the CD4^+^:CD8^+^ T cell ratio in CN-6 were significant prognostic factors (Figure 6B). This suggested that T cell activity specifically in CN-6 (tumor boundary) could be important in the antitumoral immune response.

**Figure S5:**
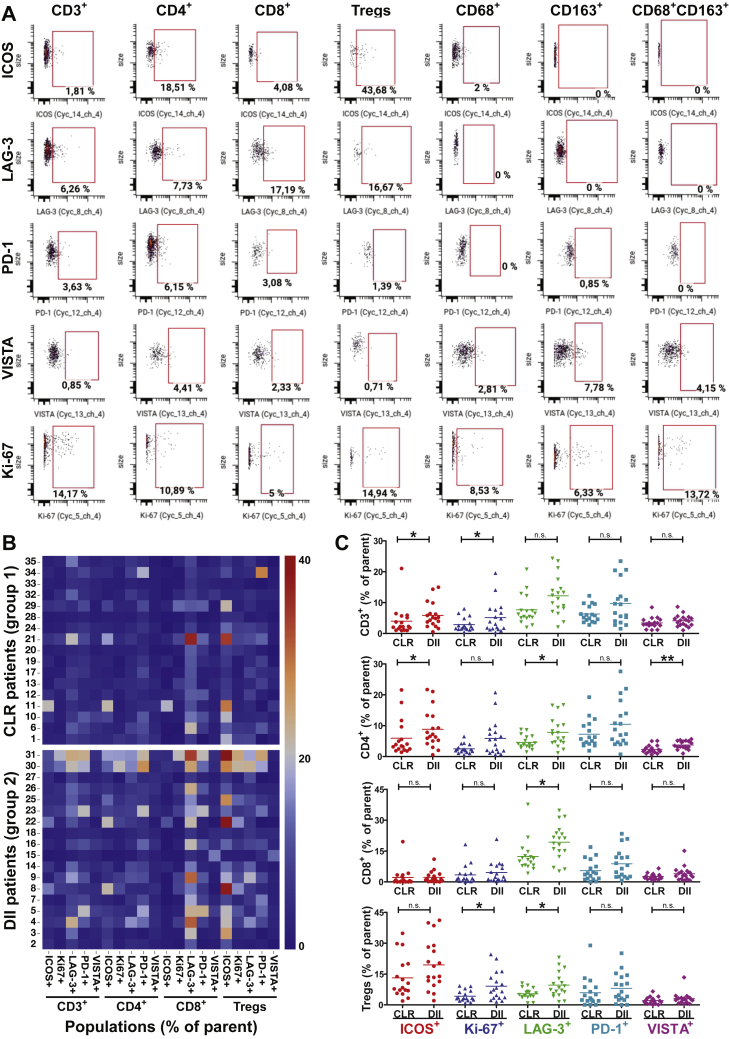
Gating Strategy for Analysis of Checkpoint Molecule Expression on T Cells and Macrophage Populations, Related to Figure 6 (A) Representative dot plots from CellEngine are shown for each marker and population. (B) Heatmap of marker-positive cell populations per patient. (C) Frequencies of marker-positive cell populations per patient. Data are mean values from four biological replicates (TMA cores) per patient (^∗^p < 0.05, ^∗∗^p < 0.01, Student’s t test).

In addition to quantifying frequencies and ratios of T cell subsets, CODEX visualization allowed simultaneous investigation of cellular functional states based on measured levels of key activation, co-stimulatory, and checkpoint molecules. We manually gated the functional markers PD-1, Ki-67 (a proliferation marker), and inducible costimulator (ICOS) on the T cell subsets (Figures 6C and S5). We then quantified the relative proportions of marker-positive (PD-1^+^, Ki-67^+^, ICOS^+^) T cell subsets across the nine CNs, pooling cells from all patients (Figure 6D). These functional markers were not included during identification of CTs or CNs.

In CN-2 (bulk tumor), the proportion of T cells expressing at least one of these three markers was approximately twice as high as in any other CN (Figure 6D). The extent of this enrichment intratumorally, even in comparison with the tumor boundary (CN-6), was striking. In addition to there being changes in the frequency of “functional” CD4^+^ and CD8^+^ T cells and Treg cells (as indicated by marker positivity) between CN-2 and other CNs, we would also expect there to be differences in the specific markers expressed by these marker-positive cells. For each of these subsets, and within each patient, we computed the relative proportion of ICOS^+^, Ki-67^+^, and PD-1^+^ cells among marker-positive cells in CN-1, CN-2, CN-4, and CN-6. We observed that the proportion of marker-positive cells that were ICOS^+^ was significantly higher in CN-1,CN-4, and CN-6 compared with CN-2 (Figure 6E). This indicates that T cell subsets expressing the activation marker ICOS are less frequent in the bulk tumor as a proportion of marker-positive cells. In contrast, we found that the frequency of Ki-67^+^CD4^+^ T cells was highest in the bulk tumor (Figure 6E, center panel). No significant differences were observed for the PD-1^+^ subsets (Figure 6E, bottom row). These data point toward a change in the inflammatory milieu within the bulk tumor compared with the tumor boundary and other iTME CNs and raise the question of which signals maintain this discrepant relationship.

### CN Functional States with Respect to T Cells Are Distinct between CLR and DII Patients and Are Correlated with Survival

If the biological processes related to antitumoral immunity co-occurring in each CN are altered between patient groups, we would expect to observe concurrent changes in the frequencies of the relevant functional CTs therein. We first built two statistical models to test whether we can classify patients as CLR versus DII using frequencies of T cells and macrophages expressing the functional markers PD-1, Ki-67, and ICOS. In the first model, we used only the overall frequency of each CT across the non-follicular CNs. In the second model, we used the frequencies of CTs in each of the non-follicular CNs (we refer to these in the following as “CN-specific CT frequencies”). Evaluating these models across repeated holdouts, we observed that including spatial information improved classification accuracy (Figure 6F). Thus, the frequencies of CTs within certain compartments could contain additional information distinguishing patient groups beyond what is contained in their overall frequencies.

This begged the question of whether CN-specific CT frequencies are more different between patient groups than expected by the differences in the overall frequencies of the corresponding CTs (i.e., when was a CT “differentially enriched” in a given CN between the patient groups?). This would be expected when there were differences in the functional state of a given CN and not just changes in overall cellular composition of the iTME. Therefore, for each CN-specific CT frequency, we estimated a linear model including as covariates patient group and overall frequency of the corresponding CT and visualized the estimated effects of the patient group as a heatmap. According to this model, a significant coefficient indicates that the given CT is more enriched in a given CN in one group; i.e., the CN-specific CT frequency is higher in one group than what can be explained by changes in the overall frequency of that CT (Figure 6G; STAR Methods).

All CNs except CN-8 (smooth muscle) exhibited significant differential enrichment of at least one functional cell subset between patient groups. In CLR patients, there was increased enrichment of Ki-67^+^CD8^+^ T cells in CN-1, Ki-67^+^ macrophages in CN-2, and Ki-67^+^ Treg cells and Ki-67^+^CD4^+^ T cells in CN-3 (Figure 6G, first 3 columns). In DII patients, there was increased enrichment of Ki-67^+^ Treg cells in CN-4; ICOS^+^ Treg cells, ICOS^+^ and Ki-67^+^ CD8^+^ T cells, and Ki-67^+^ macrophages in CN-6; ICOS^+^ Treg cells in CN-7; and PD-1^+^ and ICOS^+^ CD4^+^ T cells in CN-9 (Figure 6G, columns 4–8).

That there was differential enrichment of four CTs in CN-6 compared with only one in CN-2 suggested that changes in immune activity in the tumor boundary CN could be implicated in the impaired survival of DII patients (Figure 6G). In the tensor decomposition, we observed increased coupling between CN-1 (T cell enriched) and CN-4 (macrophage enriched) in DII patients. In addition, Ki-67^+^CD8^+^ T cells were less enriched in CN-1 and Ki-67^+^ Treg cells were more enriched in CN-4 in DII compared with CLR patients (Figure 6H), and activated ICOS^+^ Treg cells were enriched in CN-6 in DII patients (Figure 6G). These data suggest that, in DII patients, immunosuppressive activity is increased in CN-4 and CN-6 and that, in CN-6, this could oppose the cytotoxic activity from Ki-67^+^ and ICOS^+^ CD8^+^ T cells. In contrast, in CLR patients, there could be increased, unopposed cytotoxic activity in CN-1.

Different CTs could have different roles in iTME function in each patient group. We therefore computed a “CN functional state alteration score” for each marker-positive CTs. We compared the improvement in classification accuracy for a linear model trained to classify patients by group when the CN-specific frequencies for that CT in the non-follicular CNs were included in addition to its overall frequency across repeated holdouts (STAR Methods). We found that Ki-67^+^ Treg cells were the most CN functional state altering CT, followed by Ki-67^+^ macrophages (Figure 6I). Within CD4^+^ T cells, the PD-1^+^ subset had the strongest score for functional state alteration, suggesting a possible role of this T cell subset in driving the differences in antitumoral immune response between patient groups.

**Figure S6:**
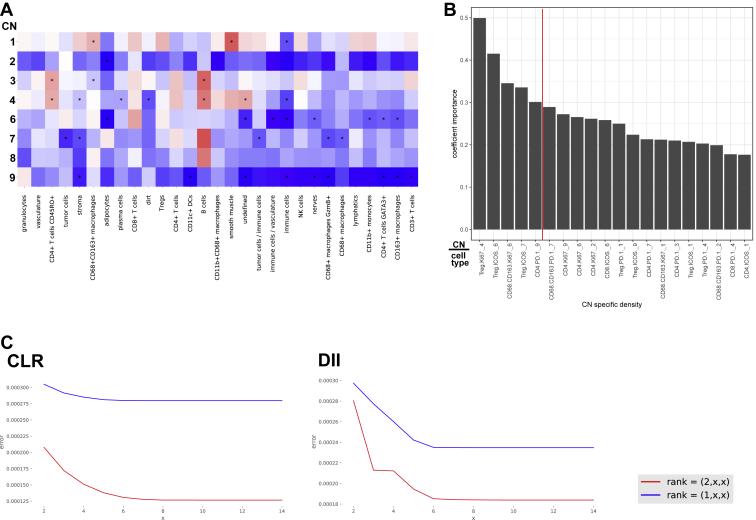
Heatmap of Estimated Differential Enrichment Coefficients, Feature Importance for the Classification Model, and Elbow Points for Tucker Tensor Decomposition, Related to Figure 6 and STAR Methods (A) Heatmap of estimated differential enrichment coefficients for cell types not shown in Figure 6G. Asterisks indicate CNs and cell types with a regression p value < 0.05 (not adjusted for multiple tests). A positive coefficient (red) indicates that the corresponding cell type is more enriched in DII patients than in CLR patients in the given CN. (B) Feature importance for classification model. Bar plot of absolute coefficient z-scores for CN-specific cell type frequencies, estimated from a model classifying patient groups using iterative resampling, as described in Figure 6F. The five CN-specific cell type frequencies with a coefficient importance of 0.3 or higher (left to red dotted line) were considered for assessment with respect to survival in DII patients. (C) Elbow points for Tucker tensor decomposition. Tensor decomposition loss for choices of rank in patient space, CN space, and cell-type space used for selection of decomposition rank in each patient group. Blue lines, one tissue module; red lines, two tissue modules. The elbow point was found at 6 CN modules and cell type modules (red line).

The granulocyte-enriched CN-9 stood out in the tensor decomposition analysis as being uniquely present in a tissue module distinct from the tumor in both patient groups. We also observed that PD-1^+^ and ICOS^+^ CD4^+^ T cells were more enriched in CN-9 in DII than in CLR patients (Figure 6G). In addition, CD11c^+^ DCs were present in CN-9 and were differentially enriched between patient groups (Figures 4B and S6A). These results suggest that certain processes occurring in CN-9, such as antigen presentation, could play key roles in the antitumoral immune response. Could the functional state of CN-9 contribute to the antitumoral response?

Survival differences between CLR and DII patients are associated with the presence of TLS (Figure 1). However, prognosticators of survival have not been established previously in DII patients. We assessed whether the frequencies of PD-1^+^ and ICOS^+^ CD4^+^ T cells in CN-9 were prognosticators of survival in DII patients. Notably, the PD-1^+^CD4^+^ T cell frequency in CN-9 was a significant prognostic factor for overall survival (p = 0.006, Cox proportional hazards likelihood ratio test, 18 patients, 13 deaths) (Figures 6J and 6K). Neither the overall frequency of PD-1^+^CD4^+^ T cells in the patient tissue nor the overall relative presence of CN-9 alone was a significant prognosticator of outcome (data not shown). The positive association of the frequency of PD-1^+^CD4^+^ T cells in CN-9 with overall survival is visible in Figure 6J, and this count can be used to stratify patients, as shown in Figure 6K. These findings could imply that specific events occurring in CN-9, related to production, maintenance, or function of PD-1^+^CD4^+^ T cells, could contribute to the antitumoral immune response in CRC. Furthermore, although we could only compute the association with survival in DII patients, CN-9 is present in both groups, indicating its relevance to all CRC patients. The only other features tested for association with survival (excluding those in Figure 6B) were the frequencies of Ki-67^+^ Treg cells in CN-4, ICOS^+^ Treg cells in CN-6 and CN-7, as well as Ki-67^+^CD68^+^CD163^+^ macrophages in CN-6 because these, in addition to PD-1^+^CD4^+^ T cells in CN-9, were the five most predictive features in the classification model in Figure 6F (Figure S6B).

Taken together, these data indicate that the functional states of CNs are different between CLR and DII patients and that CN functional states are potentially functionally relevant for the antitumoral immune response. Specifically, the functional state of a granulocyte-enriched CN, indicated by its frequency of PD-1^+^CD4^+^ T cells, was associated with overall survival in DII patients. These results imply that some aspect of the T cell subset activity harbored within the granulocyte-enriched CN could contribute to positive immune activity.

### Correlated CN Functional States Suggest Immunosuppressive Inter-CN Communication and an Altered Communication Network in DII Patients

The tensor decomposition suggested that different CNs can recruit similar combinations of CTs, which could be interpreted as a form of communication between these CNs. We therefore expected that other forms of communication between CNs could give rise to functional state correlations. These correlations could be mediated by biological processes such as immune cell infiltration, antigen presentation, cytokine production, or processes still to be determined.

**Figure 7:**
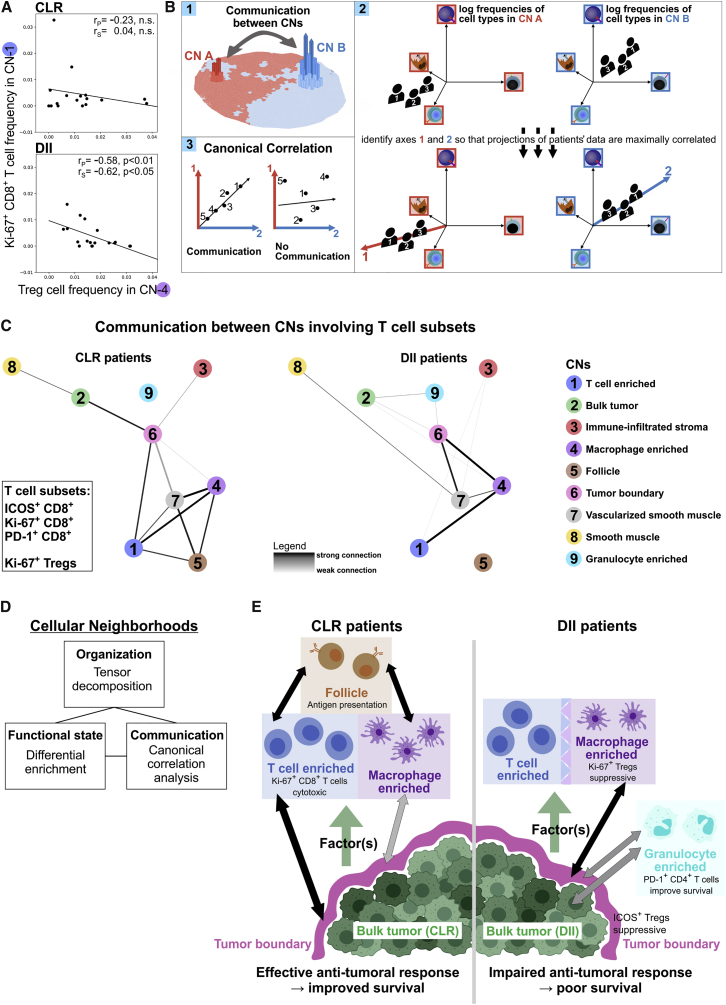
Altered Inter-CN Communication Favors Immunosuppression in DII Patients (A) Correlation of the frequency of Ki-67^+^CD8^+^ T cells in CN-1 (T cell enriched) and the frequency of Tregs in CN-4 (macrophage enriched) in each patient group. Spearman rank and Pearson correlation coefficients and p values are shown. (B) Schematic of the canonical correlation analysis (CCA). (C) The canonical correlation with respect to the frequencies of ICOS^+^, Ki-67^+^, and PD-1^+^ CD8^+^ T cells as well as Ki-67^+^ Treg cells in each pair of CNs was compared with a permuted null distribution within each patient group. Pairs of CNs whose observed canonical correlation with respect to these CTs was higher than 90% of permutations were connected by edges and visualized as a graph. (D) Conceptual framework for describing CRC iTME spatial behavior. (E) Model of differences in the iTME between CLR and DII patients with respect to CN organization (Figure 5), cellular function (Figure 6), and inter-CN communication (Figures 7A–7C).

Ki-67^+^ Treg cells were more enriched in CN-4 in DII patients, and Ki-67^+^CD8^+^ T cells were more enriched in CN-1 in CLR patients (Figures 6G and 6H). Given that Treg cells are capable of suppressing CD8^+^ T cell activity (Chen et al., 2005), we determined whether the frequency of Treg cells in CN-4 was correlated with the frequency of proliferating (Ki-67^+^) CD8^+^ T cells in CN-1 in each patient group separately. A significant negative correlation was observed only in DII patients (Figure 7A), suggesting that a suppressive program involving Treg cells and CD8^+^ T cells across CN-1 and CN-4 was only active in this patient group. Moreover, this finding suggested that there were changes in inter-CN communication between patient groups.

Should there not also be differences in communication with respect to multiple cell subsets and across other pairs of CNs? We explicitly mapped the communication between different CNs involving T cells by performing canonical correlation analysis (CCA; STAR Methods; Hardoon et al., 2004). CCA has been used to identify common signals across multiple data types (for example, in multi-omics analyses; Witten and Tibshirani, 2009), and so we used it to find common signals across multiple CNs with respect to their CN-specific CT frequencies. Briefly, for any given pair of CNs, the frequencies of the CTs of interest are computed within those CNs (Figure 7B.1). CCA then identifies the two combinations of CN-specific CT frequency variations, one from each CN, so that their correlation is maximized (Figure 7B.2). This maximal correlation is the canonical correlation between two CNs, which we used as a proxy for functionally relevant communication (Figure 7B.3). We applied CCA to the frequencies of PD-1^+^, Ki-67^+^ and ICOS^+^ CD8^+^ T cells as well as Ki-67^+^ Treg cells in each pair of CNs. In both patient groups, we identified pairs of CNs that likely communicate by comparing the observed canonical correlations with a permuted null distribution. These CN communication relationships were visualized as a graph of nodes corresponding to CNs and edges indicating possible communication for each patient group (Figure 7C).

There were interesting differences between patient groups in the communication networks of CNs with respect to functional T cell subsets. In CLR and DII patients, CN-6 (tumor boundary) played a central role. In CLR patients, CN-6 was strongly connected to CN-1 (T cell enriched) and CN-2 (bulk tumor) and weakly connected to CN-4 (macrophage enriched) (Figure 7C, left graph). CN-4 and CN-1 were not directly connected to CN-2, suggesting that functional T cell subsets in CN-1 and CN-4 could be communicating with the bulk tumor via the tumor boundary. In contrast, in DII patients, CN-6 was not connected to CN-1 but strongly connected to CN-4 and weakly connected to CN-2 (Figure 7C, right graph). This is compatible with the communication between CN-1 and CN-6 having been rerouted via CN-4 in DII patients, which, as we showed, had immunosuppressive communication with CN-1 and increased enrichment of Ki67^+^ Treg cells (Figure 6G). The fact that the edge between CN-6 (tumor boundary) and CN-2 (bulk tumor) was weaker in DII patients suggests that communication with respect to T cells between the tumor and tumor boundary could be disrupted. In addition, only in DII patients were there connections between CN-9 (granulocyte enriched) and the tumor CNs. Because the frequency of PD-1^+^CD4^+^ T cells in CN-9 was associated with survival in DII patients (Figures 6J and 6K), these data suggest a possible role of CN-9 in T cell-mediated antitumoral responses. Finally, in CLR patients, CN-5 (follicle) was connected to CN-1, CN-4, and CN-7 (vascularized smooth muscle), indicating that the processes occurring in the follicle influence T cell activity in multiple CNs.

In summary, in DII patients, correlation analysis allowed us to observe a possible immunosuppressive program at play between CN-1 (T cell enriched) and CN-4 (macrophage enriched). Moreover, the network of communication between CNs with respect to functional T cell subsets was altered between patient groups in a manner that suggested that this immunosuppressive program could be affecting the phenotypes of functional T cell subsets in the tumor boundary and bulk tumor. Thus, the increased spatial contact between CN-1 and CN-4 as well as the changes in organization of CNs and CTs observed in the tensor decomposition could play a role in the impaired outcomes of these patients.

## Discussion

The iTME is a dynamic system in which the combination of immune CT, location, and functional orientation leads to a tumor-rejecting or tumor-promoting environment (Chen and Mellman, 2017; Fridman et al., 2017). Naturally, the question arises of how a tumor avoids immune action, such as by T cell exclusion (Joyce and Fearon, 2015) or increased prevalence of certain CTs, including Treg cells, macrophages, and myeloid-derived suppressor cells (Gajewski et al., 2013; Hanahan and Weinberg, 2011; Kumar et al., 2016; Munn and Bronte, 2016). Several recent studies have characterized tumor-immune phenotypes in detail, but how spatial organization and the associated crosstalk between iTME components determine the effectiveness of the antitumoral immune responses has not yet been investigated extensively (Ali et al., 2020; Jackson et al., 2020; Kather et al., 2017, 2018; Newell and Becht, 2018).

Here we developed technology and an analytical framework to systematically describe the components of complex tissue types and their coordinated behaviors. Using CODEX data from 140 FFPE tissue regions, we identified CNs of the CRC iTME and probed their organization, functional states, and communication (Figure 7D). Our framework enabled development of a rich model of how the spatial behavior of the iTME could be different between patient groups (Figure 7E).

First, the tensor decomposition provided a structured interpretation of the differences in the underlying biology organizing the iTME in DII patients compared with CLR patients. Specifically, in DII patients, we observed coupling of T cell and macrophage CNs. These CNs were also more interdigitated in DII patients. This could potentially be attributed to recruitment factors provided by the tumor utilized by CTs occupying both of these CNs. Furthermore, the tissue modules suggest that, in CLR patients, the tumor could be interfering with the main immune processes to a lesser extent than in DII patients (Figure 7E).

Second, the CN-specific CT frequencies indicated differences in the functional states of CNs with respect to T cells. Specifically, in DII patients, CN-4 (macrophage enriched) had increased enrichment of Ki-67^+^ Treg cells, whereas CN-1 (T cell enriched) had decreased enrichment of Ki-67^+^CD8^+^ T cells. Notably, the functional state of CN-9 (granulocyte enriched) with respect to PD-1^+^CD4^+^ T cells was associated with improved survival outcomes in DII patients.

Finally, we identified possible changes in the communication involving T cells across CNs. Specifically, in DII patients, CN-4 appeared to suppress immune activation in CN-1. Furthermore, CCA suggested that, in CLR patients, there was direct communication between CN-1 and CN-6 (tumor boundary), which could be attributed to T cell exchange between these CNs. In contrast, in DII patients, the more suppressive CN-4 was connected to the tumor boundary, suggesting that suppression in CN-4 might have a role in the antitumoral immune response.

In summary, we provide a conceptual framework for interpreting spatial biology in a dynamic tissue such as the tumor microenvironment. Applying these tools to large, well-annotated patient cohorts could yield clinical biomarkers, therapeutic strategies, and insight into how spatial tissue behaviors facilitate antitumoral immunity.

### Limitations of Study

The experimental technology and computational framework presented here have certain limitations. First, imaging and downstream analysis are affected by tissue quality, and some tissue types may not be suitable for multiplexed fluorescence imaging because of autofluorescence. Poorly fixed tissues can lead to low signal-to-noise ratios and potentially misleading staining patterns. Development of an antibody panel requires extensive validation across positive and negative control tissues, which is a costly process. For instance, key immune markers such as major histocompatibility complex (MHC) class I and CD16 as well as additional tumor and stroma markers could not be included in our panel at this time, whereas some included markers were statistically uninformative. Iterative refinement of informative markers will enhance future studies. Segmentation and CT identification required extensive validation and manual merging of identified clusters. This may be overcome in future studies by computational correction for batch-dependent variation in staining intensity as well as general-purpose neural network-based segmentation and CT identification. The imaging artifacts in our data were identified by a unique CT cluster and CN and, therefore, were easy to remove. In other datasets, further image processing steps may be required to handle artifacts. Additionally, the neighborhood analysis was contingent on a collection of CNs that could be readily identified and verified in the original imaging data. In other tissue types, CNs may be less well defined, and alternate representations of the tissue and techniques for assessing cellular colocalization patterns (for example, network models or geospatial analyses) may be more appropriate. Conclusive biological interpretation of the differences between patient groups observed in the tensor decomposition and CCA will require a much larger sample size than used in this study.

## STAR★Methods

### Key Resources Table

**Table:** 

REAGENT or RESOURCE	SOURCE	IDENTIFIER
**Antibodies and Proteins**		

Purified antibodies, see Table S1B	various	various
Mouse IgG	Sigma	I5381
Rat IgG	Sigma	I4131
Biotinylated VG1 hyaluronan-detection reagent	Bollyky Lab, Stanford U	Clark et al., 2011

**Oligonucleotides**		

CODEX oligonucleotides, see Table S1C	TriLink Biotechnologies and Integrated DNA Technologies	N/A

**Biological Samples**		

FFPE tissue blocks	Institute of Pathology, University of Bern	N/A

**Chemicals and Reagents**		

Streptavidin-PE	Biolegend	405203
PBS	Thermo Fisher Scientific	14190-250
NaCl	Thermo Fisher Scientific	S271-10
Na_2_HPO_4_	Sigma	S7907
NaH_2_PO_4_ ∙ 7 H_2_O	Sigma	S9390
MgCl_2_ ∙ 6 H_2_O	Sigma	M2670
NaN_3_	Sigma	S8032
EDTA	Sigma	93302
TCEP	Sigma	C4706
NaOH	Sigma	S8263
BS3	Thermo Fisher Scientific	21580
DMSO	Thermo Fisher Scientific	D128-4
DMSO	Sigma	472301
DMSO ampoules	Sigma	D2650
Paraformaldehyde ampoules, 16%	Thermo Fisher Scientific	50-980-487
BSA	Sigma	A3059
Tris 1 M, pH 8.0	Teknova	T1080
Candor PBS antibody stabilizer solution	Thermo Fisher Scientific	NC0436689
Salmon sperm DNA, sheared	Thermo Fisher Scientific	AM9680
Triton™ X-100	Sigma	T8787
Ethanol, 100%	Sigma	E7023
Acetone, 100%	Thermo Fisher Scientific	A929-4
Methanol, 100%	Thermo Fisher Scientific	A412-4
Trizma® HCl	Sigma	T3253
Trizma® Base	Sigma	T1503
Drierite indicating desiccant	Thermo Fisher Scientific	07-578-3A
Bondic polyacrylamide gel	Amazon	B018IBEHQU
Dako target retrieval solution, pH 9.0	Agilent	S236784-2
TBS IHC wash buffer with Tween® 20	Cell Marque	935B-09
Antibody diluent	Agilent	S080981-2
Protein block, serum-free	Agilent	X090930-2
Dual endogenous enzyme-blocking reagent	Agilent	S200380-2
Liquid DAB+ substrate chromogen system	Agilent	K346711-2
EnVision+ HRP Mouse	Agilent	K400011-2
EnVision+ HRP Rabbit	Agilent	K400211-2
Hematoxylin, ready-to-use	Agilent	S330930-2
Eosin Y solution	Sigma	HT110116
Cytoseal XYL	Thermo Fisher Scientific	8312-4
Sally Hansen Nail Polish, clear	Amazon	B00CMFMYEG

**Critical Commercial Instruments, Consumables, Kits and Assays**		

LTS filter tips, 10 μl	Rainin	30389225
LTS filter tips, 200 μl	Rainin	30389239
LTS filter tips, 1000 μl	Rainin	30389212
Amicon™ Ultra Centrifugal Filters, 50kDa	Thermo Fisher Scientific	UFC505096
Nalgene™ Rapid Flow 500 ml filter, 0.2 μm	Thermo Fisher Scientific	09-740-28C
Glass coverslips, 22x22 mm, # 1 1/2	Electron Microscopy Sciences	72204-01
Frosted microscope slides	Thermo Fisher Scientific	12-550-343
Glass coverslip storage box	Qintay	CS-22
22x22 mm coverslip mounting gaskets	Qintay	TMG-22
Wheaton™ Coverslip glass jars	Thermo Fisher Scientific	02-912-637
Dumont #5/45 coverslip forceps	Fine Science Tools	11251-33
ST4020 small linear stainer	Leica	14050946425
Labconco Fast-Freeze Flasks, Complete Assembly, 900ml	Thermo Fisher Scientific	10-269-63
FreeZone® 4.5 Plus Cascade Benchtop Freeze Dry System	Labconco	7386030
Lab Vision™ PT module	Thermo Fisher Scientific	A80400012
8-strip tubes, 0.2 ml	E&K Scientific	280008
8-strip caps, flat top	E&K Scientific	491008
8-strip caps, dome top	E&K Scientific	491018
CODEX acrylic plates	Bayview Plastic Solutions	custom made
BZ-X710 fluorescence microscope	Keyence	N/A
Hoechst 33342	Thermo Fisher Scientific	62249
DRAQ5	Cell Signaling Technology	4084L
Vectabond™	Vector Labs	SP-1800
Vacuum Desiccators, 23L	Thermo Fisher Scientific	08-648-112
Corning™ black 96-well plates	Thermo Fisher Scientific	07-200-762
Axygen aluminum sealing film	VWR Scientific	47734-817
TMA Grand Master	3DHistech	N/A
Pannoramic P250 digital slide scanner	3DHistech	N/A
CODEX System	Akoya Biosciences	N/A

**Deposited Data**		

Single-cell data table	Mendeley	https://dx.doi.org/10.17632/mpjzbtfgfr.1
Flow formatted data	CellEngine	https://cellengine.com/#/experiments/5ea1170788ae4203c2959042
Processed primary imaging data	The Cancer Imaging Archive	https://doi.org/10.7937/tcia.2020.fqn0-0326

**Software and Algorithms**		

BZ-X viewer	Keyence	N/A
CODEX driver	Akoya Biosciences	N/A
CODEX Toolkit, version 1.3.5	https://github.com/nolanlab/CODEX	Goltsev et al., 2018
Microvolution software for deconvolution	https://www.microvolution.com/	N/A
ImageJ (Fiji version 2.0.0)	https://imagej.net/	N/A
VorteX (X-shift clustering algorithm)	https://github.com/nolanlab/VORTEX	Samusik et al., 2016
CellEngine	https://cellengine.com	Bjornson-Hooper et al., 2019
R, version 3.4.3	https://www.r-project.org	N/A
R studio desktop, version 1.1.423	https://www.rstudio.com/	N/A
Neighborhood analysis notebooks	This paper	https://github.com/nolanlab/NeighborhoodCoordination
Tensorly Python package	http://tensorly.org/	Kossaifi et al., 2019
Statsmodel Python package	https://www.statsmodels.org/	Seabold and Perktold, 2010
Scikit learn Python package	https://scikit-learn.org/	Pedregosa et al., 2011
Survival R package	https://cran.r-project.org/web/packages/survival/index.html	Therneau, 2015
Glmnet R package	https://cran.r-project.org/web/packages/glmnet/index.html	Friedman et al., 2010
Visreg R package	https://cran.r-project.org/web/packages/visreg/index.html	Breheny and Burchett, 2013
Deldir R package	https://cran.r-project.org/web/packages/deldir/index.html	N/A
ComplexHeatmap R package	https://bioconductor.org/packages/release/bioc/html/ComplexHeatmap.html	Gu et al., 2016
The Human Protein Atlas	http://www.proteinatlas.org	Uhlén et al., 2015
Pathology Outlines	http://www.pathologyoutlines.com	N/A

### Resource Availability

#### Lead Contact

Further information and requests for reagents and resources should be directed to and will be fulfilled by the lead contact, Garry P. Nolan (gnolan@stanford.edu).

#### Materials Availability

All materials used in this study are commercially available, as specified in the Key Resources Table and Table S1. Instructions on how to generate DNA oligonucleotide-conjugated antibodies are available in STAR Methods.

#### Data and Code Availability

Software code created for this study can be obtained at:

https://github.com/nolanlab/NeighborhoodCoordination

Single cell data table can be downloaded from Mendeley:

https://dx.doi.org/10.17632/mpjzbtfgfr.1

Flow formatted data can be obtained from CellEngine:

https://cellengine.com/#/experiments/5ea1170788ae4203c2959042

Processed primary imaging data can be obtained from The Cancer Imaging Archive:

https://doi.org/10.7937/tcia.2020.fqn0-0326

Raw primary imaging data can be obtained from the authors directly upon reasonable request.

### Experimental Model and Subject Details

A cohort of 715 patients who underwent surgery for primary colorectal cancer between 2003 and 2014 at the University Hospital Bern, Switzerland, was screened. Clinicopathological data for all patients were extracted from clinical and pathological reports. The peritumoral inflammatory reaction was retrospectively assessed in all patients by L.N., under the supervision of I.Z. and C.M.S., in a blinded fashion using digitally scanned H&E-stained tumor sections. The tumor invasive margin was assessed for the presence of CLR according to the Graham-Appelman (G-A) criteria (Graham and Appelman, 1990). Cases were categorized as either G-A 0 (lymphoid aggregates absent), G-A 1 (occasional lymphoid aggregates with rare or absent germinal centers) or G-A 2 (intense reaction with numerous lymphoid aggregates and germinal centers). The overall density of the peritumoral immune infiltrate was determined according to the Klintrup-Mäkinen (K-M) score (Klintrup et al., 2005), with low-grade (absent or mild and patchy inflammatory infiltrate; score 0-1) or high-grade (dense, linear inflammatory infiltrate with destruction of cancer cell islets; score 2-3) scorings. Cases with pre-operative therapy, pathological tumor, nodes, metastasis (pTNM) stage 0-2, absent peritumoral inflammatory infiltrate (K-M score 0), and those with insufficient material or information (total of 566 cases) were excluded. From the remaining 149 cases, 62 patients were identified based on their unique pattern of peritumoral inflammation and split into two groups: CLR group (G-A 2, any K-M grade) versus DII group (G-A 0, K-M high-grade). Subsequently, 35 cases (17 CLR [10 females, 7 males] versus 18 DII [8 females, 10 males]) were selected at random, matched for gender, age, and cancer type, location, and cancer stage (for patient details, see Figure 1D and Table S1). The use of patient tissue samples and data was approved by the local Ethics Committee of the Canton of Bern (KEK 200/2014) and by Stanford’s Institutional Review Board (HSR 48803).

### Method Details

#### Construction of tissue microarrays

FFPE tissue blocks were retrieved from the tissue archive at the Institute of Pathology, University of Bern, Switzerland. For the multi-tumor TMA, 70 unique different tissues were selected (54 different cancers and non-malignant tumors as well as 16 normal tissues; for details see Table S1E and Data S2A). Tumor and normal tissue regions were annotated on corresponding hematoxylin and eosin (H&E)-stained sections by a board-certified surgical pathologist (C.M.S.). A next-generation TMA (ngTMA®) with 0.6 mm diameter cores was assembled using a TMA Grand Master automated tissue microarrayer (3DHistech).

For the CRC study, two independent 70-core ngTMAs were created, containing four 0.6-mm cores per patient. TMA cores were digitally annotated by L.N., under the supervision of C.M.S and I.Z., as follows: CLR group, two regions containing a tertiary lymphoid structure and two diffuse immune infiltrate regions per patient; DII group, four diffuse immune infiltrate regions per patient. TMAs were sectioned at 3 μm thickness, stained with H&E, and digitized using a Pannoramic P250 digital slide scanner (3DHistech).

Square glass coverslips (Electron Microscopy Sciences) were pre-treated with Vectabond™ (Vector Labs) according to the manufacturer’s instructions. Briefly, coverslips were immersed in 100% acetone for 5 min and then incubated in a mixture of 2.5 mL Vectabond™ and 125 mL 100% acetone in a glass beaker for 30 min. Coverslips were washed in 100% acetone for 30 s and air-dried, baked at 70°C for 1 h, and stored at room temperature. The 4-μm thick sections of the TMAs were mounted on Vectabond™-treated coverslips and stored in a coverslip storage box (Qintay) at 4°C in a vacuum desiccator (Thermo Fisher) containing drierite desiccant (Thermo Fisher) until analysis.

#### Buffers and solutions

Buffers and solutions were prepared, filtered sterile using 500 mL 0.2-μm pore size filters and stored at room temperature unless otherwise specified. Staining solution 1 (S1): 5 mM EDTA (Sigma), 0.5% w/v bovine serum albumin (BSA, Sigma) and 0.02% w/v NaN_3_ (Sigma) in PBS (Thermo Fisher Scientific); storage at 4°C. Staining solution 2 (S2): 61 mM NaH_2_PO_4_ ∙ 7 H_2_O (Sigma), 39 mM NaH_2_PO_4_ (Sigma) and 250 mM NaCl (Sigma) in a 1:0.7 v/v solution of S1 and doubly-distilled H_2_O (ddH_2_O); final pH 6.8-7.0. Staining solution 4 (S4): 0.5 M NaCl in S1. TE buffer: 10 mM Tris pH 8.0 (Teknova), 1 mM EDTA and 0.02% w/v NaN_3_ in ddH_2_O. Tris stock solution (for conjugation buffer), 50 mM, pH 7.2 (at room temperature) was prepared in ddH_2_O using Trizma HCl and Trizma Base according to Sigma’s Trizma mixing table. Buffer C (for conjugation): 150mM NaCl, 2 mM Tris stock solution, pH 7.2, 1 mM EDTA and 0.02% w/v NaN_3_ in ddH_2_O. CODEX 2.0 buffer (H2): 150mM NaCl, 10 mM Tris pH 7.5 (Teknova), 10 mM MgCl_2_ ∙ 6 H_2_O (Sigma), 0.1% w/v Triton™ X-100 (Sigma) and 0.02% w/v NaN_3_ in ddH_2_O. Blocking reagent 1 (B1): 1 mg/ml mouse IgG (Sigma) in S2. Blocking reagent 2 (B2): 1 mg/ml rat IgG (Sigma) in S2. Blocking reagent 3 (B3): Sheared salmon sperm DNA, 10 mg/ml in H_2_O (Thermo Fisher). Blocking component 4 (BC4): Mixture of 57 non-modified CODEX oligonucleotides (see Table S1C) at a final concentration of 0.5 mM each in TE buffer. BS3 fixative solution (BS3): 200 mg/ml BS3 (Thermo Fisher) in DMSO from a freshly opened ampoule (Sigma); stored at −20°C in 15 μL aliquots. TCEP solution: 0.5 M TCEP (Sigma) in ddH_2_O, pH 7.0. Rendering buffer: 20% DMSO (v/v) in H2 buffer. Stripping buffer: 80% DMSO (v/v) in H2 buffer.

#### Generation of CODEX DNA-conjugated antibodies

All pipetting was performed using LTS filter tips (Rainin). Maleimide-modified short DNA oligonucleotides (for sequences, see Table S1C) were purchased from TriLink. Maleimide groups were deprotected by heating in toluene at 90°C for 4h (with exchange of toluene after 2h). Deprotected oligonucleotides were repeatedly washed in 100% ethanol, resuspended in buffer C, and aliquoted at 50 μg in 0.2 mL 8-strip tubes (E&K Scientific). Oligonucleotides were flash-frozen in liquid N_2_, lyophilized overnight in 900 mL Labconco Fast-Freeze Flasks (Thermo Fisher) using a FreeZone® 4.5 Plus lyophilizer (Labconco) and stored until conjugation at −20°C in an airtight box containing desiccant. Conjugations were performed at a 2:1 weight/weight ratio of oligonucleotide to antibody, with at least 100 μg of antibody per reaction. All centrifugation steps were at 12,000 g for 8 min, unless otherwise specified. Purified, carrier-free antibodies (for details on clones and manufacturers, see Table S1B) were concentrated on 50 kDa filters and sulfhydryl groups were activated using a mixture of 2.5 mM TCEP and 2.5 mM EDTA in PBS, pH 7.0, for 30 min at room temperature. After washing the antibody with buffer C, activated oligonucleotide was resuspended in buffer C containing NaCl at a final concentration of 400 mM. Oligonucleotide was then added to the antibody and incubated for 2 h at room temperature. The conjugated antibody was washed by resuspending and spinning down three times in PBS containing 900 mM NaCl. It was then eluted by centrifugation at 3,000 g for 2 min in PBS-based antibody stabilizer (Thermo Fisher) containing 0.5 M NaCl, 5 mM EDTA, and 0.02% w/v NaN_3_ (Sigma), and stored at 4°C.

#### CODEX FFPE tissue staining and fixation

Coverslips were handled using Dumont coverslip forceps (Fine Science Tools). For deparaffinization, coverslips were baked at 70°C for at least 1 h, followed by immersion in fresh xylene for 30 min. Sections were rehydrated in descending concentrations of ethanol (100% twice, 95% twice, 80%, 70%, ddH_2_O twice; each step for 3 min). Heat-induced epitope retrieval (HIER) was performed in a Lab Vision™ PT module (Thermo Fisher) using Dako target retrieval solution, pH 9 (Agilent) at 97°C for 10 min. After cooling to room temperature for 30 min, coverslips were washed for 10 min in 1x TBS IHC wash buffer with Tween® 20 (Cell Marque). Tissues were encircled using polyacrylamide gel (Bondic), and nonspecific binding was blocked for 1 h at room temperature using 100 μL of blocking buffer [S2 buffer containing B1 (1:20), B2 (1:20), B3 (1:20), and BC4 (1:15)]. For each coverslip, DNA-conjugated antibodies were added to 50 μL of blocking buffer on a 50-kDa filter unit, concentrated by spinning at 12,000 g for 8 min, and resuspended in blocking buffer to a final volume of 100 μl. This antibody cocktail was then added to the coverslip and staining was performed in a sealed humidity chamber overnight at 4°C on a shaker. After staining, coverslips were washed for 4 min in S2 and fixed in S4 containing 1.6% paraformaldehyde for 10 min, followed by three washes in PBS. Then, coverslips were incubated in 100% methanol on ice for 5 min, followed by three washes in PBS. Fresh BS3 fixative was prepared immediately before final fixation by thawing and diluting one 15 μL aliquot of BS3 in 1 mL PBS. Final fixation was performed at room temperature for 20 min, followed by three washes in PBS. Thereafter, coverslips were stored in S4 in a 6-well plate at 4°C for up to two weeks, or further processed for imaging.

#### Immunohistochemistry

Sections were cut to 4 μm thickness and placed on frosted histology glass slides (Thermo Fisher). H&E stained sections were obtained from each FFPE block. Deparaffinization, rehydration, and HIER were performed on an ST4020 small linear stainer (Leica) as described above. Nonspecific binding was blocked for 1 h at room temperature using 100 μL of serum-free protein block (Agilent). Antibodies were diluted in 100 μL antibody diluent (Agilent), and sections were stained overnight in a sealed humidity chamber at 4°C on a shaker. After staining, slides were washed for 10 min in 1x TBS IHC wash buffer with Tween® 20 (Cell Marque), and specimens were covered with dual endogenous enzyme-blocking reagent (Agilent) for 5-10 min at room temperature, followed by washing for 10 min. Bound antibodies were then visualized using the HRP/liquid DAB+ substrate chromogen system (Agilent) according to the manufacturer’s instructions. Sections were counterstained with hematoxylin, followed by dehydration, mounting, and imaging in brightfield mode on a BZ-X710 inverted fluorescence microscope (Keyence).

#### CODEX antibody screening, validation and titration

Antibodies were first screened and validated in CODEX single-stainings on tonsil tissue or a multi-tumor TMA, with cross-validation by manual IHC (Figures 2 and S1; Data S1; Table S1B). Briefly, after antibody staining and fixation, 100 nM of fluorescent DNA probe was added to the tissue in rendering buffer, containing 0.7 mg/ml sheared salmon sperm DNA, and was incubated at room temperature for 5-10 min, followed by washing with rendering buffer and S4 buffer. Coverslips were mounted onto glass slides with nail polish (Sally Hansen) or Cytoseal XYL (Thermo Fisher), dried in the dark and imaged on a BZ-X710 inverted fluorescence microscope. All validation was performed by or under the supervision of a board-certified surgical pathologist (C.M.S.) and confirmed with online databases (The Human Protein Atlas, Pathology Outlines) and the published literature.

#### CODEX multi-cycle reaction and image acquisition

Coverslips were removed from S4, and the tissue was covered with a small piece of cling film. The non-tissue containing parts of the coverslips were rinsed in ddH_2_O to remove salt residues and thoroughly dried using vacuum. Then, coverslips were mounted onto custom-made CODEX acrylic plates (Bayview Plastic Solutions; blueprints available upon request) using coverslip mounting gaskets (Qintay), creating a well in the acrylic plate above the tissue section for liquid storage and exchange. The cling film was removed, and the tissue was stained with Hoechst nuclear stain at a dilution of 1:1000 in H2 buffer for 1 min, followed by three washes with H2 buffer. The CODEX acrylic plate was mounted onto a custom-designed plate holder (blueprints available upon request) and secured onto the stage of a BZ-X710 inverted fluorescence microscope. Fluorescent oligonucleotides (concentration: 400 nM) were aliquoted in Corning™ black 96-well plates in 250 μL H2 buffer containing Hoechst nuclear stain (1:600) and 0.5 mg/ml sheared salmon sperm DNA, according to the multi-cycle reaction panels. For details on the order of fluorescent oligonucleotides and microscope light exposure times, see Table S1D. Black plates were sealed with aluminum sealing film (VWR Scientific) and kept at room temperature during the multi-cycle reaction. The final concentration of fluorescent oligonucleotides in the tissue-containing imaging well corresponded to 80 nM (1:5 dilution in rendering buffer).

Automated image acquisition and fluidics exchange were performed using an Akoya CODEX instrument and CODEX driver software (Akoya Biosciences). During imaging, the tissue was kept in H2 buffer. Hybridization of the fluorescent oligonucleotides was performed in rendering buffer. After imaging, fluorescent oligonucleotides were removed using stripping buffer. Overviews of the TMA were acquired manually using a CFI Plan Apo λ 2x/0.10 objective (Nikon), and automated imaging was performed using a CFI Plan Apo λ 20x/0.75 objective (Nikon). For multi-cycle imaging of the TMA spots, the multipoint function of the BZ-X viewer software (Keyence) was manually programmed to the center of each TMA spot and set to 17 Z stacks. Hoechst nuclear stain (1:3000 final concentration) was imaged in each cycle at an exposure time of 1/175 s. Biotinylated VG1 hyaluronan-detection reagent was produced as previously described (Clark et al., 2011), used at a dilution of 1:500, and visualized in the last imaging cycle using streptavidin-PE (1:2500 final concentration). DRAQ5 nuclear stain (1:500 final concentration) was added and visualized in the last imaging cycle. After each multi-cycle reaction, H&E-stainings were performed according to standard pathology procedures, and tissues were reimaged in brightfield mode.

A multi-cycle experiment performed the using multi-tumor TMA with 55 different antibodies, two nuclear markers and H&E took about 36 h to run and resulted in 3,630 tissue protein expression readouts (approximately 2,000 cells per 0.6-mm diameter core; total of 7.26 × 10^6^ single-cell protein readouts).

#### Figure creation

Parts of Figures 1B, 2A, and 3A were created using templates from Biorender (https://biorender.com). Parts of Figures 1, 4, 6, S4, and S5, and Data S5 and S6 were created and corresponding statistical analyses were performed using GraphPad Prism® 5.0 (GraphPad Software).

### Quantification and Statistical Analysis

#### Computational image processing

Raw TIFF image files were processed using the CODEX Toolkit uploader (Goltsev et al., 2018). Briefly, this software computationally concatenates and drift-compensates the images using Hoechst nuclear stain as a reference, removes out-of-focus light using the Microvolution deconvolution algorithm (Microvolution), subtracts the background (using blank imaging cycles without fluorescent oligonucleotides), and creates hyperstacks of all fluorescence channels and imaging cycles of the imaged TMA spots. The following settings in the CODEX uploader were used for the TMA experiments: Microscope: Keyence BZ-X710. Deconvolution: Microvolution. Objective type: Air. Magnification (x): 20. Numerical aperture: 0.75. Lateral resolution (nm/pixel): 377.442. Z pitch (nm): 1500. Number of Z-slices: 17. Color mode: grayscale. Drift compensation channel index: 1. Drift compensation reference cycle: 1. Best focus channel: 1. Best focus cycle: 1. Number of cycles / Range: 1-23 (multi-tumor TMA), 1-24 (CRC TMA). Tiling mode: snake. Region size X and Y: both 1. Tile overlap X and Y: both 0. H&E staining: yes (no in the case of background subtraction). Focusing fragment: no. Background subtraction: yes (no if H&E staining was co-processed). Use blind deconvolution: yes. Use bleach-minimizing cropping: no. Processing only, export as TIFF.

After uploading, all spots of each TMA were stitched together into a single 10x7 spots file using the grid/collection stitching plugin (Preibisch et al., 2009) in ImageJ software (Fiji, version 2.0.0). Antibody stainings were visually assessed for each channel and cycle in each spot, and seven-color overlay figures for selected markers were generated.

Hyperstacks from the CRC TMA spots were segmented based on DRAQ5 nuclear stain, pixel intensities were quantified, and spatial fluorescence compensation was performed using the CODEX toolkit segmenter, with the following settings: Radius: 7. Max. cutoff: 1.0. Min. cutoff: 0.07. Relative cutoff: 0.2. Cell size cutoff factor: 0.4. Nuclear stain channel: 4. Nuclear stain cycle: 23. Membrane stain channel: 1. Membrane stain cycle: −1 (i.e., not used). Use membrane: false. Inner ring size: 1.0. Delaunay graph: false. Anisotropic region growth: false. Comma-separated value (CSV) and flow cytometry standard (FCS) files were generated from each TMA spot and used for further downstream analysis.

#### Cleanup gating, unsupervised clustering and cluster validation

All 140 background-subtracted FCS files from both CRC TMAs were imported into CellEngine (https://cellengine.com). Gates were tailored for each file individually in a blinded manner by two experts in flow and mass cytometry (C.M.S and D.R.M.). Nucleated cells were positively identified, and artifacts were removed by gating on Hoechst1/DRAQ5 double-positive cells, followed by gating on focused cells in the Z plane (Data S5). After cleanup gating, FCS files were re-exported and subsequently imported into VorteX clustering software, where they were subjected to unsupervised X-shift clustering using an angular distance algorithm (Samusik et al., 2016). The following data import settings were applied: Numerical transformation: none. Noise threshold: no. Feature rescaling: none. Normalization: none. Merge all files into one dataset: yes. Clustering was based on all antibody markers except CD57, CD71, CD194 (CCR4), CDX2, Collagen IV, MMP9 and MMP12. The following settings were used for clustering: Distance measure: Angular distance. Clustering algorithm: X-shift (gradient assignment). Density estimate: N nearest neighbors (fast). Number of neighbors for density estimate (K): From 150 to 5, steps 30. Number of neighbors: determine automatically.

The optimal cluster number was determined using the elbow point validation tool and was at K = 40, resulting in 143 clusters. Clusters and corresponding data were exported as a CSV file and were manually verified and assigned to cell types by overlaying the single cells from each cluster onto the stitched TMA images in ImageJ, based on the unique cluster identifiers and cellular X/Y position, using custom-made ImageJ scripts (available upon request). Clusters with similar morphological appearance in the tissue and similar marker expression profiles were merged, and artifacts were removed, resulting in 28 final clusters. Minimal spanning trees of the clusters were generated in VorteX, based on angular distance, and were exported for each marker (Data S3).

#### Manual gating of cell types and checkpoint-positive cell subsets

After cleanup gating, the frequencies of major immune cell types (Data S5) and their expression of Ki-67 and checkpoint molecules (Figure S5) were manually gated in a blinded manner for each TMA spot in CellEngine, and statistics were exported for further analysis. For checkpoint-positive cell subsets, quantified checkpoint expression was compared to the raw image for each spot and gates were adjusted to best represent the raw image.

#### Generation of Voronoi diagrams and contact matrices

FCS files were exported from VorteX and subjected to a custom-made Java algorithm to create Voronoi diagrams and cell-to-cell contact matrices (code available upon request).

#### Details of statistical tests

In addition to the methods listed below, the specific details of statistical tests (values of n, p values, type of statistical test, definition of center etc.) are reported in the figure legends.

#### Computation of pairwise cell-cell contacts

Direct neighbors of each cell were determined by Delaunay triangulation as implemented in the *deldir* R package, using the default settings. From the original 28 cell clusters, two clusters were removed (undefined and tumor/immune cells), and the remaining clusters were merged into 14 clusters (Figure S4B). To represent associations of cells from various clusters, likelihood ratios or relative frequencies were calculated between the various clusters for each group of patients, according to the following formulas:

Likelihood ratios:

$$\frac{Nij*Nt}{Ni\text{∗}Nj}$$

Relative frequencies:

$$\frac{Nij}{Ni}$$

where

Nij is the number of edges between cells in cluster 1 and cluster 2

Nt is the total number of edges, $\sum_{i,j}Nij$

$$Ni\;is=\sum_{j}Nij$$

$$Nj\;is=\sum_{i}Nij$$

Log_2_ ratios of these metrics for CLR and DII patients were generated from the resulting matrices. A heatmap of the resulting values was plotted using the Heatmap function from the *ComplexHeatmap* R package after removing contacts between clusters where the number of unique adjacent cells was < 100 in both patient groups.

#### Neighborhood identification

For each of the 258,385 cells across these tissues across all spots and patient groups, a ‘window’ was captured consisting of the 10 nearest neighboring cells (including the center cell) as measured by Euclidean distance between X/Y coordinates. These windows were then clustered by their composition with respect to the 29 cell types that had previously been identified by X-shift clustering and supervised annotation and merging (Data S6). Of these, 28 cell clusters had been assigned to biological cell types and one to imaging artifacts, such as tissue folds and autofluorescent precipitations. This latter was included to identify poor quality regions of the image. Specifically, each window was converted to a vector of length 29 containing the frequency of each of the 29 cell types among the 10 neighbors, and the windows were subsequently clustered using Python’s *scikit-learn* implementation of MiniBatchKMeans with *k* = 10. Each cell was then allocated to the CN that its surrounding window was. To validate the CN assignment, these allocations were overlaid on the original tissue H&E-stained and fluorescent images. During this process, the CN cluster that contained the imaging artifacts (cellular cluster 29) was removed.

#### Non-negative Tucker tensor decomposition

The tensor of CN-cell type distributions for each patient, with dimensions patients x cell types x CNs, was produced by computing the frequency of each cell type in each CN in the non-follicular compartments (i.e., all CNs except CN-5). This tensor was split along the patient direction by patient group (CLR and DII). Non-negative Tucker decomposition as implemented in the *Tensorly* Python package was applied to each tensor (Kossaifi et al., 2019). The ranks in each dimension (2,6,6) were selected by a visual elbow point method assessing the decomposition loss (Figure S6C). Several random-starts were utilized to ensure stability.

The cell type modules correspond to the factors in cell-type space. The CN modules correspond to the factors in CN space. The interactions comprising a tissue module correspond to each 6x6 slice of the 2x6x6 core tensor.

#### Differential enrichment analyses

Linear models *Y*_*n,c*_ = β_0_ + β_1_*X* + β_3_*Y*_c_ + e were estimated, where *Y*_c_ is the log overall frequency of cell type c, *X* is an indicator variable for patient group, *Y*_*n,c*_ is the log frequency of cell type c in CN n, β_i_ are coefficients, and e is mean zero Gaussian noise. A pseudocount of 1e^-3^ was added prior to taking logs. These were estimated using the *statsmodels* Python package (Seabold and Perktold, 2010). The coefficient estimates and p values for β_1_ were extracted and visualized.

#### Classification of groups

Classification models were L1 regularized logistic regression models, fit using the *glmnet* R package (Friedman et al., 2010). Features were computed under the transformation x - > log(1e^-3^+ x) and z-normalized across the dataset prior to inclusion in any models.

Repeated hold-out (RHO) was utilized to estimate prediction error, which utilized 10 training samples from each patient group. The L1 regularization parameter was chosen for each sampled train-test split by cross-validation on the sampled training set, and a model using this regularization parameter was fit on the sampled training set. The model was evaluated on the sampled test set, and ROC curves were estimated on the test set (of size 15). This was repeated 1000 times. The feature importance was computed as the z-score of the absolute value of the coefficient across resampling, as reported in (Laurin et al., 2016).

#### CN functional state alteration score

The CN functional state alteration score was computed for each cell type individually. Specifically, for each cell type, the classification performance (AUC) was estimated using 200 RHO samples for two models classifying patients as CLR vs. DII. The first model included as features the overall frequency of that cell type. The second model included as features the overall frequency as well as the CN-specific cell type frequencies of that cell type in all CNs except CN-5. Each cell type had a different accuracy of classification with respect to these two models. The CN functional state alteration score was defined as the improvement in classification between the two models. Specifically, the negative log (base 10) of the p value of a one-sided t test for a greater mean in the AUC across RHO samples between the second and first model was used to quantitate them. Since this entire procedure contained randomness, it was repeated 10 times to estimate the variability across the dataset of the CN functional state alteration score.

#### Survival analysis

We tested the log (1e^-3^ + frequency in CN-9) for each of PD-1^+^CD4^+^ and ICOS^+^CD4^+^ T cells individually, in Cox proportional hazards regression models, estimated using the *survival* R package (Therneau, 2015). The p value displayed was from the Cox regression model, and the Kaplan-Meier curve displayed was computed using the optimal split of the samples along the PD-1^+^CD4^+^ T cell frequency in CN-9 variable. The partial residual plot in Figure 6J was created using the *visreg* R package (Breheny and Burchett, 2013). The proportional hazards assumption was supported by non-significant relationships between scaled Schoenfeld residuals and time as computed using the cox.zph function of the *survival* R package.

#### Canonical correlation analysis

For each CN, the log CN-specific cell type frequency of each of ICOS^+^, Ki-67^+^, and PD-1^+^ and CD8^+^ T cells as well as Ki-67^+^ Tregs was computed. For each pair of CNs, estimated canonical directions for the frequencies of these cells in each CN was estimated using the *scikit-learn* Python package (Pedregosa et al., 2011). For each pair of CNs, patients with no cells assigned to either CN were not included in the analysis. The correlation of the projections along these canonical directions was compared to a permutation distribution, corresponding to 5000 random permutations of the data. The permutation p value, i.e., the percentage of permutations whose estimated canonical correlation exceeded the observed one, was interpreted as the strength of communication.

## Supplemental Information

Document S1. Data S1–S7

Table S1. CRC Patient Characteristics, Antibodies, CODEX Oligonucleotides, CODEX Multi-Cycle Panels, Multi-tumor TMA, and Cell Types, Related to Figures 1, 2, 3, S2, and S3 and Data S1 and S2 (A) CRC patient characteristics. (B) Antibodies, clones, manufacturers and corresponding CODEX oligonucleotides. (C) Sequences of CODEX oligonucleotides. (D) CODEX multi-cycle panels. (E) Tissue composition of the multi-tumor TMA. (F) Cell types per patient.
