# Coordinated Cellular Neighborhoods Orchestrate Antitumoral Immunity at the Colorectal Cancer Invasive Front

**DOI:** 10.1016/j.cell.2020.10.021

**Published:** 2020-10-29

**Authors:** Christian M. Schürch, Salil S. Bhate, Graham L. Barlow, Darci J. Phillips, Luca Noti, Inti Zlobec, Pauline Chu, Sarah Black, Janos Demeter, David R. McIlwain, Shigemi Kinoshita, Nikolay Samusik, Yury Goltsev, Garry P. Nolan

(Cell *182*, 1341–1359.e1–e19; September 3, 2020)

During preparation of our article, Shigemi Kinoshita was inadvertently omitted as an author. The author list and Author Contributions section of the online article have been updated. All co-authors have approved the changes. We apologize for the omission.

