# Supplementary material for: Coordinated Cellular Neighborhoods Orchestrate Antitumoral Immunity at the Colorectal Cancer Invasive Front
Source: Cell. 2020 Sep 3;182(5):1341–1359.e19. doi: 10.1016/j.cell.2020.07.005 (PMC7479520; doi:10.1016/j.cell.2020.07.005)
Supplement: Document S1. Data S1–S7 [file mmc1.pdf]

Cell, Volume *182*

Supplemental Information

Coordinated Cellular Neighborhoods Orchestrate Antitumoral Immunity at the Colorectal Cancer Invasive Front

Christian M. Schürch, Salil S. Bhate, Graham L. Barlow, Darci J. Phillips, Luca Noti, Inti Zlobec, Pauline Chu, Sarah Black, Janos Demeter, David R. McIlwain, Nikolay Samusik, Yury Goltsev, and Garry P. Nolan

Data S1. Screening and Validation of CODEX Antibodies, Related to Figure 2, Table S1 and STAR Methods. Antibodies were conjugated to DNA oligonucleotides and tested individually along with cross-validation in standard IHC using the same, non-conjugated antibody clone. Clones, manufacturers, and staining specifications are listed for each antibody, and examples of IHC staining, CODEX staining (false gray color fluorescence images), and similar areas on independent H&E-stained sections are shown. Brightness and contrast adjusted. Scale bars, 100 μm.

Staining specifications	IHC	CODEX	H&E

Antigen: CD1a
Clones: O10 + C1A/711

Company:
Novus Biologicals (NBP2-34698)

Tissue: Skin

Dilution:
IHC: 1:600
CODEX: 1:100

CODEX oligo: 43-Alexa647

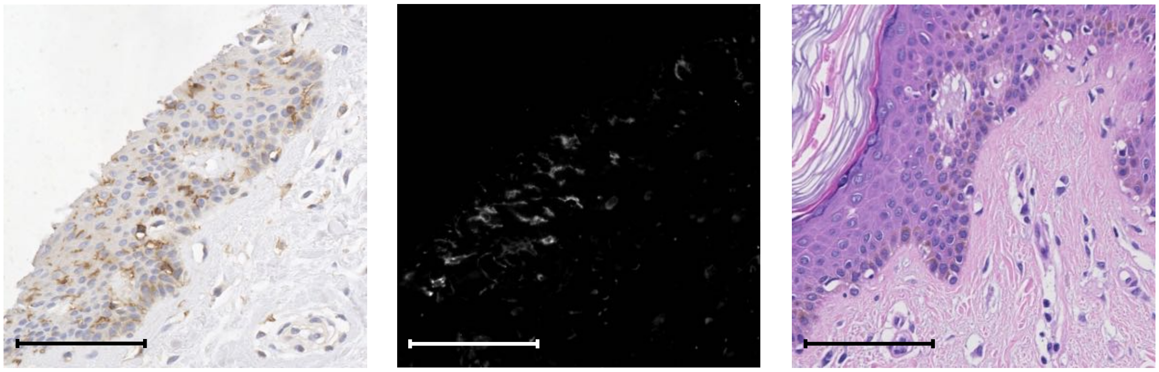

Antigen: CD2
Clone: RPA-2.10

Company:
BioLegend (300202)

Tissue: Skin T cell lymphoma

Dilution:
IHC: 1:5
CODEX: 1:25

CODEX oligo: 25-Alexa647

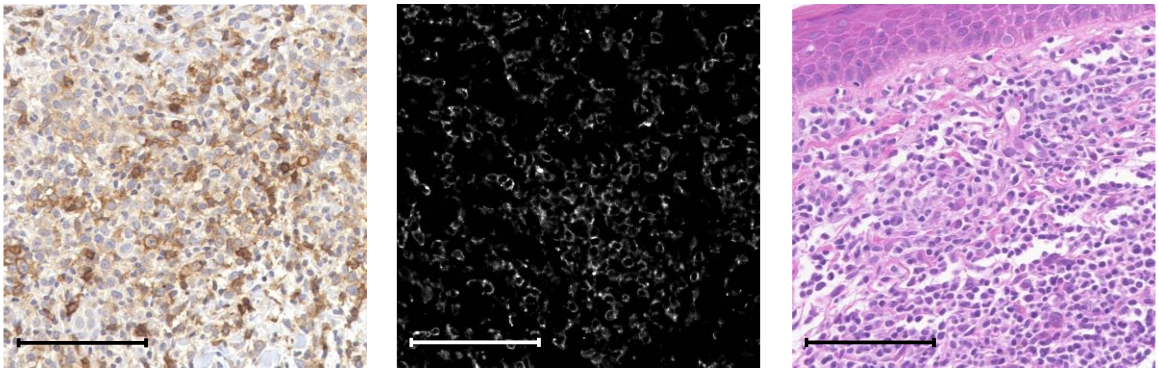

Antigen: CD3
Clone: D7A6E or MRQ-39

Companies:
Cell Signaling Technology (custom)
Cell Marque (custom)

Tissue: Tonsil

Dilution:
IHC: 1:100
CODEX: 1:100

CODEX oligo: 77-Alexa647

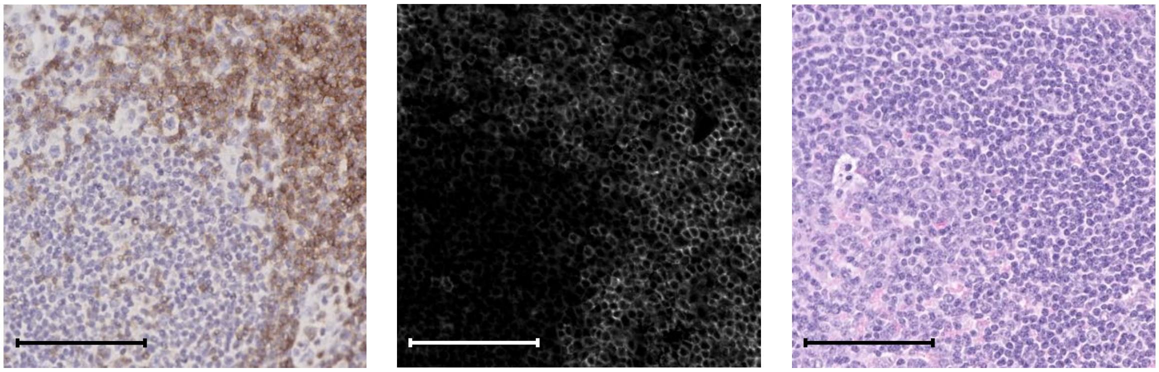

Antigen: CD4
Clone: EPR6855

Company:
Abcam (ab181724)

Tissue: Tonsil

Dilution:
IHC: 1:25
CODEX: 1:100

CODEX oligo: 20-ATTO550

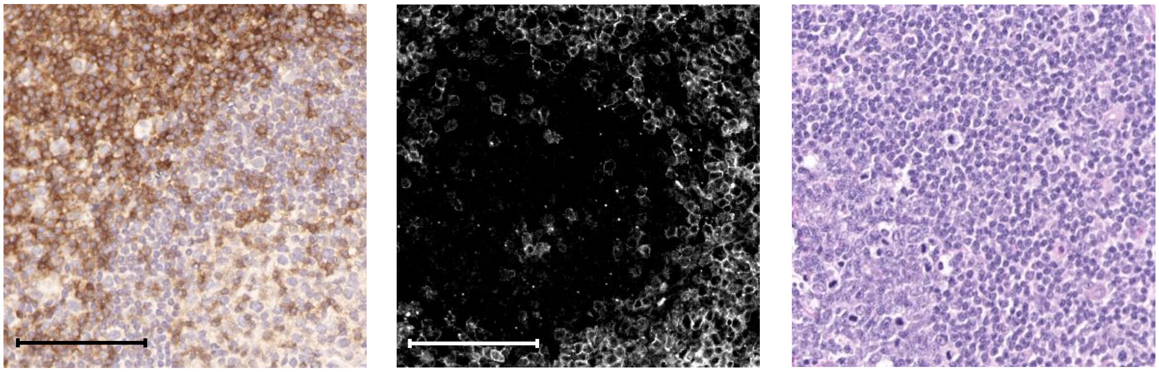

Antigen: CD5
Clone: UCHT2

Company:
BD Biosciences (555350)

Tissue: Tonsil

Dilution:
IHC: 1:5
CODEX: 1:25

CODEX oligo: 75-ATTO550

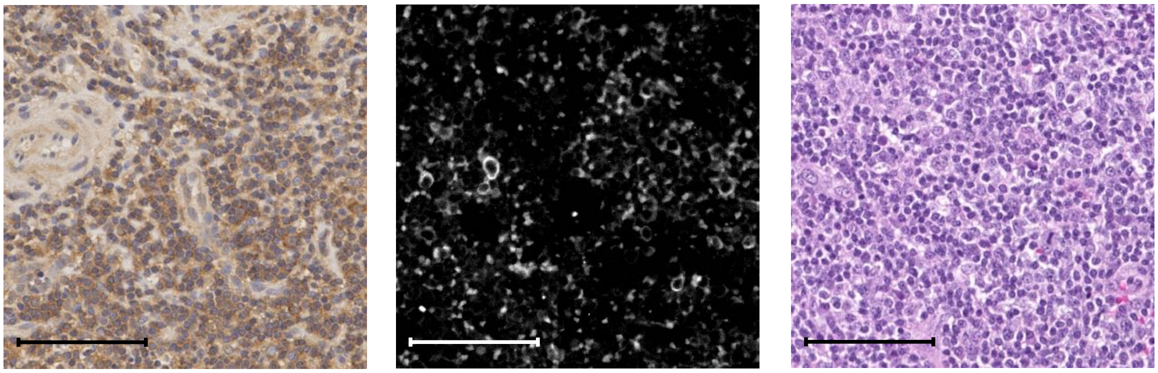

Staining specifications | IHC | CODEX | H&E

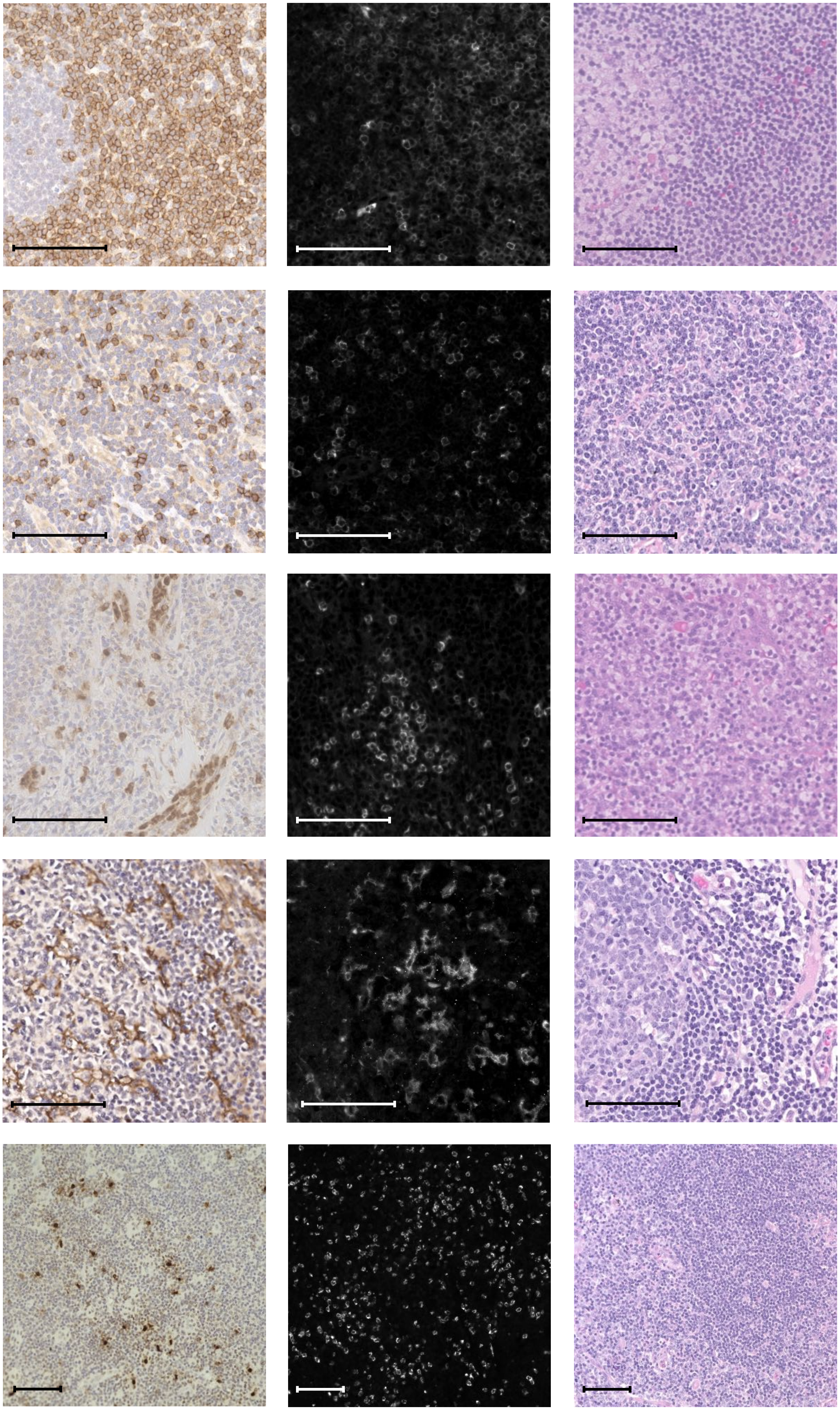

Antigen: CD7
Clone: MRQ-56

Company:
Cell Marque (custom)

Tissue: Tonsil

Dilution:
IHC: 1:50
CODEX: 1:100

CODEX oligo: 63-Alexa488

Antigen: CD8
Clone: C8/144B

Company:
Cell Marque (custom)
Santa Cruz Bio (sc-53212)

Tissue: Tonsil

Dilution:
IHC: 1:200
CODEX: 1:50

CODEX oligo: 8-Alexa488

Antigen: CD11b
Clone: EPR1344

Company:
Abcam (ab216445)

Tissue: Tonsil

Dilution:
IHC: 1:100
CODEX: 1:50

CODEX oligo: 28-Alexa647

Antigen: CD11c
Clone: EP1347Y

Company:
Abcam (ab216655)

Tissue: Tonsil

Dilution:
IHC: 1:100
CODEX: 1:50

CODEX oligo: 49-ATTO550

Antigen: CD15
Clone: MMA

Company:
BD Biosciences (559045)

Tissue: Tonsil

Dilution:
IHC: 1:100
CODEX: 1:200

CODEX oligo: 14-ATTO550

Staining specifications | **IHC** | **CODEX** | **H&E**

Antigen: CD15
Clone: HI98

Company:
Biolegend (301902)

Tissue: Tonsil

Dilution:
IHC: 1:100
CODEX: 1:200

CODEX oligo: 15-Alexa488

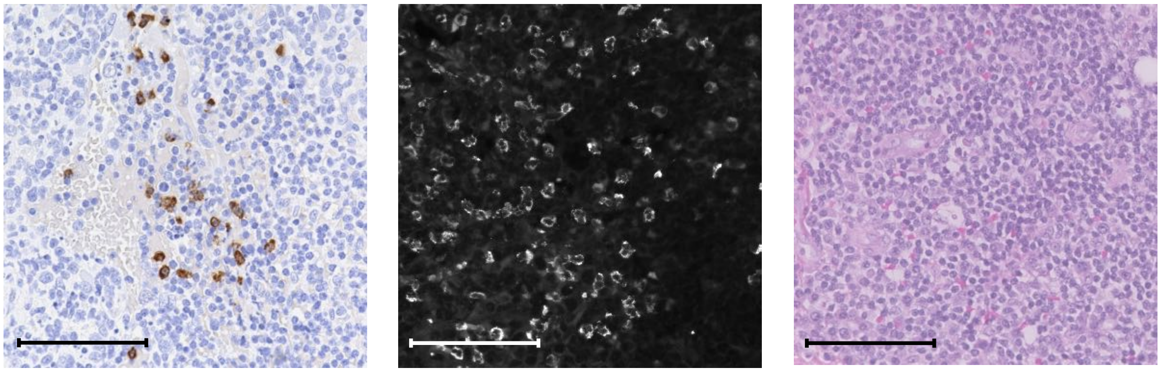

Antigen: CD16
Clone: D1N9L

Company:
Cell Signaling Technology (custom)

Tissue: Tonsil

Dilution:
IHC: 1:100
CODEX: 1:100

CODEX oligo: 26-Alexa647

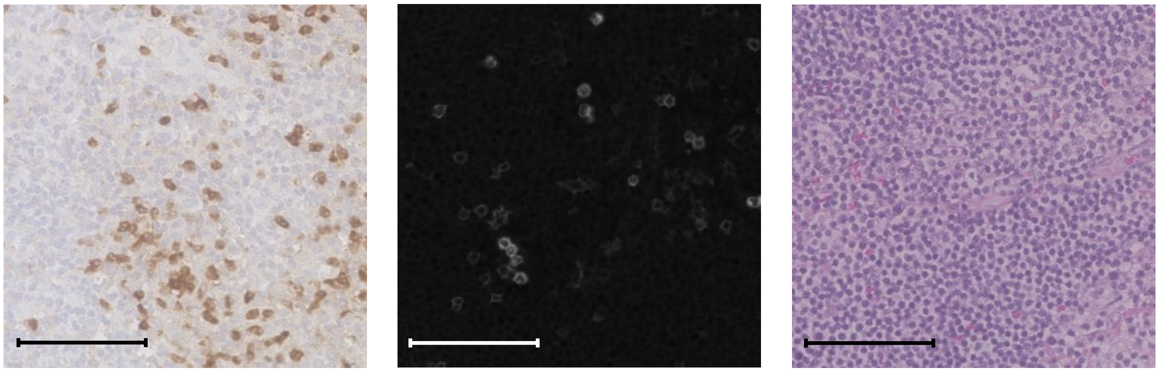

Antigen: CD20
Clone: rIGEL/773

Company:
Novus Biologicals (NBP2-54591)

Tissue: Tonsil

Dilution:
IHC: 1:100
CODEX: 1:200

CODEX oligo: 48-ATTO550

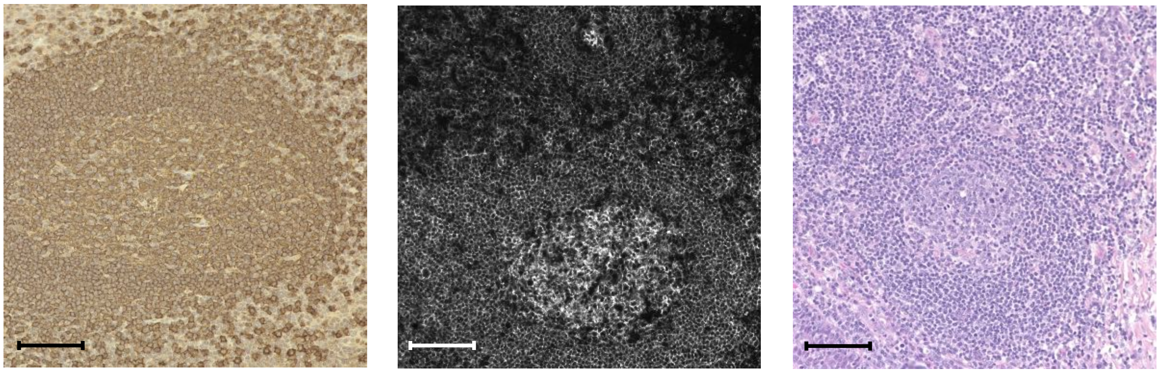

Antigen: CD20
Clone: H1

Company:
BD Biosciences (555677)

Tissue: Tonsil

Dilution:
IHC: 1:200
CODEX: 1:10

CODEX oligo: 48-ATTO550

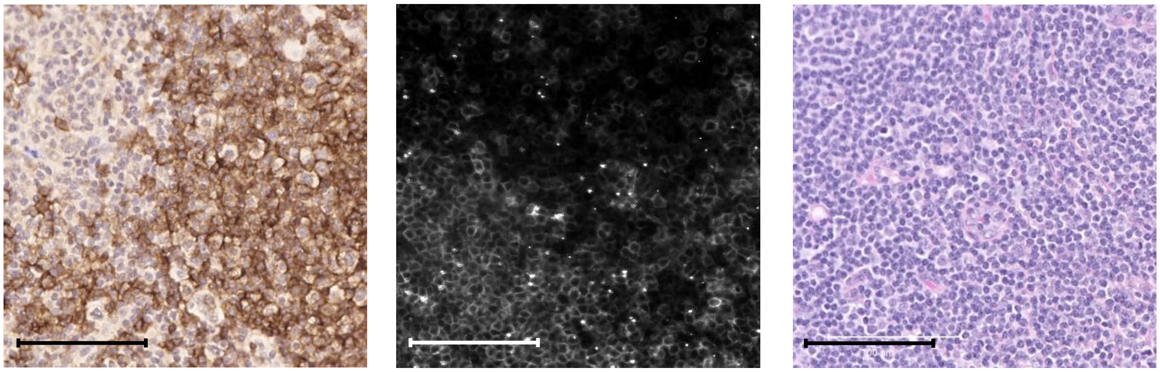

Antigen: CD21
Clone: Bu32

Company:
Biolegend (354902)

Tissue: Tonsil

Dilution:
IHC: 1:100
CODEX: 1:50

CODEX oligo: 21-Alexa647

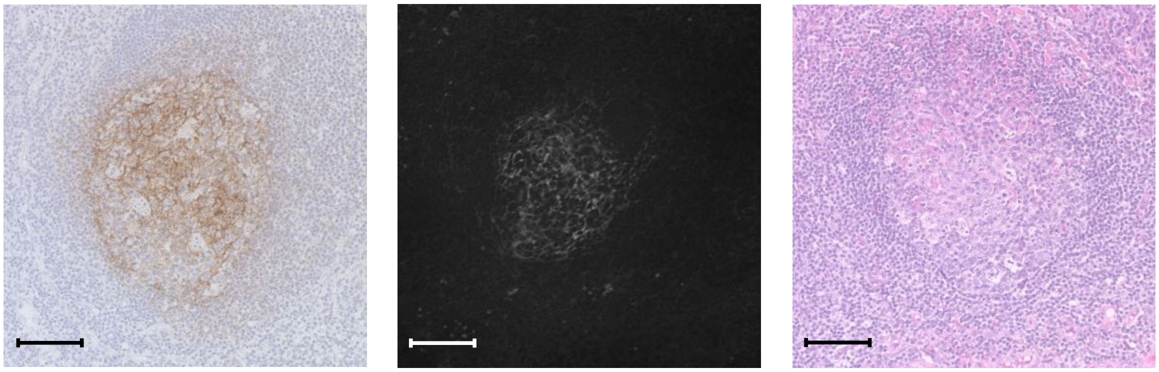

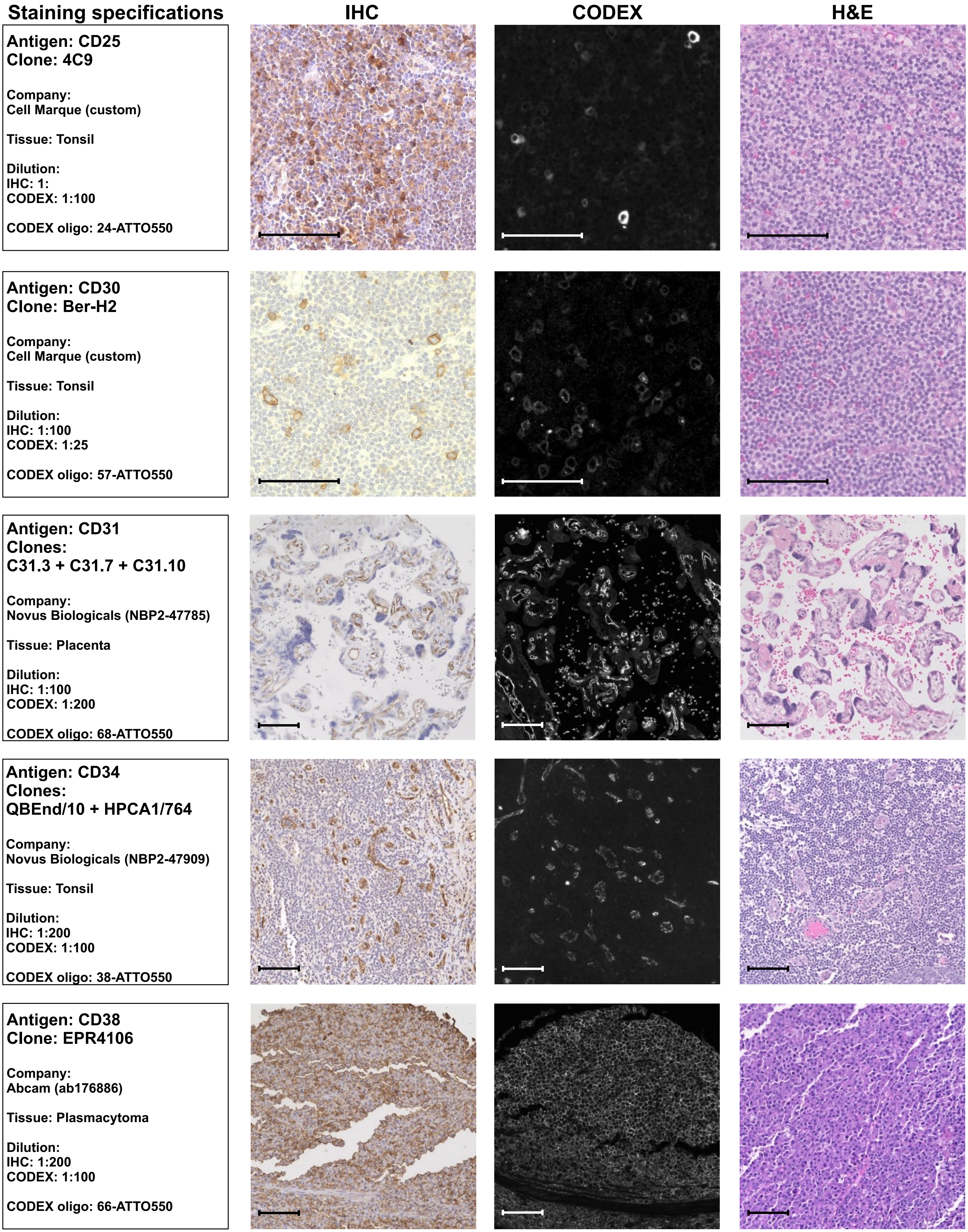

Staining specifications
IHC
CODEX
H&E
Antigen: CD25
Clone: 4C9
Company:
Cell Marque (custom)
Tissue: Tonsil
Dilution:
IHC: 1:
CODEX: 1:100
CODEX oligo: 24-ATTO550
Antigen: CD30
Clone: Ber-H2
Company:
Cell Marque (custom)
Tissue: Tonsil
Dilution:
IHC: 1:100
CODEX: 1:25
CODEX oligo: 57-ATTO550
Antigen: CD31
Clones:
C31.3 + C31.7 + C31.10
Company:
Novus Biologicals (NBP2-47785)
Tissue: Placenta
Dilution:
IHC: 1:100
CODEX: 1:200
CODEX oligo: 68-ATTO550
Antigen: CD34
Clones:
QBEnd/10 + HPCA1/764
Company:
Novus Biologicals (NBP2-47909)
Tissue: Tonsil
Dilution:
IHC: 1:200
CODEX: 1:100
CODEX oligo: 38-ATTO550
Antigen: CD38
Clone: EPR4106
Company:
Abcam (ab176886)
Tissue: Plasmacytoma
Dilution:
IHC: 1:200
CODEX: 1:100
CODEX oligo: 66-ATTO550

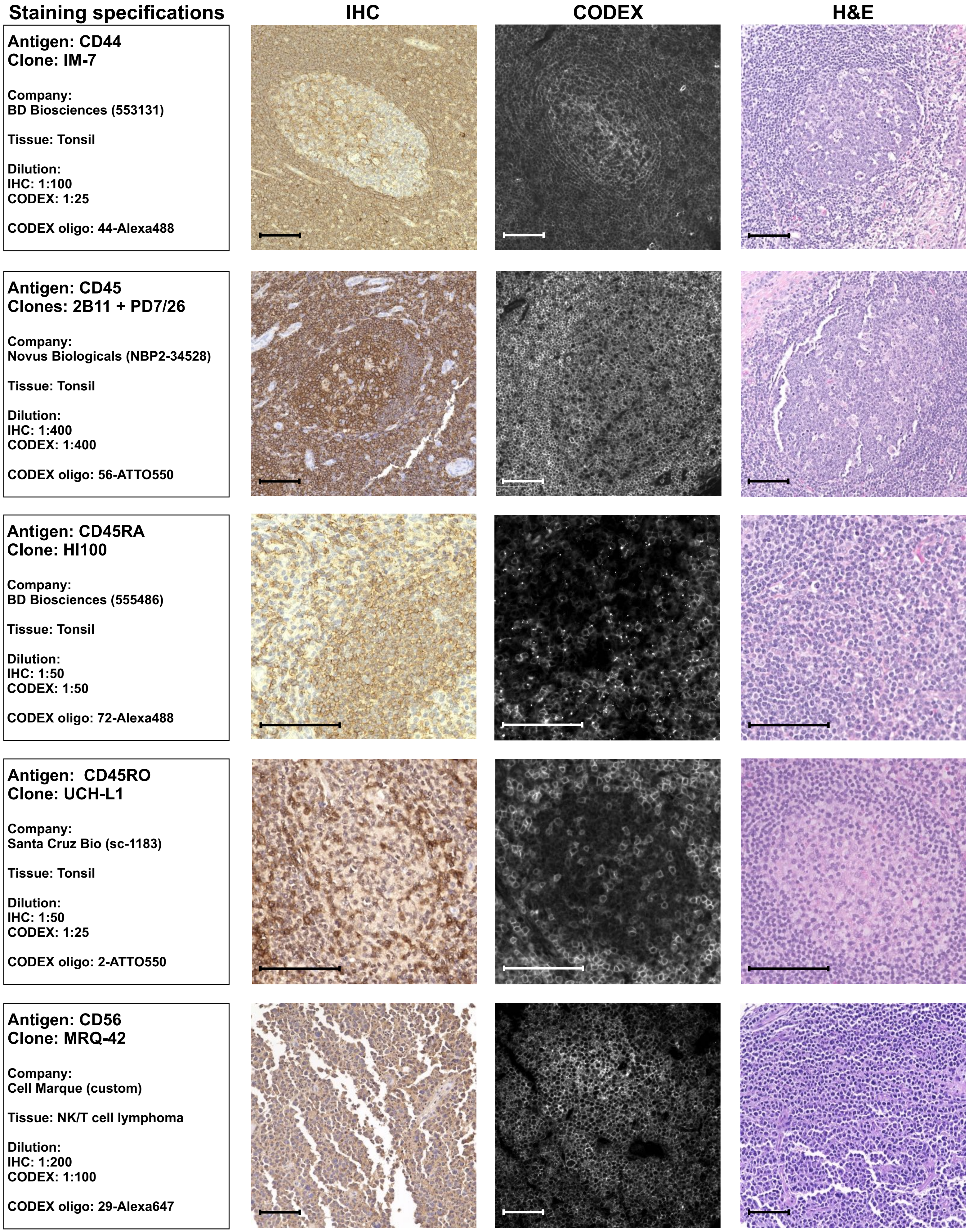
Staining specifications
IHC
CODEX
H&E
Antigen: CD44
Clone: IM-7
Company:
BD Biosciences (553131)
Tissue: Tonsil
Dilution:
IHC: 1:100
CODEX: 1:25
CODEX oligo: 44-Alexa488
Antigen: CD45
Clones: 2B11 + PD7/26
Company:
Novus Biologicals (NBP2-34528)
Tissue: Tonsil
Dilution:
IHC: 1:400
CODEX: 1:400
CODEX oligo: 56-ATTO550
Antigen: CD45RA
Clone: HI100
Company:
BD Biosciences (555486)
Tissue: Tonsil
Dilution:
IHC: 1:50
CODEX: 1:50
CODEX oligo: 72-Alexa488
Antigen: CD45RO
Clone: UCH-L1
Company:
Santa Cruz Bio (sc-1183)
Tissue: Tonsil
Dilution:
IHC: 1:50
CODEX: 1:25
CODEX oligo: 2-ATTO550
Antigen: CD56
Clone: MRQ-42
Company:
Cell Marque (custom)
Tissue: NK/T cell lymphoma
Dilution:
IHC: 1:200
CODEX: 1:100
CODEX oligo: 29-Alexa647

Staining specifications

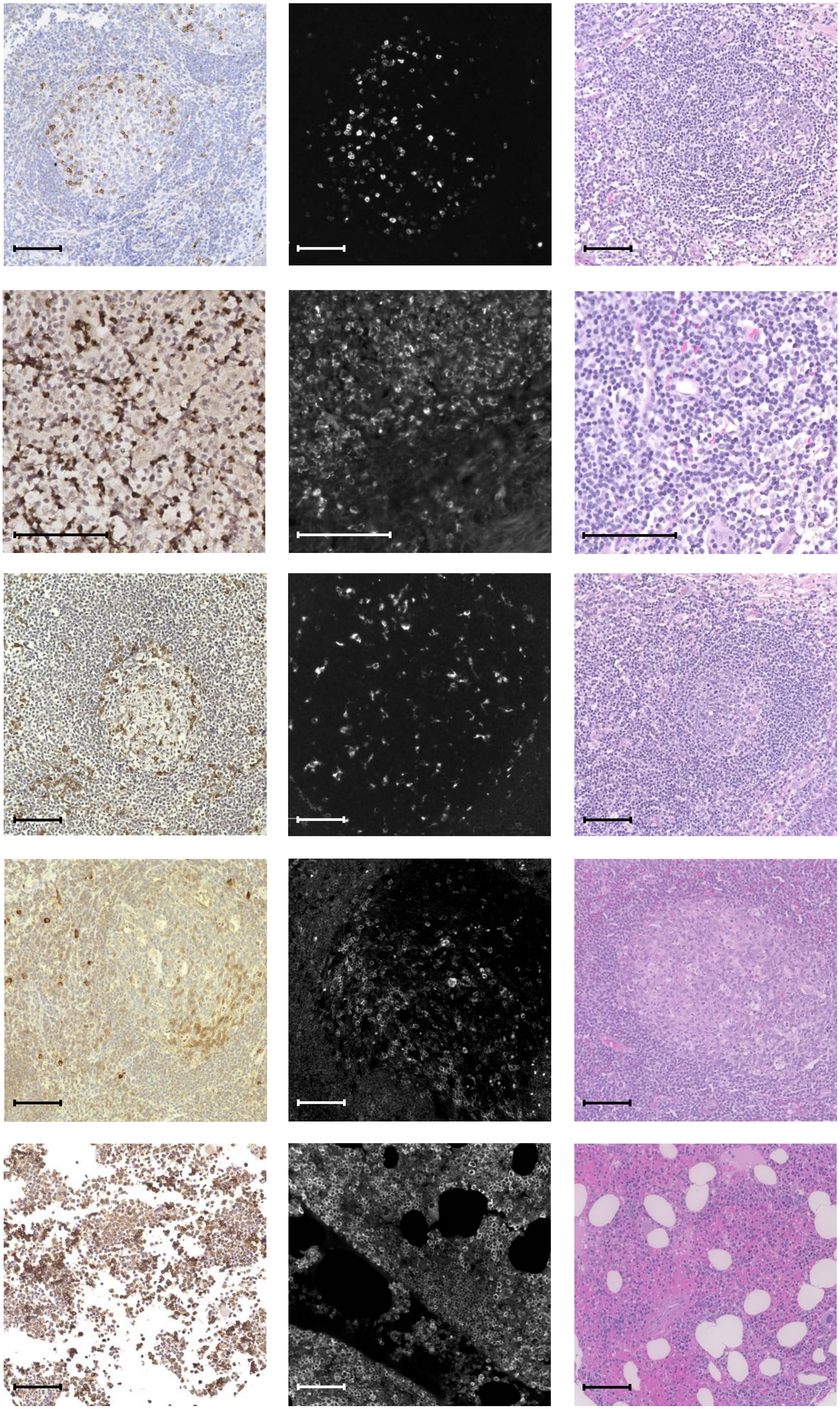

Antigen: CD57
Clone: HCD57

Company:
BioLegend (322325)

Tissue: Tonsil

Dilution:
IHC: 1:100
CODEX: 1:200

CODEX oligo: ST30-ATTO550

Antigen: CD66a
Clone: B1.1/CD66

Company:
BD Biosciences (551354)

Tissue: Tonsil

Dilution:
IHC: 1:200
CODEX: 1:200

CODEX oligo: 41-Alexa488

Antigen: CD68
Clone: D4B9C or KP-1

Companies:
Cell Signaling Technology (custom)
Biolegend (916104)

Tissue: Tonsil

Dilution:
IHC: 1:100
CODEX: 1:200

CODEX oligo: 70-Alexa647

Antigen: CD69
Clone: polyclonal

Company:
Novus Biologicals (AF2359)

Tissue: Tonsil

Dilution:
IHC: 1:200
CODEX: 1:200

CODEX oligo: 36-ATTO550

Antigen: CD71
Clone: MRQ-48

Company:
Cell Marque (custom)

Tissue: Myelolipoma

Dilution:
IHC: 1:400
CODEX: 1:100

CODEX oligo: 3-Alexa647

Staining specifications	IHC	CODEX	H&E

Antigen: CD79a
Clone: JBC117

Company:
Cell Marque (custom)

Tissue: Follicular lymphoma

Dilution:
IHC: 1:300
CODEX: 1:20

CODEX oligo: 46-Alexa488

Antigen: CD138 (Syndecan-1)
Clone: B-A38

Company:
Invitrogen (MA1-10091)

Tissue: Colorectal carcinoma

Dilution:
IHC: 1:100
CODEX: 1:50

CODEX oligo: ST76-Alexa647

Antigen: CD162 (CLA)
Clone: HECA-452

Company:
BD Biosciences (555946)

Tissue: Classic Hodgkin lymphoma

Dilution:
IHC: 1:600
CODEX: 1:200

CODEX oligo: 46-Alexa47

Antigen: CD163
Clone: EDHu-1

Company:
Novus Biologicals (NB110-48686)

Tissue: Tonsil

Dilution:
IHC: 1:200
CODEX: 1:200

CODEX oligo: 45-Alexa647

Antigen: CD164
Clone: N6B6

Company:
BD Biosciences (551296)

Tissue: Tonsil

Dilution:
IHC: 1:100
CODEX: 1:200

CODEX oligo: 69-Alexa488

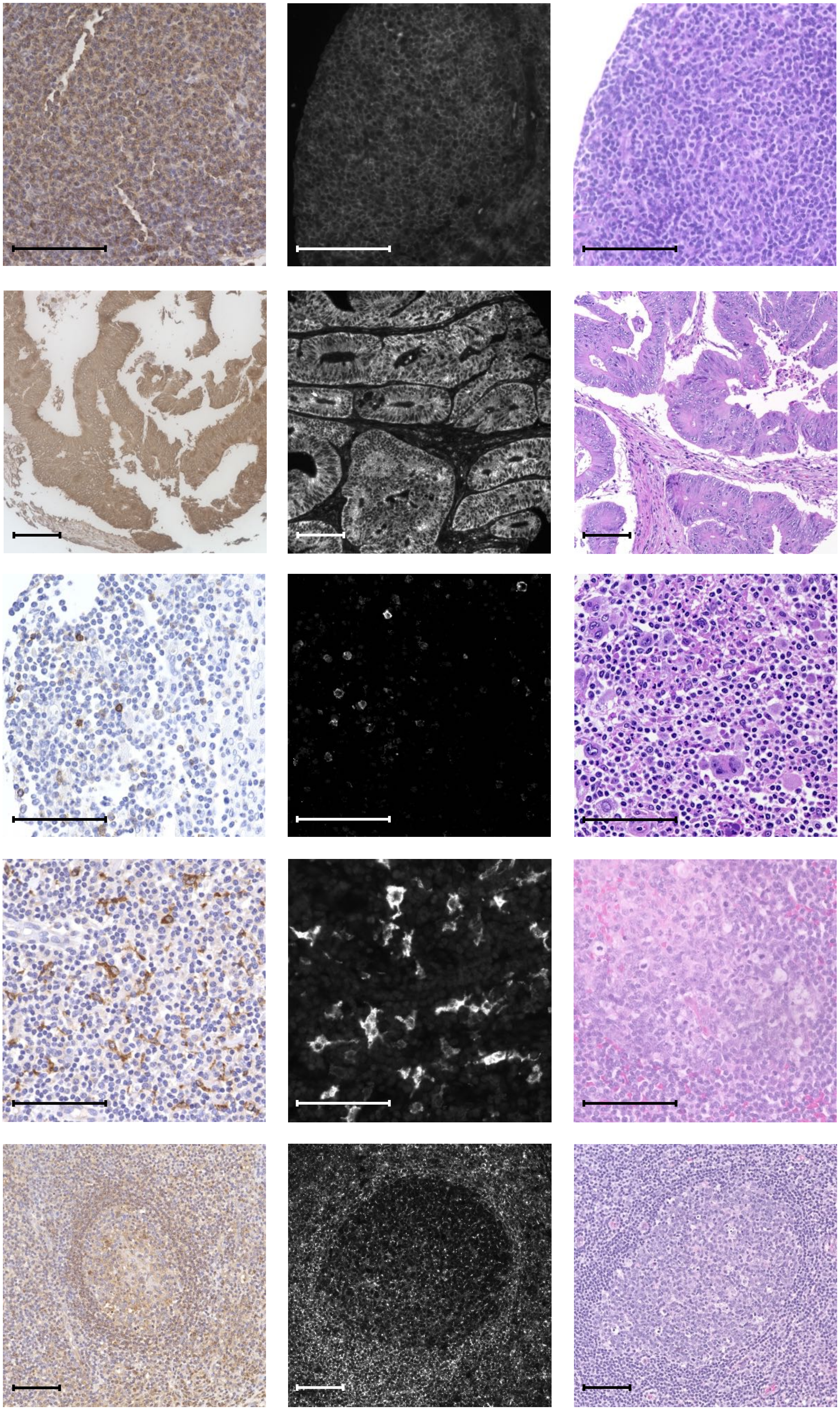

Staining specifications

Antigen: CD194 (CCR4)
Clone: L291H4

Company:
BioLegend (359402)

Tissue: Cholangiocarcinoma

Dilution:
IHC: 1:50
CODEX: 1:10

CODEX oligo: ST2-ATTO550

Antigen: CD223 (LAG-3)
Clone: D2G4O or 17B4

Companies:
Cell Signaling Technology (custom)
LSBio (LS-C18692-100)

Tissue: Tonsil

Dilution:
IHC: 1:300
CODEX: 1:20

CODEX oligo: 42-Alexa647

Antigen: CD235a
Clone: GA-R2

Company:
BD Biosciences (555569)

Tissue: Spleen

Dilution:
IHC: 1:400
CODEX: 1:200

CODEX oligo: 69-Alexa488

Antigen: CD274 (PD-L1)
Clone: E1L3N

Company:
Cell Signaling Technology (custom)

Tissue: Tonsil

Dilution:
IHC: 1:50
CODEX: 1:100

CODEX oligo: 11-ATTO550

Antigen: CD278 (ICOS)
Clone: D1K2T

Company:
Cell Signaling Technology (custom)

Tissue: Tonsil

Dilution:
IHC: 1:200
CODEX: 1:20

CODEX oligo: 74-ATTO550

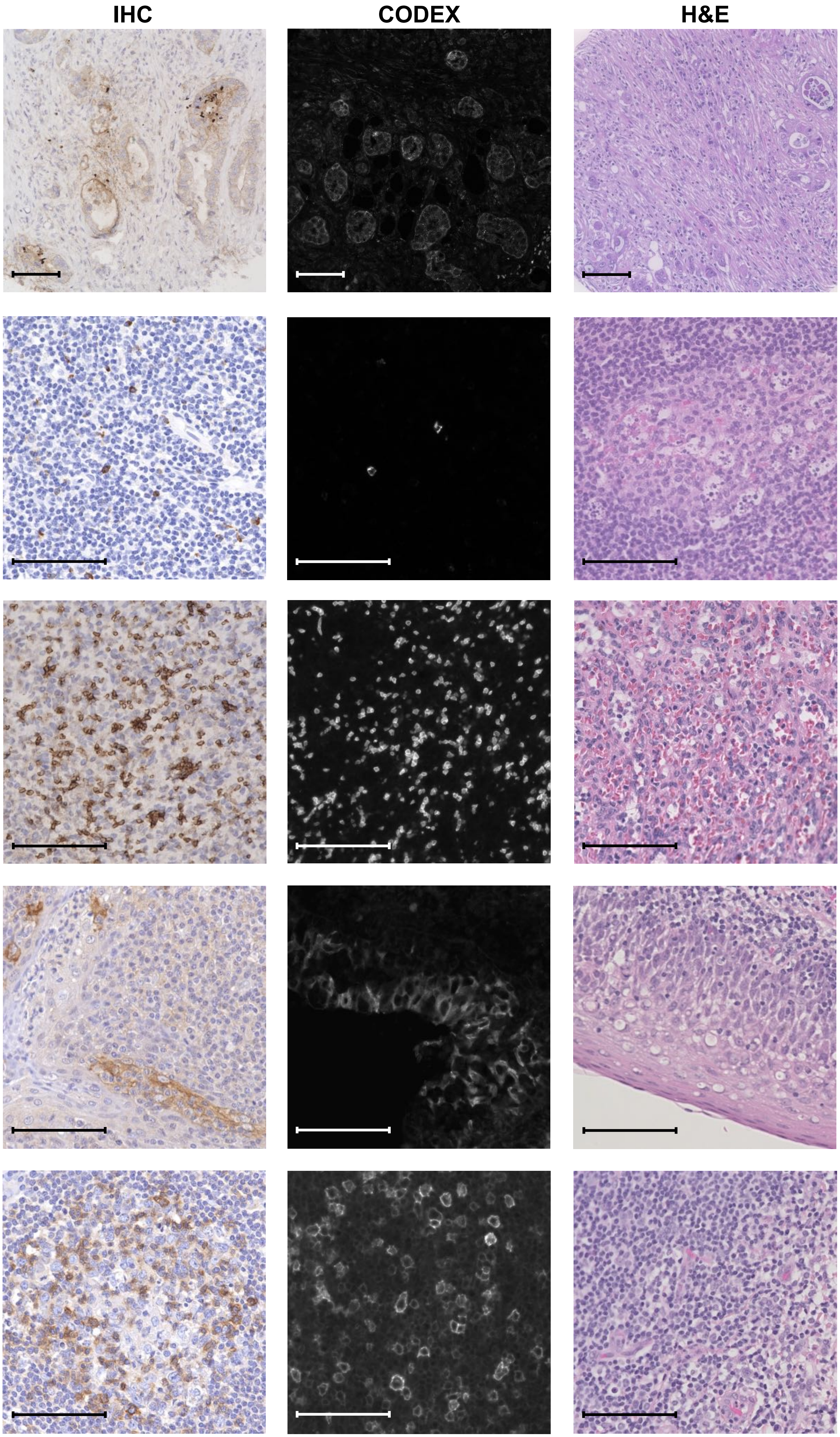

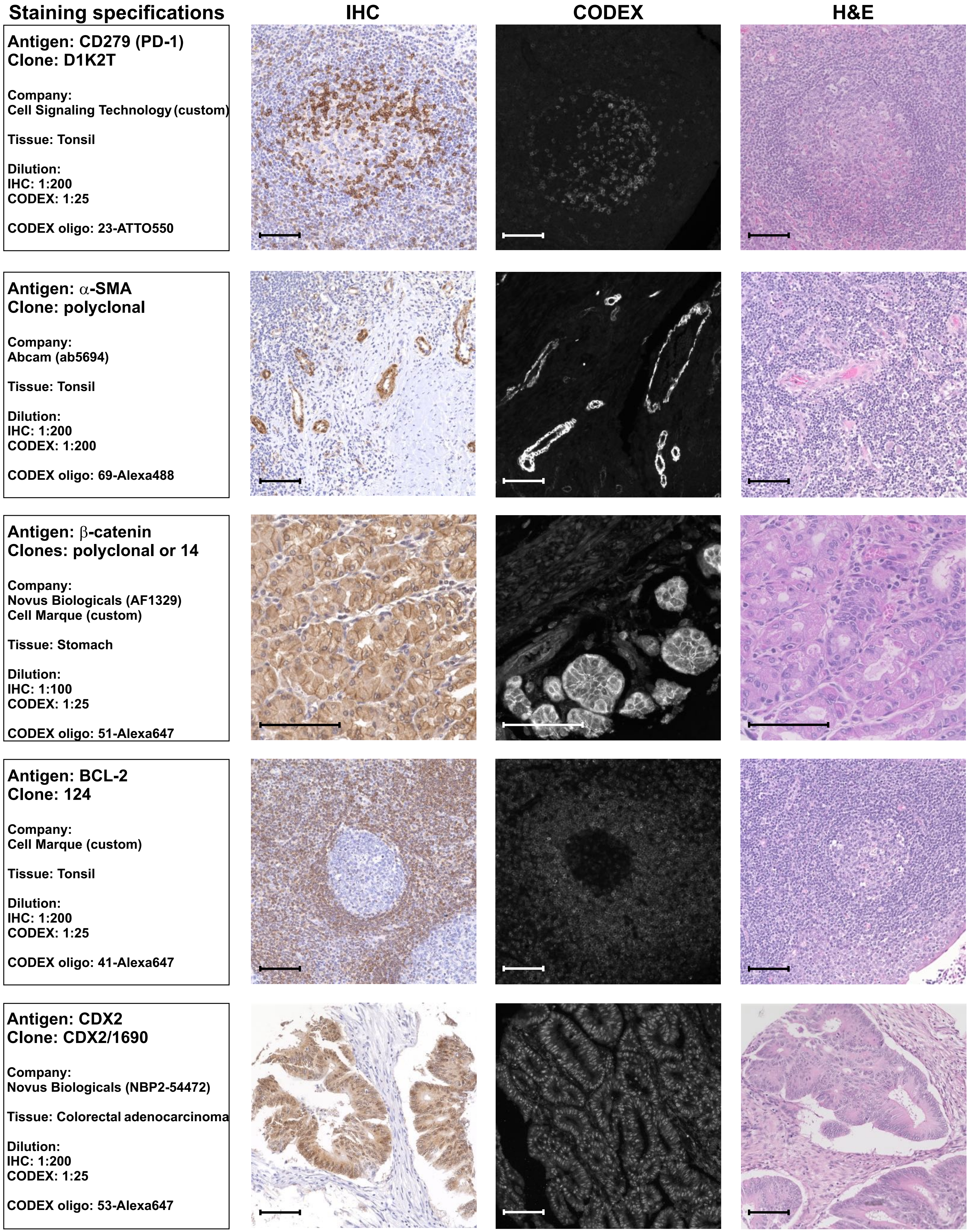
Staining specifications
IHC
CODEX
H&E
Antigen: CD279 (PD-1)
Clone: D1K2T
Company:
Cell Signaling Technology (custom)
Tissue: Tonsil
Dilution:
IHC: 1:200
CODEX: 1:25
CODEX oligo: 23-ATTO550
Antigen: α-SMA
Clone: polyclonal
Company:
Abcam (ab5694)
Tissue: Tonsil
Dilution:
IHC: 1:200
CODEX: 1:200
CODEX oligo: 69-Alexa488
Antigen: β-catenin
Clones: polyclonal or 14
Company:
Novus Biologicals (AF1329)
Cell Marque (custom)
Tissue: Stomach
Dilution:
IHC: 1:100
CODEX: 1:25
CODEX oligo: 51-Alexa647
Antigen: BCL-2
Clone: 124
Company:
Cell Marque (custom)
Tissue: Tonsil
Dilution:
IHC: 1:200
CODEX: 1:25
CODEX oligo: 41-Alexa647
Antigen: CDX2
Clone: CDX2/1690
Company:
Novus Biologicals (NBP2-54472)
Tissue: Colorectal adenocarcinoma
Dilution:
IHC: 1:200
CODEX: 1:25
CODEX oligo: 53-Alexa647

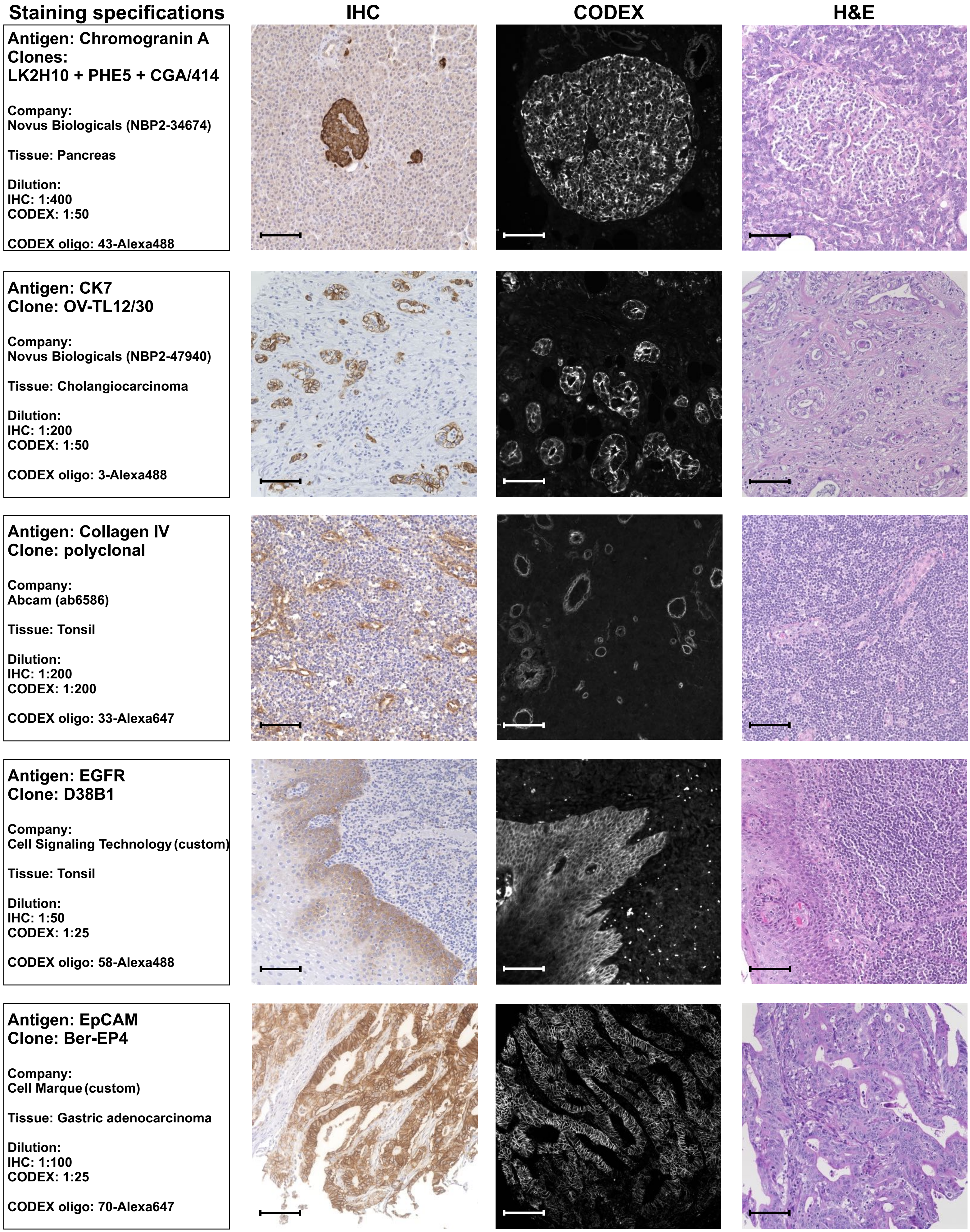
Staining specifications
IHC
CODEX
H&E
Antigen: Chromogranin A
Clones:
LK2H10 + PHE5 + CGA/414
Company:
Novus Biologicals (NBP2-34674)
Tissue: Pancreas
Dilution:
IHC: 1:400
CODEX: 1:50
CODEX oligo: 43-Alexa488
Antigen: CK7
Clone: OV-TL12/30
Company:
Novus Biologicals (NBP2-47940)
Tissue: Cholangiocarcinoma
Dilution:
IHC: 1:200
CODEX: 1:50
CODEX oligo: 3-Alexa488
Antigen: Collagen IV
Clone: polyclonal
Company:
Abcam (ab6586)
Tissue: Tonsil
Dilution:
IHC: 1:200
CODEX: 1:200
CODEX oligo: 33-Alexa647
Antigen: EGFR
Clone: D38B1
Company:
Cell Signaling Technology (custom)
Tissue: Tonsil
Dilution:
IHC: 1:50
CODEX: 1:25
CODEX oligo: 58-Alexa488
Antigen: EpCAM
Clone: Ber-EP4
Company:
Cell Marque (custom)
Tissue: Gastric adenocarcinoma
Dilution:
IHC: 1:100
CODEX: 1:25
CODEX oligo: 70-Alexa647

Staining specifications	IHC	CODEX	H&E

Antigen: FOXP3
Clone: 236A/E7

Company:
Invitrogen (14-4777-80)

Tissue: Tonsil

Dilution:
IHC: 1:20
CODEX: 1:100

CODEX oligo: 61-Alexa647

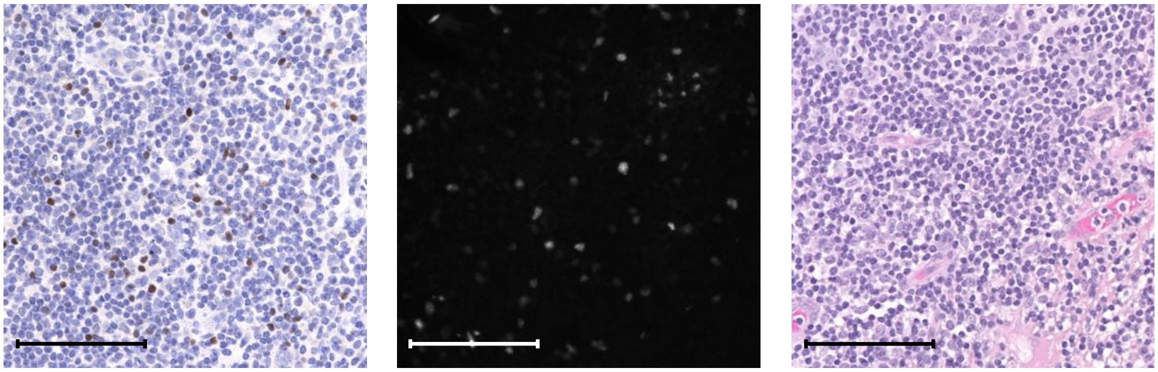

Antigen: GATA3
Clone: L50-823

Company:
Cell Marque (custom)

Tissue: Breast lobular carcinoma

Dilution:
IHC: 1:600
CODEX: 1:100

CODEX oligo: 60-Alexa647

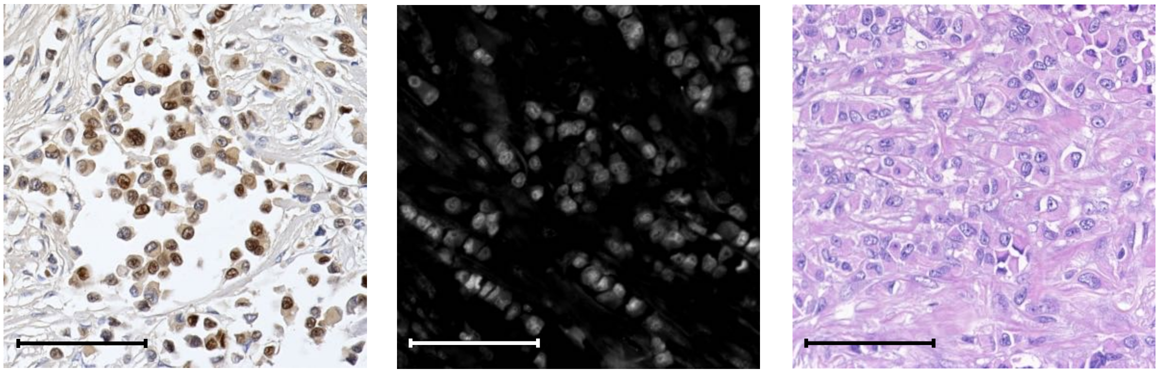

Antigen: GFAP
Clone: 2.2B10

Company:
Invitrogen (130300)

Tissue: Glioblastoma

Dilution:
IHC: 1:100
CODEX: 1:25

CODEX oligo: 46-Alexa488

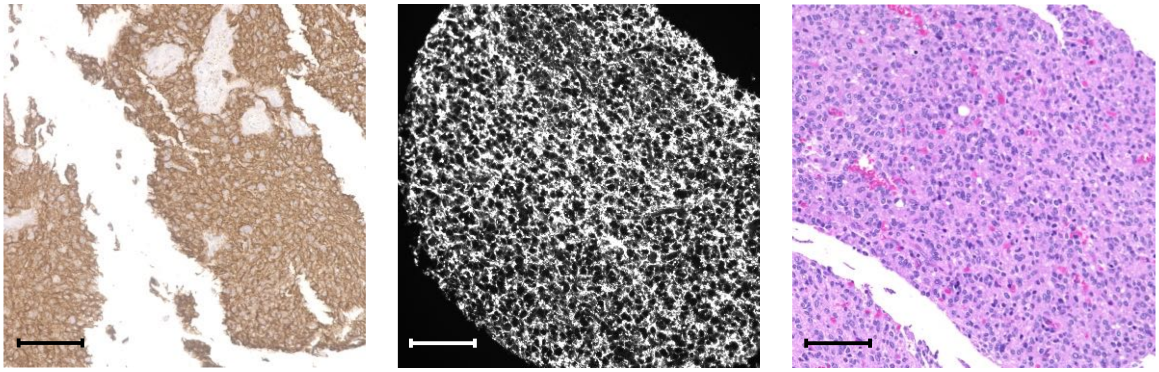

Antigen: Granzyme B
Clone: EPR20129-217

Company:
Abcam (ab219803)

Tissue: Tonsil

Dilution:
IHC: 1:200
CODEX: 1:100

CODEX oligo: 81-Alexa647

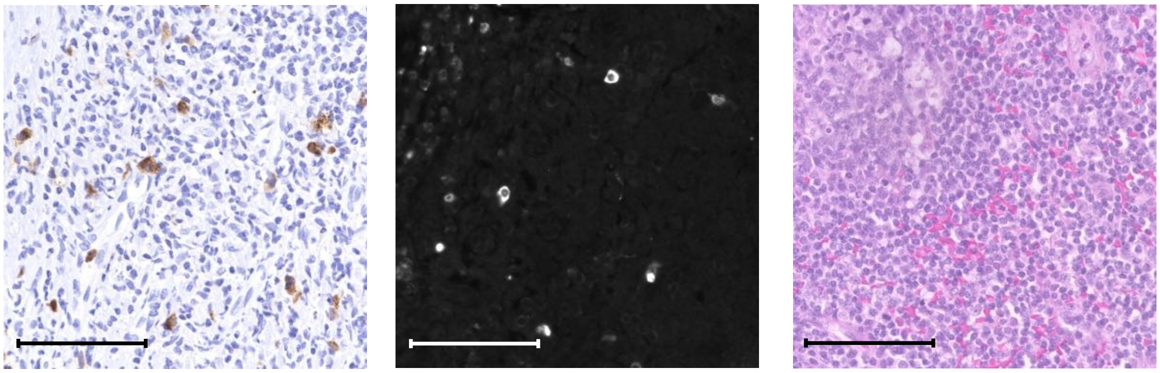

Antigen: Hep-Par 1
Clone: OCH1E5

Company:
Santa Cruz Bio (sc-58693)

Tissue: Hepatocellular carcinoma

Dilution:
IHC: 1:25
CODEX: 1:100

CODEX oligo: 28-Alexa488

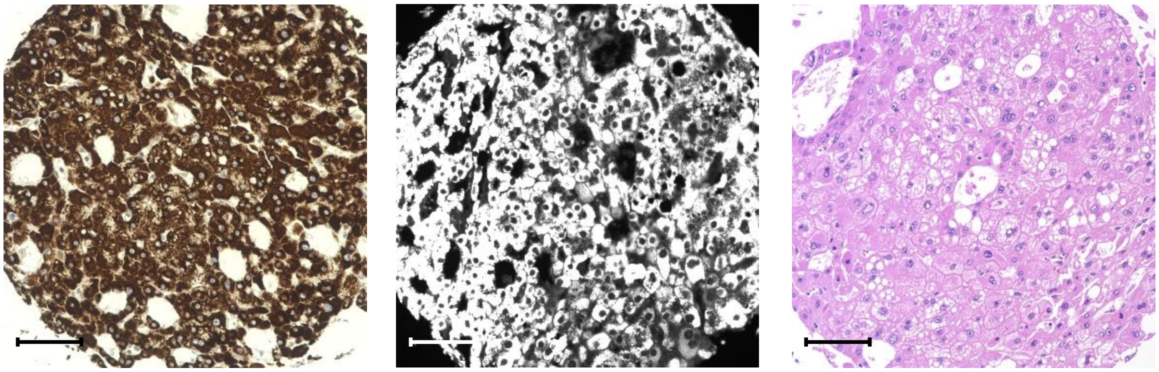

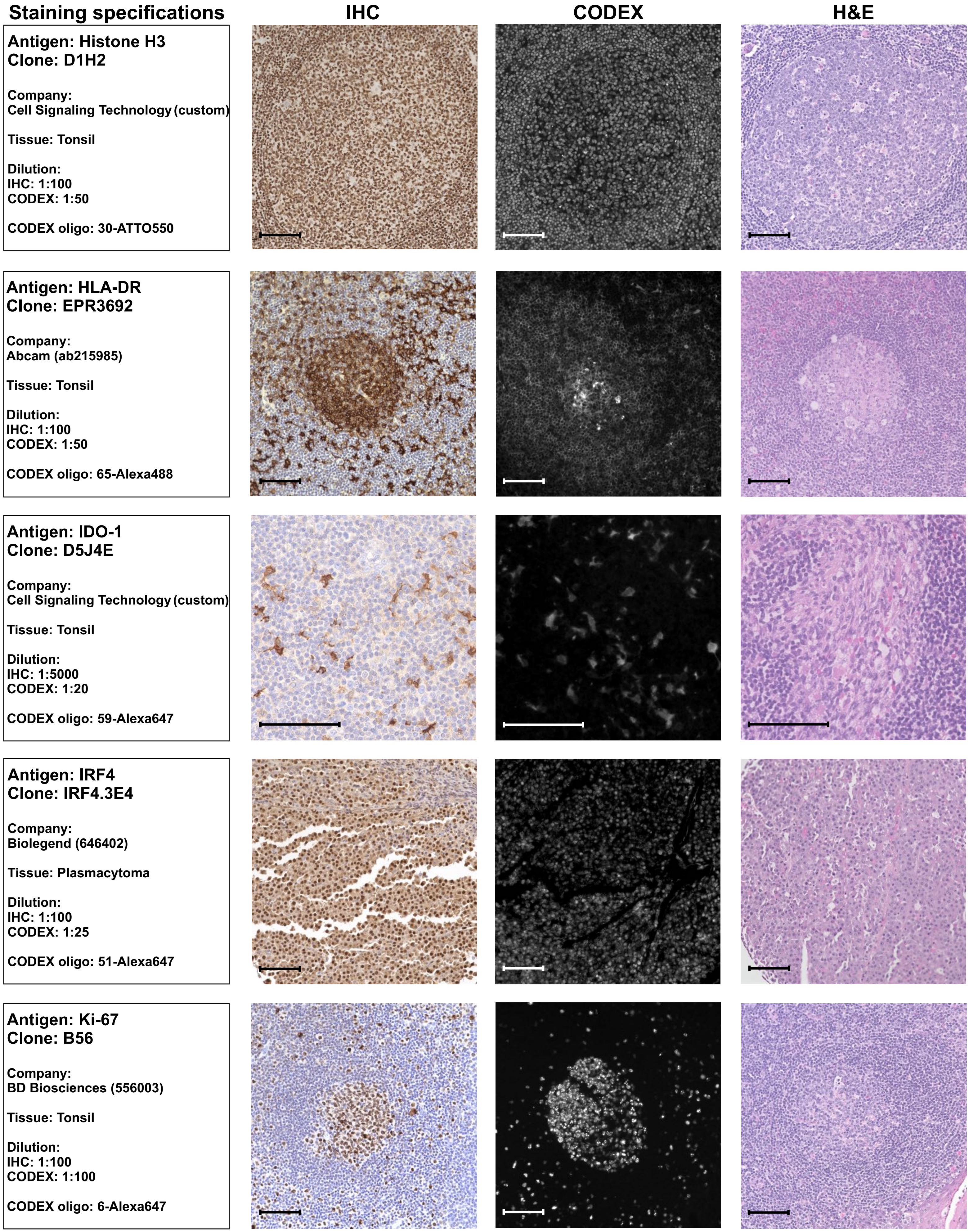
Staining specifications
IHC
CODEX
H&E
Antigen: Histone H3
Clone: D1H2
Company:
Cell Signaling Technology (custom)
Tissue: Tonsil
Dilution:
IHC: 1:100
CODEX: 1:50
CODEX oligo: 30-ATTO550
Antigen: HLA-DR
Clone: EPR3692
Company:
Abcam (ab215985)
Tissue: Tonsil
Dilution:
IHC: 1:100
CODEX: 1:50
CODEX oligo: 65-Alexa488
Antigen: IDO-1
Clone: D5J4E
Company:
Cell Signaling Technology (custom)
Tissue: Tonsil
Dilution:
IHC: 1:5000
CODEX: 1:20
CODEX oligo: 59-Alexa647
Antigen: IRF4
Clone: IRF4.3E4
Company:
Biolegend (646402)
Tissue: Plasmacytoma
Dilution:
IHC: 1:100
CODEX: 1:25
CODEX oligo: 51-Alexa647
Antigen: Ki-67
Clone: B56
Company:
BD Biosciences (556003)
Tissue: Tonsil
Dilution:
IHC: 1:100
CODEX: 1:100
CODEX oligo: 6-Alexa647

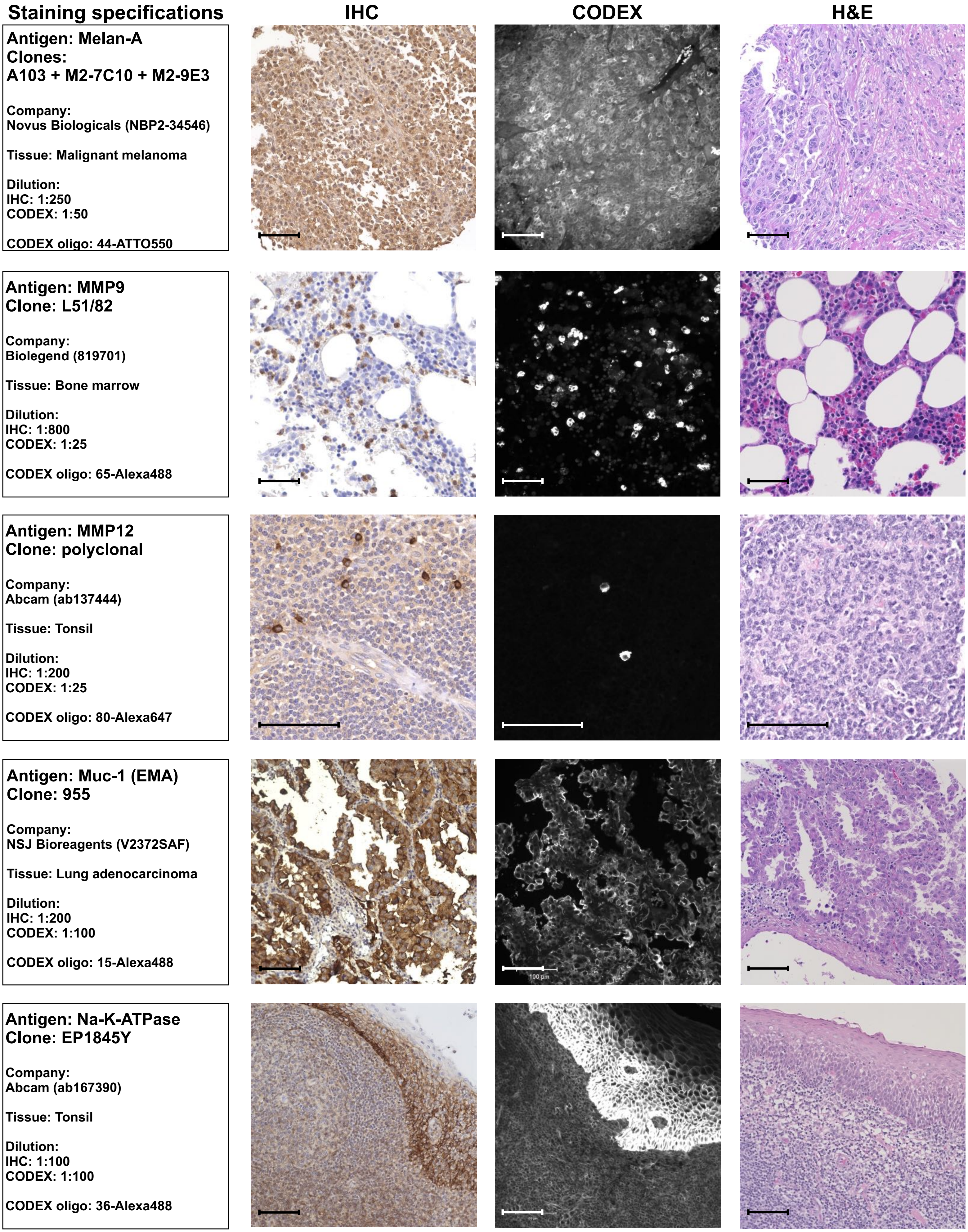
Staining specifications
IHC
CODEX
H&E
Antigen: Melan-A
Clones:
A103 + M2-7C10 + M2-9E3
Company:
Novus Biologicals (NBP2-34546)
Tissue: Malignant melanoma
Dilution:
IHC: 1:250
CODEX: 1:50
CODEX oligo: 44-ATTO550
Antigen: MMP9
Clone: L51/82
Company:
Biolegend (819701)
Tissue: Bone marrow
Dilution:
IHC: 1:800
CODEX: 1:25
CODEX oligo: 65-Alexa488
Antigen: MMP12
Clone: polyclonal
Company:
Abcam (ab137444)
Tissue: Tonsil
Dilution:
IHC: 1:200
CODEX: 1:25
CODEX oligo: 80-Alexa647
Antigen: Muc-1 (EMA)
Clone: 955
Company:
NSJ Bioreagents (V2372SAF)
Tissue: Lung adenocarcinoma
Dilution:
IHC: 1:200
CODEX: 1:100
CODEX oligo: 15-Alexa488
Antigen: Na-K-ATPase
Clone: EP1845Y
Company:
Abcam (ab167390)
Tissue: Tonsil
Dilution:
IHC: 1:100
CODEX: 1:100
CODEX oligo: 36-Alexa488

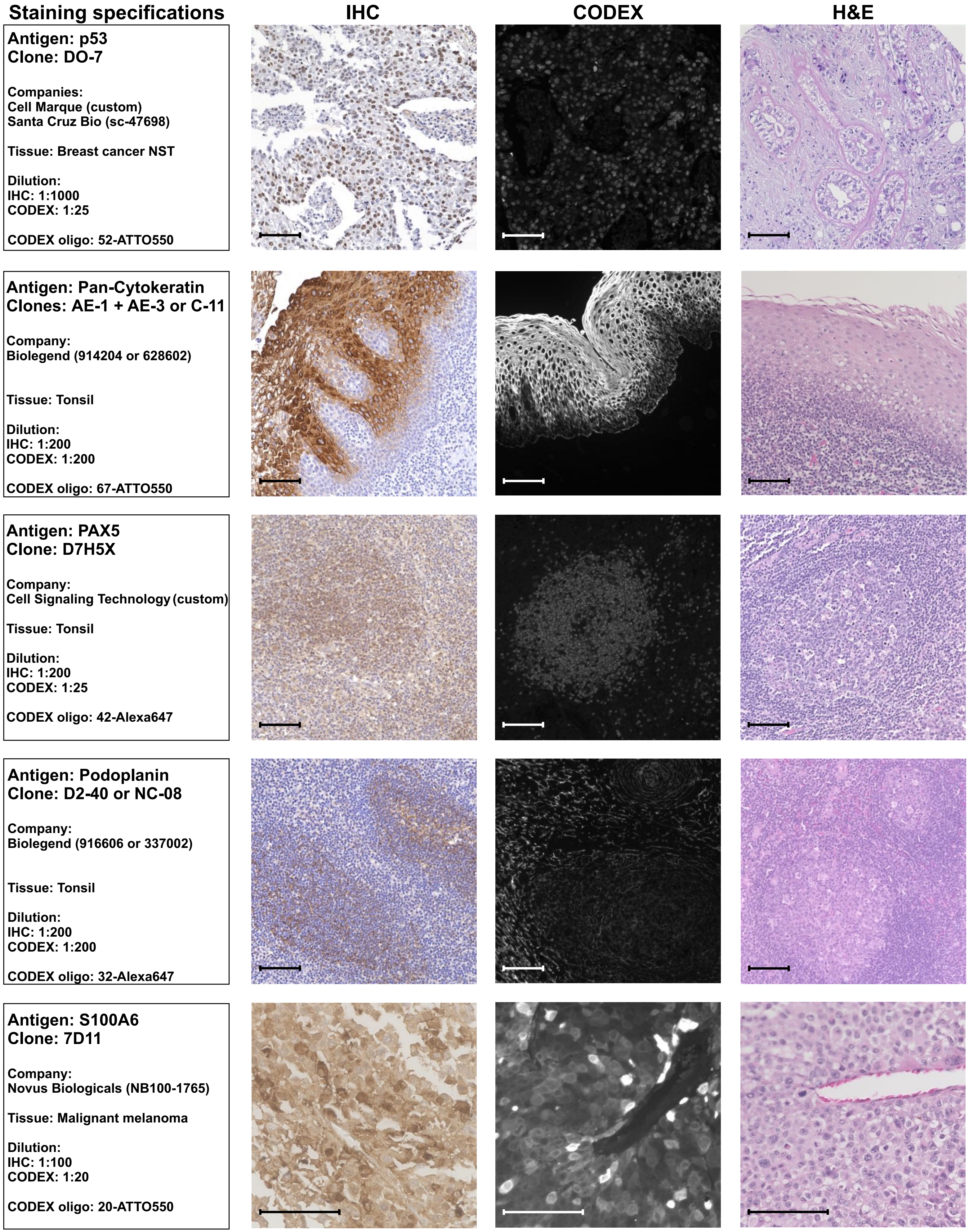
Staining specifications
IHC
CODEX
H&E
Antigen: p53
Clone: DO-7
Companies:
Cell Marque (custom)
Santa Cruz Bio (sc-47698)
Tissue: Breast cancer NST
Dilution:
IHC: 1:1000
CODEX: 1:25
CODEX oligo: 52-ATTO550
Antigen: Pan-Cytokeratin
Clones: AE-1 + AE-3 or C-11
Company:
Biolegend (914204 or 628602)
Tissue: Tonsil
Dilution:
IHC: 1:200
CODEX: 1:200
CODEX oligo: 67-ATTO550
Antigen: PAX5
Clone: D7H5X
Company:
Cell Signaling Technology (custom)
Tissue: Tonsil
Dilution:
IHC: 1:200
CODEX: 1:25
CODEX oligo: 42-Alexa647
Antigen: Podoplanin
Clone: D2-40 or NC-08
Company:
Biolegend (916606 or 337002)
Tissue: Tonsil
Dilution:
IHC: 1:200
CODEX: 1:200
CODEX oligo: 32-Alexa647
Antigen: S100A6
Clone: 7D11
Company:
Novus Biologicals (NB100-1765)
Tissue: Malignant melanoma
Dilution:
IHC: 1:100
CODEX: 1:20
CODEX oligo: 20-ATTO550

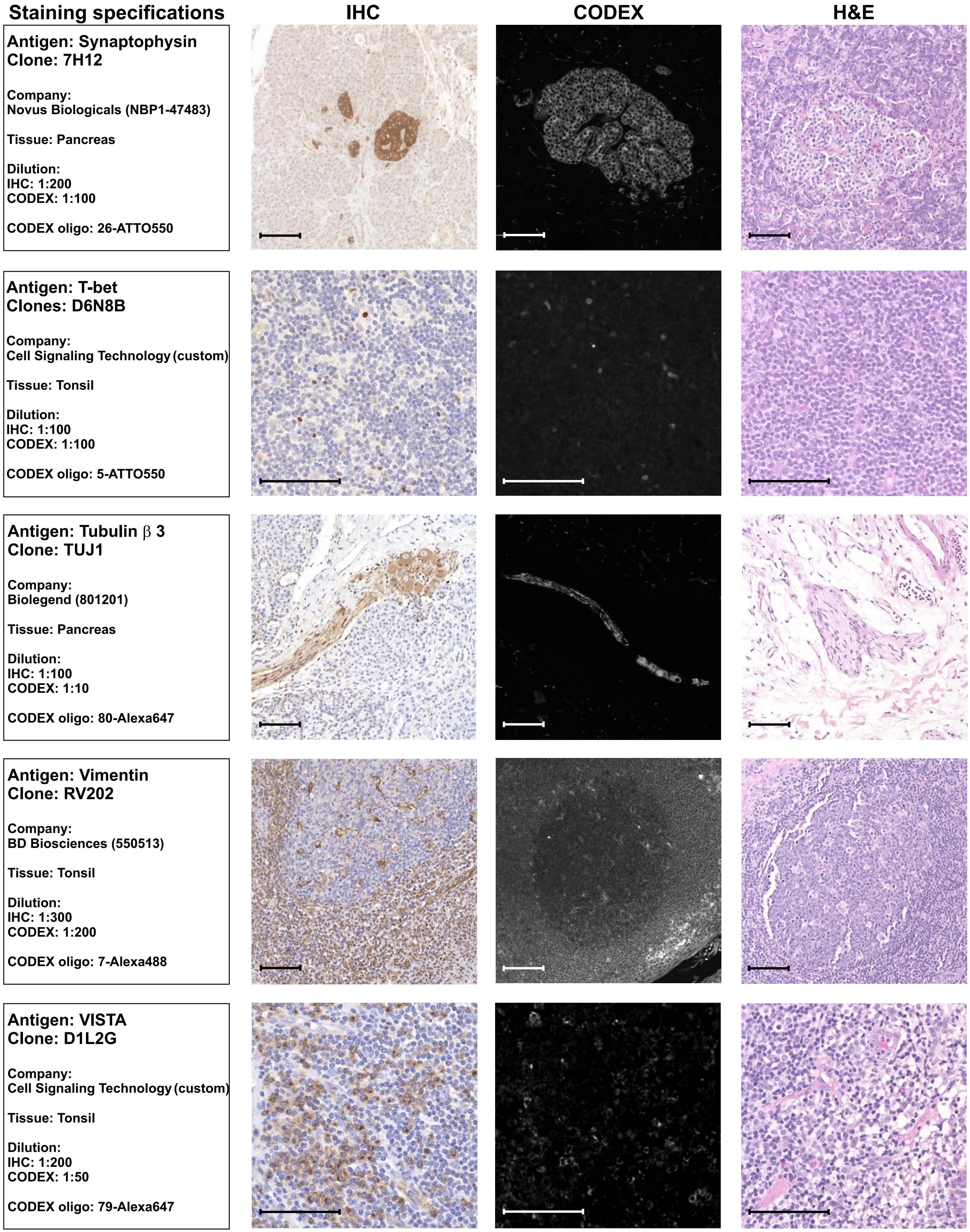
Staining specifications
IHC
CODEX
H&E
Antigen: Synaptophysin
Clone: 7H12
Company:
Novus Biologicals (NBP1-47483)
Tissue: Pancreas
Dilution:
IHC: 1:200
CODEX: 1:100
CODEX oligo: 26-ATTO550
Antigen: T-bet
Clones: D6N8B
Company:
Cell Signaling Technology (custom)
Tissue: Tonsil
Dilution:
IHC: 1:100
CODEX: 1:100
CODEX oligo: 5-ATTO550
Antigen: Tubulin β 3
Clone: TUJ1
Company:
Biolegend (801201)
Tissue: Pancreas
Dilution:
IHC: 1:100
CODEX: 1:10
CODEX oligo: 80-Alexa647
Antigen: Vimentin
Clone: RV202
Company:
BD Biosciences (550513)
Tissue: Tonsil
Dilution:
IHC: 1:300
CODEX: 1:200
CODEX oligo: 7-Alexa488
Antigen: VISTA
Clone: D1L2G
Company:
Cell Signaling Technology (custom)
Tissue: Tonsil
Dilution:
IHC: 1:200
CODEX: 1:50
CODEX oligo: 79-Alexa647

Data S2. Hematoxylin & Eosin (H&E) Stainings of TMAs and Corresponding Cellular Neighborhoods, Related to Figures 2-4 and Table S1. (A) Representative H&E-stained section of the multi-tumor TMA. For details of tissues and tumor types, see Table S1. **(B-C)** Representative H&E-stained sections of CRC TMAs A and B. Cores are arranged according to patient number (1-35), with two cores per patient per TMA (4 cores per patient in total). **(B)**, TMA A and **(C)**, TMA B. See Table S1 for detailed patients' characteristics. (A-C) Scale bars, 1 mm. **(D-G)** Cellular neighborhoods and corresponding H&E stainings for all cores of both CRC TMAs. Scale bars, 0.3 mm.

A H&E-stain of multi-tumor TMA

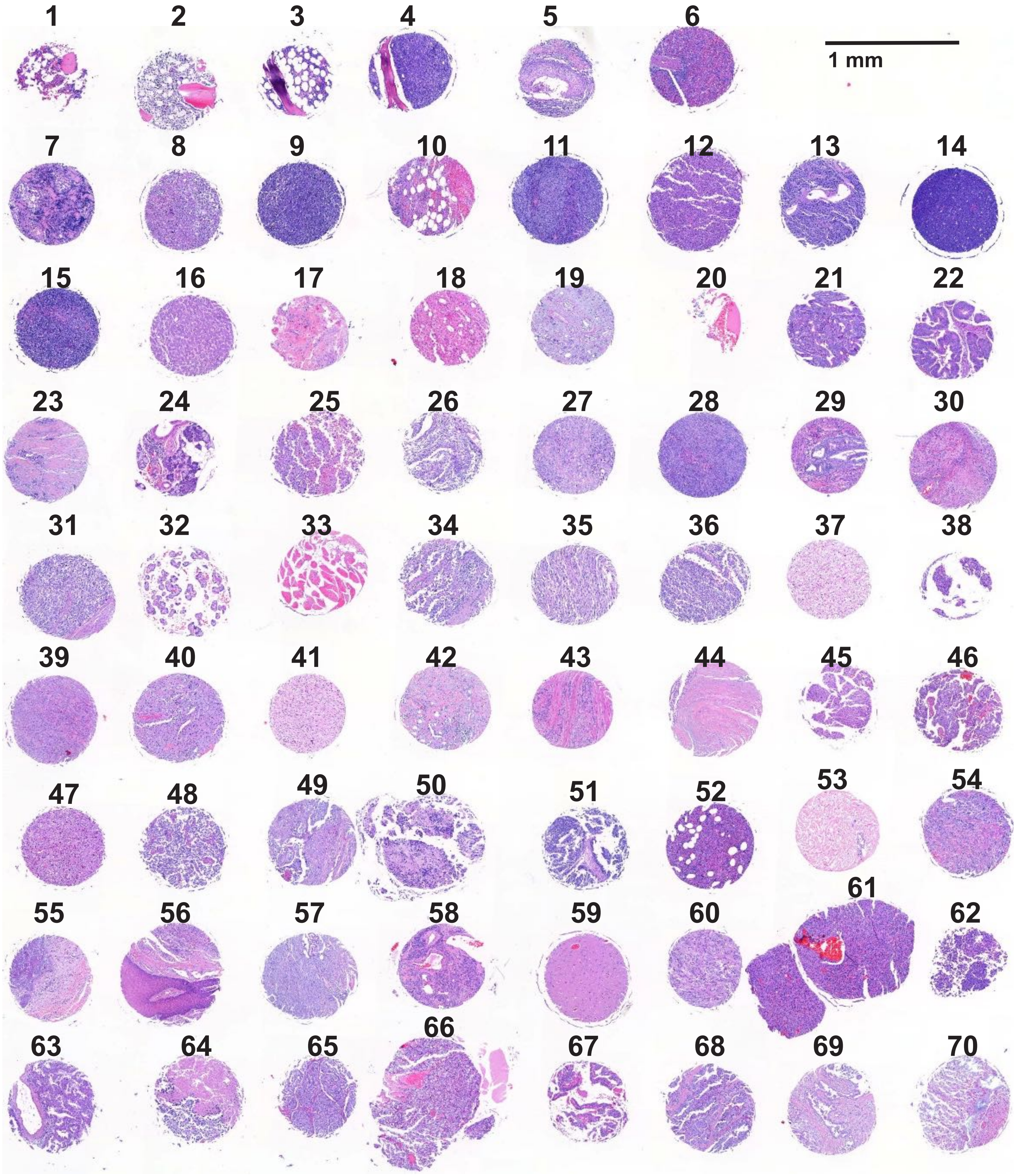

B H&E-stain of CRC TMA A

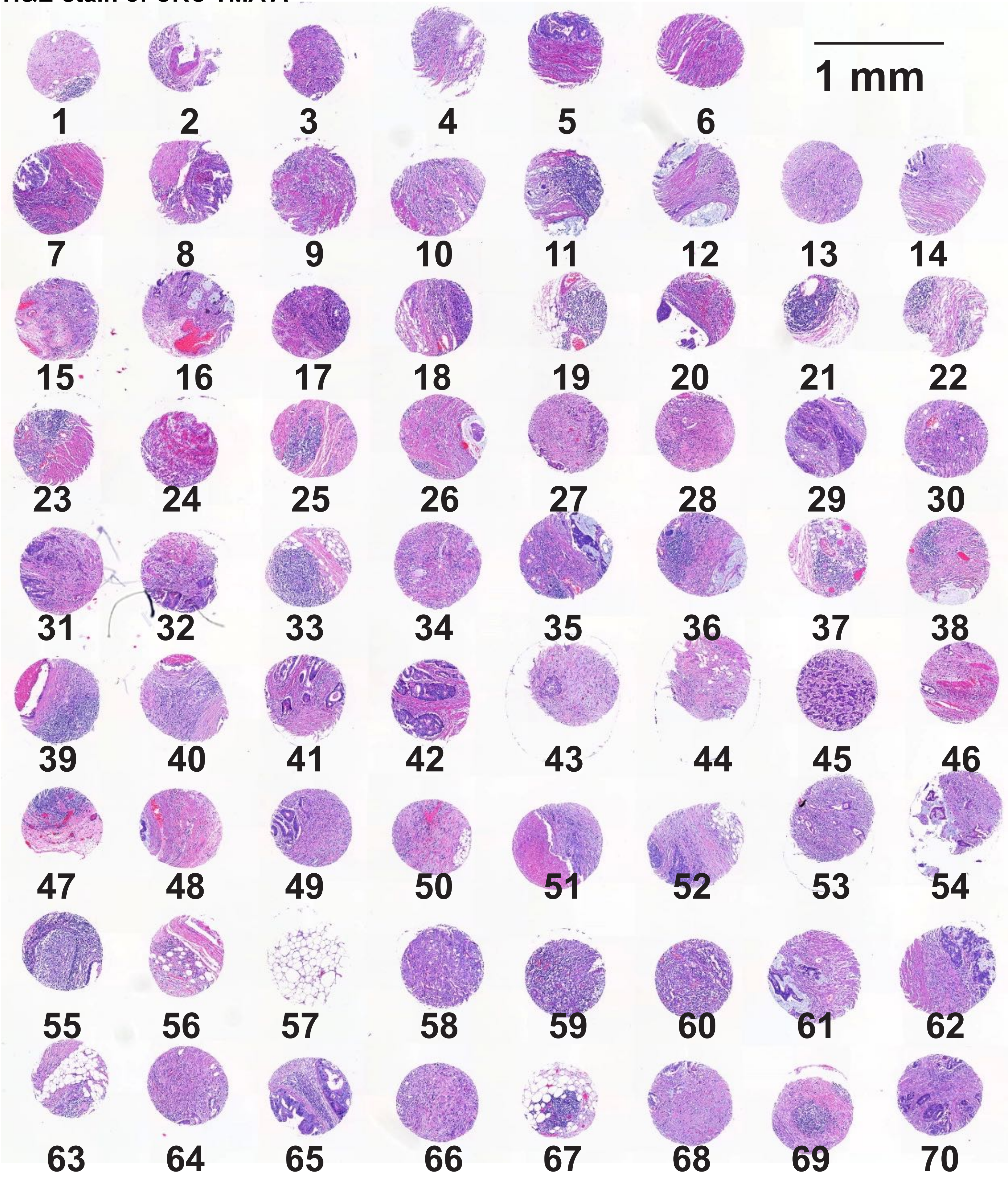

C H&E-stain of CRC TMA B

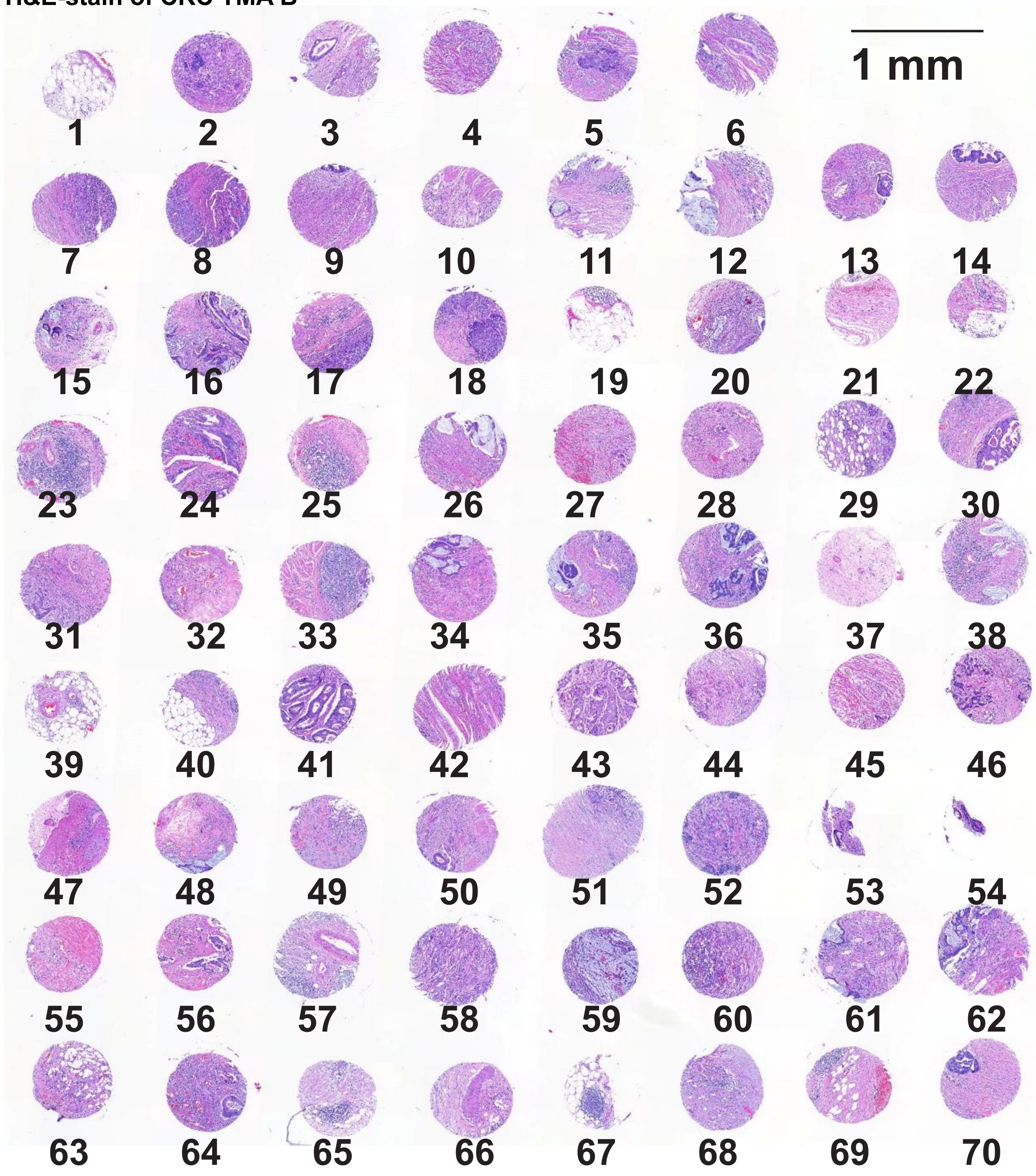

CRC TMA A

D

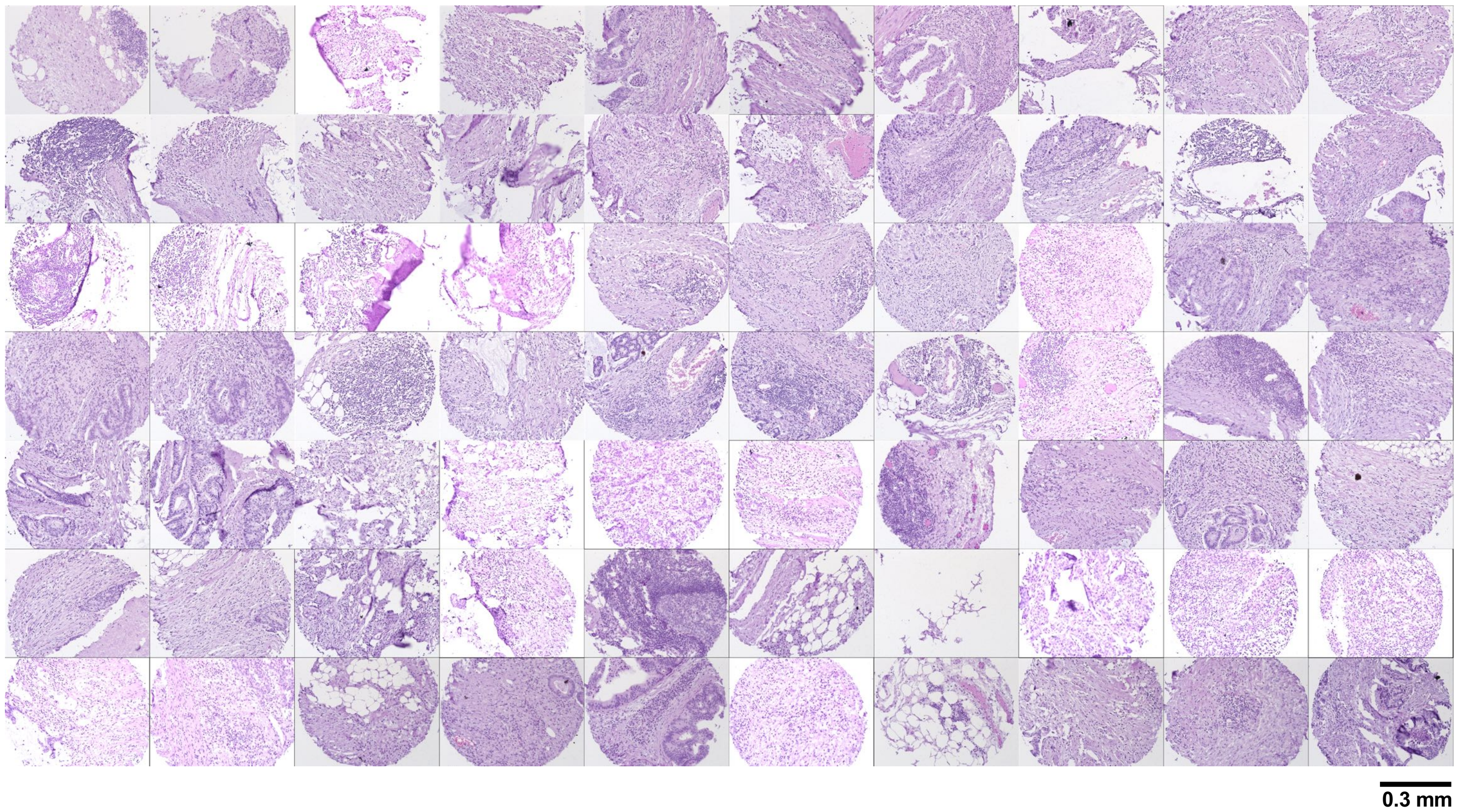
0.3 mm

E

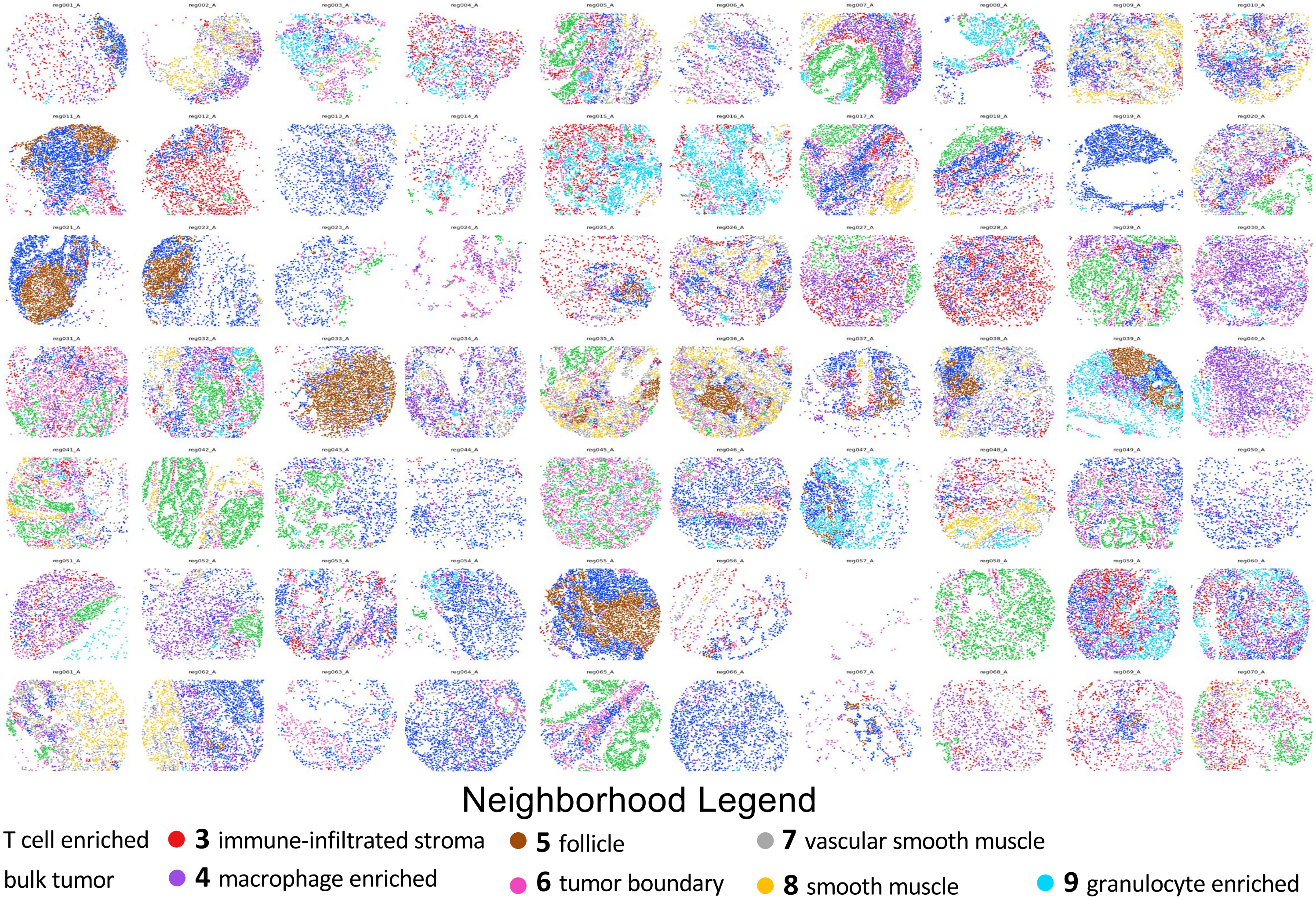
Neighborhood Legend
1 T cell enriched
2 bulk tumor
3 immune-infiltrated stroma
4 macrophage enriched
5 follicle
6 tumor boundary
7 vascular smooth muscle
8 smooth muscle
9 granulocyte enriched

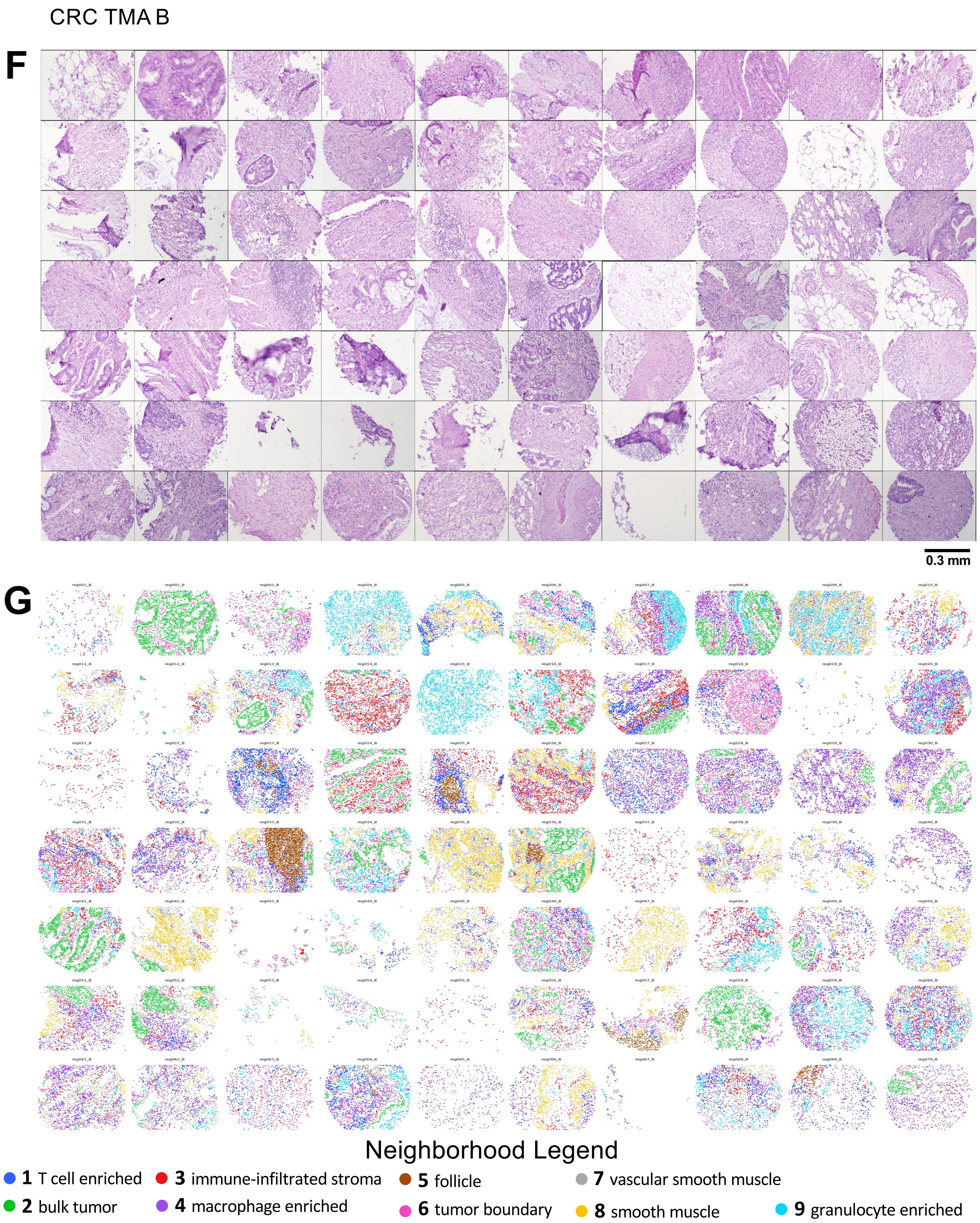
CRC TMA B
F
0.3 mm
G
Neighborhood Legend
1 T cell enriched
2 bulk tumor
3 immune-infiltrated stroma
4 macrophage enriched
5 follicle
6 tumor boundary
7 vascular smooth muscle
8 smooth muscle
9 granulocyte enriched

Data S3. Minimal Spanning Trees (MSTs) and Mean Maker Expression of 28 CRC Clusters from Unsupervised X-shift Clustering, Related to Figure 3 and STAR Methods. MSTs show the relationship between the clusters (edges and distances), their sizes, and their mean marker expression, for each patient group. MSTs were generated in VorteX for each marker analyzed. Group 1, CLR patients; group 2, DII patients.

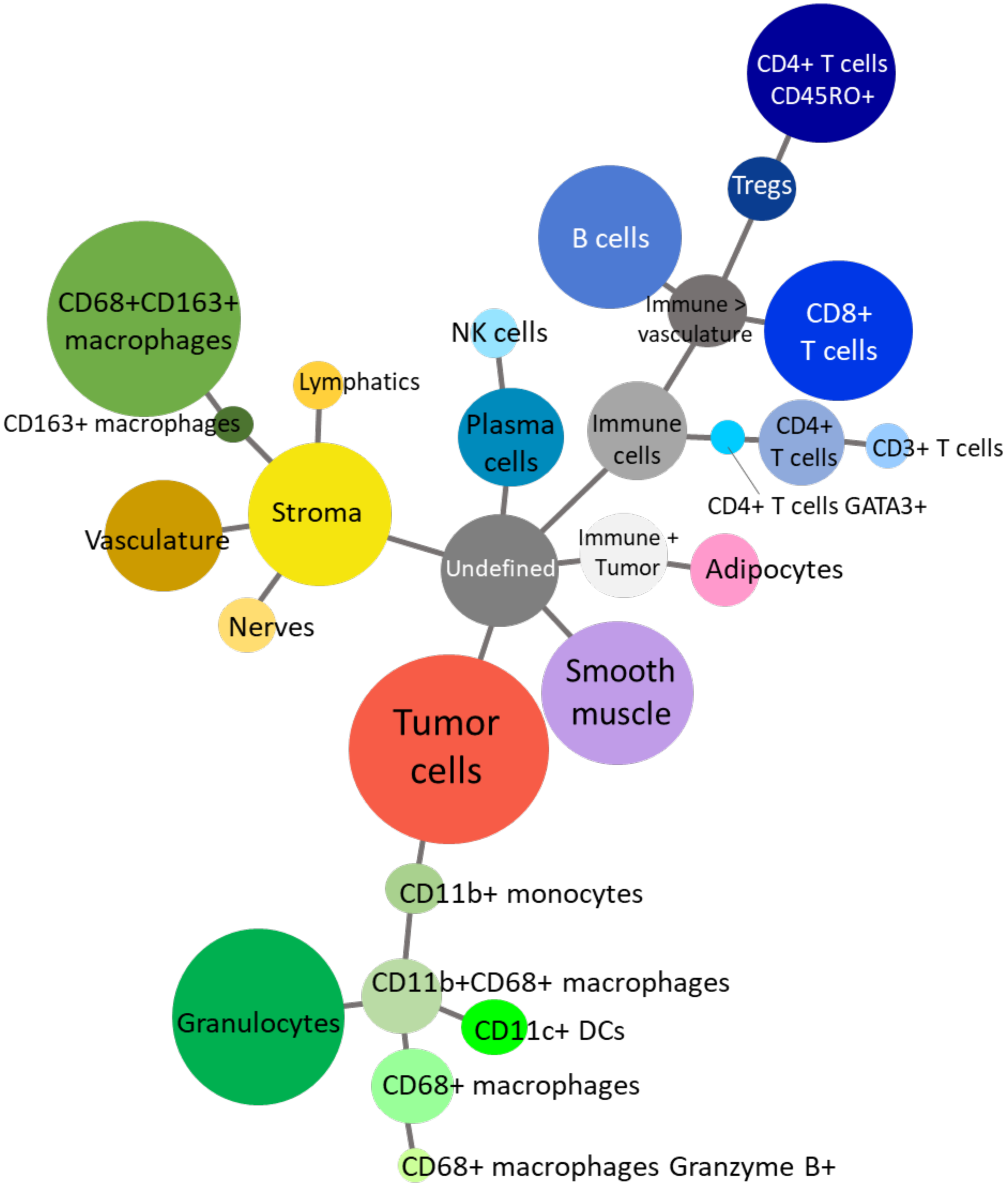

28 unique clusters

CD2

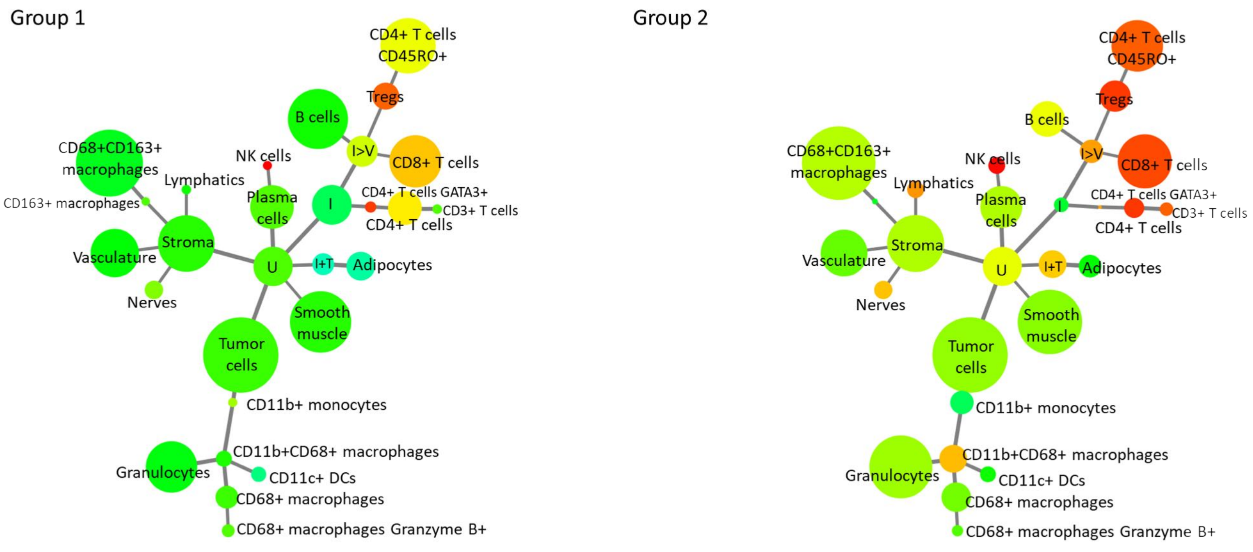
Group 1
CD4+ T cells CD45RO+
Tregs
B cells
I>V
CD68+CD163+ macrophages
NK cells
CD8+ T cells
Lymphatics
Plasma cells
I
CD4+ T cells GATA3+
CD3+ T cells
CD4+ T cells
CD163+ macrophages
Stroma
Vasculature
U
I+T
Adipocytes
Nerves
Smooth muscle
Tumor cells
CD11b+ monocytes
CD11b+CD68+ macrophages
Granulocytes
CD11c+ DCs
CD68+ macrophages
CD68+ macrophages Granzyme B+
Group 2
CD4+ T cells CD45RO+
Tregs
B cells
I>V
CD68+CD163+ macrophages
NK cells
CD8+ T cells
Lymphatics
Plasma cells
I
CD4+ T cells GATA3+
CD3+ T cells
CD4+ T cells
Stroma
Vasculature
U
I+T
Adipocytes
Nerves
Smooth muscle
Tumor cells
CD11b+ monocytes
CD11b+CD68+ macrophages
Granulocytes
CD11c+ DCs
CD68+ macrophages
CD68+ macrophages Granzyme B+

CD3

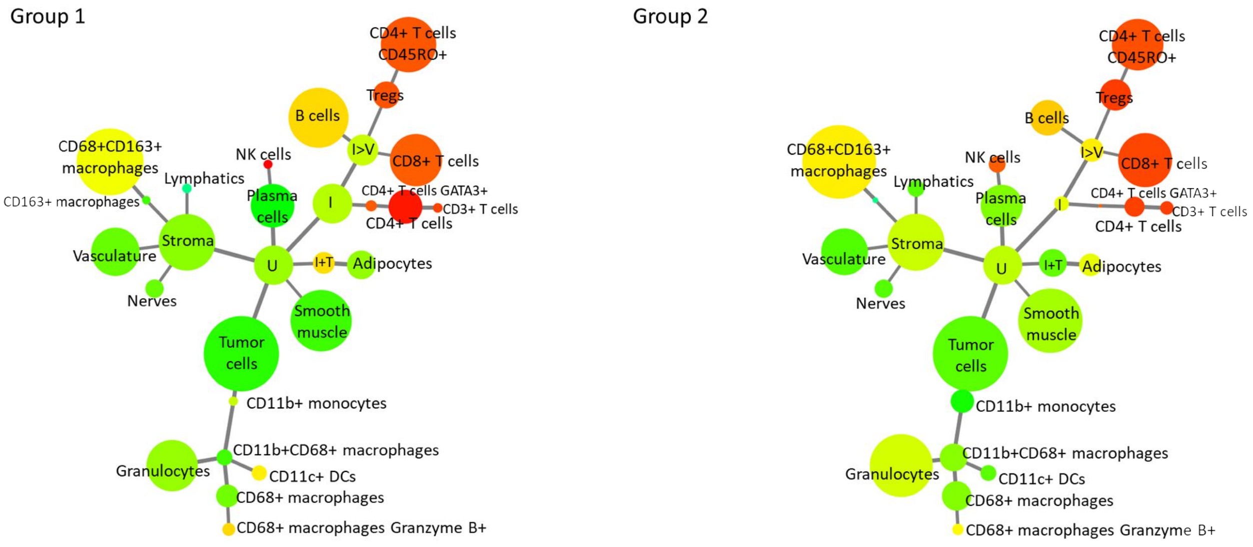
Group 1
CD4+ T cells CD45RO+
Tregs
B cells
I>V
CD68+CD163+ macrophages
NK cells
CD8+ T cells
Lymphatics
Plasma cells
I
CD4+ T cells GATA3+
CD3+ T cells
CD4+ T cells
CD163+ macrophages
Stroma
Vasculature
U
I+T
Adipocytes
Nerves
Smooth muscle
Tumor cells
CD11b+ monocytes
CD11b+CD68+ macrophages
Granulocytes
CD11c+ DCs
CD68+ macrophages
CD68+ macrophages Granzyme B+
Group 2
CD4+ T cells CD45RO+
Tregs
B cells
I>V
CD68+CD163+ macrophages
NK cells
CD8+ T cells
Lymphatics
Plasma cells
I
CD4+ T cells GATA3+
CD3+ T cells
CD4+ T cells
Stroma
Vasculature
U
I+T
Adipocytes
Nerves
Smooth muscle
Tumor cells
CD11b+ monocytes
CD11b+CD68+ macrophages
Granulocytes
CD11c+ DCs
CD68+ macrophages
CD68+ macrophages Granzyme B+

CD4

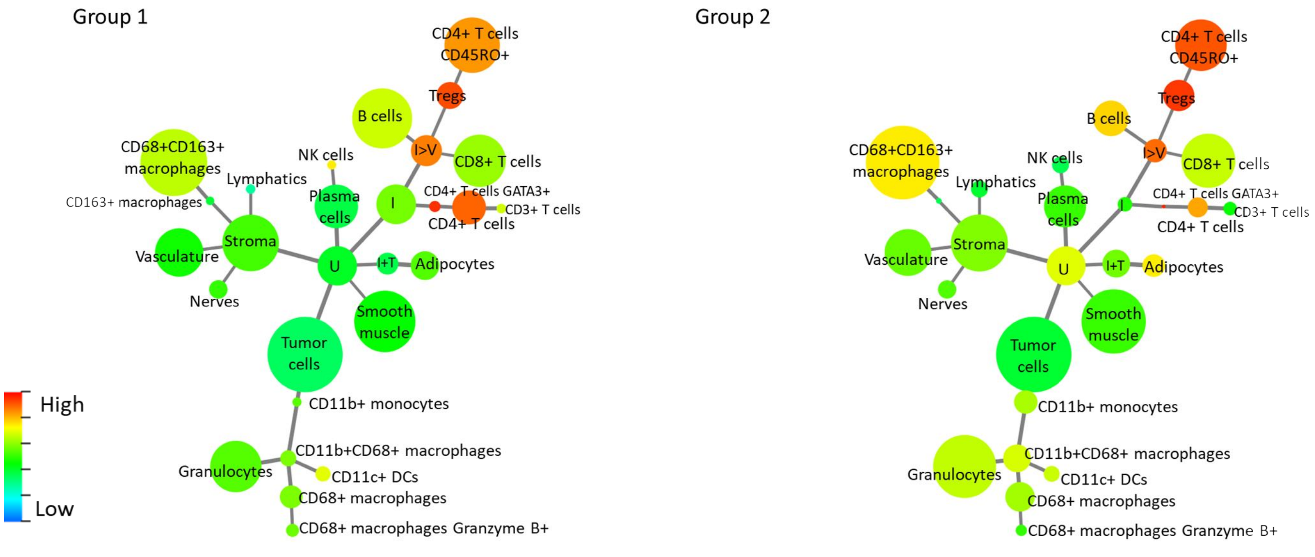
Group 1
CD4+ T cells CD45RO+
Tregs
B cells
I>V
CD68+CD163+ macrophages
NK cells
CD8+ T cells
Lymphatics
Plasma cells
I
CD4+ T cells GATA3+
CD3+ T cells
CD4+ T cells
CD163+ macrophages
Stroma
Vasculature
U
I+T
Adipocytes
Nerves
Smooth muscle
Tumor cells
CD11b+ monocytes
CD11b+CD68+ macrophages
Granulocytes
CD11c+ DCs
CD68+ macrophages
CD68+ macrophages Granzyme B+
Group 2
CD4+ T cells CD45RO+
Tregs
B cells
I>V
CD68+CD163+ macrophages
NK cells
CD8+ T cells
Lymphatics
Plasma cells
I
CD4+ T cells GATA3+
CD3+ T cells
CD4+ T cells
Stroma
Vasculature
U
I+T
Adipocytes
Nerves
Smooth muscle
Tumor cells
CD11b+ monocytes
CD11b+CD68+ macrophages
Granulocytes
CD11c+ DCs
CD68+ macrophages
CD68+ macrophages Granzyme B+
High
Low

CD5

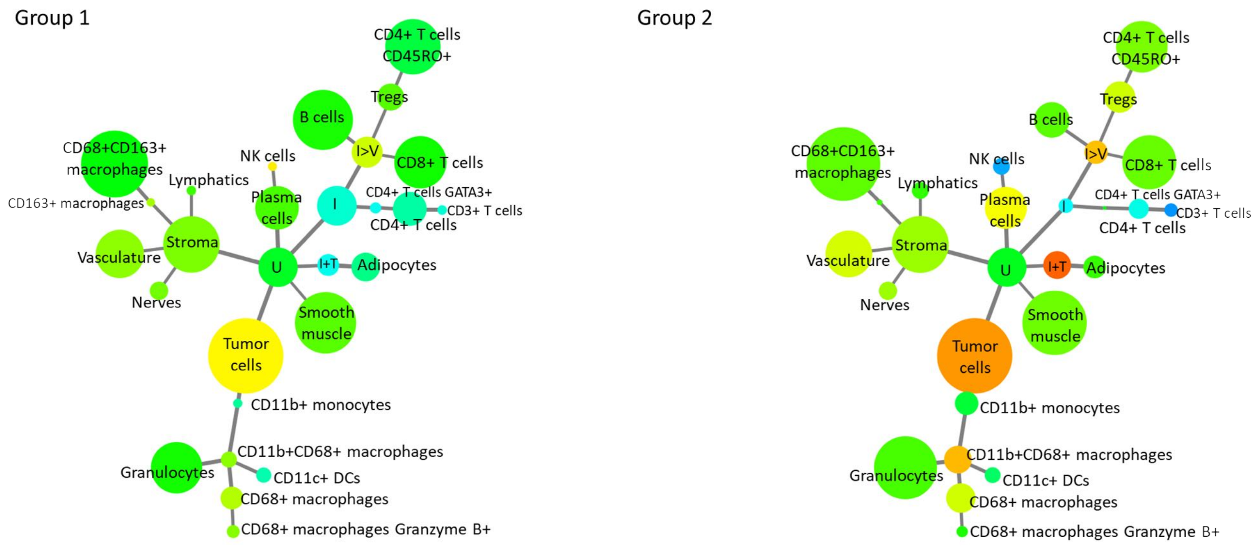

Group 1
CD4+ T cells
CD45RO+
Tregs
B cells
I>V
CD8+ T cells
CD68+CD163+
macrophages
NK cells
Lymphatics
Plasma
cells
I
CD4+ T cells GATA3+
CD3+ T cells
CD4+ T cells
CD163+ macrophages
Stroma
Vasculature
U
I+T
Adipocytes
Nerves
Smooth
muscle
Tumor
cells
CD11b+ monocytes
CD11b+CD68+ macrophages
Granulocytes
CD11c+ DCs
CD68+ macrophages
CD68+ macrophages Granzyme B+
Group 2
CD4+ T cells
CD45RO+
Tregs
B cells
I>V
CD8+ T cells
CD68+CD163+
macrophages
NK cells
Lymphatics
Plasma
cells
I
CD4+ T cells GATA3+
CD3+ T cells
CD4+ T cells
Stroma
Vasculature
U
I+T
Adipocytes
Nerves
Smooth
muscle
Tumor
cells
CD11b+ monocytes
CD11b+CD68+ macrophages
Granulocytes
CD11c+ DCs
CD68+ macrophages
CD68+ macrophages Granzyme B+

CD7

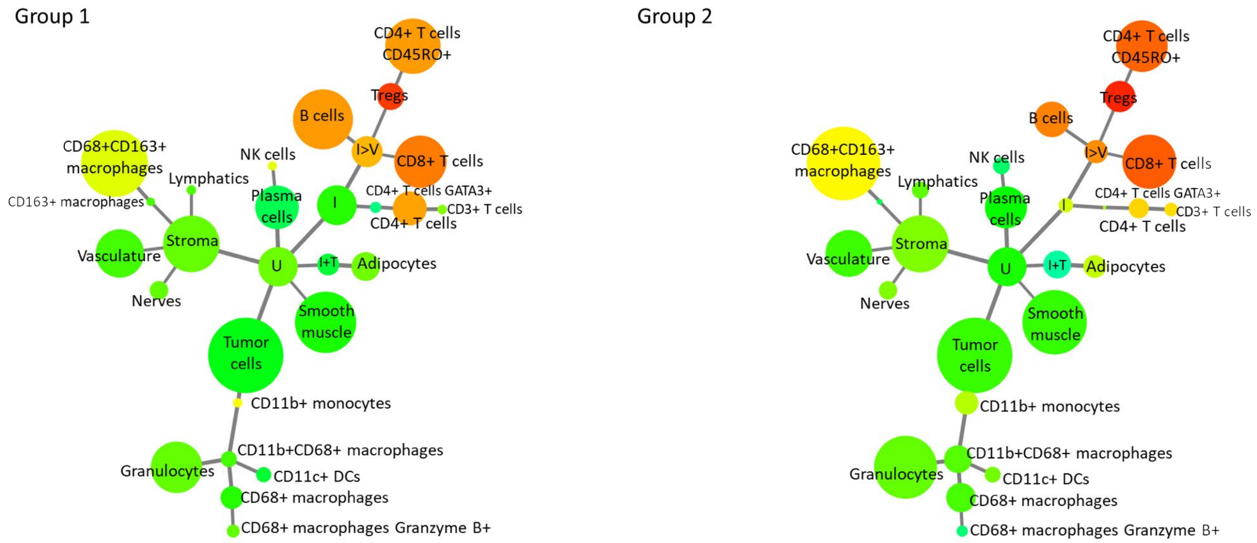

Group 1
CD4+ T cells
CD45RO+
Tregs
B cells
I>V
CD8+ T cells
CD68+CD163+
macrophages
NK cells
Lymphatics
Plasma
cells
I
CD4+ T cells GATA3+
CD3+ T cells
CD4+ T cells
CD163+ macrophages
Stroma
Vasculature
U
I+T
Adipocytes
Nerves
Smooth
muscle
Tumor
cells
CD11b+ monocytes
CD11b+CD68+ macrophages
Granulocytes
CD11c+ DCs
CD68+ macrophages
CD68+ macrophages Granzyme B+
Group 2
CD4+ T cells
CD45RO+
Tregs
B cells
I>V
CD8+ T cells
CD68+CD163+
macrophages
NK cells
Lymphatics
Plasma
cells
I
CD4+ T cells GATA3+
CD3+ T cells
CD4+ T cells
Stroma
Vasculature
U
I+T
Adipocytes
Nerves
Smooth
muscle
Tumor
cells
CD11b+ monocytes
CD11b+CD68+ macrophages
Granulocytes
CD11c+ DCs
CD68+ macrophages
CD68+ macrophages Granzyme B+

CD8

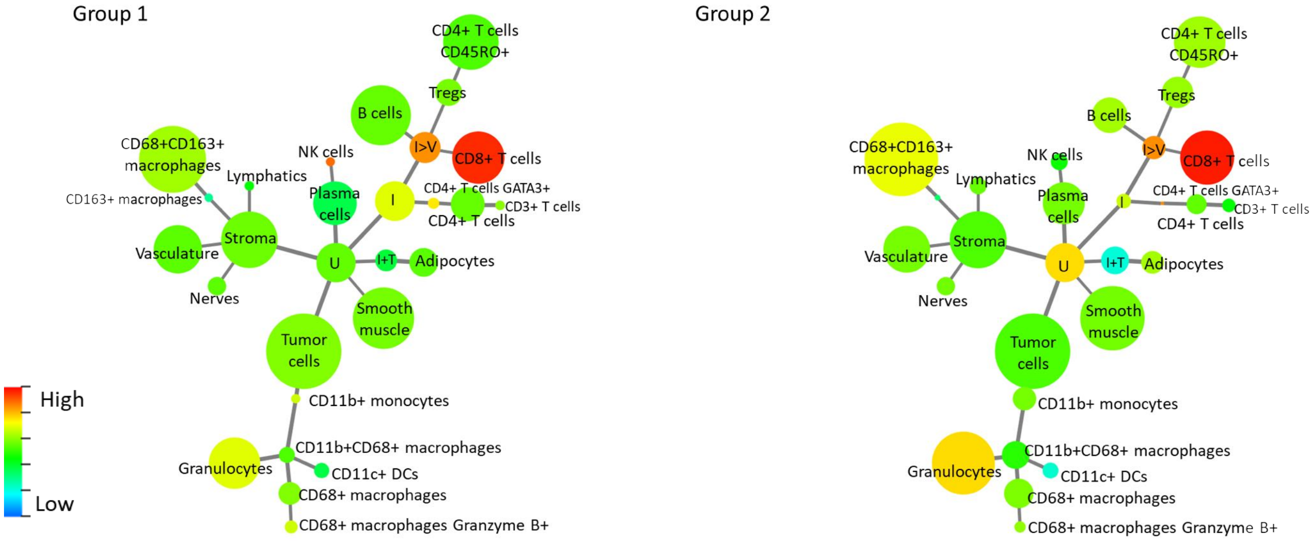

Group 1
CD4+ T cells
CD45RO+
Tregs
B cells
I>V
CD8+ T cells
CD68+CD163+
macrophages
NK cells
Lymphatics
Plasma
cells
I
CD4+ T cells GATA3+
CD3+ T cells
CD4+ T cells
CD163+ macrophages
Stroma
Vasculature
U
I+T
Adipocytes
Nerves
Smooth
muscle
Tumor
cells
CD11b+ monocytes
CD11b+CD68+ macrophages
Granulocytes
CD11c+ DCs
CD68+ macrophages
CD68+ macrophages Granzyme B+
Group 2
CD4+ T cells
CD45RO+
Tregs
B cells
I>V
CD8+ T cells
CD68+CD163+
macrophages
NK cells
Lymphatics
Plasma
cells
I
CD4+ T cells GATA3+
CD3+ T cells
CD4+ T cells
Stroma
Vasculature
U
I+T
Adipocytes
Nerves
Smooth
muscle
Tumor
cells
CD11b+ monocytes
CD11b+CD68+ macrophages
Granulocytes
CD11c+ DCs
CD68+ macrophages
CD68+ macrophages Granzyme B+
High
Low

CD11b

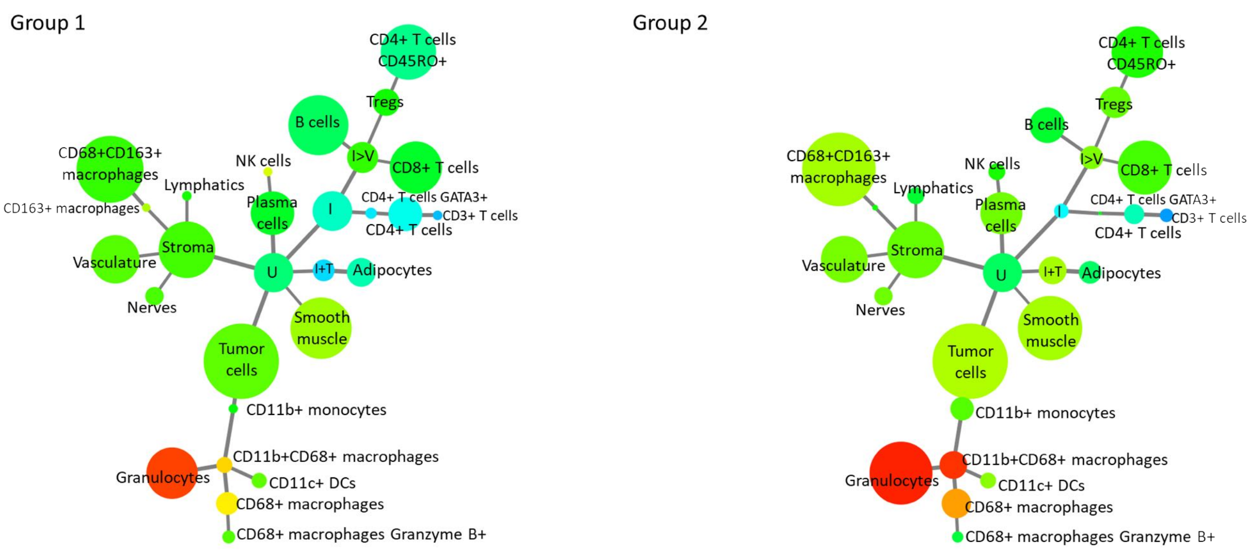

CD11c

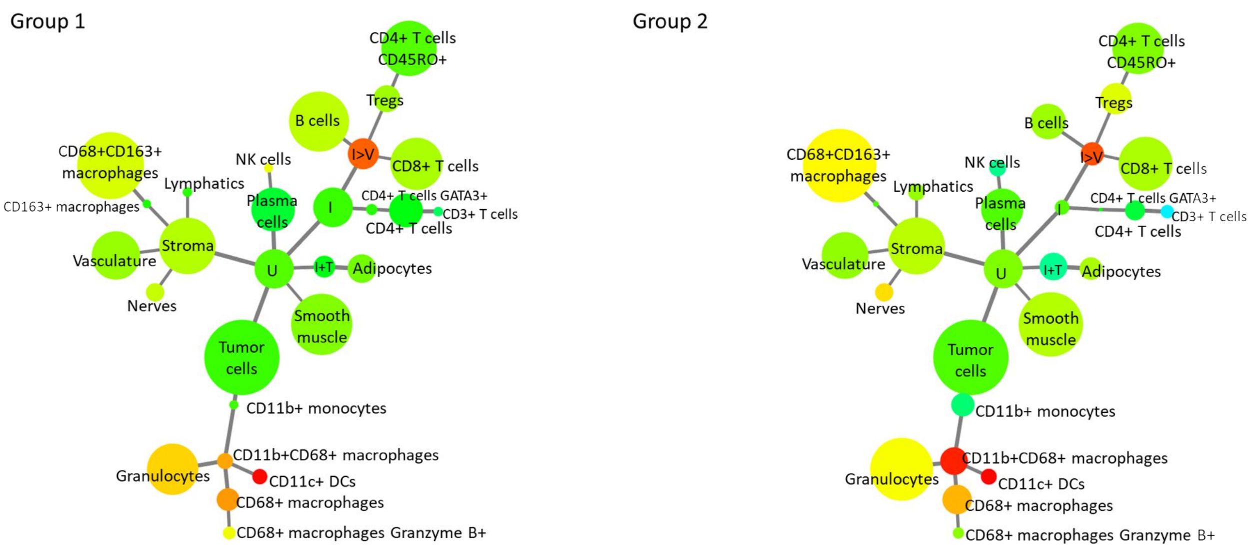

CD15

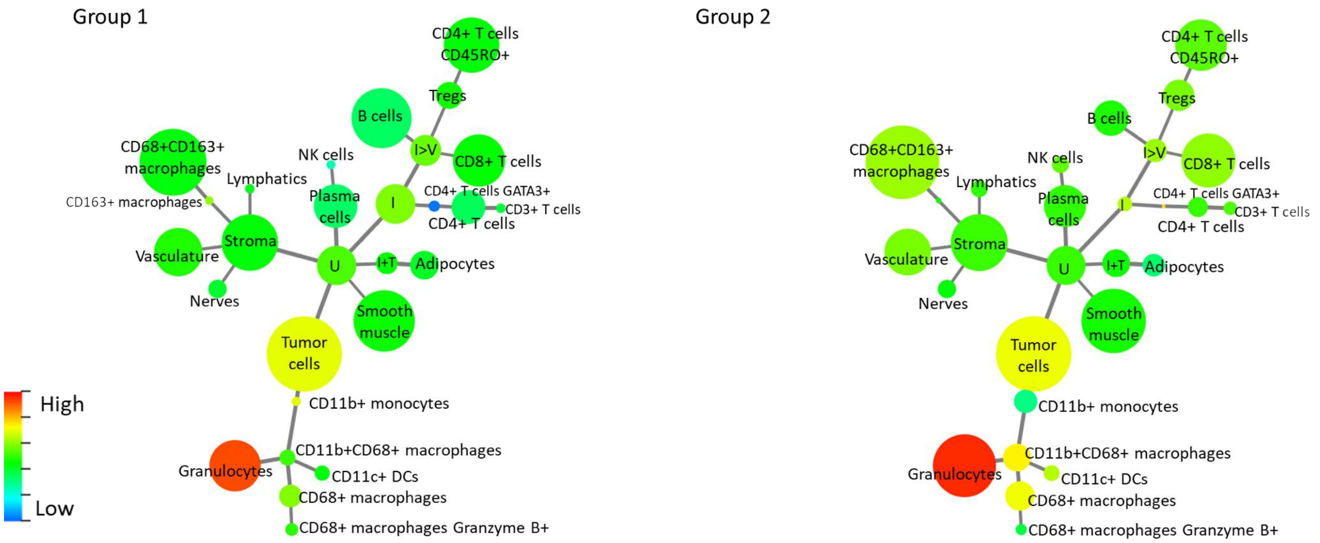

CD20

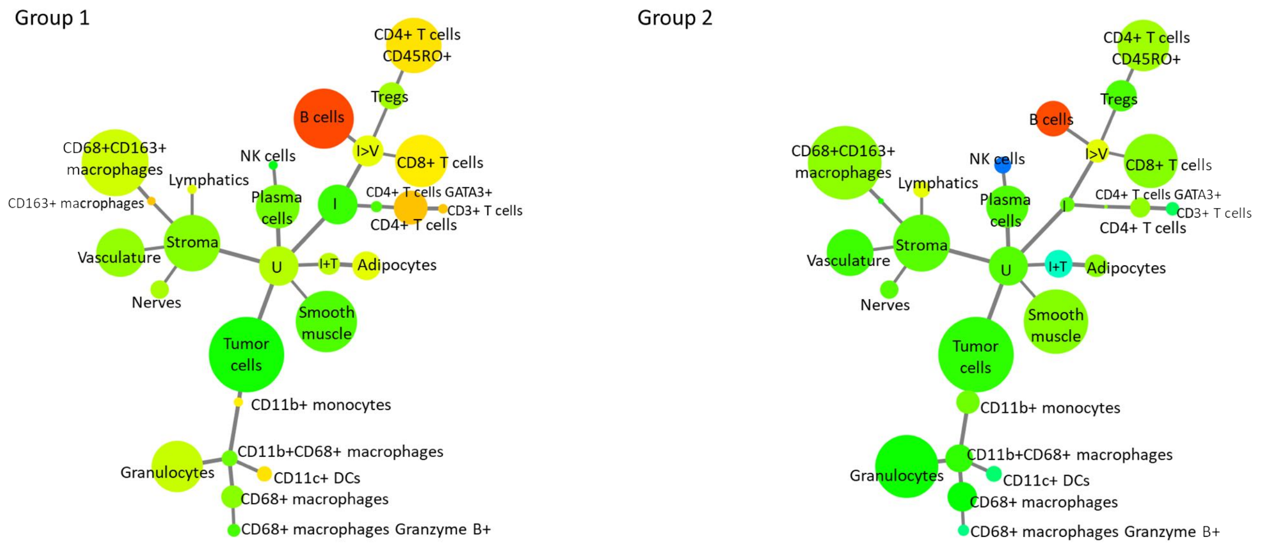

CD21

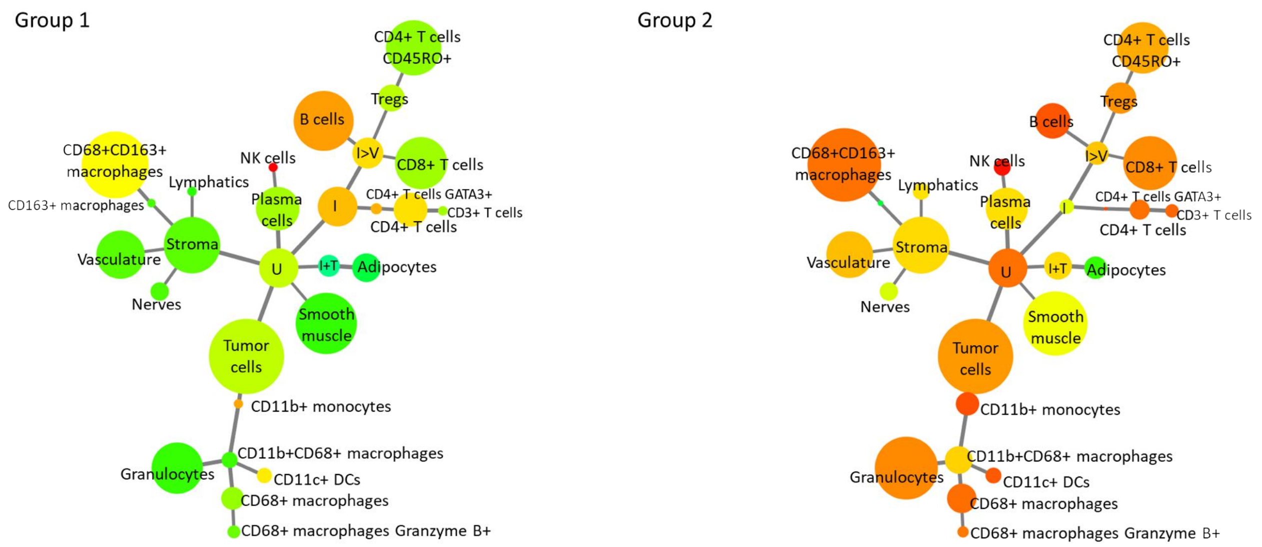

CD25

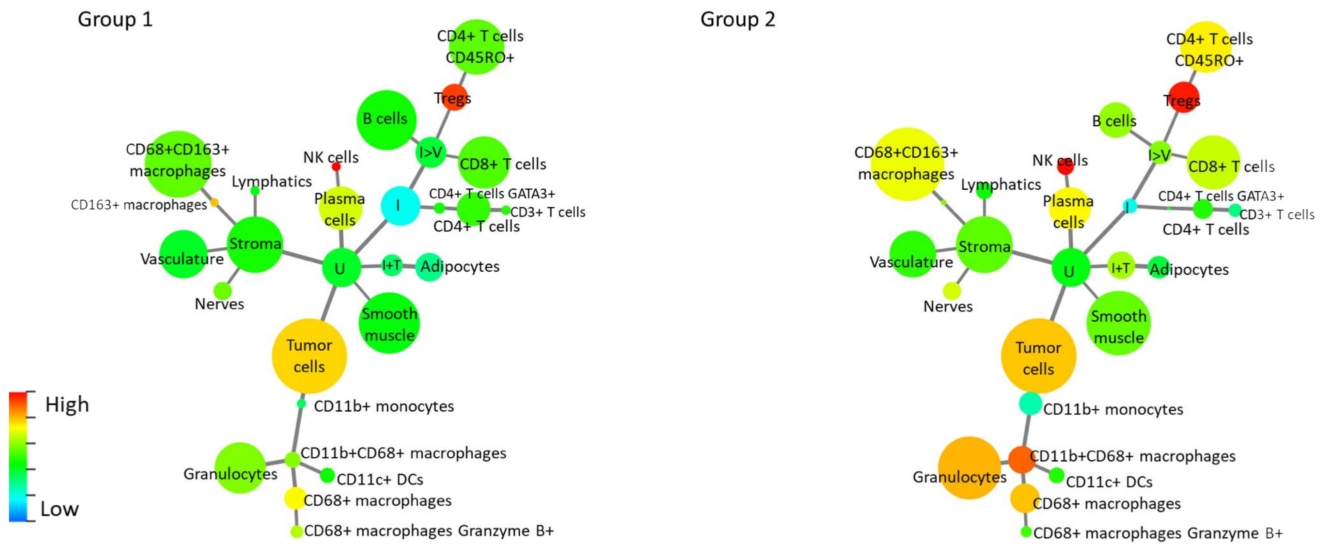

CD30

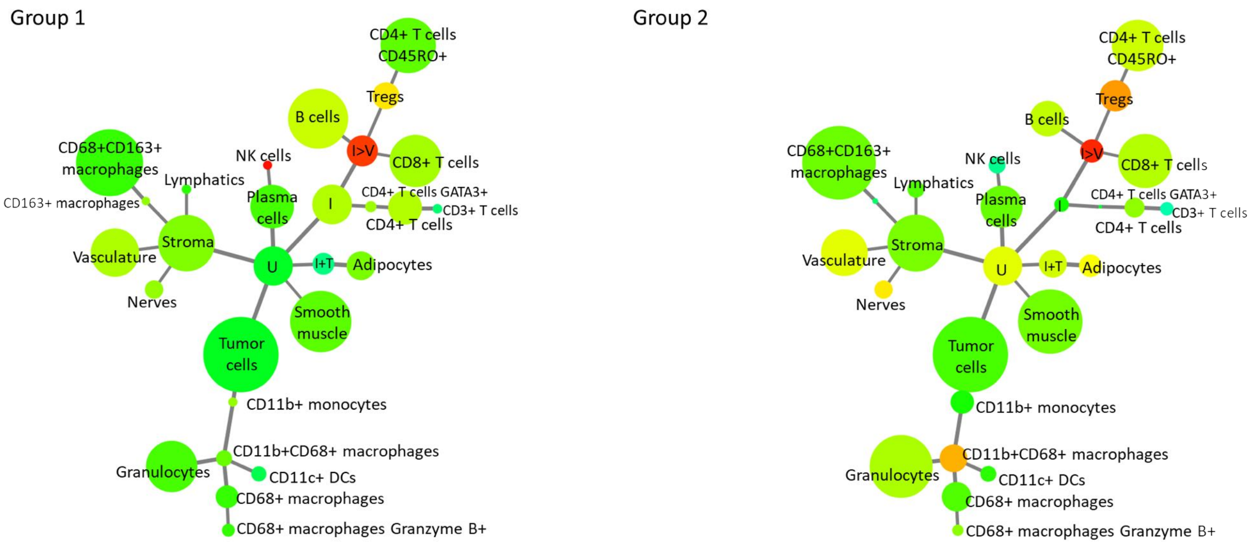

CD31

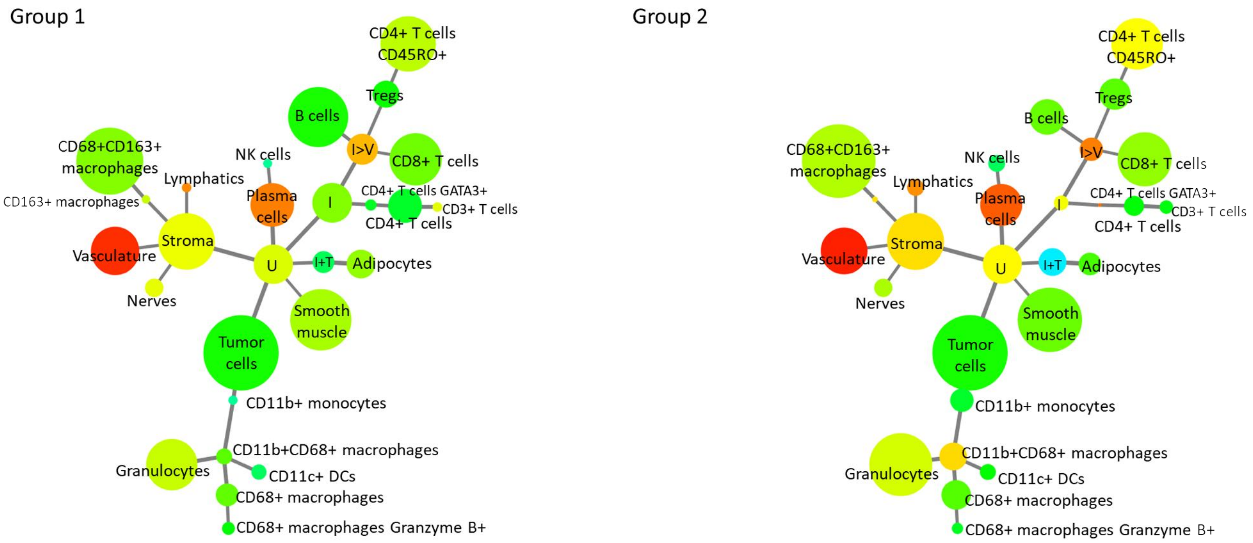

CD34

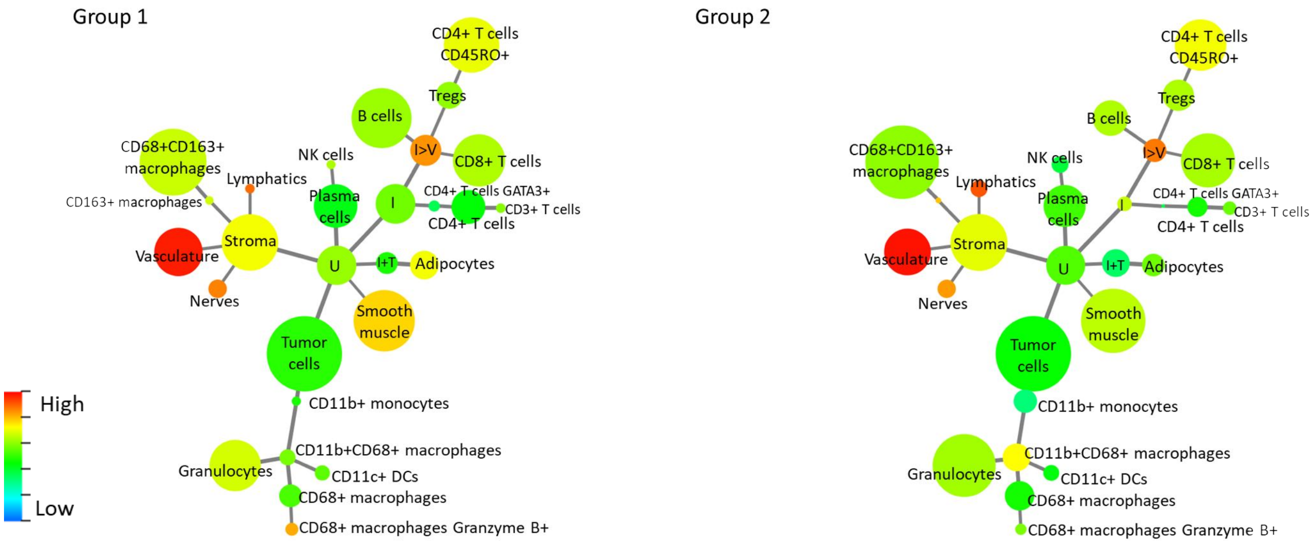

CD38

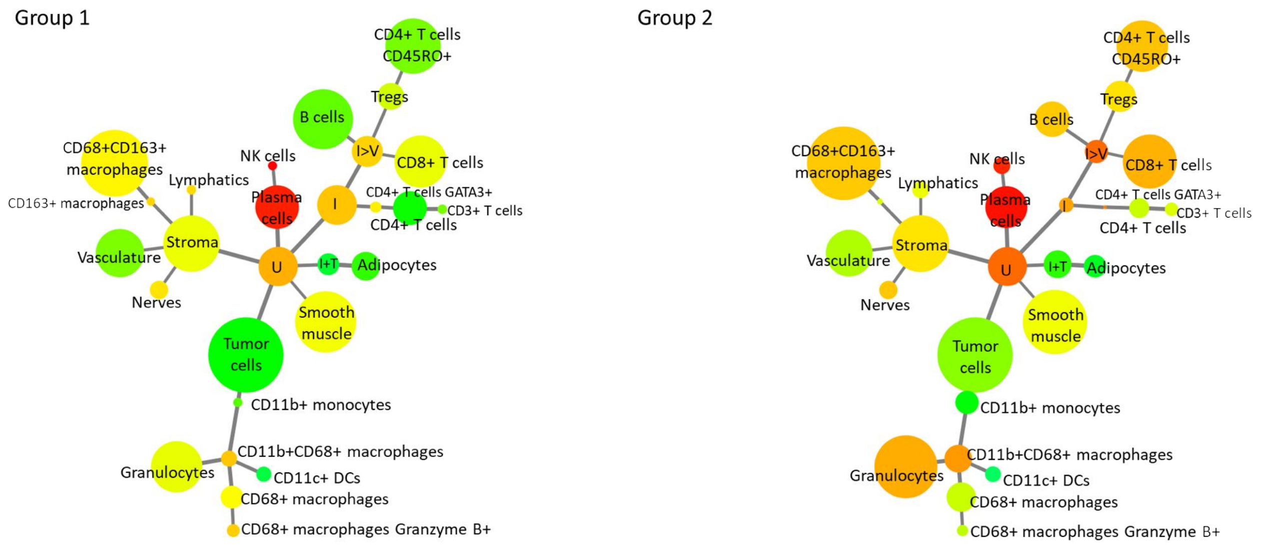

CD44

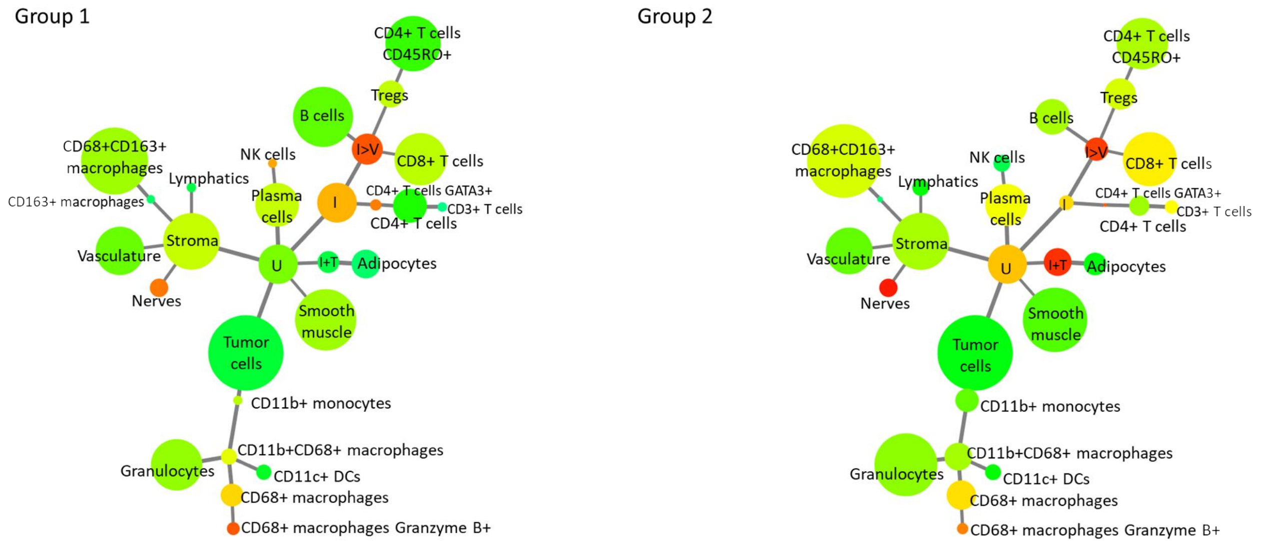

CD45

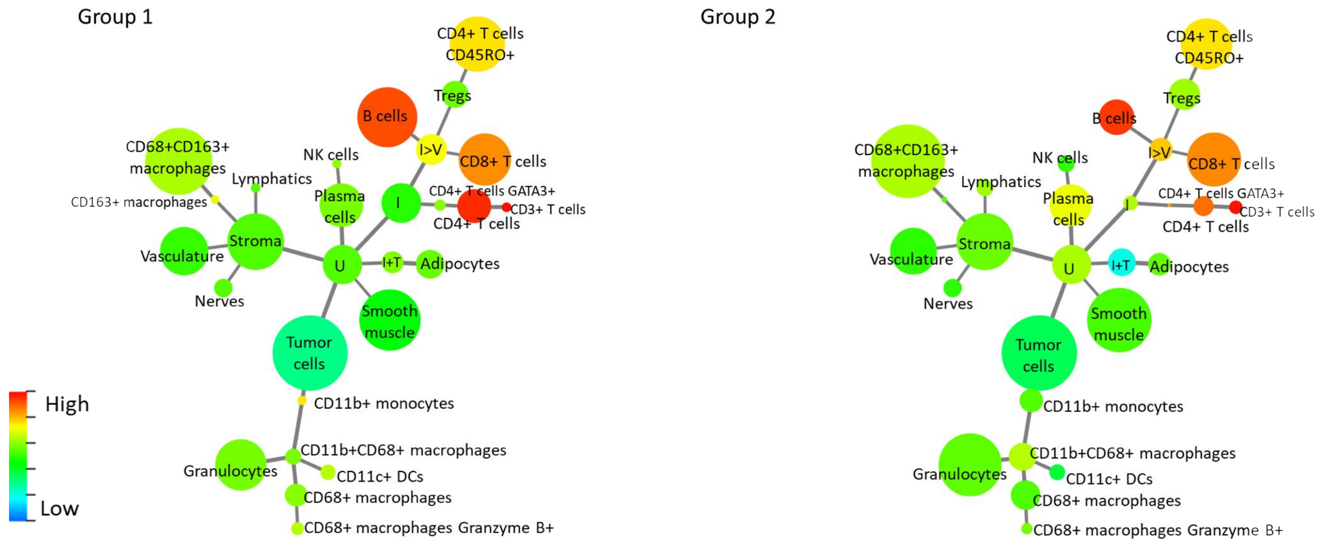

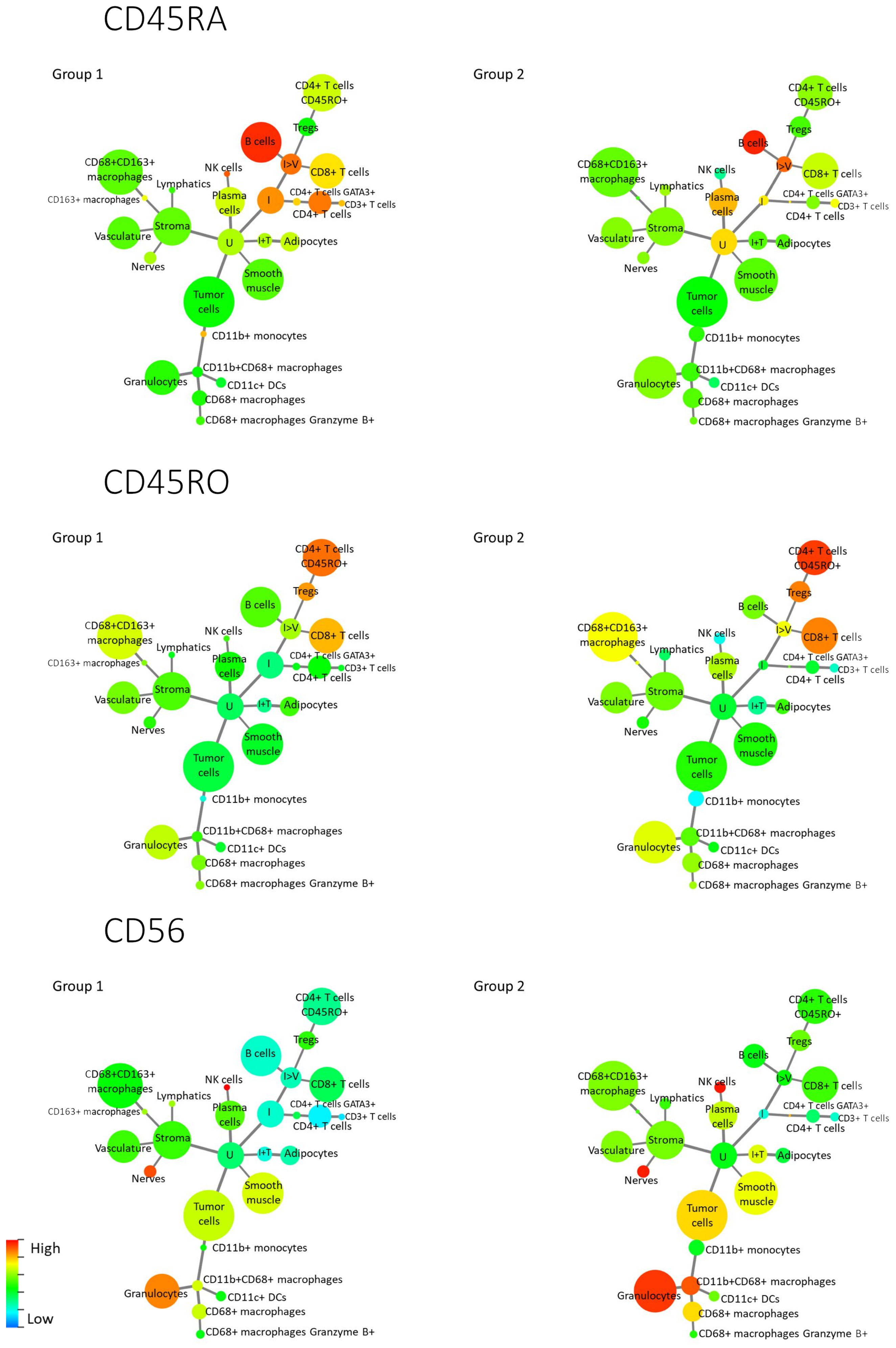
CD45RA
Group 1
CD4+ T cells CD45RO+
Tregs
B cells
I>V
CD8+ T cells
NK cells
CD68+CD163+ macrophages
Lymphatics
Plasma cells
I
CD4+ T cells GATA3+
CD3+ T cells
CD4+ T cells
CD163+ macrophages
Stroma
U
I+T
Adipocytes
Vasculature
Nerves
Smooth muscle
Tumor cells
CD11b+ monocytes
CD11b+CD68+ macrophages
Granulocytes
CD11c+ DCs
CD68+ macrophages
CD68+ macrophages Granzyme B+
Group 2
CD4+ T cells CD45RO+
Tregs
B cells
I>V
CD8+ T cells
NK cells
CD68+CD163+ macrophages
Lymphatics
Plasma cells
I
CD4+ T cells GATA3+
CD3+ T cells
CD4+ T cells
Stroma
U
I+T
Adipocytes
Vasculature
Nerves
Smooth muscle
Tumor cells
CD11b+ monocytes
CD11b+CD68+ macrophages
Granulocytes
CD11c+ DCs
CD68+ macrophages
CD68+ macrophages Granzyme B+
CD45RO
Group 1
CD4+ T cells CD45RO+
Tregs
B cells
I>V
CD8+ T cells
NK cells
CD68+CD163+ macrophages
Lymphatics
Plasma cells
I
CD4+ T cells GATA3+
CD3+ T cells
CD4+ T cells
CD163+ macrophages
Stroma
U
I+T
Adipocytes
Vasculature
Nerves
Smooth muscle
Tumor cells
CD11b+ monocytes
CD11b+CD68+ macrophages
Granulocytes
CD11c+ DCs
CD68+ macrophages
CD68+ macrophages Granzyme B+
Group 2
CD4+ T cells CD45RO+
Tregs
B cells
I>V
CD8+ T cells
NK cells
CD68+CD163+ macrophages
Lymphatics
Plasma cells
I
CD4+ T cells GATA3+
CD3+ T cells
CD4+ T cells
Stroma
U
I+T
Adipocytes
Vasculature
Nerves
Smooth muscle
Tumor cells
CD11b+ monocytes
CD11b+CD68+ macrophages
Granulocytes
CD11c+ DCs
CD68+ macrophages
CD68+ macrophages Granzyme B+
CD56
Group 1
CD4+ T cells CD45RO+
Tregs
B cells
I>V
CD8+ T cells
NK cells
CD68+CD163+ macrophages
Lymphatics
Plasma cells
I
CD4+ T cells GATA3+
CD3+ T cells
CD4+ T cells
CD163+ macrophages
Stroma
U
I+T
Adipocytes
Vasculature
Nerves
Smooth muscle
Tumor cells
CD11b+ monocytes
CD11b+CD68+ macrophages
Granulocytes
CD11c+ DCs
CD68+ macrophages
CD68+ macrophages Granzyme B+
Group 2
CD4+ T cells CD45RO+
Tregs
B cells
I>V
CD8+ T cells
NK cells
CD68+CD163+ macrophages
Lymphatics
Plasma cells
I
CD4+ T cells GATA3+
CD3+ T cells
CD4+ T cells
Stroma
U
I+T
Adipocytes
Vasculature
Nerves
Smooth muscle
Tumor cells
CD11b+ monocytes
CD11b+CD68+ macrophages
Granulocytes
CD11c+ DCs
CD68+ macrophages
CD68+ macrophages Granzyme B+
High
Low

CD57

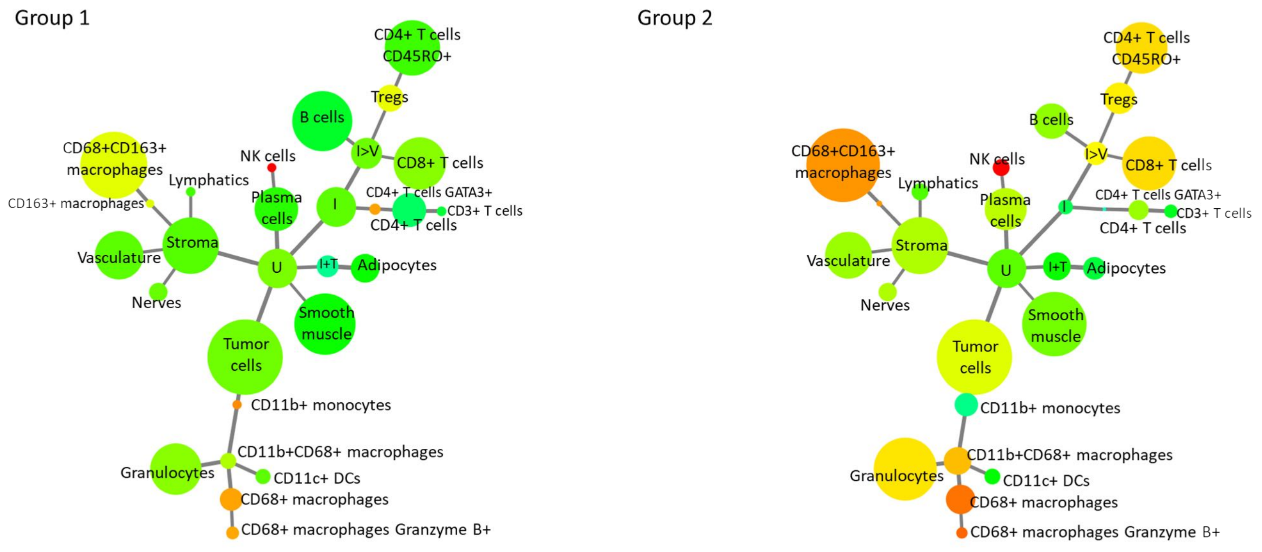

CD68

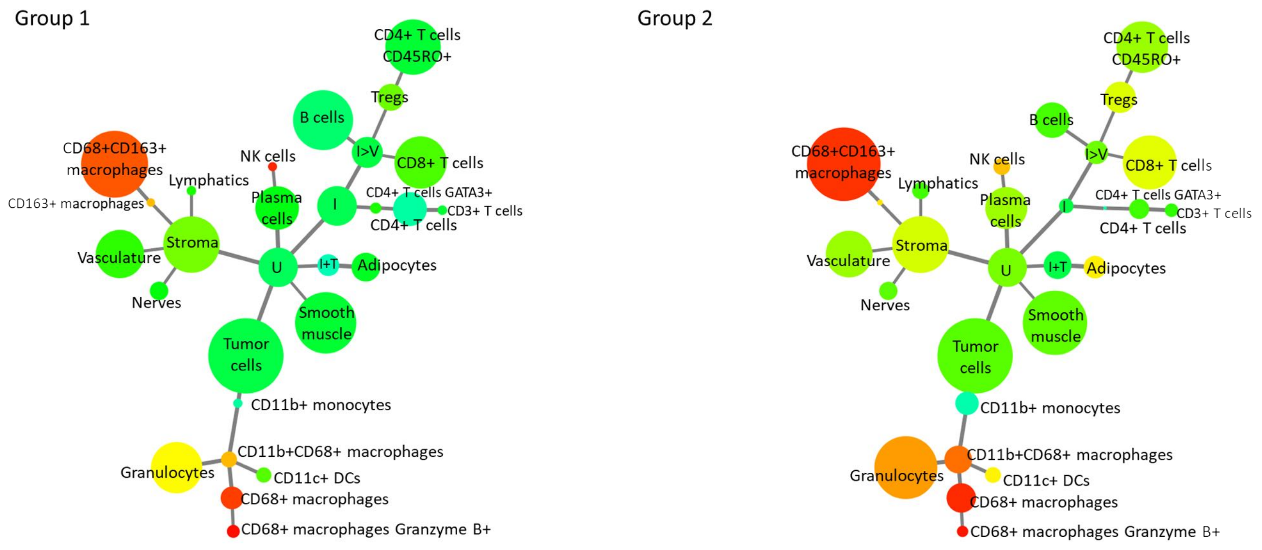

CD71

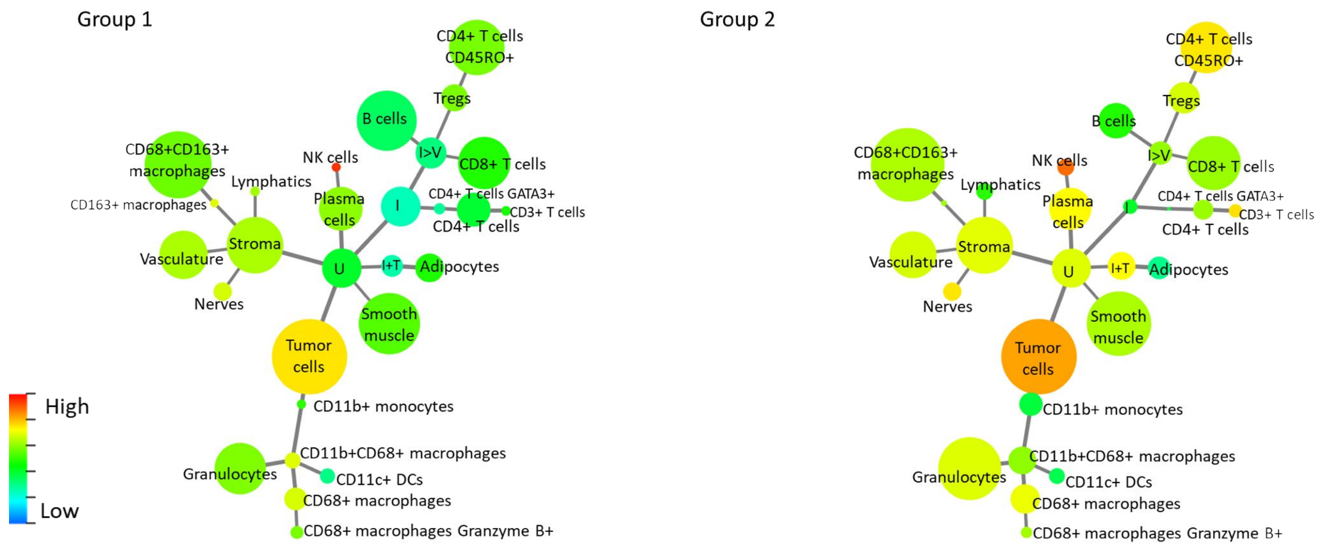

CD138

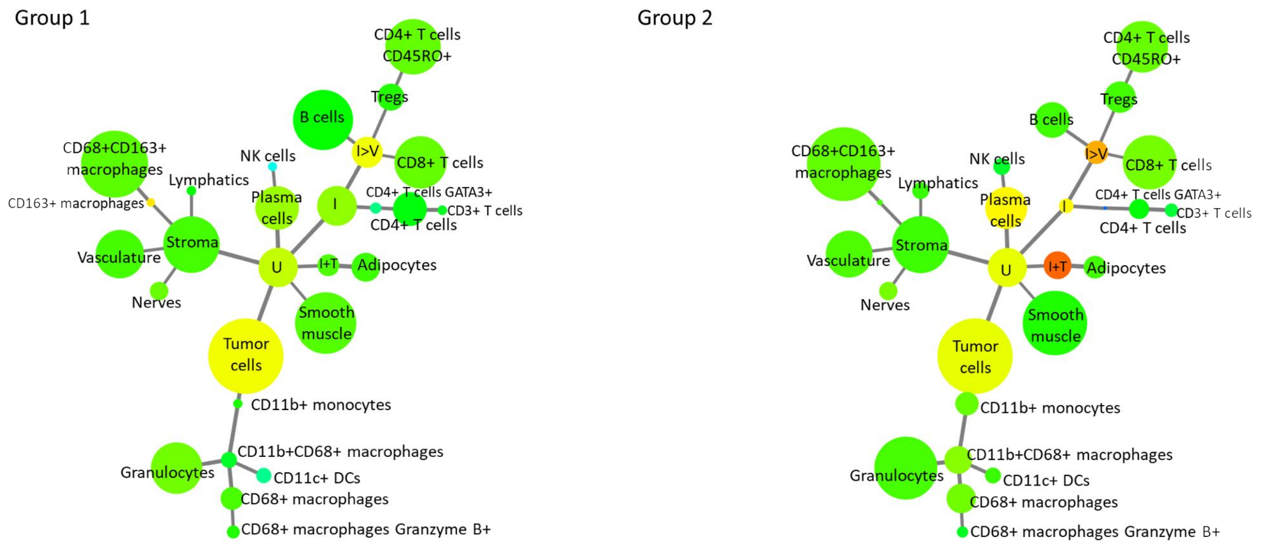

CD163

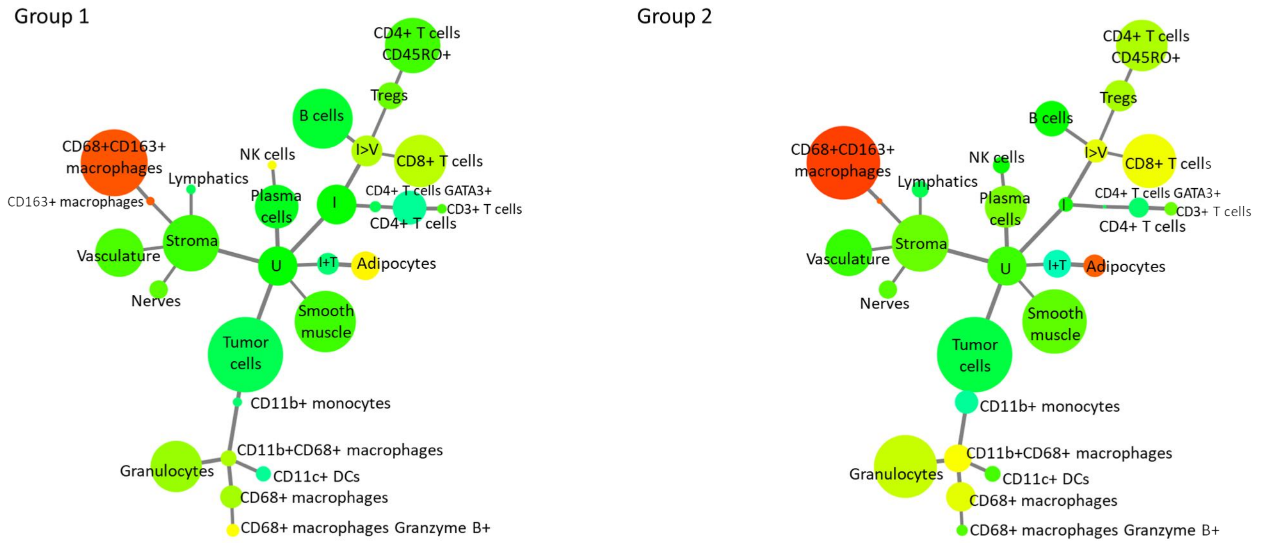

CD194

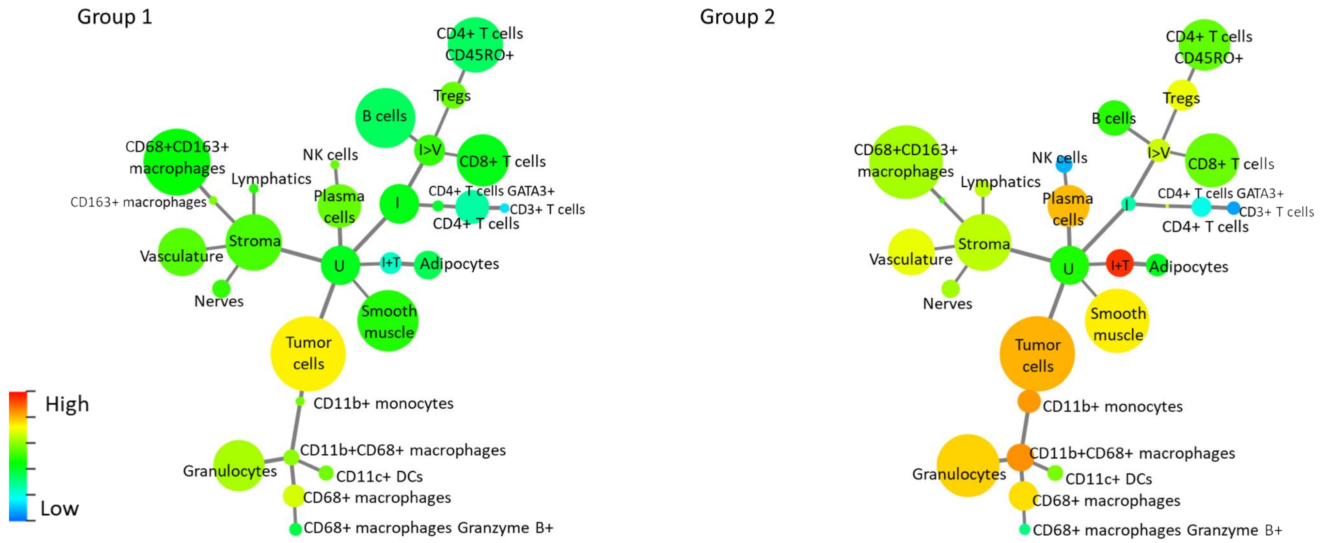

BCL-2

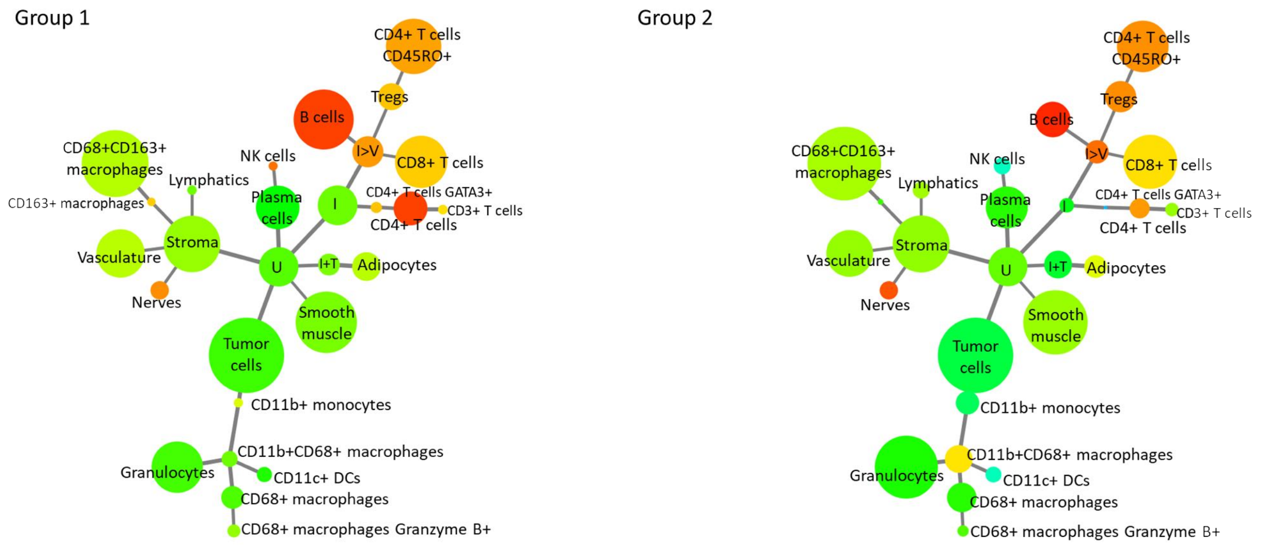

Beta-catenin

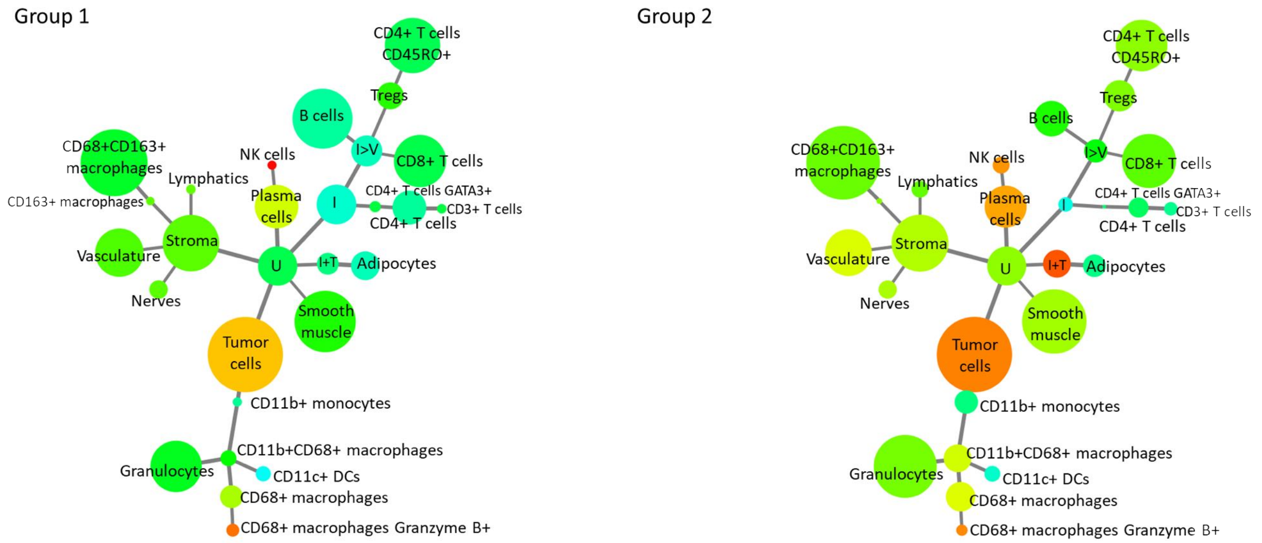

CDX2

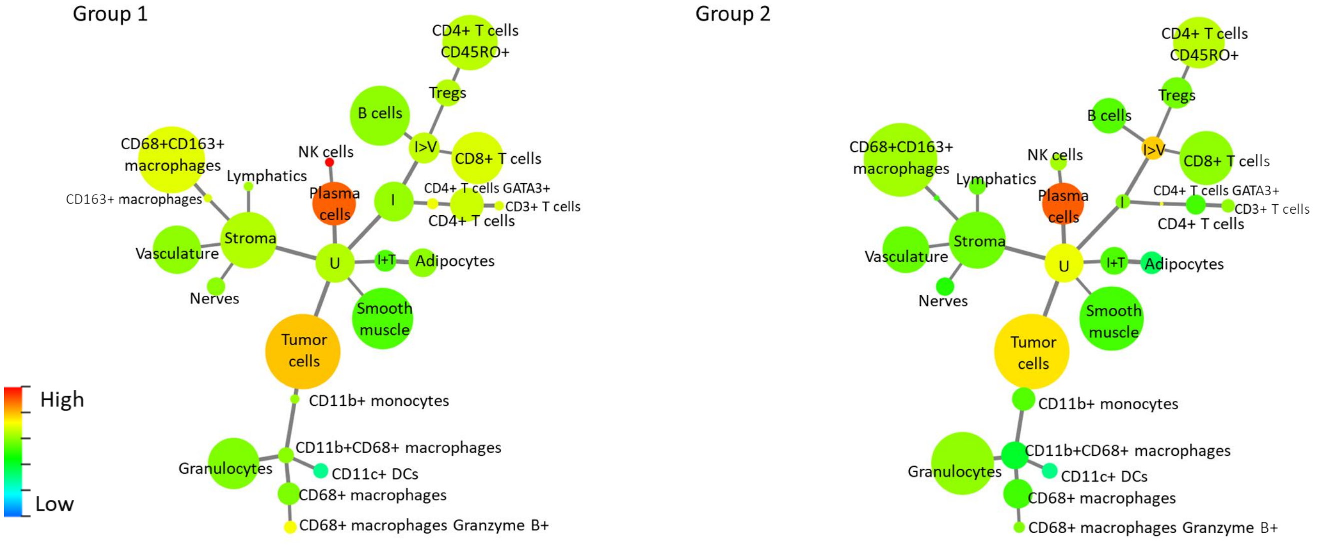

Chromogranin A

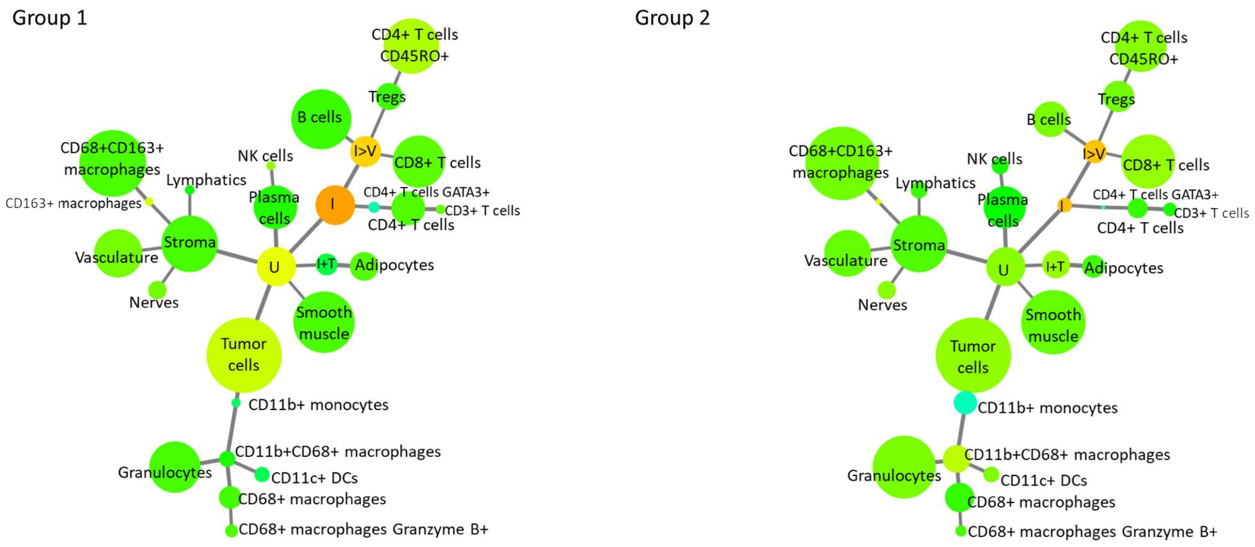

Collagen IV

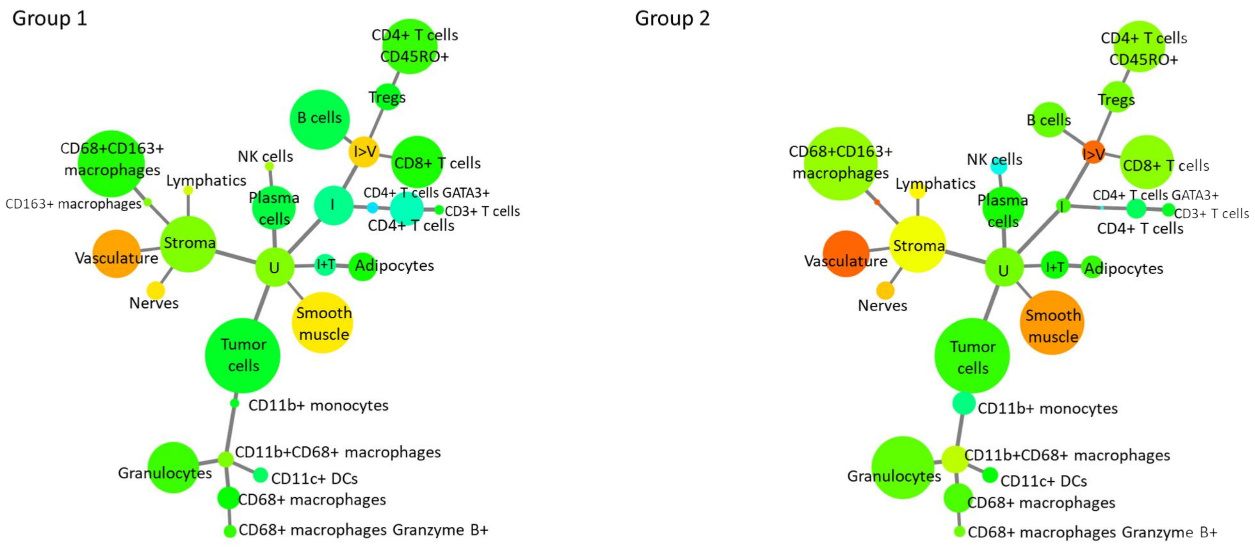

Cytokeratin

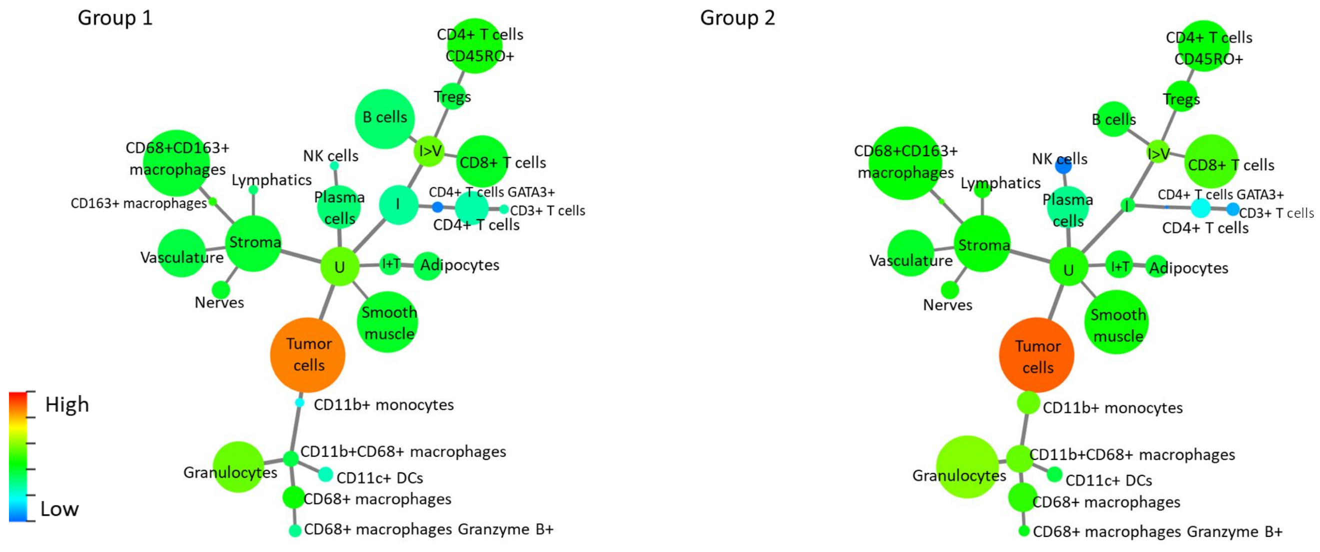

EGFR

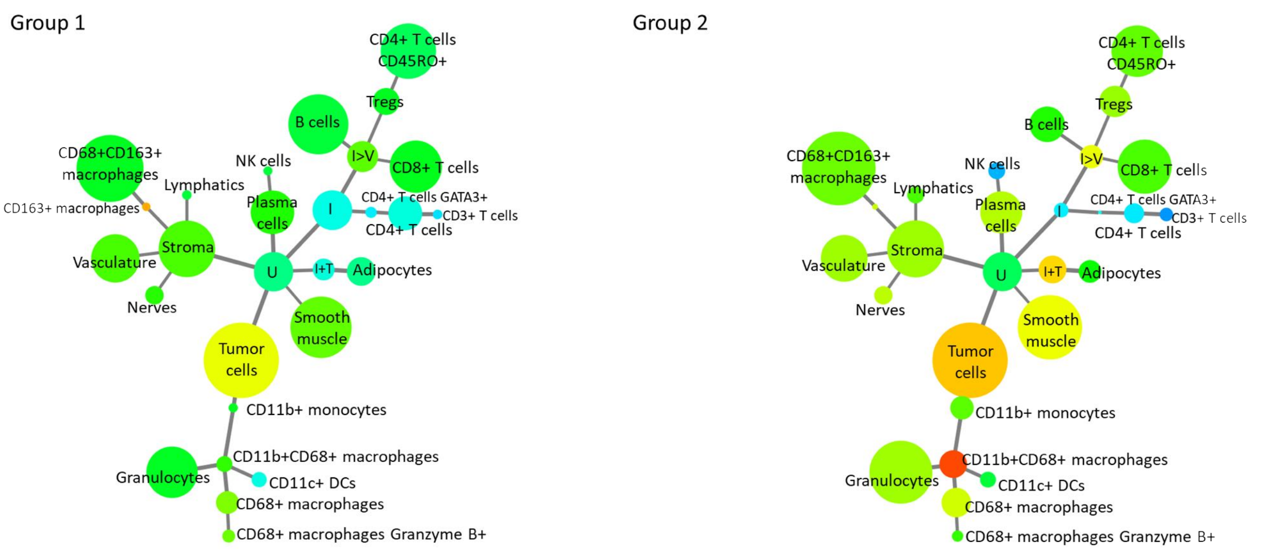

FoxP3

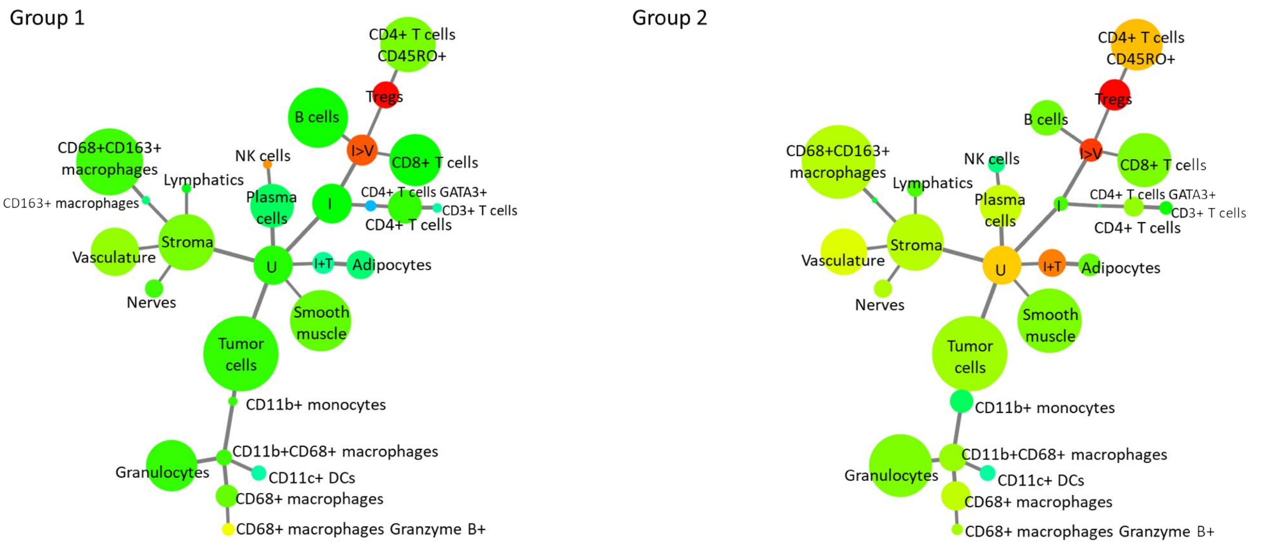

GATA3

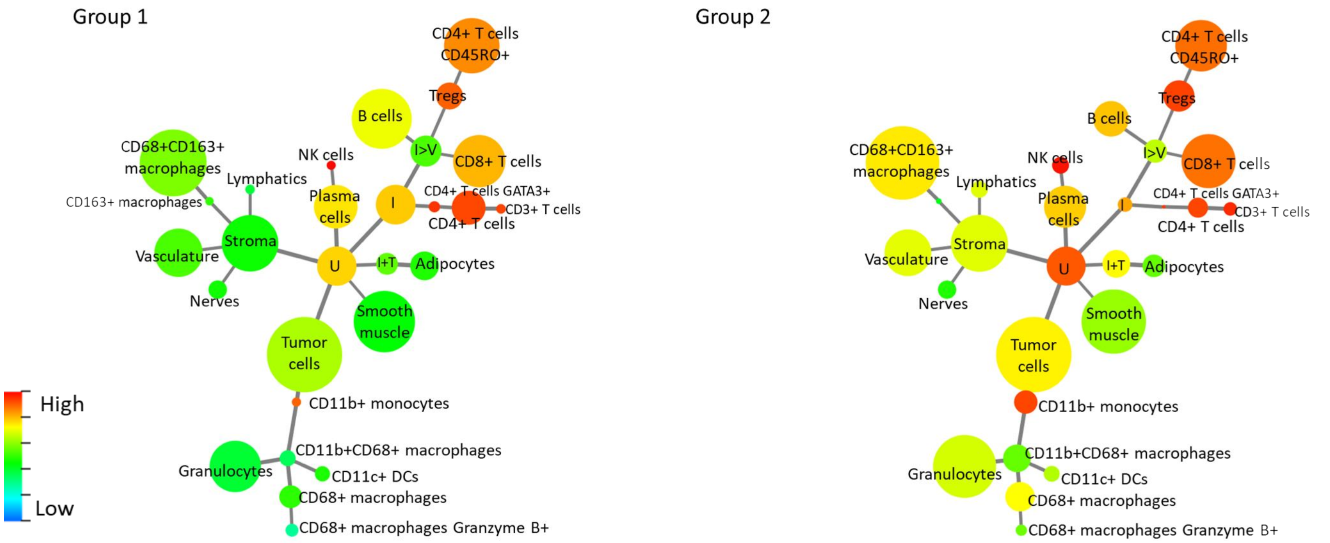

GFAP

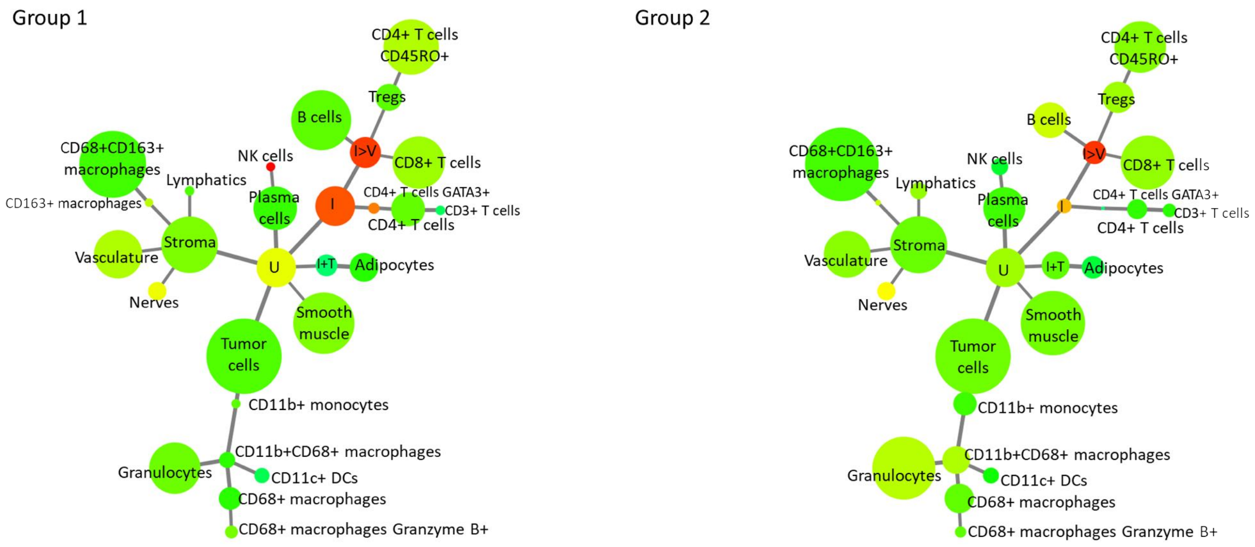

Granzyme B

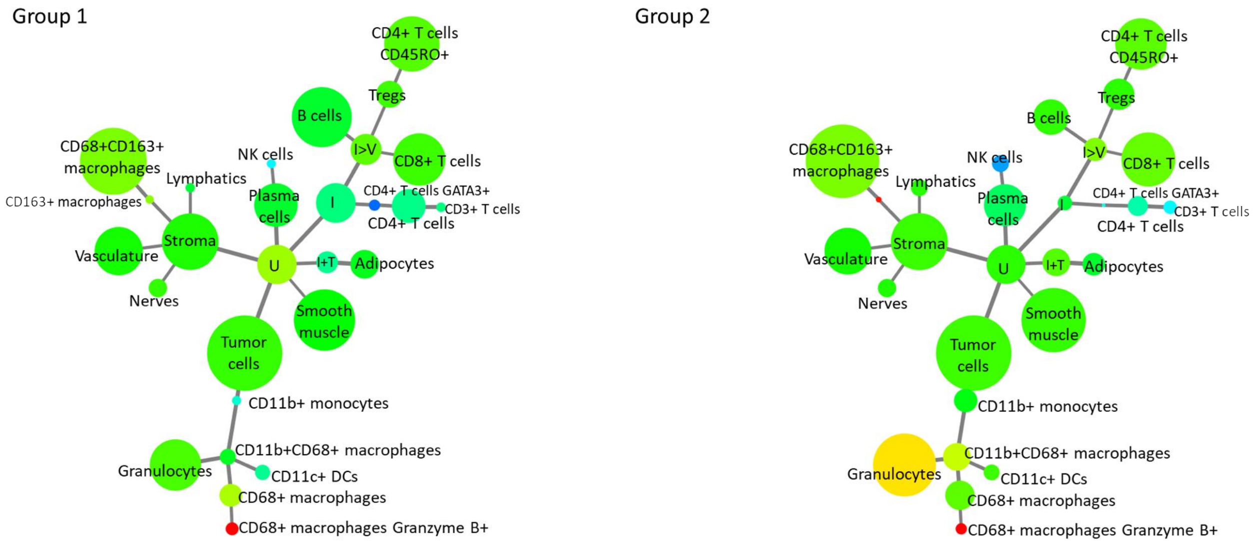

HLA-DR

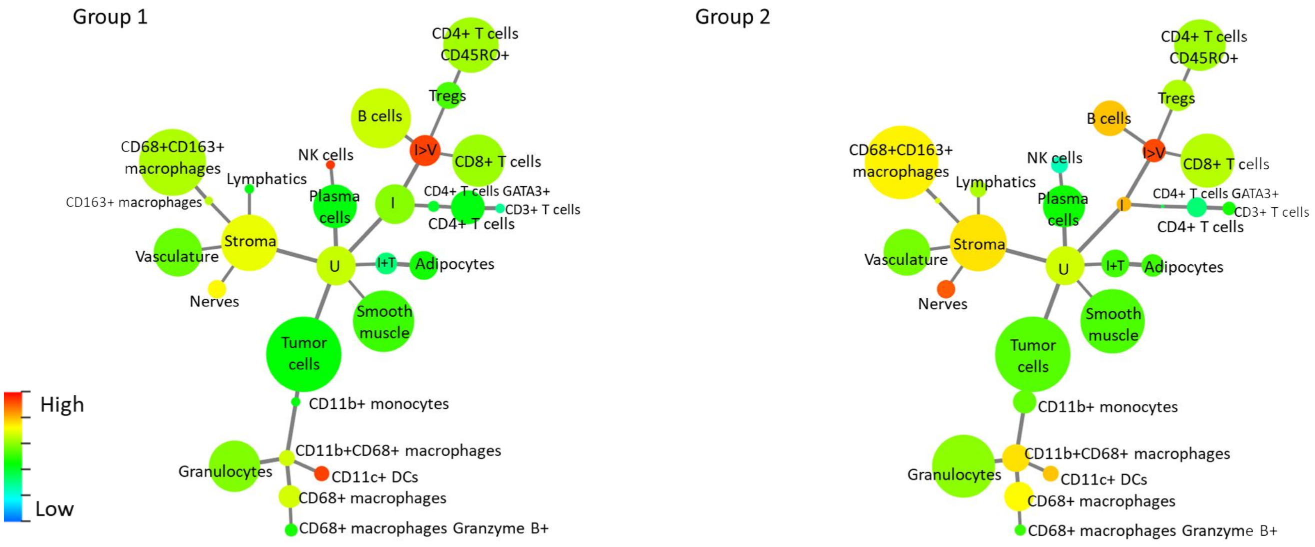

ICOS

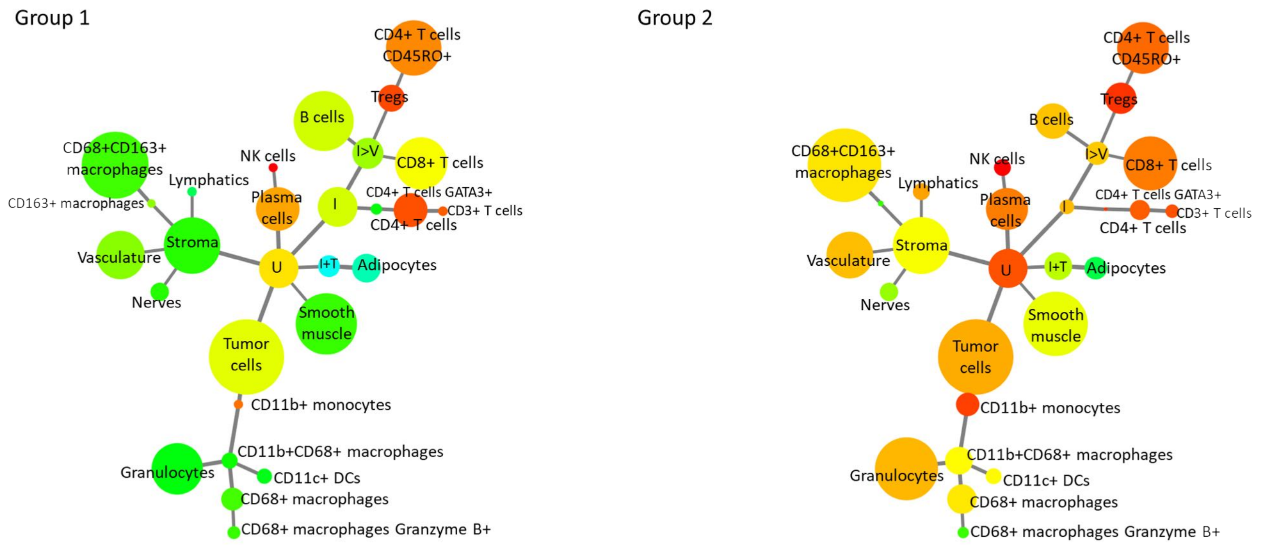

IDO-1

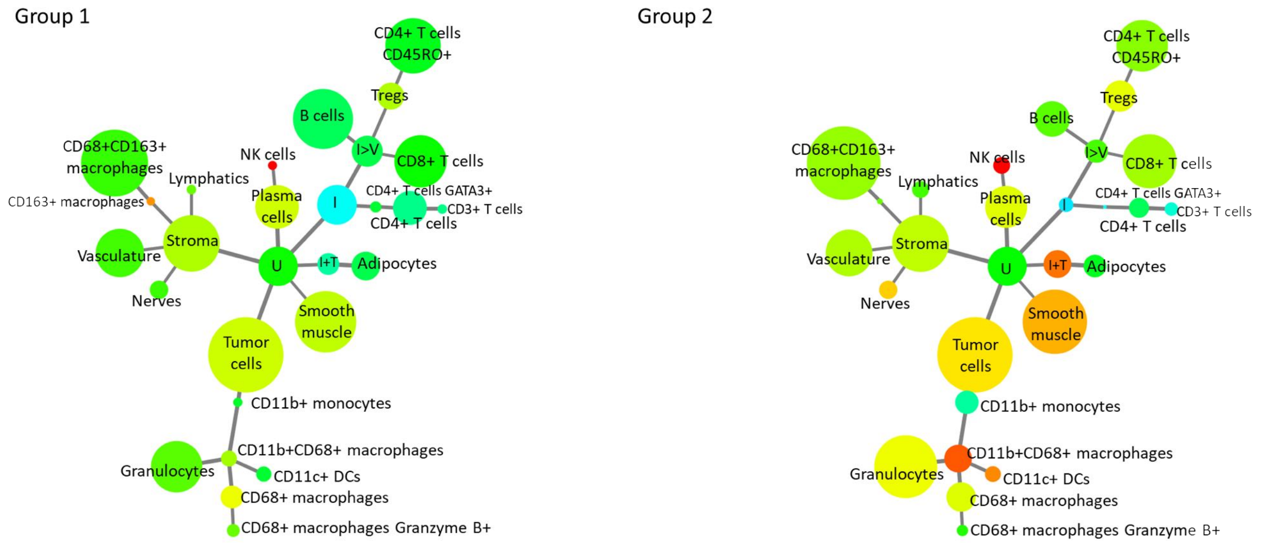

Ki-67

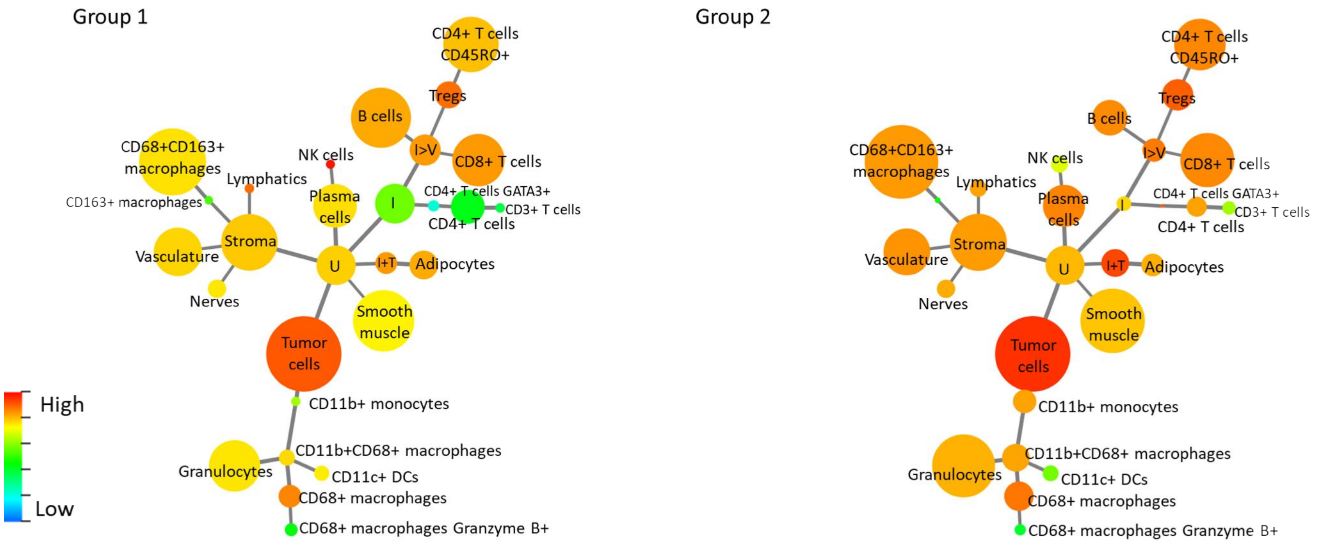

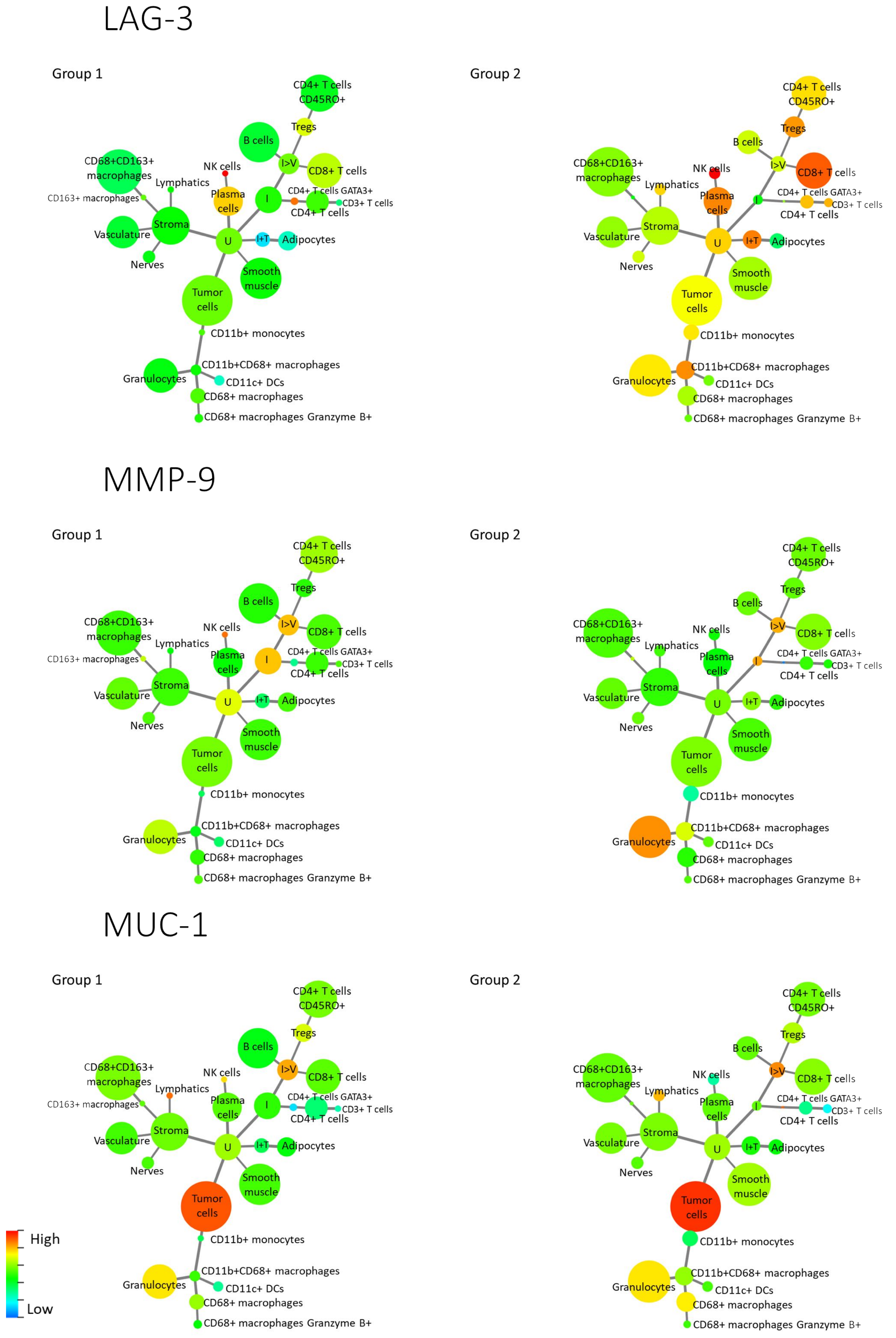
LAG-3
Group 1
Group 2
MMP-9
Group 1
Group 2
MUC-1
Group 1
Group 2
CD4+ T cells CD45RO+
Tregs
B cells
I>V
CD8+ T cells
NK cells
CD68+CD163+ macrophages
Lymphatics
CD163+ macrophages
Plasma cells
I
CD4+ T cells GATA3+
CD3+ T cells
CD4+ T cells
Vasculature
Stroma
U
I+T
Adipocytes
Nerves
Smooth muscle
Tumor cells
CD11b+ monocytes
CD11b+CD68+ macrophages
Granulocytes
CD11c+ DCs
CD68+ macrophages
CD68+ macrophages Granzyme B+
High
Low

Na-K-ATPase

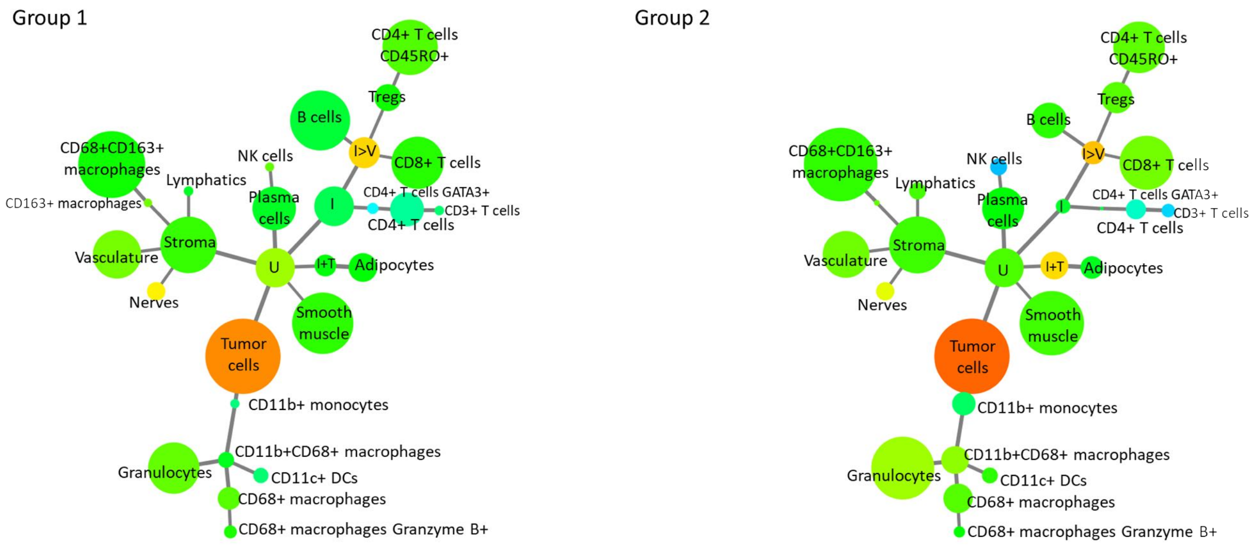

p53

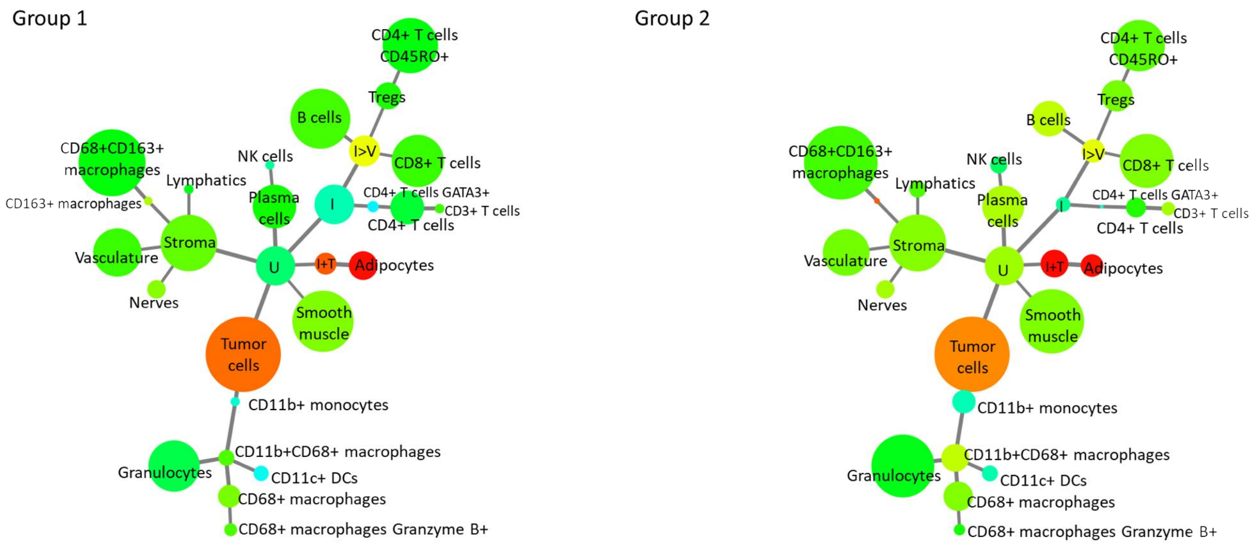

PD-1

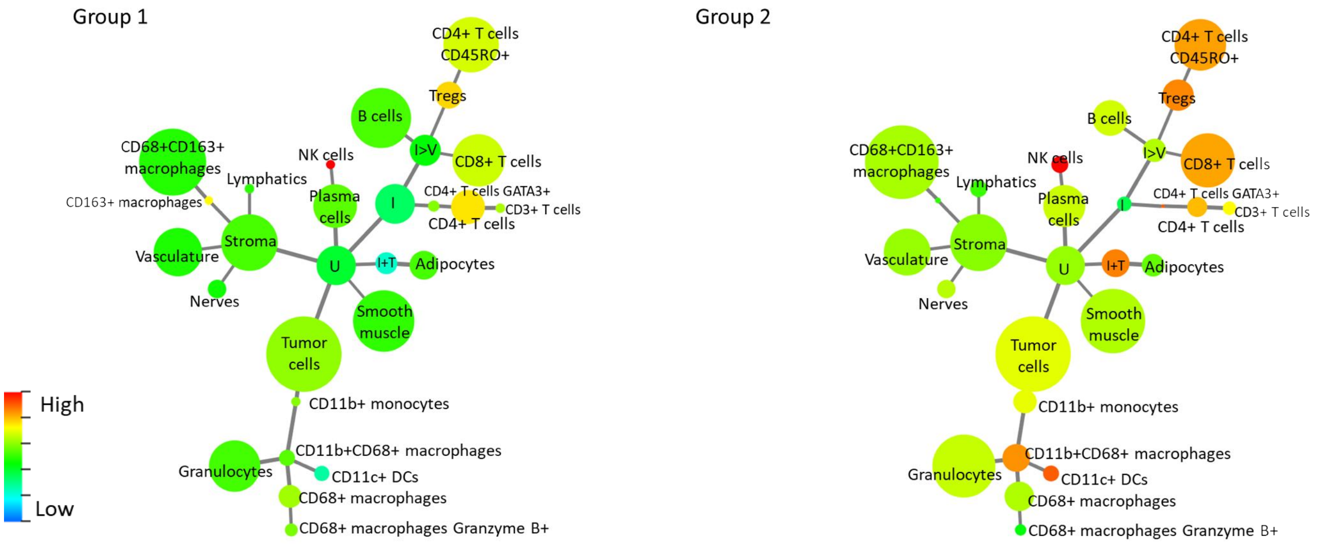

PD-L1

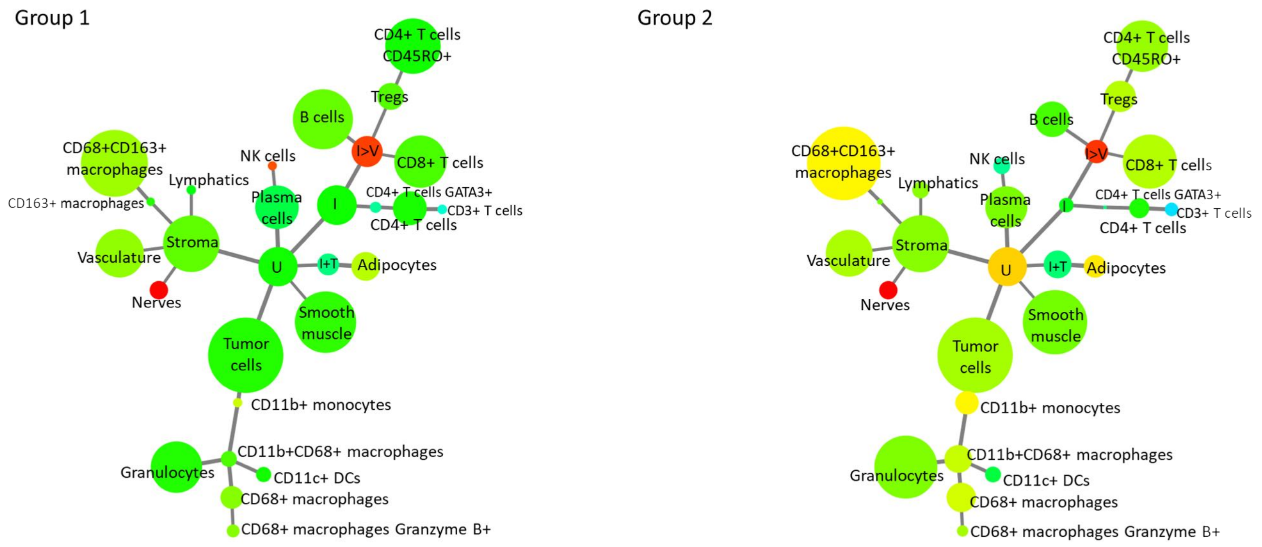

Podoplanin

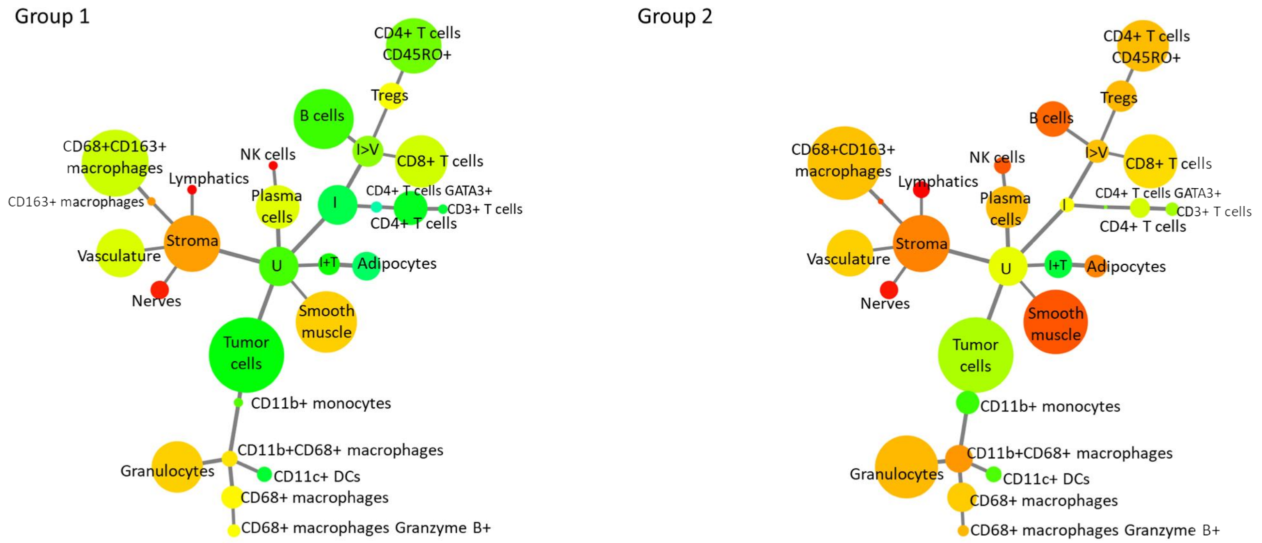

Smooth Muscle Actin (SMA)

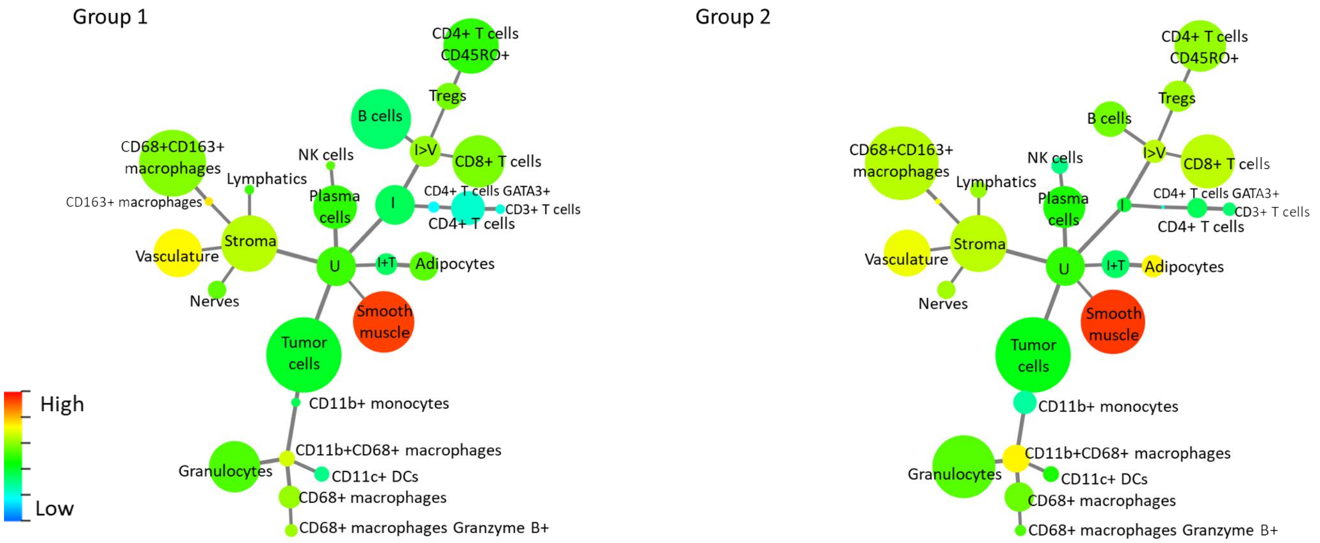

Synaptophysin

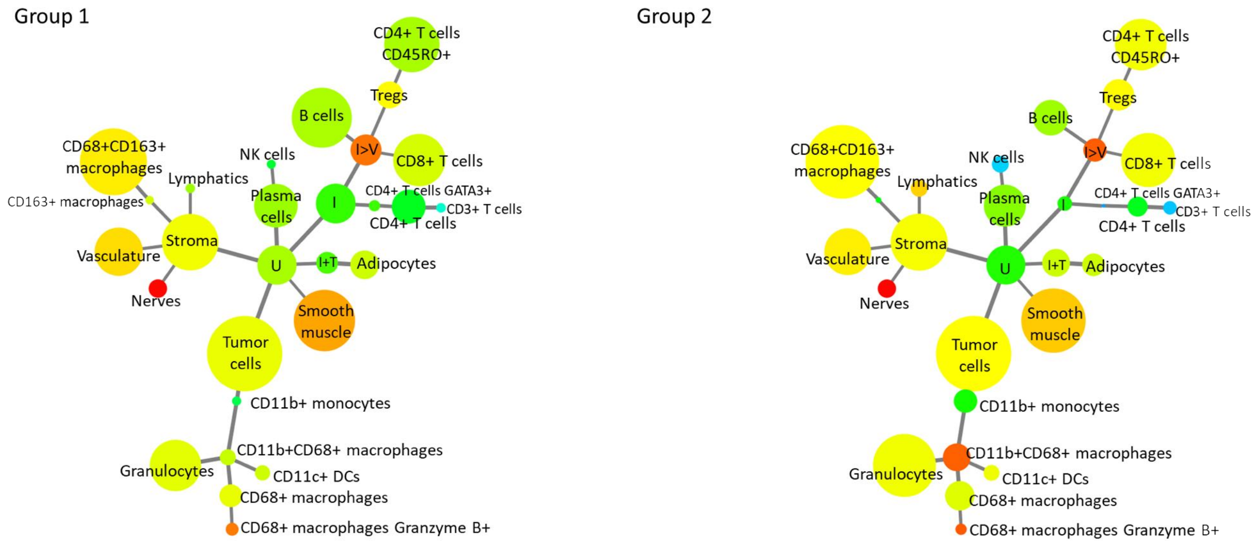

T-bet

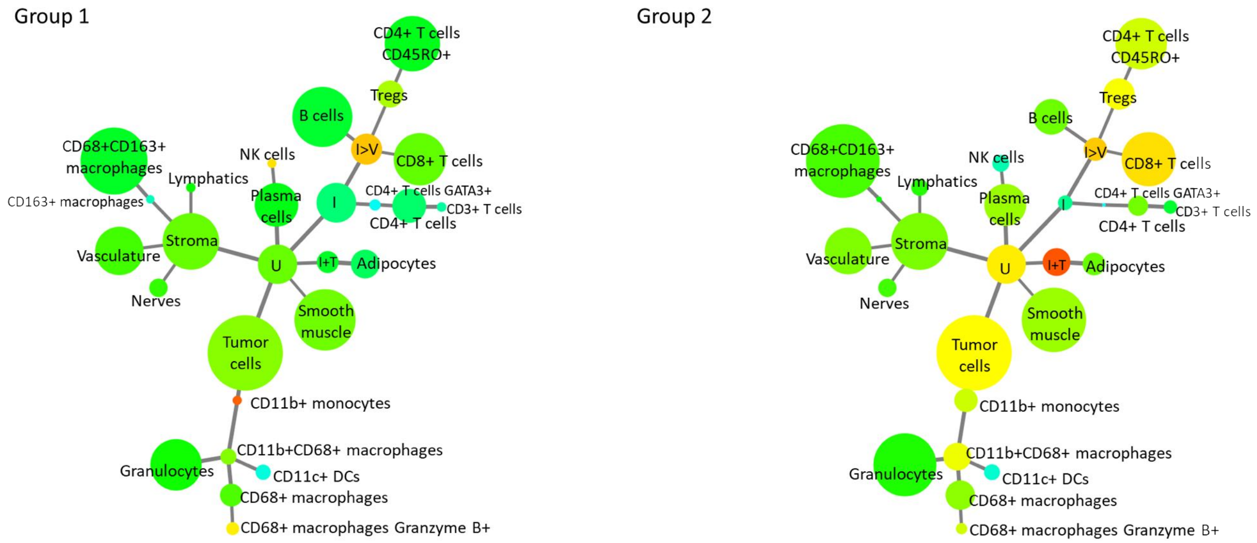

Vimentin

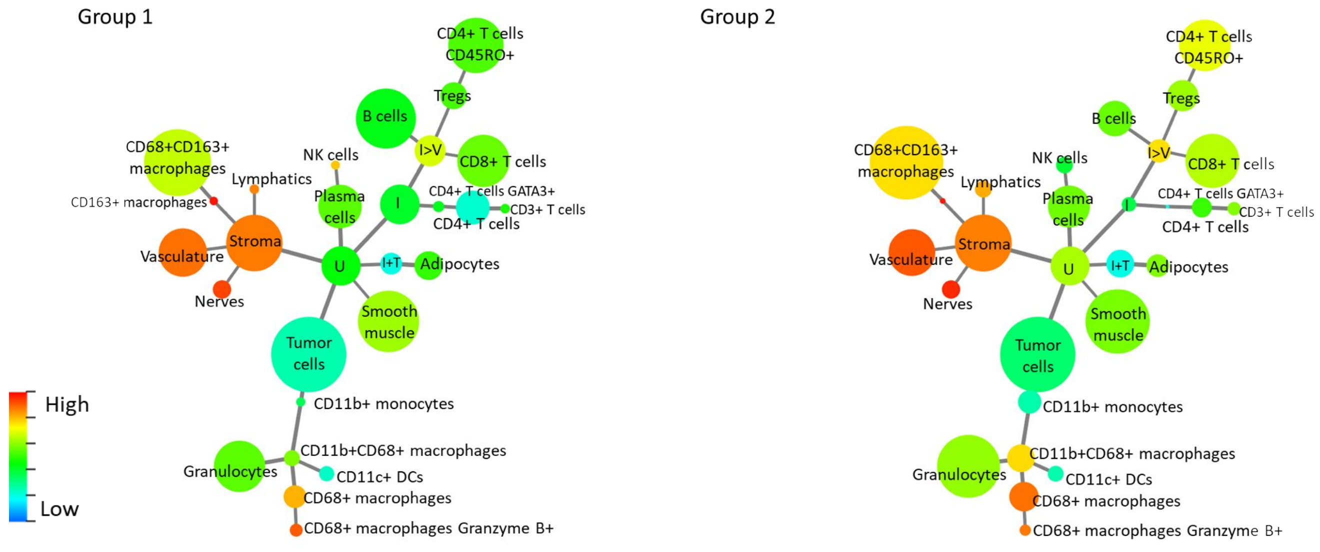

VISTA

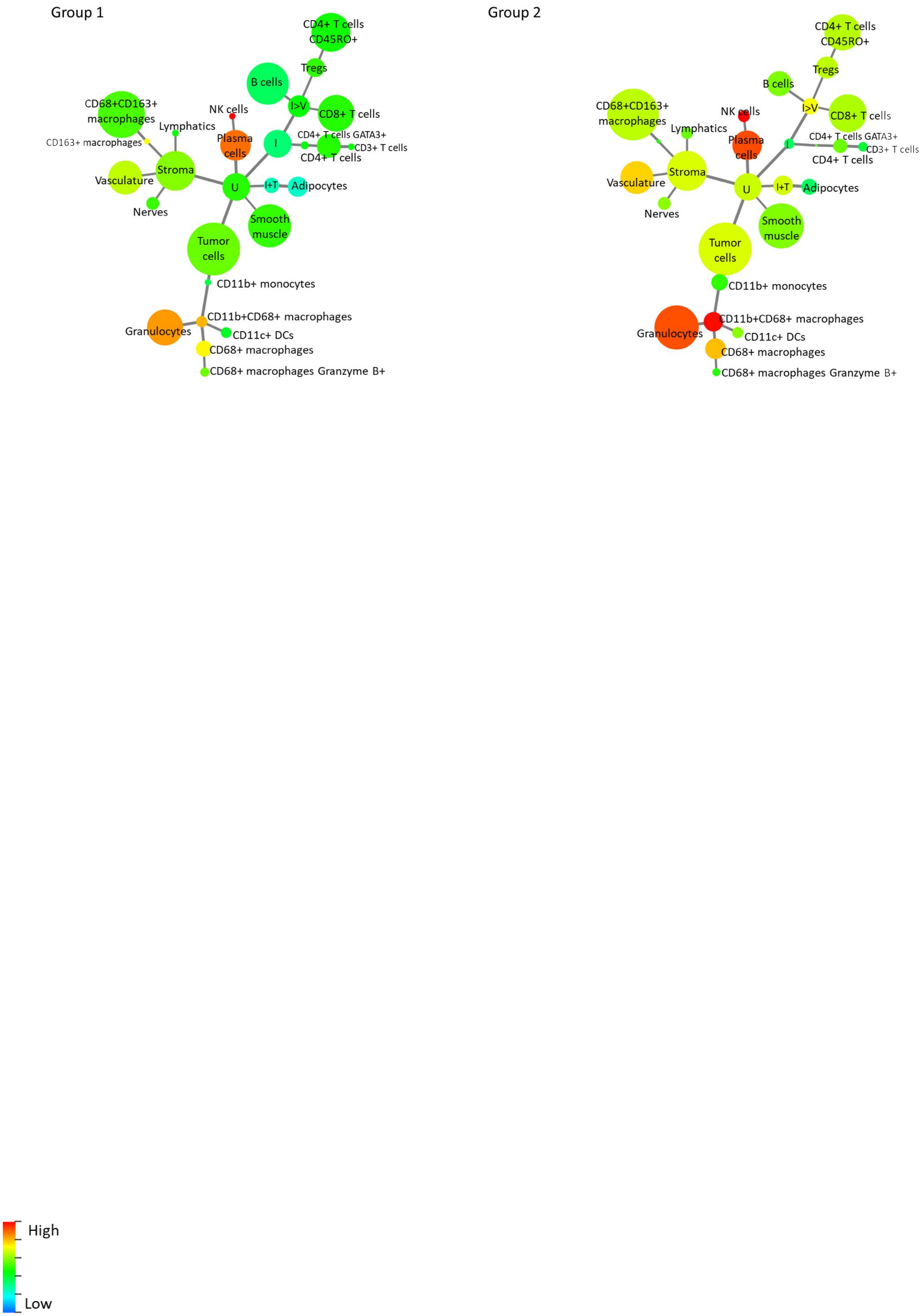

Group 1
CD4+ T cells CD45RO+
Tregs
B cells
I>V
CD8+ T cells
CD68+CD163+ macrophages
NK cells
Lymphatics
Plasma cells
I
CD4+ T cells GATA3+
CD3+ T cells
CD4+ T cells
CD163+ macrophages
Stroma
Vasculature
U
I+T
Adipocytes
Nerves
Smooth muscle
Tumor cells
CD11b+ monocytes
CD11b+CD68+ macrophages
Granulocytes
CD11c+ DCs
CD68+ macrophages
CD68+ macrophages Granzyme B+
Group 2
CD4+ T cells CD45RO+
Tregs
B cells
I>V
CD8+ T cells
CD68+CD163+ macrophages
NK cells
Lymphatics
Plasma cells
I
CD4+ T cells GATA3+
CD3+ T cells
CD4+ T cells
Stroma
Vasculature
U
I+T
Adipocytes
Nerves
Smooth muscle
Tumor cells
CD11b+ monocytes
CD11b+CD68+ macrophages
Granulocytes
CD11c+ DCs
CD68+ macrophages
CD68+ macrophages Granzyme B+
High
Low

Data S4. Supervised Annotation of CRC Clusters Based on Marker Expression, Tissue Localization and Morphology, Related to Figure 3 and STAR Methods. After X-shift clustering, single cells from the 143 resulting initial clusters were overlaid on the raw data fluorescent images and on H&E stains of TMAs based on X/Y positions and visually verified based on marker expression profiles, morphology, and localization within the tissue. Similar clusters were manually merged, resulting in 28 final clusters. Here, 18 representative clusters are shown as yellow crosses based on X/Y coordinates of the cells contained in that cluster, overlaid on stitched montages of different TMA cores. For each of these 18 clusters, three examples of markers important for cluster identification (2 positive and 1 negative) and DRAQ5 nuclear stain (right panels) are shown as well as a global overview of cellular distribution of that cluster within a single TMA spot (yellow crosses on black background, left panel).

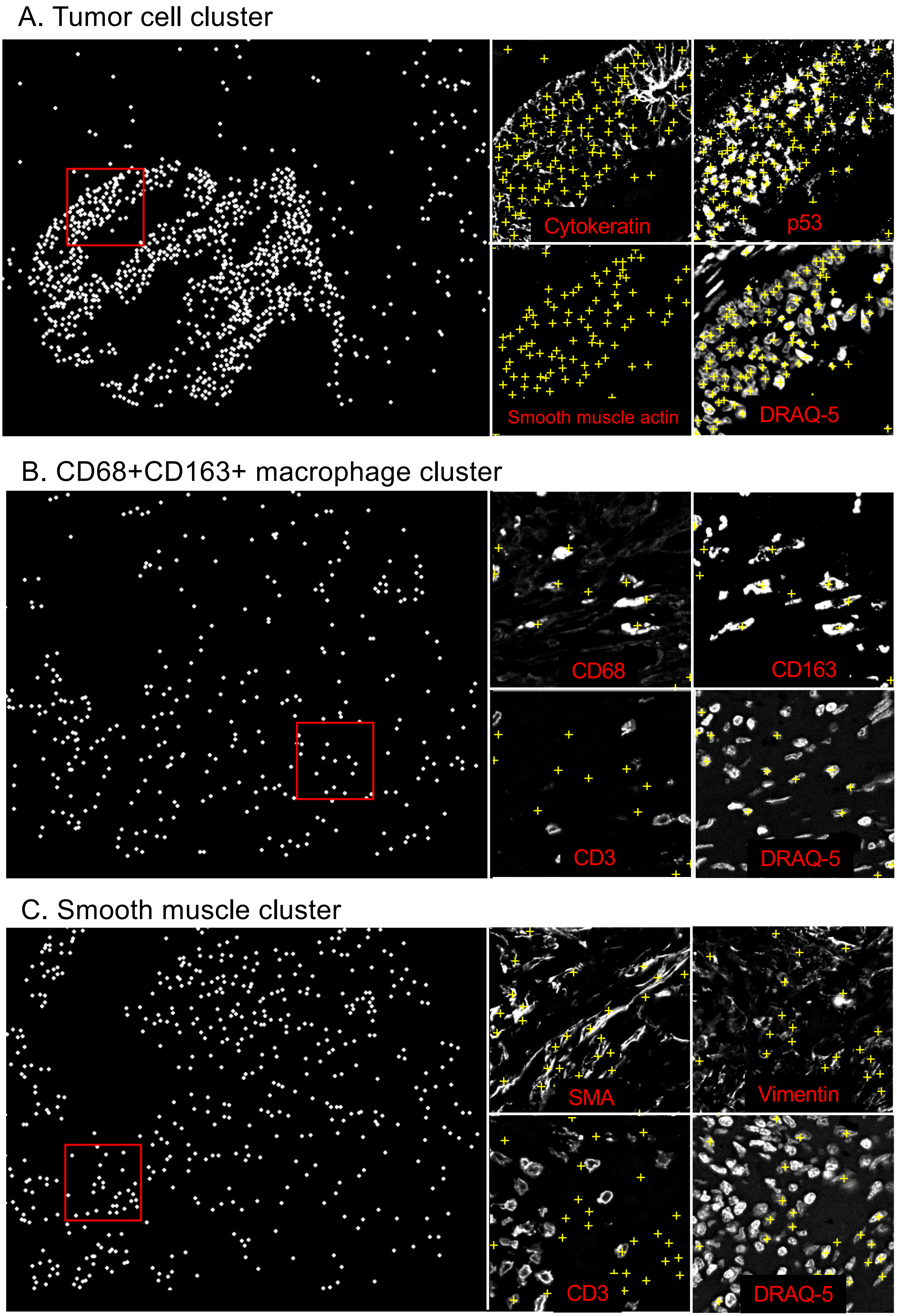
A. Tumor cell cluster
Cytokeratin
p53
Smooth muscle actin
DRAQ-5
B. CD68+CD163+ macrophage cluster
CD68
CD163
CD3
DRAQ-5
C. Smooth muscle cluster
SMA
Vimentin
CD3
DRAQ-5

D. Granulocyte cluster

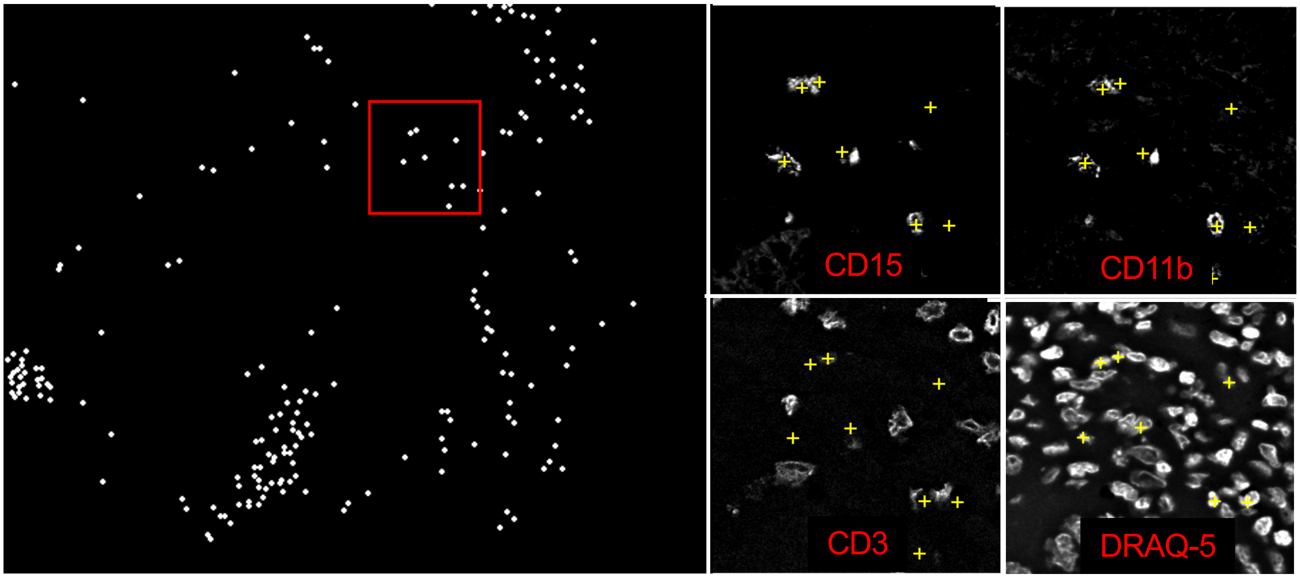

E. Stroma cluster

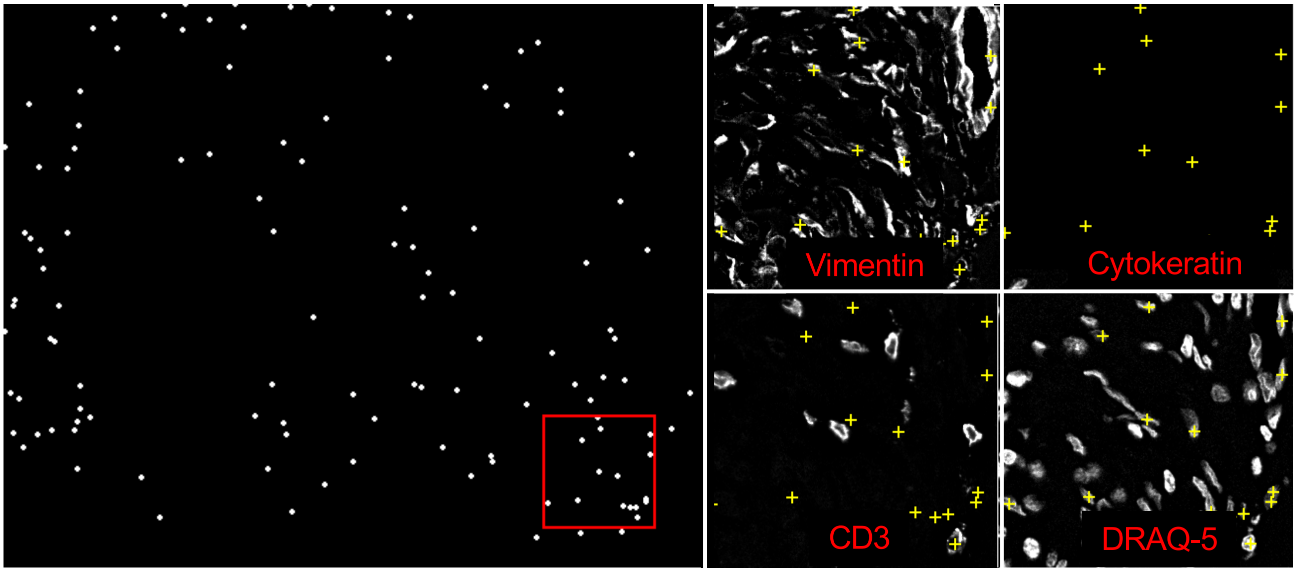

F. CD4+CD45RO+ T cell cluster

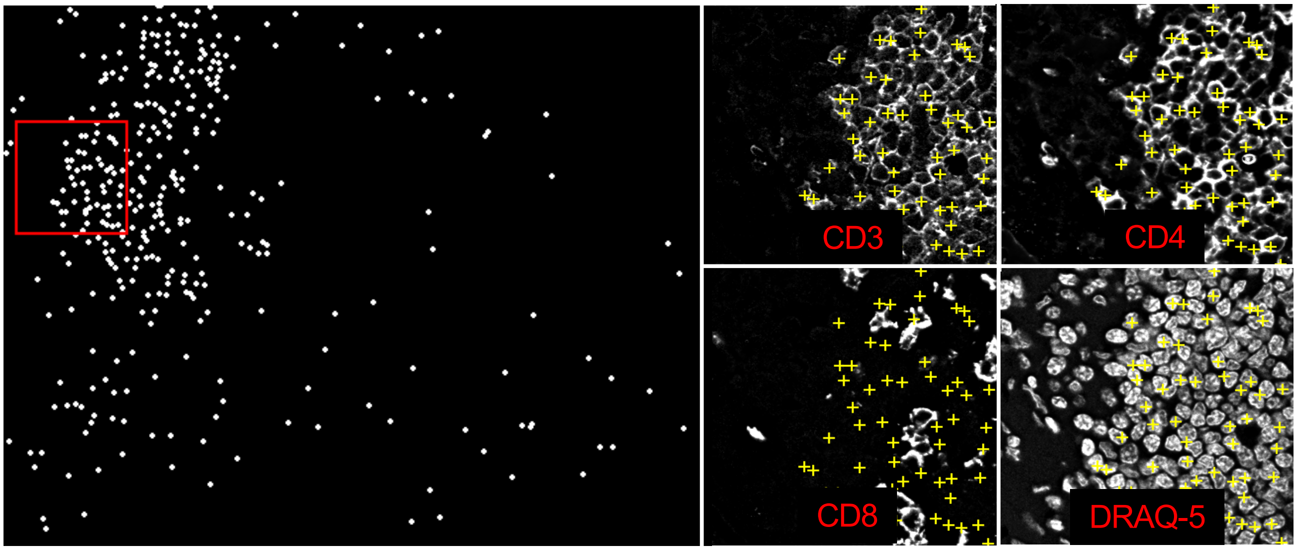

G. CD8+ T cell cluster

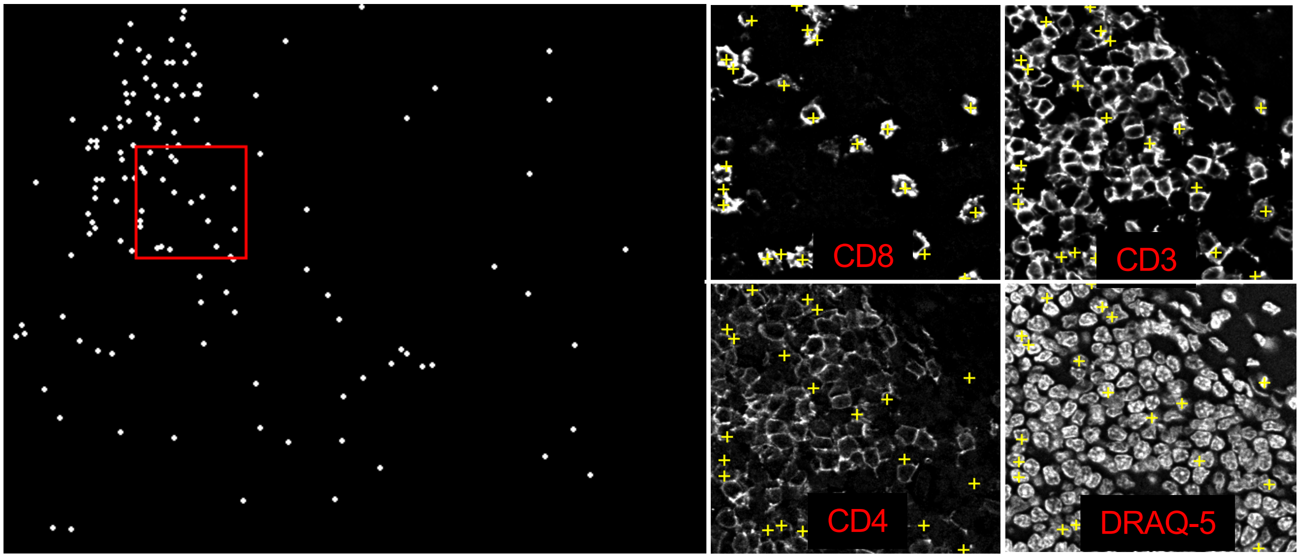

H. B cell cluster

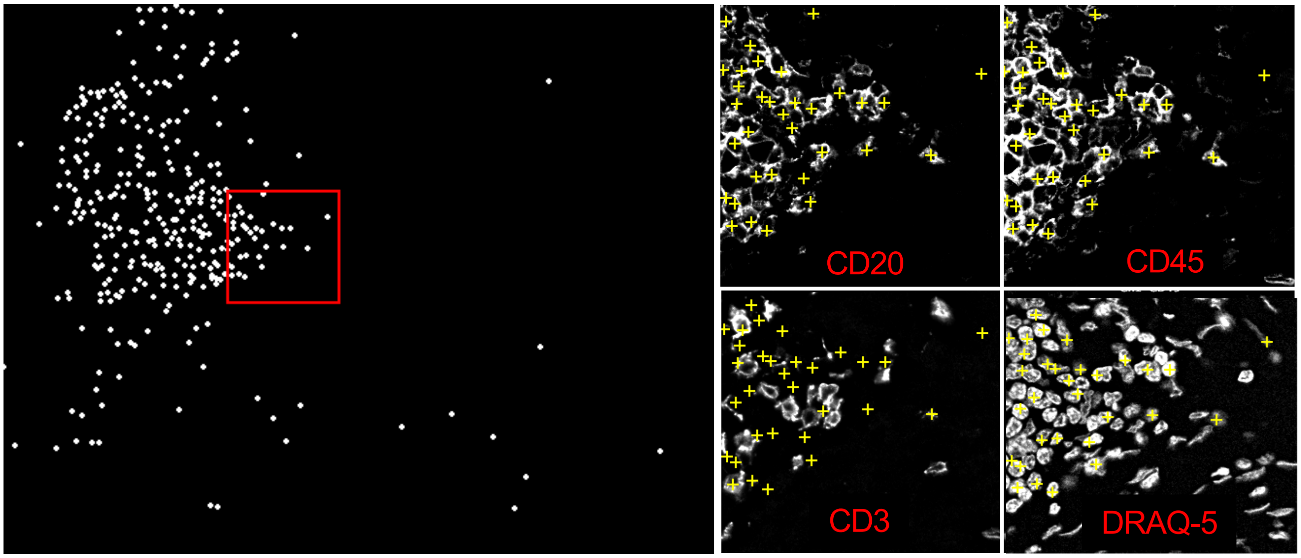

I. Vasculature cluster

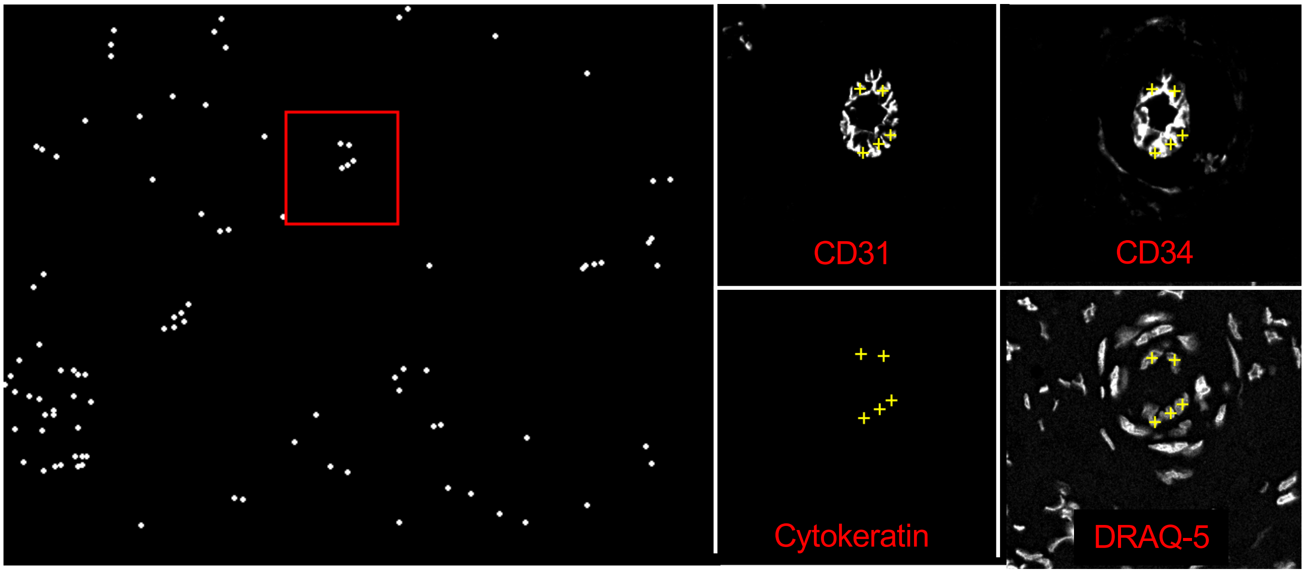

J. Plasma cell cluster

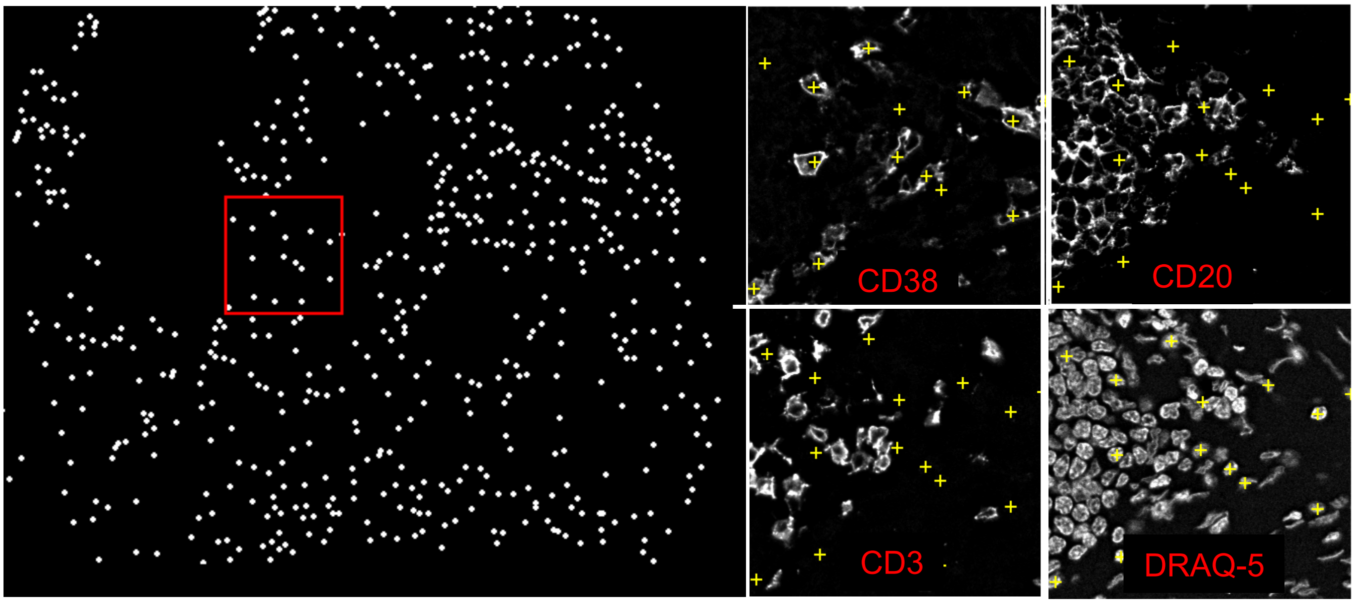

K. Treg cluster

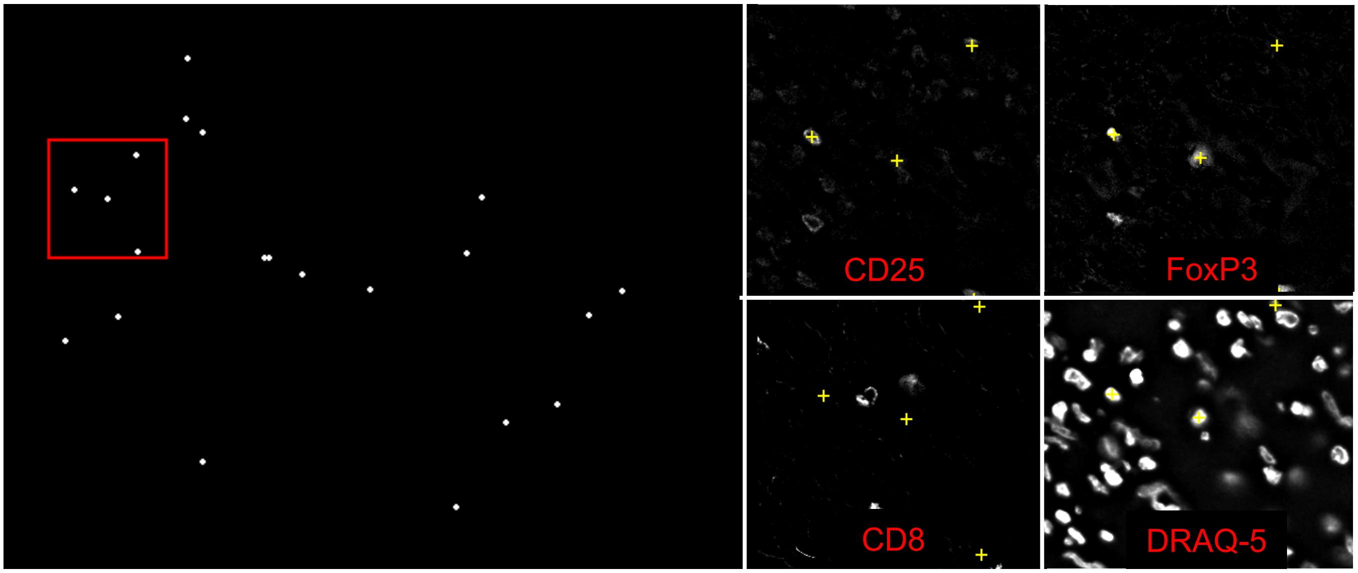

L. CD4+ T cell cluster

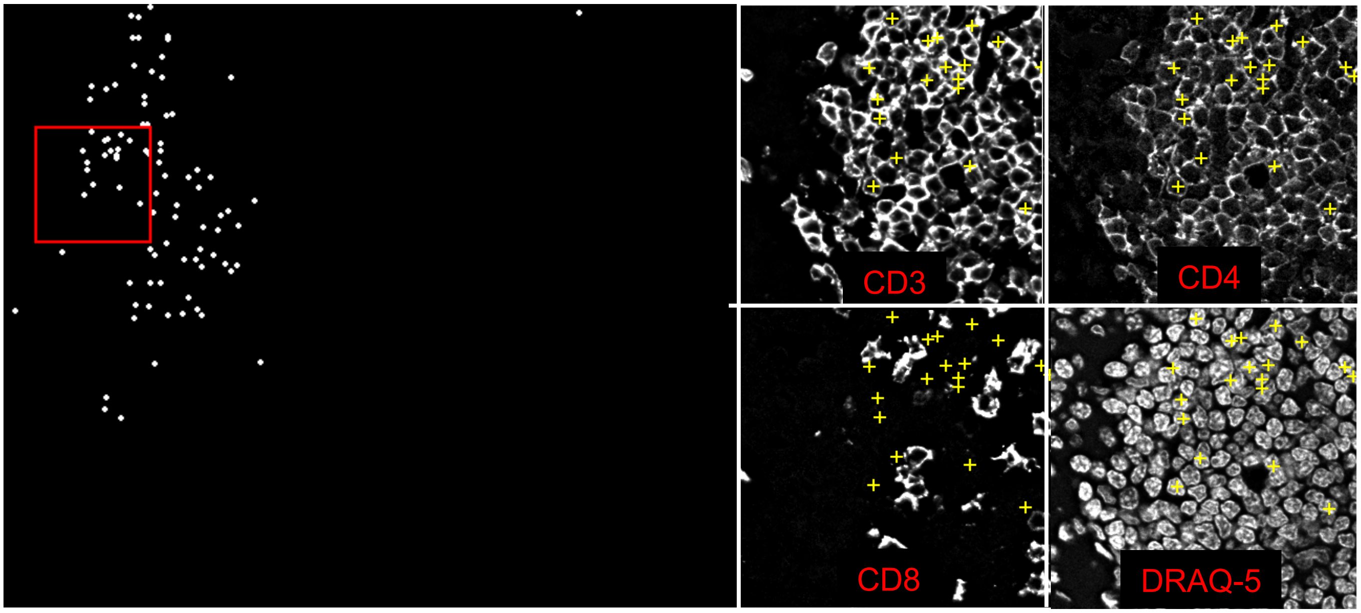

M. Adipocyte cluster

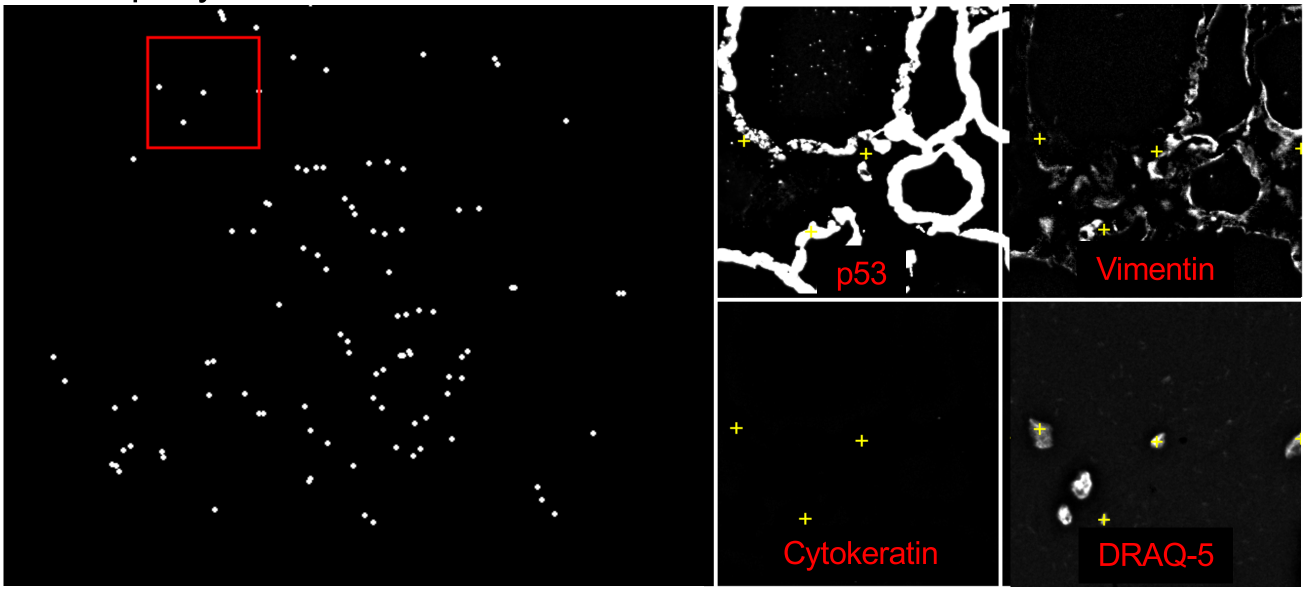

N. CD68+ macrophage cluster

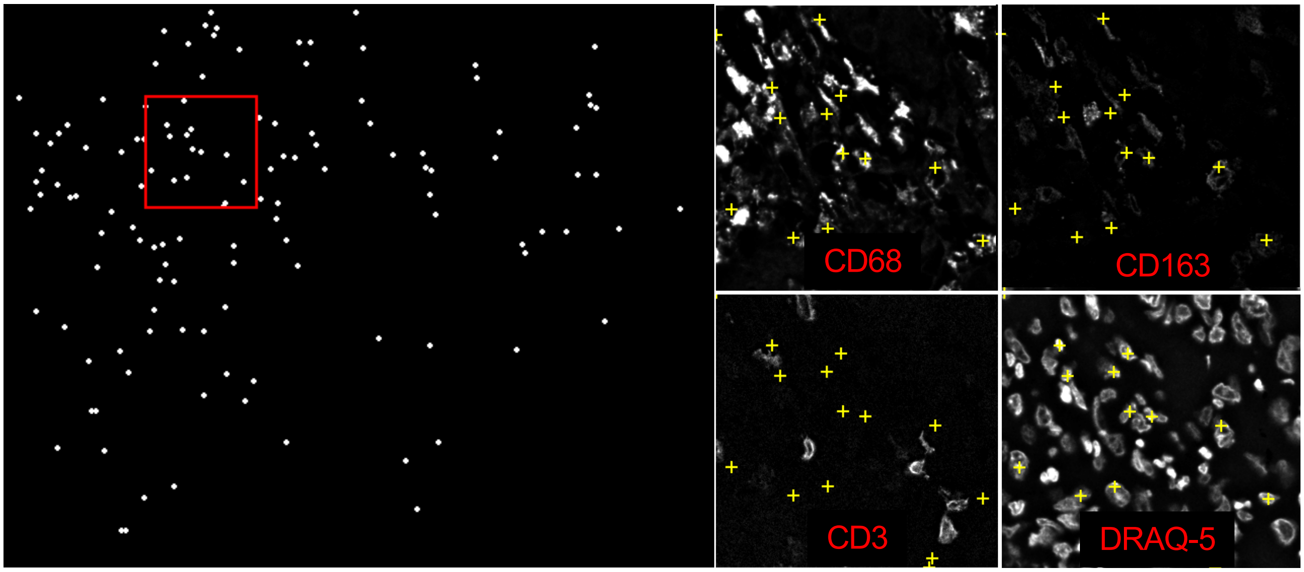

O. CD11b+CD68+ macrophage cluster

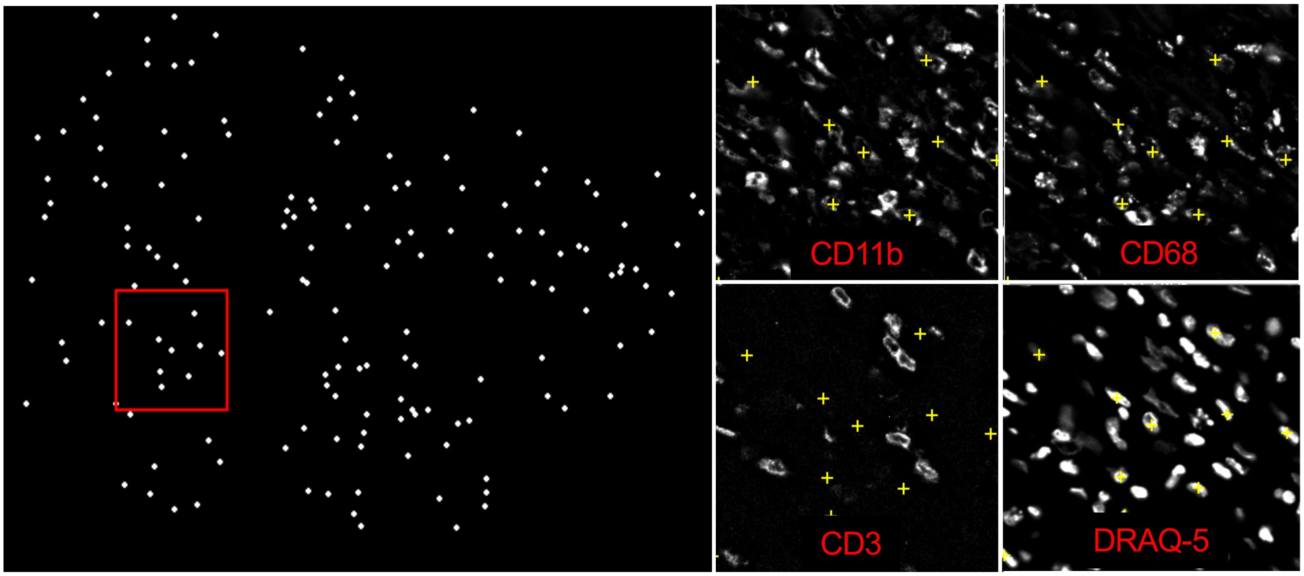

P. CD11c dendritic cell cluster

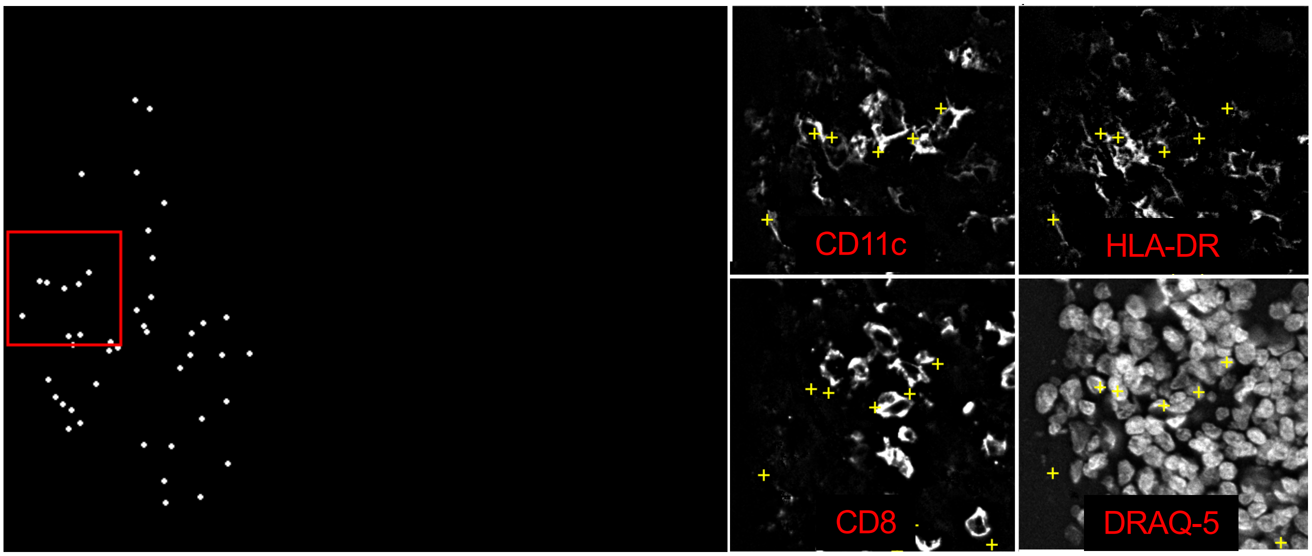

Q. NK cell cluster

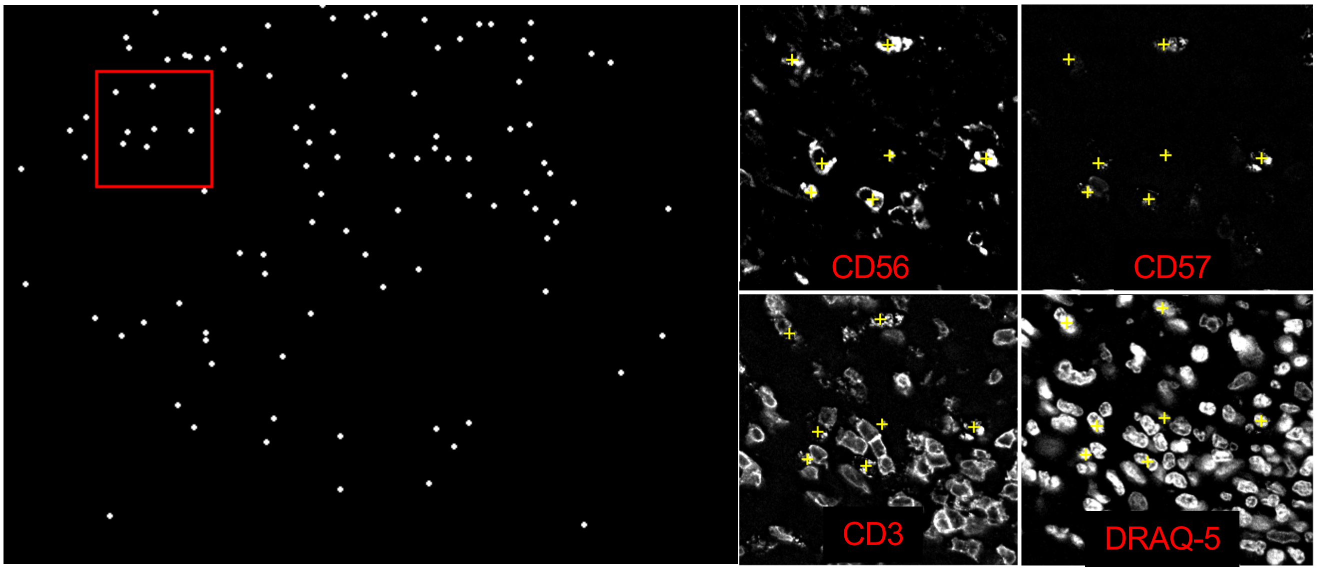

R. CD3+ T cell cluster

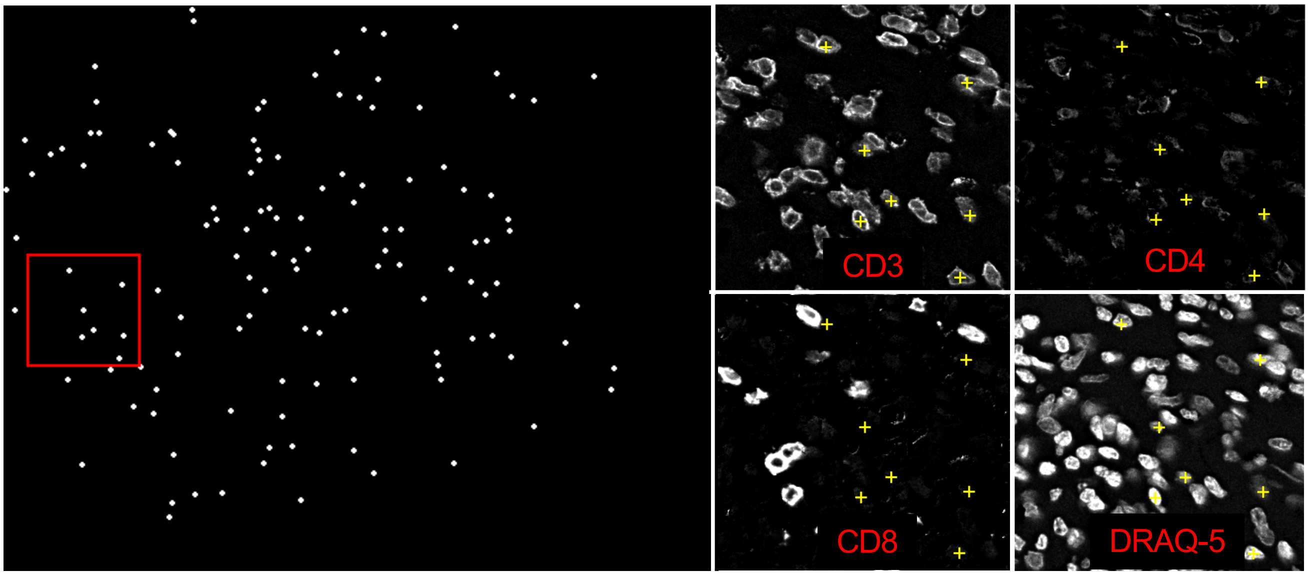

Data S5. Comparison of Unsupervised X-shift Clustering vs. Supervised Manual Gating of Cell Populations, Related to Figure 3 and STAR Methods. (A) Flow cytometry standard (FCS) files from segmented images were imported into CellEngine (www.cellengine.com). Gates were tailored individually for each file and cell population. (1) Cleanup gating: Nucleated cells were selected for by gating on cells positive for Hoechst (cycle 1) and DRAQ5 (cycle 23), and out-of-focus events were removed by gating on the focused Z planes. (2) FCS files were exported for X-shift unsupervised clustering in VorteX and (3) were further analyzed for major immune cell types in CellEngine. The gating strategy to identify T cells ($CD3^{+}$), cytotoxic T cells ($CD3^{+}CD8^{+}$), T helper cells ($CD3^{+}CD4^{+}$), Tregs ($CD3^{+}CD4^{+}CD25^{+}FOXP3^{+}$), B cells ($CD3\text{-}CD20^{+}$), NK cells ($CD3^{-}CD20^{-}CD56^{+}$), $CD68^{+}$ macrophages ($CD3^{-}CD20^{-}CD56^{-}CD68^{+}$), CD163 $^{+}$macrophages ($CD3^{-}CD20^{-}CD56^{-}CD163^{+}$), $CD68^{+}CD163^{+}$ double-positive macrophages ($CD3^{-}CD20^{-}CD56^{-}CD68^{+}CD163^{+}$), lymphatics ($CD3^{-}CD20^{-}CD56^{-}Podoplanin^{+}$), vasculature ($CD3^{-}CD20^{-}CD56^{-}CD31^{+}$), dendritic cells ($CD3^{-}CD20^{-}CD56^{-}CD11c^{+}$), granulocytes ($CD3^{-}CD20^{-}CD56^{-}CD15^{+}$), and plasma cells ($CD3^{-}CD20^{-}CD56^{-}CD68^{-}CD163^{-}CD38^{+}$) is shown. **(B)** Identification and quantification of major immune cell populations in CODEX data is comparable between supervised manual gating and unsupervised X-shift clustering. Numbers of cells per TMA spot and relative cell frequencies for manual gating in CellEngine (left columns, blue background) vs. unsupervised clustering in VorteX (right columns, white background) of selected major cell subsets. **(C)** Correlation of major immune cell populations quantified by supervised manual gating and unsupervised X-shift clustering. Correlation diagrams based on individual TMA cores for each cell population.

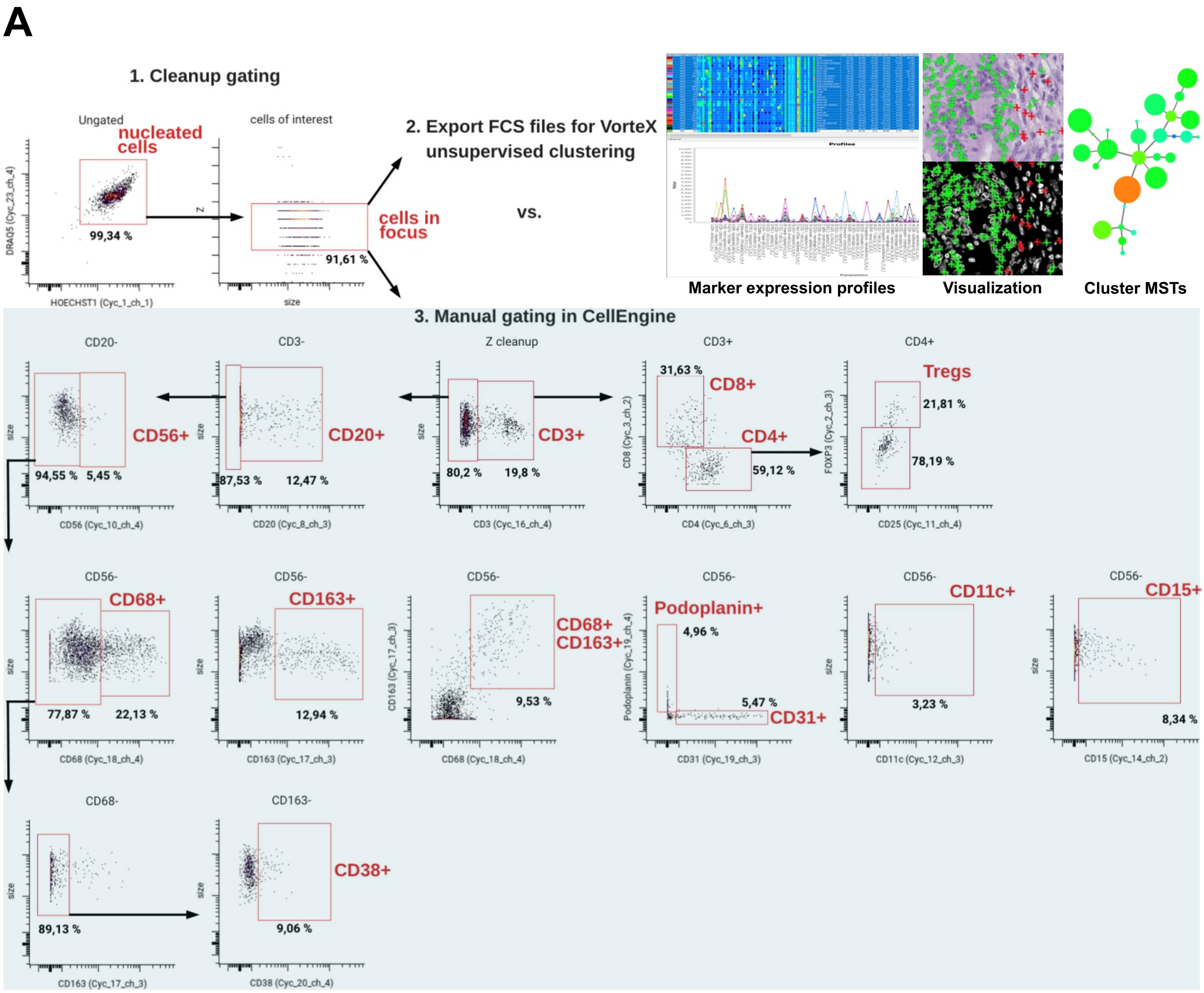
A
1. Cleanup gating
Ungated
nucleated cells
99,34 %
DRAQ5 (Cyc_23_ch_4)
HOECHST1 (Cyc_1_ch_1)
cells of interest
cells in focus
91,61 %
Z
size
2. Export FCS files for VorteX unsupervised clustering
vs.
Marker expression profiles
Visualization
Cluster MSTs
3. Manual gating in CellEngine
CD20-
CD56+
94,55 %
5,45 %
CD56 (Cyc_10_ch_4)
CD3-
CD20+
87,53 %
12,47 %
CD20 (Cyc_8_ch_3)
Z cleanup
CD3+
80,2 %
19,8 %
CD3 (Cyc_16_ch_4)
CD3+
31,63 %
CD8+
CD4+
59,12 %
CD8 (Cyc_3_ch_2)
CD4 (Cyc_6_ch_3)
CD4+
Tregs
21,81 %
78,19 %
FOXP3 (Cyc_2_ch_3)
CD25 (Cyc_11_ch_4)
CD56-
CD68+
77,87 %
22,13 %
CD68 (Cyc_18_ch_4)
CD56-
CD163+
12,94 %
CD163 (Cyc_17_ch_3)
CD56-
CD68+ CD163+
9,53 %
CD163 (Cyc_17_ch_3)
CD68 (Cyc_18_ch_4)
CD56-
Podoplanin+
4,96 %
5,47 %
CD31+
Podoplanin (Cyc_19_ch_4)
CD31 (Cyc_19_ch_3)
CD56-
CD11c+
3,23 %
CD11c (Cyc_12_ch_3)
CD56-
CD15+
8,34 %
CD15 (Cyc_14_ch_2)
CD68-
89,13 %
CD163 (Cyc_17_ch_3)
CD163-
CD38+
9,06 %
CD38 (Cyc_20_ch_4)
size

B

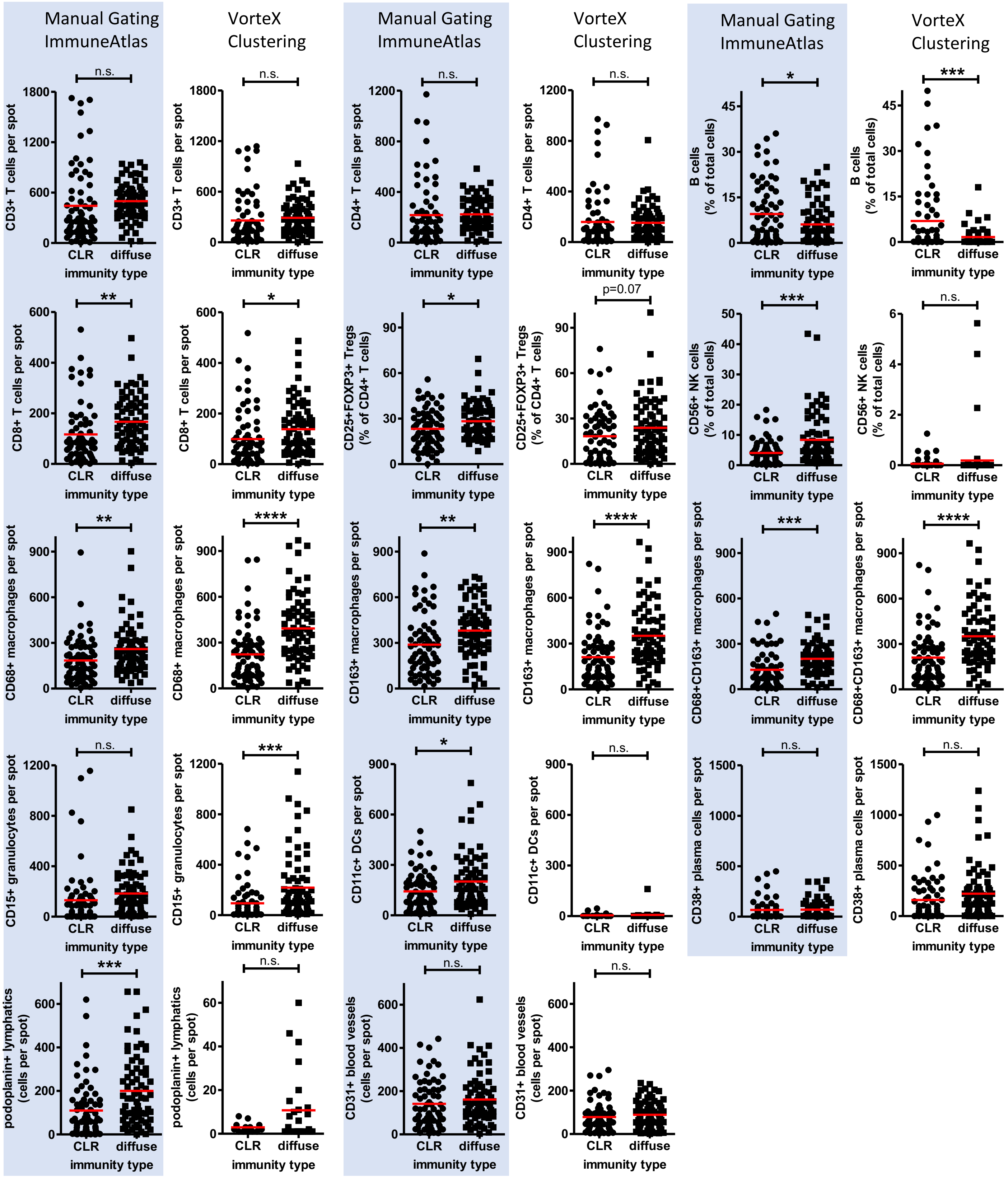

Manual Gating ImmuneAtlas
VorteX Clustering
Manual Gating ImmuneAtlas
VorteX Clustering
Manual Gating ImmuneAtlas
VorteX Clustering
CD3+ T cells per spot
CD4+ T cells per spot
B cells (% of total cells)
CD8+ T cells per spot
CD25+FOXP3+ Tregs (% of CD4+ T cells)
CD56+ NK cells (% of total cells)
CD68+ macrophages per spot
CD163+ macrophages per spot
CD68+CD163+ macrophages per spot
CD15+ granulocytes per spot
CD11c+ DCs per spot
CD38+ plasma cells per spot
podoplanin+ lymphatics (cells per spot)
CD31+ blood vessels (cells per spot)
CLR
diffuse
immunity type
n.s.
*
**
***
****
p=0.07

C

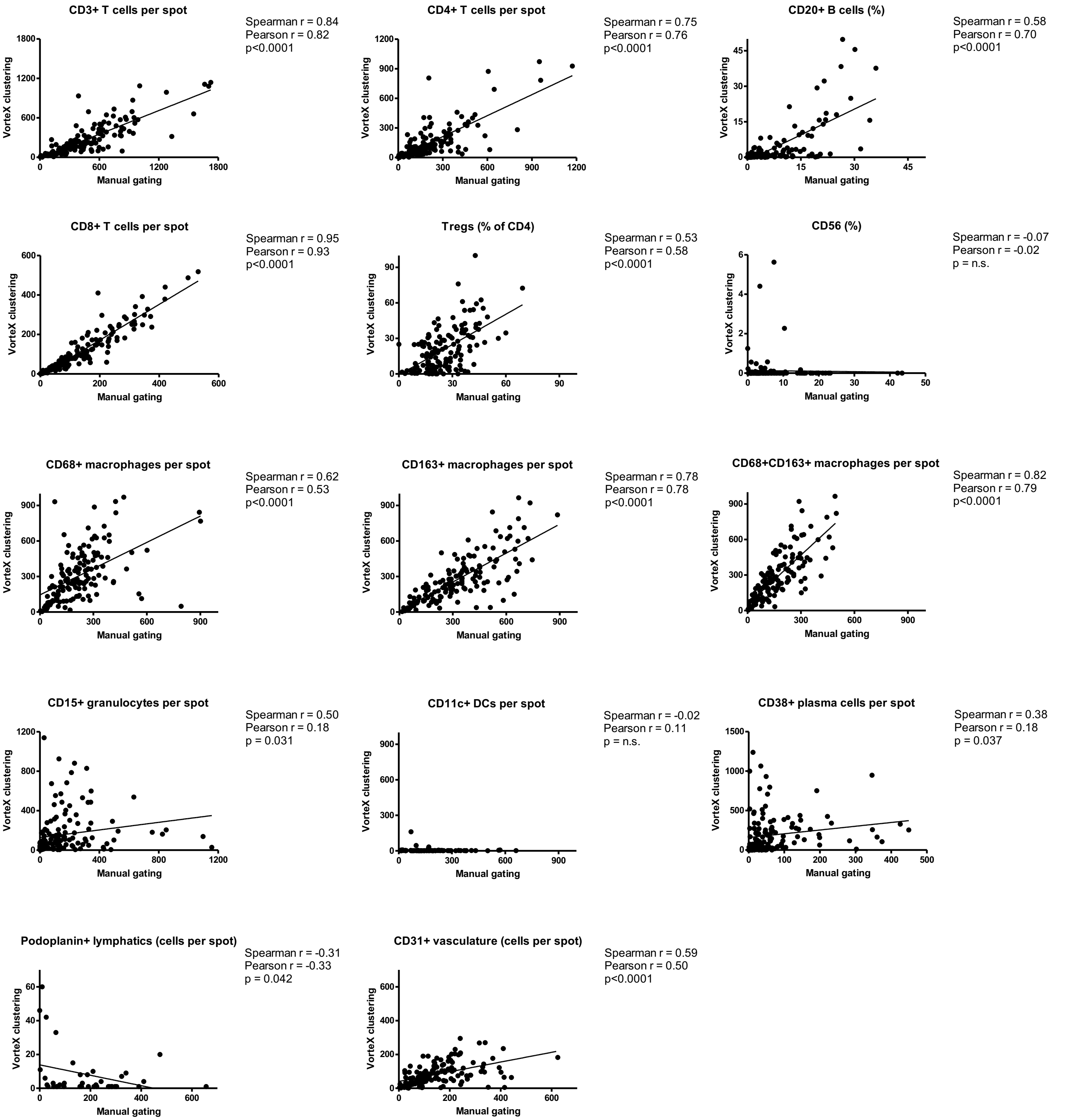
CD3+ T cells per spot
Spearman r = 0.84
Pearson r = 0.82
p<0.0001
CD4+ T cells per spot
Spearman r = 0.75
Pearson r = 0.76
p<0.0001
CD20+ B cells (%)
Spearman r = 0.58
Pearson r = 0.70
p<0.0001
CD8+ T cells per spot
Spearman r = 0.95
Pearson r = 0.93
p<0.0001
Tregs (% of CD4)
Spearman r = 0.53
Pearson r = 0.58
p<0.0001
CD56 (%)
Spearman r = -0.07
Pearson r = -0.02
p = n.s.
CD68+ macrophages per spot
Spearman r = 0.62
Pearson r = 0.53
p<0.0001
CD163+ macrophages per spot
Spearman r = 0.78
Pearson r = 0.78
p<0.0001
CD68+CD163+ macrophages per spot
Spearman r = 0.82
Pearson r = 0.79
p<0.0001
CD15+ granulocytes per spot
Spearman r = 0.50
Pearson r = 0.18
p = 0.031
CD11c+ DCs per spot
Spearman r = -0.02
Pearson r = 0.11
p = n.s.
CD38+ plasma cells per spot
Spearman r = 0.38
Pearson r = 0.18
p = 0.037
Podoplanin+ lymphatics (cells per spot)
Spearman r = -0.31
Pearson r = -0.33
p = 0.042
CD31+ vasculature (cells per spot)
Spearman r = 0.59
Pearson r = 0.50
p<0.0001
VorteX clustering
Manual gating

Data S6. Voronoi Diagrams and Distribution of the 28 CRC Clusters, and Distribution of the Eight Merged Immune Clusters, Related to Figure 3. (A) Five representative Voronoi diagrams of the 28 CRC clusters are shown for each patient group. **(B)** The frequencies of the 28 CRC clusters for all patients and for each group are listed. Significant differences are highlighted in bold (Mann-Whitney test). Data represent mean values from four biological replicates (TMA cores) per patient. **(C)** Distributions of the 28 clusters in all CRC patients and CLR and DII groups. **(D)** Distributions of the 28 clusters in each individual patient. **(E)** The frequencies of the eight merged immune clusters for all patients and for each group are listed. Significant differences are highlighted in bold (Mann-Whitney test). **(F)** Cell numbers (left panel) and distributions of the eight merged clusters (right panel) in each individual patient.

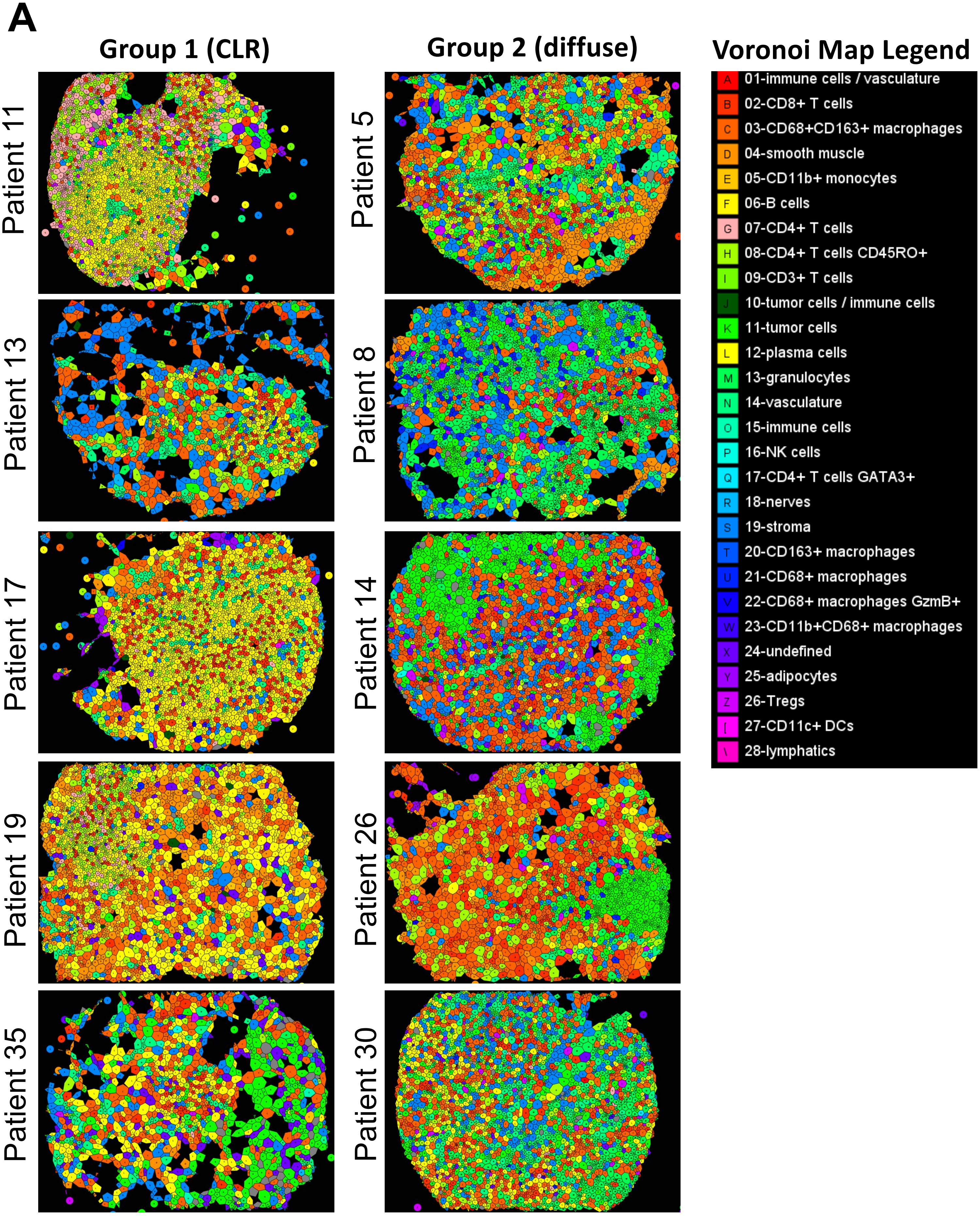
A
Group 1 (CLR)
Group 2 (diffuse)
Voronoi Map Legend
Patient 11
Patient 13
Patient 17
Patient 19
Patient 35
Patient 5
Patient 8
Patient 14
Patient 26
Patient 30
A 01-immune cells / vasculature
B 02-CD8+ T cells
C 03-CD68+CD163+ macrophages
D 04-smooth muscle
E 05-CD11b+ monocytes
F 06-B cells
G 07-CD4+ T cells
H 08-CD4+ T cells CD45RO+
I 09-CD3+ T cells
J 10-tumor cells / immune cells
K 11-tumor cells
L 12-plasma cells
M 13-granulocytes
N 14-vasculature
O 15-immune cells
P 16-NK cells
Q 17-CD4+ T cells GATA3+
R 18-nerves
S 19-stroma
T 20-CD163+ macrophages
U 21-CD68+ macrophages
V 22-CD68+ macrophages GzmB+
W 23-CD11b+CD68+ macrophages
X 24-undefined
Y 25-adipocytes
Z 26-Tregs
[27-CD11c+ DCs
\ 28-lymphatics

B

	All CRC Patients (n=251,028 cells) Mean % ± SD	Group 1 (CLR) (n=107,966 cells) Mean % ± SD	Group 2 (Diffuse) (n=143,062 cells) Mean % ± SD	Mann-Whitney Test p-value
Tumor cells	19.3 ± 14.4%	19.3 ± 18.9%	19.3 ± 9.1%	0.3141
CD68+CD163+ macrophages	15.6 ± 7.1%	13.4 ± 6.4%	17.6 ± 7.2%	0.0831
Smooth muscle	10.2 ± 8.4%	9.5 ± 7.0%	10.9 ± 9.8%	0.9605
**Granulocytes**	**8.5 ± 8.2%**	**5.8 ± 5.3%**	**11.0 ± 9.7%**	**0.0496**
Stroma	8.0 ± 4.3%	7.9 ± 4.7%	8.0 ± 4.1%	0.9080
CD4+ T cells CD45RO+	7.0 ± 5.0%	7.5 ± 5.5%	6.6 ± 4.6%	0.7043
CD8+ T cells	7.0 ± 4.3%	6.0 ± 2.9%	7.9 ± 5.3%	0.6322
**B cells**	**5.1 ± 7.5%**	**8.8 ± 9.4%**	**1.7 ± 2.3%**	**0.0009**
Vasculature	4.5 ± 2.2%	4.8 ± 2.4%	4.2 ± 1.9%	0.4780
Plasma cells	3.3 ± 4.0%	3.8 ± 4.0%	2.9 ± 4.2%	0.2038
Undefined	2.9 ± 3.8%	2.9 ± 2.9%	3.0 ± 4.6%	0.2283
**Immune cells (mixed)**	**1.5 ± 4.5%**	**3.0 ± 6.2%**	**0.1 ± 0.2%**	**0.0306**
**Tregs**	**1.1 ± 0.7%**	**0.9 ± 0.6%**	**1.4 ± 0.7%**	**0.0218**
**CD4+ T cells**	**1.0 ± 2.5%**	**1.8 ± 3.4%**	**0.3 ± 0.7%**	**0.0167**
Immmune cells / vasculature	1.0 ± 1.8%	1.5 ± 2.5%	0.5 ± 0.8%	0.1923
**Adipocytes**	**0.8 ± 1.1%**	**1.2 ± 1.0%**	**0.5 ± 1.1%**	**0.0004**
**CD68+ macrophages**	**0.8 ± 1.0%**	**0.5 ± 0.5%**	**1.1 ± 1.3%**	**0.0333**
**Tumor cells / immune cells**	**0.6 ± 2.0%**	**0.5 ± 1.0%**	**0.8 ± 2.6%**	**0.0127**
**CD11b+CD68+ macrophages**	**0.5 ± 1.4%**	**0.2 ± 0.1%**	**0.9 ± 2.0%**	**0.0459**
CD11b+ monocytes	0.3 ± 1.5%	0.0 ± 0.1%	0.6 ± 2.1%	0.4485
CD11c+ DCs	0.2 ± 0.4%	0.2 ± 0.2%	0.2 ± 0.5%	0.1326
Nerves	0.2 ± 0.3%	0.2 ± 0.3%	0.2 ± 0.3%	0.9605
NK cells	0.1 ± 0.4%	0.0 ± 0.1%	0.2 ± 0.5%	0.5323
Lymphatics	0.1 ± 0.3%	0.0 ± 0.1%	0.2 ± 0.4%	0.2452
CD3+ T cells	0.1 ± 0.2%	0.0 ± 0.1%	0.1 ± 0.3%	0.6111
CD68+ macrophages GzmB+	0.1 ± 0.1%	0.1 ± 0.1%	0.1 ± 0.1%	0.7965
CD4+ T cells GATA3+	0.0 ± 0.2%	0.1 ± 0.2%	0.0 ± 0.0%	0.3029
CD163+ macrophages	0.0 ± 0.0%	0.0 ± 0.1%	0.0 ± 0.0%	0.4905

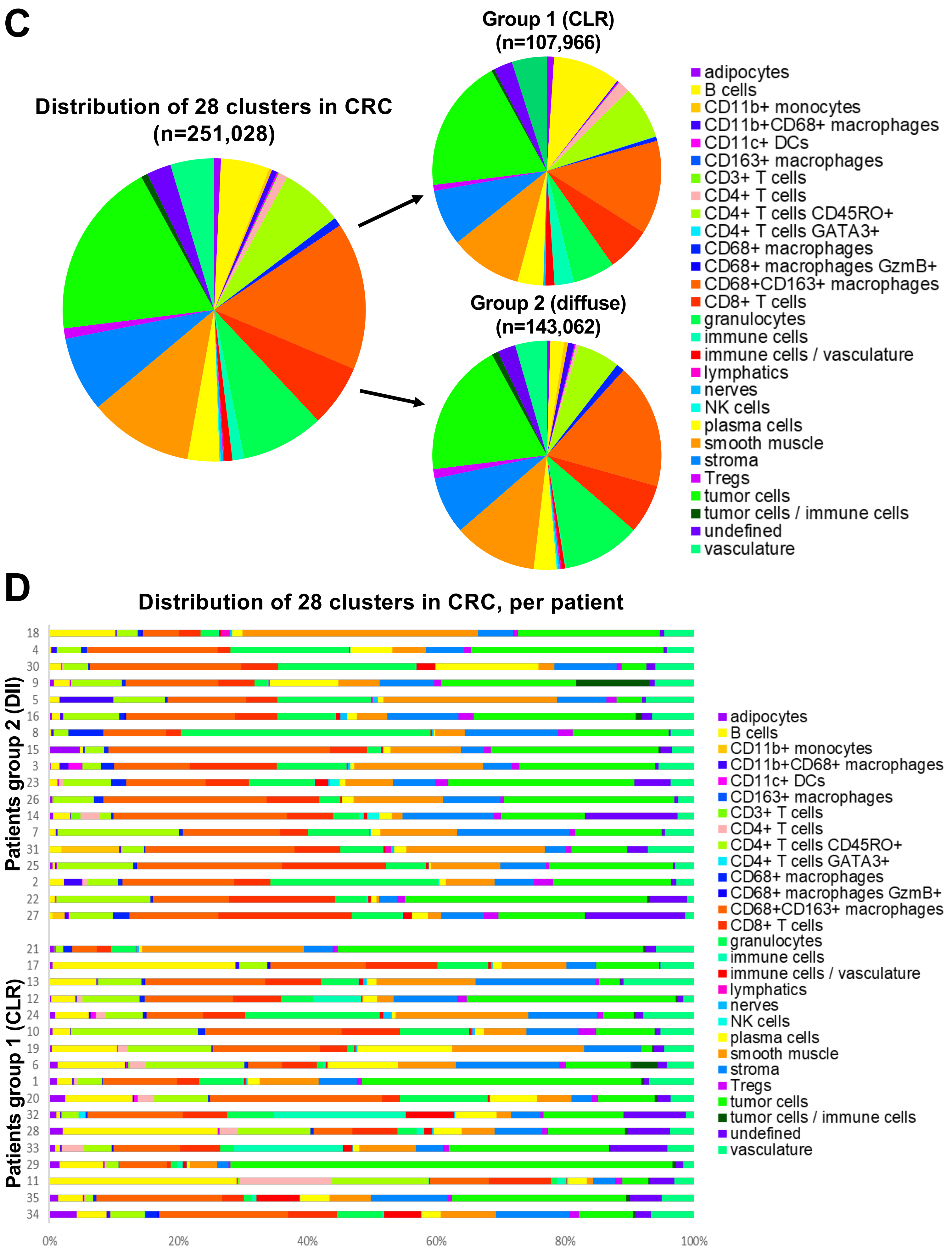
C
Distribution of 28 clusters in CRC
(n=251,028)
Group 1 (CLR)
(n=107,966)
Group 2 (diffuse)
(n=143,062)
adipocytes
B cells
CD11b+ monocytes
CD11b+CD68+ macrophages
CD11c+ DCs
CD163+ macrophages
CD3+ T cells
CD4+ T cells
CD4+ T cells CD45RO+
CD4+ T cells GATA3+
CD68+ macrophages
CD68+ macrophages GzmB+
CD68+CD163+ macrophages
CD8+ T cells
granulocytes
immune cells
immune cells / vasculature
lymphatics
nerves
NK cells
plasma cells
smooth muscle
stroma
Tregs
tumor cells
tumor cells / immune cells
undefined
vasculature
D
Distribution of 28 clusters in CRC, per patient
Patients group 2 (DII)
18
4
30
9
5
16
8
15
3
23
26
14
7
31
25
2
22
27
Patients group 1 (CLR)
21
17
13
12
24
10
19
6
1
20
32
28
33
29
11
35
34
0%
20%
40%
60%
80%
100%

E

	All CRC Patients (n=131,329 immune cells) Mean % ± SD	Group 1 (CLR) (n=56,343 immune cells) Mean % ± SD	Group 2 (Diffuse) (n=74,986 immune cells) Mean % ± SD	Mann-Whitney Test p-value
**B cells**	**9.5 ± 12.2%**	**15.3 ± 13.5%**	**4.0 ± 7.9%**	**0.0008**
**Macrophages**	**33.5 ± 13.2%**	**28.4 ± 11.2%**	**38.4 ± 13.5%**	**0.0496**
Tregs	2.2 ± 1.3%	1.8 ± 1.3%	2.6 ± 1.2%	0.0535
CD11c+ DCs	0.4 ± 0.8%	0.4 ± 0.4%	0.4 ± 1.1%	0.0512
CD4+ T cells	14.7 ± 9.0%	16.2 ± 9.5%	13.3 ± 8.6%	0.3641
CD8+ T cells	13.1 ± 7.3%	11.2 ± 3.9%	15.0 ± 9.2%	0.4187
NK cells	0.4 ± 1.0%	0.1 ± 0.1%	0.8 ± 1.4%	0.7197
Other immune cells	26.3 ± 14.4%	26.7 ± 13.3%	25.9 ± 15.7%	0.6322

F

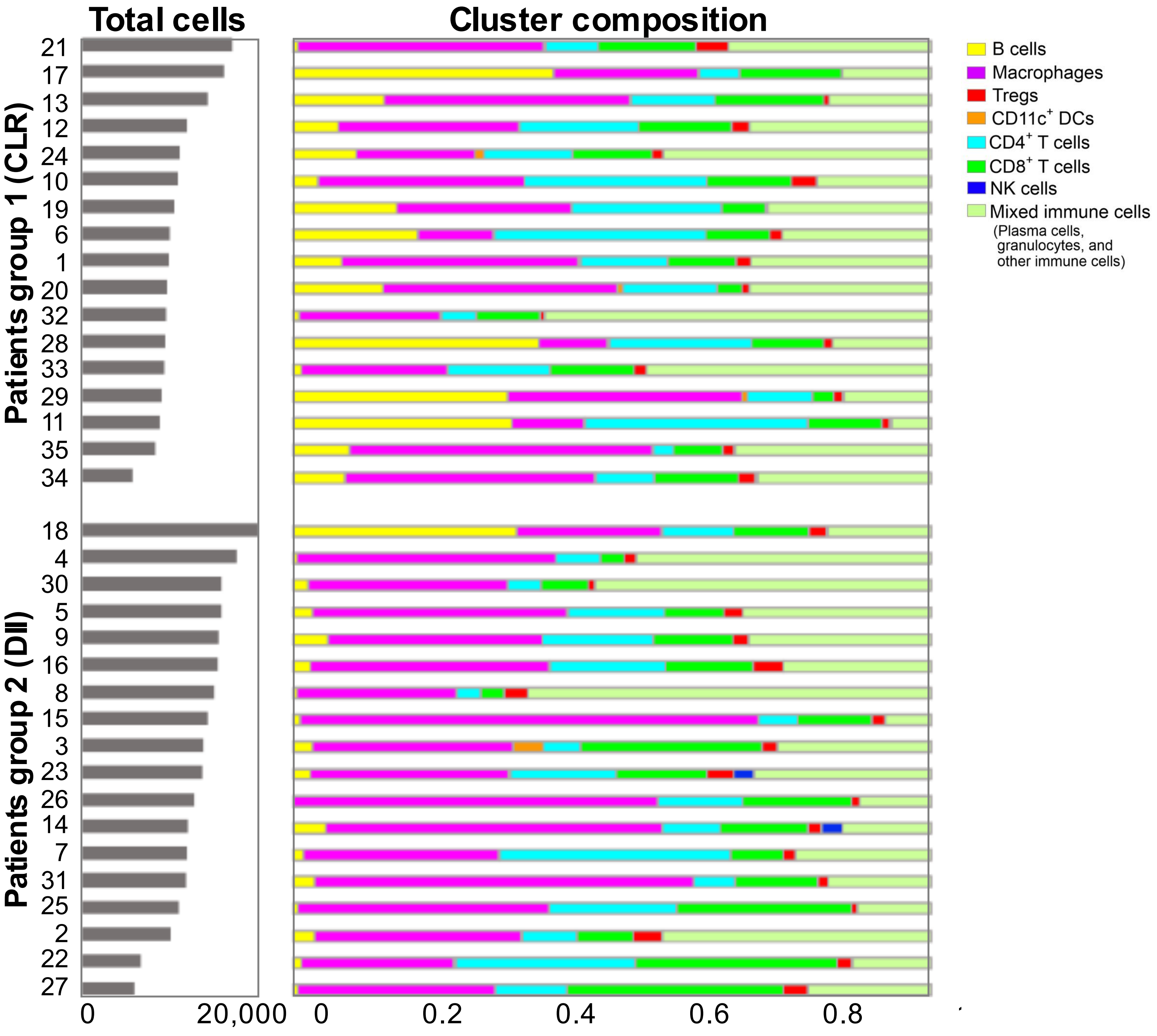

Data S7. Tensor Decomposition, Related to Figure 5.

We motivate our use of tensor methods for describing differences in the variation across patients' joint CN-CT compositions by discussing the limitations of traditional PCA for this purpose. One possibility for describing the differences, between patient groups, in variation across patients' joint CN-CT compositions, would have been to first perform PCA (by flattening each patient's 2D matrix to a 1D vector), and subsequently describe how the identified axes were different. However, this would have eliminated the information that CNs and CTs form two distinct but coupled views of the iTME. This coupling corresponds exactly to the fact that the underlying biological programs drive multiple distinct CTs to be found together in multiple distinct CNs. For example, multiple CTs might share combinations of cytokine receptors, and cytokine gradients might promote combinations of CNs.

An example which illustrates how underlying biology could give rise to the tensor decomposition output is depicted as a schematic in the **Figure** below: (1) The tissue is formed by the interaction of CN 'recruitment factors' (for example, cytokines) shared by multiple CNs to recruit cell types by interacting with cognate 'cellular localization factors' (for example, cytokine receptors) shared by multiple cell types (**Panel 1**, top aspect). The term factor should be viewed in a statistical sense and could represent more complicated programs than a single ligand or receptor. Different factors can interact to different extents (**Panel 1**, lower aspect). (2) Different interacting pairs of recruitment and localization factors are found together in the tissue, giving rise to the observed distribution of CNs and cell types (**Panel 2**). In the left region, the blue and red CNs share a recruitment factor (heart-shaped indentation), so share a common cell type (green) with a cognate localization factor (heart). In the right region, the orange and the gray cells share a localization factor (circle), so are found in multiple CNs. The green CN uses multiple recruitment factors, one shared with the yellow CN. Distinct interacting pairs of recruitment and localization factors co-occur across patients (red and blue found together, and yellow and green found together), each co-occurring collection of interacting pairs corresponding to a tissue module. These recruitment and localization factors are inferred from the tensor decomposition output, visualized as tissue modules comprised of CN modules and cell type (CT) modules, with interactions between them represented as edges (**Panel 3**). Note that there is a common collection of CT modules and CN modules that are present to different extents in each tissue module. The contribution of each CN module and CT module to each tissue module is represented by its shading (**Panel 3**). In tissue module 1 (top box), the CN module in the first row is interpreted as the recruitment factor with a circular indentation. This is because it contains yellow and green CNs, and there is a strong edge with the CT module containing the orange and grey cell types, and a weak edge with the CT module containing the blue cell type. The CN module with just the green CN (row 2) is interpreted as the recruitment factor with the square indentation. This is because that CN module does not contain any other CNs and has only one edge with one CT module containing the blue cell type. Since the red and green CNs are not found in the same patients, the CN module with the red and blue CNs and its cognate CT module with just the green cell type are faint in tissue module 1 and form tissue module 2. Note that the CN modules and the cell type modules are identified by their mutual dependence.

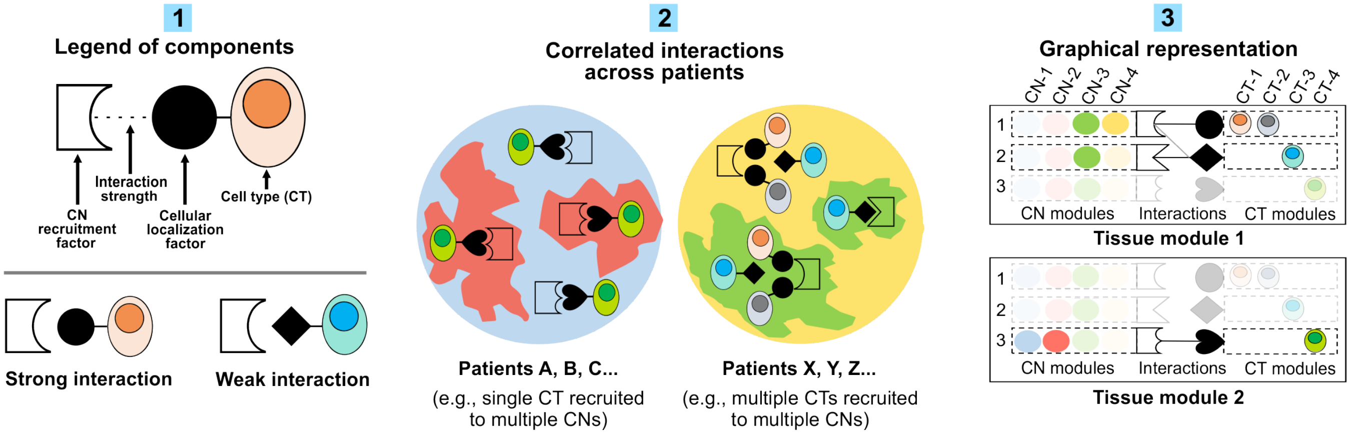

Schematic illustrating the interpretation of the tensor decomposition output. **(1)** Legend of components: A CN module corresponds to a cell recruitment program utilized by the CNs comprising that module, and a CT module corresponds to a cell type localization program utilized by the cell types comprising that module. Different pairs of recruitment programs and localization programs interact to different strengths. **(2)** Different pairs of interacting recruitment programs and localization programs co-occur to form the tissue through balanced interactions between recruitment and localization factors. These combinations yield similar combinations of CNs and cell types within them across patients. **(3)** Graphical representation of tissue modules corresponding to combinations of interacting pairs, indicated by edges, of CN modules (left column) and CT modules (right column). CN modules and CT modules are common across both tissue modules. In each tissue module, the transparency of each CN module and CT module corresponds to the weight of the maximum edge of which it is part, i.e. indicating its contribution to that tissue module.
